# piRNA PROPER Suppresses *DUSP1* Translation by Targeting *N* ^6^‐Methyladenosine‐Mediated RNA Circularization to Promote Oncogenesis of Prostate Cancer

**DOI:** 10.1002/advs.202402954

**Published:** 2024-07-04

**Authors:** Shuai Ben, Zhutao Ding, Junyi Xin, Feng Li, Yifei Cheng, Silu Chen, Lulu Fan, Qin Zhang, Shuwei Li, Mulong Du, Zhengdong Zhang, Gong‐Hong Wei, Gong Cheng, Meilin Wang

**Affiliations:** ^1^ The Affiliated Suzhou Hospital of Nanjing Medical University Suzhou Municipal Hospital Gusu School Nanjing Medical University Suzhou 215002 China; ^2^ Department of Environmental Genomics Jiangsu Key Laboratory of Cancer Biomarkers Prevention and Treatment Collaborative Innovation Center for Cancer Personalized Medicine School of Public Health Nanjing Medical University Nanjing 211166 China; ^3^ Department of Genetic Toxicology The Key Laboratory of Modern Toxicology of Ministry of Education Center for Global Health School of Public Health Nanjing Medical University Nanjing 211166 China; ^4^ Department of Ophthalmology Shanghai General Hospital School of Medicine Shanghai Jiao Tong University Shanghai 200080 China; ^5^ Department of Bioinformatic School of Biomedical Engineering and Informatics Nanjing Medical University Nanjing Jiangsu 211166 China; ^6^ State Key Laboratory of Reproductive Medicine Nanjing Medical University Nanjing Jiangsu 211100 China; ^7^ Disease Networks Research Unit Faculty of Biochemistry and Molecular Medicine & Biocenter Oulu University of Oulu Oulu 90220 Finland; ^8^ Department of Biostatistics Center for Global Health School of Public Health Nanjing Medical University Nanjing 211166 China; ^9^ Fudan University Shanghai Cancer Center & MOE Key Laboratory of Metabolism and Molecular Medicine and Department of Biochemistry and Molecular Biology of School of Basic Medical Sciences Shanghai Medical College of Fudan University Shanghai 200032 China; ^10^ Department of Urology The First Affiliated Hospital of Nanjing Medical University & Jiangsu Province People's Hospital Nanjing 210029 China

**Keywords:** *N* ^6^‐methyladenosine, PIWI‐interacting RNA, Prostate cancer, Single nucleotide polymorphism

## Abstract

Genetic and epigenetic alterations occur in many physiological and pathological processes. The existing knowledge regarding the association of PIWI‐interacting RNAs (piRNAs) and their genetic variants on risk and progression of prostate cancer (PCa) is limited. In this study, three genome‐wide association study datasets are combined, including 85,707 PCa cases and 166,247 controls, to uncover genetic variants in piRNAs. Functional investigations involved manipulating piRNA expression in cellular and mouse models to study its oncogenetic role in PCa. A specific genetic variant, rs17201241 is identified, associated with increased expression of PROPER (piRNA overexpressed in prostate cancer) in tumors and are located within the gene, conferring an increased risk and malignant progression of PCa. Mechanistically, PROPER coupled with YTHDF2 to recognize *N* ^6^‐methyladenosine (m^6^A) and facilitated RNA‐binding protein interactions between EIF2S3 at 5′‐untranslated region (UTR) and YTHDF2/YBX3 at 3′‐UTR to promote *DUSP1* circularization. This m^6^A‐dependent mRNA‐looping pattern enhanced *DUSP1* degradation and inhibited *DUSP1* translation, ultimately reducing DUSP1 expression and promoting PCa metastasis via the p38 mitogen‐activated protein kinase (MAPK) signaling pathway. Inhibition of PROPER expression using antagoPROPER effectively suppressed xenograft growth, suggesting its potential as a therapeutic target. Thus, targeting piRNA PROPER‐mediated genetic and epigenetic fine control is a promising strategy for the concurrent prevention and treatment of PCa.

## Graphical abstract

This study identified a prostate cancer risk‐associated SNP, rs17201241, located within an oncogenic piRNA called PROPER. PROPER‐YTHDF2/YBX3/EIF2S3‐DUSP1 signaling axis guides *DUSP1* degradation and translation control based on an m^6^A methylation‐regulated mRNA‐looping.

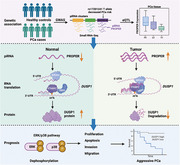

## 1 Introduction

Prostate cancer (PCa) is the second most common cancer in males and the fifth leading cause of cancer death worldwide.^[^ ^1^ ^]^ Emerging evidence has demonstrated that genetic and epigenetic events, including genetic variants,^[^ ^2^, ^3^ ^]^ non‐coding RNAs (ncRNAs),^[^ ^4^, ^5^ ^]^ and *N* ^6^‐methyladenosine (m^6^A) methylation,^[^ ^6^ ^]^ contribute to the development and progression of PCa. Genome‐wide association studies (GWASs) including our previous reports coupled with large‐scale collaborative replication efforts in Asians have identified over 200 genetic variants that contribute to the development of PCa.^[^ ^7^, ^8^ ^]^ As with most other diseases and traits, these risk‐associated single nucleotide polymorphisms (SNPs) map primarily to noncoding regions of the genome.

Among different types of ncRNAs, PIWI‐interacting RNAs (piRNAs) are a new type of small ncRNAs that were initially identified with high abundance in germline cells, showing most well‐known and critical regulatory functions in silencing transposons to maintain genome integrity in gametogenesis and fertility.^[^ ^9^, ^10^ ^]^ Nevertheless, several studies have reported additional regulatory roles of piRNAs in cancers of different origins, such as germline cells or somatic tissues. For instance, piRNAs such as piR‐823, piR‐001773, and piR‐017184 were found to play critical roles in epigenetic regulation of cancer progression.^[^ ^11^, ^12^ ^]^ Our previous study showed that piRNAs were abundantly expressed and harbored a large number of genetic variants in various tumors, suggesting their potential roles in carcinogenesis.^[^ ^13^ ^]^ However, the molecular mechanisms underlying the causal and biological effects of these piRNA‐associated SNPs conferring the risk of cancers, including PCa, remain largely unknown.

In addition, mounting epitranscriptomic evidence has elucidated the crucial role of m^6^A in malignancies.^[^ ^14^ ^]^ In general, the m^6^A modification is enriched around translation stop codons and in the 3′‐untranslated region (UTR) to regulate the mRNA fate.^[^ ^15^ ^]^ The dynamically modified m^6^A sites are recognized and executed by variable readers, such as YTH domain‐containing proteins and heterogeneous nuclear ribonucleoproteins (hnRNPs) family proteins, thereby regulating mRNA splicing, exporting, stability, and translation processes.^[^ ^16^ ^]^ As a crucial m^6^A reader, the YTH domain family protein 2 (YTHDF2) preferentially recognizes m^6^A and recruits RNA‐degrading enzymes or adaptor proteins to trigger rapid degradation of the m^6^A‐containing mRNA, thereby interfering with mRNA translation.^[^ ^17^ ^]^ However, the mechanisms of ncRNA‐participated m^6^A‐containing mRNA stability and translation regulation in PCa remain elusive.

Given the complexity of post‐transcriptional modifications in regulating gene expression, it is necessary to combine these with classical regulatory layers, such as those involving the well‐explored mitogen‐activated protein kinase (MAPK) pathways, to explain the mechanisms of oncogenesis. Dual‐specificity phosphatases (DUSPs) are crucial regulators of cellular signaling pathways, particularly the MAPK pathways.^[^ ^18^ ^]^ These enzymes dephosphorylate both threonine and tyrosine residues on MAPKs, effectively modulating their activity and ensuring precise cellular responses to various stimuli. DUSPs play vital roles in controlling cell proliferation, differentiation, and apoptosis, thus maintaining cellular homeostasis.^[^ ^19^ ^]^ By regulating MAPK signaling, DUSP1 can impact processes essential for metastasis, such as cell migration, invasion, and the epithelial‐mesenchymal transition (EMT). In the context of PCa, DUSP1 is downregulated and is significant due to its ability to influence tumor progression and metastasis.^[^ ^20^, ^21^ ^]^ However, the post‐transcriptional modification and regulation of DUSP1 itself in PCa has not been completely understood.

In this study, combined GWASs enabled us to find the PCa risk‐associated variant rs17201241, residing in PROPER (piRNA overexpressed in prostate cancer), which is aberrantly highly expressed in advanced PCa. We identified an unexpected RNA epigenetic regulatory mechanism by which PROPER is assembled with m^6^A binding protein YTHDF2 and YBX3 to form a piRNA‐induced silencing complex (pi‐RISC), thereby inhibiting translation and promoting degradation of *DUSP1* with the translation initiation factor EIF2S3 in an mRNA‐looping manner. Therefore, this study not only reports a previously unknown function of piRNA in post‐transcriptional regulation but also provides new therapeutic opportunities for targeting the piRNA‐mediated translation machinery in cancer.

## 2 Results

### 2.1 Identification of PCa Risk‐Associated piRNAs by GWAS Meta‐Analyses

**Figure 1:**
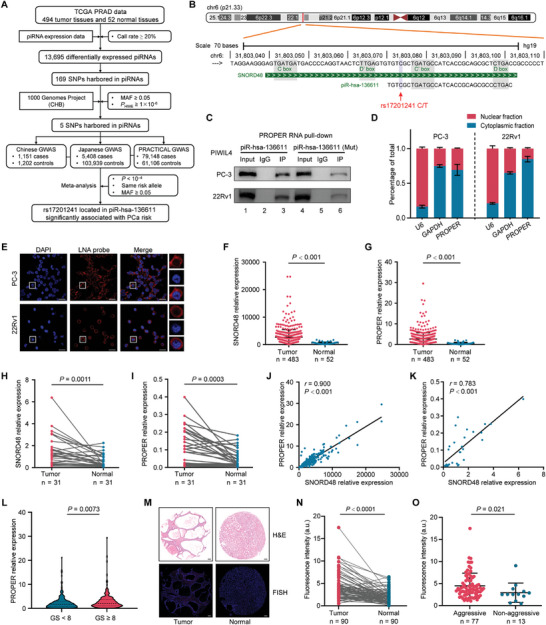
Identification and expression analyses of PCa risk‐associated piRNAs. A) Flowchart illustrating the SNP filtrating design. B) Sequences of rs17201241 (red arrow) resided piR‐hsa‐136611 derived from SNORD48 were analyzed and the conserved motifs were annotated. C) Confirmation of piR‐hsa‐136611 binding to PIWIL4 using biotinylated piR‐hsa‐136611 (Mut) probe in PC‐3 and 22Rv1 cell by biotin‐streptavidin RNA pull‐down assay. D) Distribution of PROPER in nucleus and cytoplasm of PCa cells with U6 or GAPDH as nucleus or cytoplasm markers, respectively. Data are presented as mean ± s.d. (*n* = 4). E) The detection of PROPER in PCa cells were analyzed by fluorescence in situ hybridization using DIG‐labeled locked nucleic acid (LNA) probes to PROPER (red). The nucleus was counterstained with DAPI (blue). Scale bars, 20 µm. F,G) SNORD48 (F) or PROPER (G) expression levels in PCa tissues (*n* = 483) and normal tissues (*n* = 52) from TCGA database. *P* values were calculated using unpaired two‐sided Mann‐Whitney *U*‐tests. H,I) SNORD48 (H) or PROPER (I) expression levels in 31 paired PCa tissues and normal tissues by RT‐qPCR. *P* values were calculated using paired two‐sided Mann‐Whitney *U*‐tests. J) Spearman's rank correlation coefficient analysis between SNORD48 and PROPER expression in tumors from TCGA database. K) Spearman's rank correlation coefficient analysis between SNORD48 and PROPER expression in tumors by RT‐qPCR. L) The association between PROPER expression level and Gleason score (GS) in TCGA. *P* values were calculated using unpaired two‐sided Mann‐Whitney *U*‐tests. M) The representative dot of RNA FISH of PROPER and H&E staining in prostate tissue microarray containing 90 paired PCa and adjacent normal tissues. N) Relative immunofluorescence density [arbitrary units (a.u.)] of RNA FISH between 90 paired tumor and adjacent normal tissues. Scale bars, 100 µm. *P* values were calculated using two‐sided paired Mann‐Whitney *U*‐tests. O) Relative immunofluorescence density [arbitrary units (a.u.)] of RNA FISH between aggressive and non‐aggressive PCa tissues. *P* values were calculated using unpaired two‐sided Mann‐Whitney *U*‐tests.

The flowchart of identifying PCa risk‐associated genetic variants residing in piRNAs is shown in **Figure** **1**A. A total of 13695 differentially expressed piRNAs were observed from The Cancer Genome Atlas (TCGA) cohort. Briefly, a total of 169 SNPs were extracted according to the physical locations of the differentially expressed piRNAs, after standard quality control, only five SNPs being retained for further association analyses (Figure 1A).

We then combined three independent GWAS datasets through a meta‐analysis to detect the risk loci for PCa in Asians and Europeans. The results demonstrated that only rs17201241 C>T was significantly associated with a decreased PCa risk (OR = 0.94, 95% CI = 0.91‐0.97, *P *= 7.33×10^−5^) (Table S1, Supporting Information). We further evaluated the genetic effects of rs17201241 on PCa risk with clinical features in ChinaPCa, for which the characteristics of the subjects are summarized in Table S2 (Supporting Information). There was low heterozygosity among clinical variables of N stage (*I* ^2^ = 34%, *P* = 0.22) and M stage (*I* ^2^ = 60%, *P* = 0.11) in the stratification analyses (Table S3, Supporting Information), indicating that the rs17201241 T allele may play a stronger protective role in the non‐metastatic PCa.

Next, we performed small RNA deep‐sequencing of PCa cells to find the potential PCa risk‐associated piRNA harboring the SNP rs17201241 (Table S4, Supporting Information). Unexpectedly, we found a peak around the size of 31 nts, corresponding to the length of piRNAs (Figure S1A,B, Supporting Information). By mapping clean reads to the piRBase database and published small RNA deep‐sequencing data of germ cells,^[^ ^22^, ^23^ ^]^ we found piR‐hsa‐136611 that harboring rs17201241 was widely detected both in PCa tissues, human oocytes, and early embryos (Figure S1C, Supporting Information). Notably, the sequence alignment analysis showed that piR‐hsa‐136611 was located in the same region with a small nucleolar RNA (snoRNA) named SNORD48 (Figure 1B). This piRNA retained the conserved characteristic sequences of snoRNA C’/D boxes (Figure 1B), following piRNA characteristics reported in previous studies.^[^ ^24^, ^25^ ^]^

The abundance of piR‐hsa‐136611 was relatively high in PC‐3 and 22Rv1 cells (Figure S1D, Supporting Information). To confirm the existence of piR‐hsa‐136611, we isolated small RNAs from PCa cell lines and performed a northern blot assay, which confirmed the existence of piR‐hsa‐136611 (Figure S1E, Supporting Information). We also detected the expression of PIWI proteins by western blotting using specific antibodies (Figure S1F, Supporting Information). Next, the RNA immunoprecipitation (RIP) and RNA pull‐down assays confirmed the specific binding of piR‐hsa‐136611 to the PIWIL4 protein (Figure 1C; Figure S1G, Supporting Information). Consistent with this, piR‐hsa‐136611 expression significantly decreased when knocking down PIWIL4 (Figure S1H,I, Supporting Information). Furthermore, to examine the 2′*‐O*‐methylation modification status of 3′ terminus of piR‐hsa‐136611, a typical feature of piRNA, we conducted sodium‐periodate‐mediated oxidation experiments.^[^ ^26^ ^]^ The results indicated that piR‐hsa‐136611 and positive control piR‐hsa‐1681 exhibited resistance to periodate treatment, indicating their methylation (Figure S1J, Supporting Information). In contrast, the negative control miRNA miR‐21 were oxidized after treatment and therefore degraded (Figure S1J, Supporting Information). Together, these results suggest the real existence of piR‐hsa‐136611. As piR‐hsa‐136611 has not been documented in the Genebank, we named it PROPER, which stands for a piRNA overexpressed in prostate cancer, considering the follow‐up analysis.

### 2.2 PROPER is Up‐Regulated and Promotes Malignant Phenotypes of PCa

Subcellular localization analysis revealed that PROPER is mainly distributed in the cytoplasm (Figure 1D,E). Importantly, both PROPER and its precursor SNORD48 exhibited higher expression in PCa tissues compared to normal tissues in the TCGA cohort (Figure 1F,G), which was consistent with our independent in‐house validation cohort (Figure 1H,I). Additionally, PROPER expression showed a significant positive correlation with SNORD48 in both the TCGA and our patient cohorts (Figure 1J,K). Northern blot assays also confirmed the elevated expression of PROPER in PCa (Figure S1K, Supporting Information). Moreover, PROPER was also found to be highly expressed in various other tumors (Figure S1L, Supporting Information). It is noteworthy that, increased PROPER expression was associated with higher Gleason scores and tumor progression (Figure 1L; Figure S1M, Supporting Information). To further establish the clinical relevance of PROPER expression, we performed RNA FISH using DIG‐labeled locked nucleic acid (LNA) probes targeting PROPER on a tissue microarray containing 90 paired PCa and normal tissues (Table S5, Supporting Information). The results confirmed higher expression levels of PROPER in PCa tissues compared to benign prostate samples (Figure 1M,N), particularly in aggressive PCa cases (Figure 1O).

**Figure 2:**
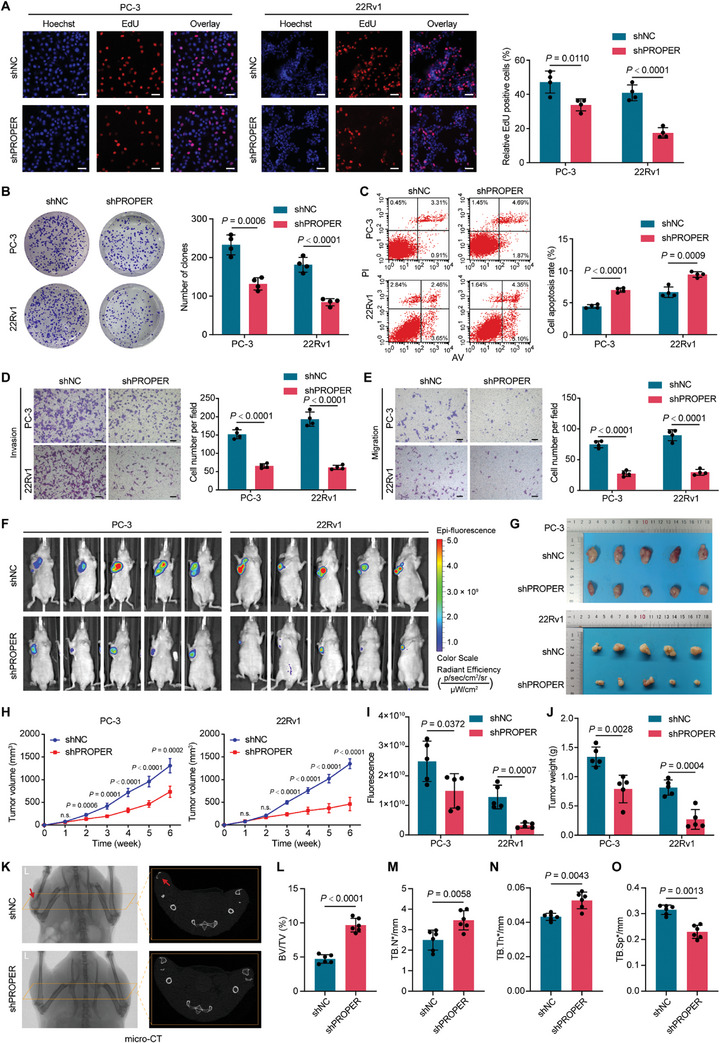
PROPER functions as an oncogene to promote malignant phenotypes and metastasis of PCa in vitro and in vivo. A) Effects of PROPER expression on the proliferation of PC‐3 and 22Rv1 cells tested by EdU assay. Nuclei labeled with hoechst are in blue. The fluorescent thymidine analog EdU was used to identify proliferative cells by labeling their DNA in red. Representative images (left, middle) and quantitative statistics by flow cytometry (right). Data are presented as mean ± s.d. (*n* = 4); *P* values were calculated using unpaired two‐sided Mann‐Whitney *U*‐tests. B) Effects of PROPER expression on the colony formation capability of PCa cells. Representative images (left) and quantification (right) of colony formation results. Data are presented as mean ± s.d. (*n* = 4); *P* values were calculated using unpaired two‐sided Mann‐Whitney *U*‐tests. C) Effects of PROPER expression on PCa cell apoptosis. Data are presented as mean ± s.d. (*n* = 4); *P* values were calculated using unpaired two‐sided Mann‐Whitney *U*‐tests. D,E) Effects of PROPER on the capability of invasion (D) and migration (E) of PCa cells in vitro. Representative images of transwell assays (left panels) and quantitative statistics (right panels). Scale bars, 100 µm. Data are presented as mean ± s.d. (*n* = 4); *P* values were calculated using unpaired two‐sided Mann‐Whitney *U*‐tests. F, G) Representative luminescence images of nude mice (F) and xenograft tumors (G) (*n* = 5). H) The growth curves of xenograft tumors. *P* values were calculated using unpaired two‐tailed Student's *t*‐tests. I,J) Quantitative statistics of luminescence imaging and tumor weight in nude mice. Data are presented as mean ± s.d. (*n* = 5); *P* values were calculated using unpaired two‐tailed Student's *t*‐tests. K) Represents the micro‐CT images of the bone metastasis from sacrifice mice. Tibiae are shown in left and the section of the micro‐CT image are shown in right (*n* = 6). L–O) Quantitative analyses of bone volume/total volume (BV/TV, %) (L), trabecular number (Tb.N*) M), trabecular thickness (Tb.Th*) (N), and trabecular separation (Tb.Sp*) (O). Data are presented as mean ± s.d. (*n*  =  6); *P* values were calculated using unpaired two‐tailed Student's *t*‐tests.

To assess the effects of PROPER on the malignant phenotype of PCa cells, we conducted a series of loss‐of‐function assays. The efficiency of shRNA‐mediated PROPER knockdown was shown in Figure S2A (Supporting Information). PROPER knockdown substantially inhibited the proliferation and the colony formation ability of PCa cells (**Figure** **2**A,B). Flow cytometry analysis revealed a significant increase in apoptosis in PROPER knockdown cells (Figure 2C). Furthermore, knockdown of PROPER significantly suppressed the migration and invasion properties of PCa cells (Figure 2D,E). We also evaluated the impact of PROPER on tumor growth in vivo by constructing a xenograft mouse model (Figure 2F). We observed a marked reduced in tumor growth rate, fluorescence intensity, and tumor weight in mice transplanted with shRNA‐mediated PROPER knockdown cells after 6 weeks (Figure 2G–J). Subsequent H&E and IHC staining demonstrated that PROPER knockdown significantly decreased the Ki‐67 signal (Figure S2B, Supporting Information). Moreover, a PCa bone metastasis model examined by micro‐computed tomography (micro‐CT) showed less bone destruction in PROPER knockdown mice compared to the control group (Figure 2K). The analysis also revealed a decreasing trend in tibial bone mineral density associated with high PROPER expression in tumors, with gradual thinning and loosening of trabecular bone micro‐architecture (Figure 2L–O; Figure S2C, Supporting Information). Collectively, these results suggest that PROPER plays an oncogenic role in PCa and indicate an obvious link between high PROPER expression and aggressive prostate tumor phenotype, potentially impacting the clinical progression of PCa.

### 2.3 The rs17201241 T Allele Affects SNORD48 Mature and PROPER Expression

We proceeded to investigate whether there is an association between variation at rs17201241 and PROPER expression. An expression quantitative trait loci (eQTL) analysis in a collection of 90 human prostate tumors demonstrated that the protective rs17201241 T allele was significantly associated with decreased PROPER levels (Figure S3A, Supporting Information). Moreover, the sequences of PROPER could be precisely aligned within the sequences of SNORD48, and the cleavage sites required for PROPER production were not random.^[^ ^24^, ^27^ ^]^ Considering that Fibrillarin (FBL) or NOP56 are known to play critical roles in snoRNA maturation, stability, and small nucleolar ribonucleoproteins (snoRNPs) complex assembly (Figure S3B, Supporting Information),^[^ ^28^ ^]^ we performed siRNA‐mediated knockdown for *FBL* and *NOP56*. As expected, this led to reduced expression of both SNORD48 and PROPER (Figure S3C–F, Supporting Information). In addition, ectopic expression of SNORD48 significantly increased PROPER expression (Figure S3G, Supporting Information). These results further confirm that SNORD48 serves as the precursor of PROPER.

Since rs17201241 is located within the kink‐turn (k‐turn) structure, which is known to be essential for snoRNP assembly,^[^ ^29^ ^]^ we hypothesized that rs17201241 could affect the binding affinity between SNORD48 and NOP56, thereby influencing SNORD48 maturation. To verify this, we modeled the 3D structure of NOP56 and SNORD48 carrying different alleles of rs17201241 (Figure S3H,I, Supporting Information). Subsequently, we conducted an *in‐slico* RNA‐protein docking analysis of SNORD48 and NOP56. The results of the molecular docking simulation supported the perfect docking of SNORD48 with NOP56, with a ZRank score of −38.773 (Figure S3J, Supporting Information). Conversely, rs17201241 resulted in a weaker interaction between SNORD48 and NOP56 with a ZRank score of −28.312 (Figure S3K, Supporting Information). Furthermore, when transfected SNORD48 plasmid harboring different alleles of rs17201241 in PC‐3 cells after knocking down of endogenous SNORD48, we found the PROPER expression significantly increased in the C allele than in the T allele group (Figure S3L, Supporting Information). These results suggest that the rs17201241 T allele may impact SNORD48 maturation and subsequently decrease the expression of SNORD48‐derived PROPER.

### 2.4 PROPER Affects YTHDF2‐Mediated mRNA Translation

We aimed to investigate the molecular mechanisms underlying the role of PROPER in PCa. Since ncRNAs can repress target genes located at a nearby genomic locus via base‐pairing rules,^[^ ^30^ ^]^ we initially examined the mRNA level of nine genes within a 0.5 m‐base region centered around PROPER (Figure S4A, Supporting Information). However, knockdown of PROPER did not alter the expression of these genes (Figure S4B, Supporting Information), implying that PROPER does not function by regulating the expression of neighboring genes.

**Figure 3:**
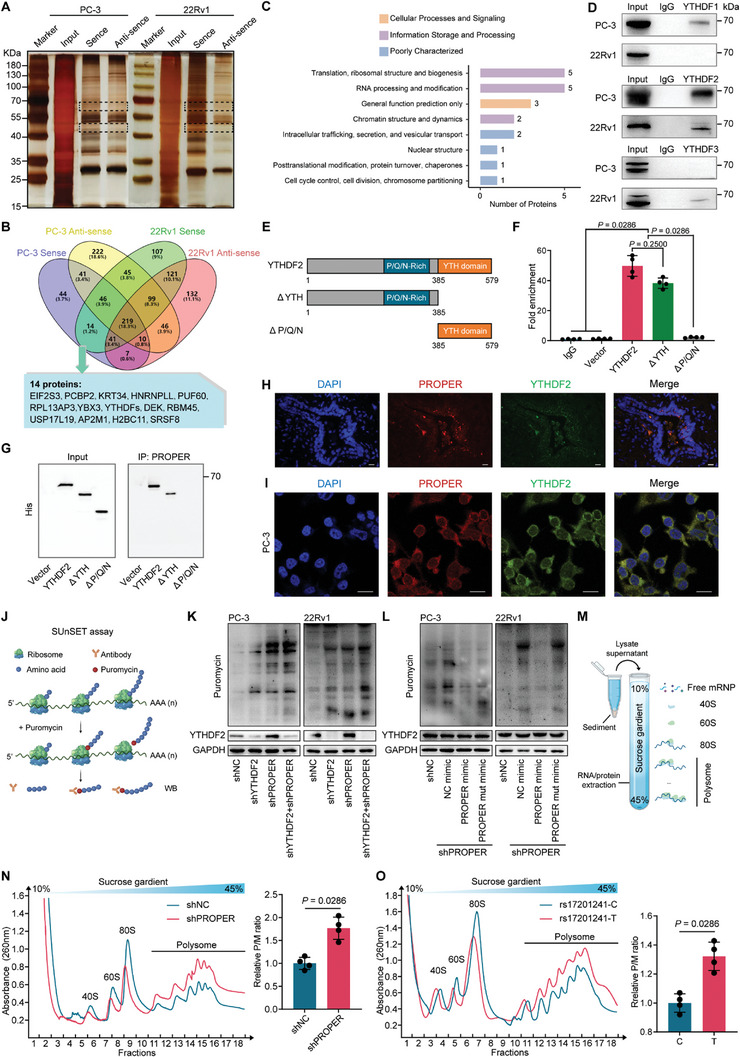
PROPER interacts with YTHDF2 to regulate mRNA translation. A) Silver staining identification of the PROPER‐protein complex pulled down by PROPER sense probe or anti‐sense probe with protein extracts from PC‐3 and 22Rv1 cells using a biotin‐based pull‐down assay. B) Venn diagram showed potential interacting RNA‐binding proteins (RBPs) of PROPER by mass spectrometry and bioinformatic analysis. C) KEGG pathway enrichment analysis of PROPER conjunct 14 proteins. D) Immunoblots of YTHDF1/2/3 enriched by PROPER probes in PC‐3 and 22Rv1 cells. IgG were set as negative controls. E) Diagrams of full‐length and domain‑truncated fragments of YTHDF2; the P/Q/N‐rich domain and YTH domain are indicated. F) RIP‐qPCR analysis of PROPER immunoprecipitated using an anti‐FLAG antibody in PC‐3 cells expressing the indicated YTHDF2 plasmids. The data were normalized to the input levels. Data are presented as mean ± s.d. (*n* = 4); *P* values were calculated using unpaired two‐tailed Student's *t*‐tests. G) RNA pull‐down of PROPER probe with FLAG‐tagged YTHDF2 domain‐deletion mutants. H,I) YTHDF2 immunofluorescence (green) and FISH analysis of PROPER (red) in PCa tissues (H) (Scale bars, 20 µm) and PC‐3 cells (I) (Scale bars, 10 µm). The nucleus was counterstained with DAPI (blue). J) Schematic of SUnSET assay that based on puromycin incorporation to monitor protein synthesis. K,L) SUnSET analyses of PC‐3 and 22Rv1 cells showing the changes in protein synthesis among the indicated samples. M) Schematic of polysome profiling that is based on sucrose‐gradient separation of translated mRNAs, which are associated with polysomes, from untranslated ones. N,O) Polysome profiles and polysome‐to‐monosome (P/M) ratio changes upon PROPER knockdown (N) and PROPER carries different alleles (O). The x axis indicates the free ribosome subunit (40S/60S), monosome (80S), and polysome fractions separated by 10%−45% sucrose gradients. The y axis indicates the absorbance at 260 nm. Data are presented as mean ± s.d. (*n* = 4); *P* values were calculated using unpaired two‐sided Mann‐Whitney *U*‐tests.

Another potential function we speculated was that PROPER interacts with proteins to form RNA‐protein complexes. Therefore, we performed biotin‐streptavidin RNA pull‐down assays using biotinylated PROPER, and several protein bands specifically enriched in PROPER were subjected to mass spectrometry (**Figure** **3**A). A total of 14 potential PROPER‐interacting proteins were reproducibly identified based on unique peptides (Figure 3B; and Table S6, Supporting Information). Notably, the most enriched pathways among these 14 proteins were involved in the “Translation, ribosomal structure and biogenesis pathway” and “RNA processing and modification pathway” (Figure 3C). Intriguingly, YTHDFs, which are well‐characterized m^6^A readers known to regulate RNA stability and translation, were found to be enriched by PROPER (Figure S4C, Supporting Information). Among the YTHDFs proteins, western blotting indicated that YTHDF2 was pulled down to a greater extent compared to YTHDF1 or YTHDF3 (Figure 3D), which was further validated by RIP‐qPCR (Figure S4D, Supporting Information). An RNA pull‐down assay using purified His‐tagged recombinant YTHDF2 proteins was performed to verify the direct interaction between PROPER and YTHDF2 (Figure S4E, Supporting Information). To identify the functional regions of YTHDF2 responsible for its binding to PROPER, we generated a series of constructs expressing truncated forms of YTHDF2 (Figure 3E). It demonstrated that the Pro/Gln/Asn(P/Q/N)‐rich domain, but not the YTH domain, was critical for the RNA‐binding activity of YTHDF2 to PROPER (Figure 3F,G). RT‐qPCR of PROPER after m^6^A methylated RIP (MeRIP) and YTHDF2 knockdown indicated that piRNA PROPER does not carry m^6^A modifications (Figure S4F,G, Supporting Information). Clinical correlation analyses of TCGA data showed that YTHDF2 was highly expressed in PCa tissues and positively associated with the tumor stage and prognosis (Figure S4H–K, Supporting Information). FISH and IF assays showed colocalization of PROPER with YTHDF2 in PCa tissues (Figure 3H). Moreover, PROPER and YTHDF2 were found to be colocalized in the cytoplasm at the cellular level (Figure 3I), which was consistent with our subcellular localization analysis (Figure 1D,E).

YTHDF2 functions by inducing degradation and altering the translation efficiency of target mRNAs in the cytoplasm through recognition of m^6^A modification sites.^[^ ^17^ ^]^ Therefore, we performed a surface sensing of translation (SUnSET) assay to detect puromycin‐labeled polypeptides, which serves as a measure of global protein synthesis (Figure 3J). The results showed that knockdown of YTHDF2 or PROPER led to increased protein synthesis, and this effect was significantly enhanced when the complex was simultaneously knock down (Figure 3K). Consistently, overexpression of PROPER, but not PROPERmut, revised this effect (Figure 3L). In line with these findings, depletion of PROPER resulted in a significant change in the polysome profile and an increased polysome‐to‐monosome (P/M) ratio (*P* = 0.0286) (Figure 3M,N), indicating that PROPER suppressed protein translation by regulating the initiation step (Figure S4L, Supporting Information). Subsequently, we generated PC‐3 cells with different alleles of rs17201241 using CRISPER/Cas9 (Figure S4M, Supporting Information), and PROPER containing the T allele exhibited a higher P/M ratio than that containing the C allele (*P* = 0.0286) (Figure 3O). Taken together, these results suggest that the PROPER/YTHDF2 complex may regulate the stability and translation of specific target RNAs at the post‐transcriptional level.

### 2.5 Analysis of the m^6^A Methylome and PROPER/YTHDF2 Downstream Targets

**Figure 4:**
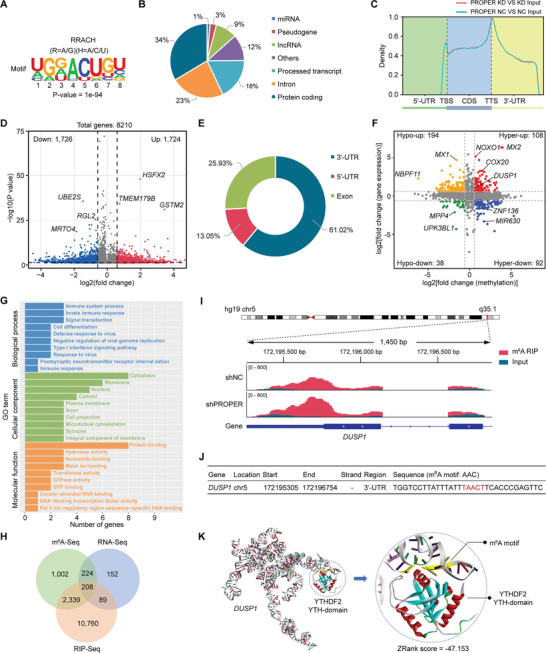
*DUSP1* is the target of the PROPER/YTHDF2 complex in PCa cells. A) The m^6^A motif detected by the MEME motif analysis with m^6^A‐Seq data. B) Percentages of various RNA species with different m^6^A modifications. C) Metagene profiles of m^6^A enrichment across mRNA transcriptome composed of 5′‐UTR, CDS, and 3′‐UTR, in PC‐3 cells treated with PEOPER knockdown and control. D) Volcano plot showing the m^6^A enrichment in mRNAs of PEOPER knockdown and control groups in PC‐3 cells. m^6^A‐containing mRNAs with 1724 increased (up) and 1726 decreased peak (down) enrichment are highlighted in red and green, respectively. Distribution of m^6^A peaks and YTHDF2‐binding peaks across transcripts. E) Percentages of m^6^A peaks in different transcript segments in PROPER knockdown and control groups in PC‐3 cells. F) Correlation between the level of gene expression (overall transcript) and changes in m^6^A levels in PROPER knockdown and control groups in PC‐3 cells. The upregulated peaks were termed as the hypermethylated m^6^A peaks and the downregulated peaks were termed as the hypomethylated m^6^A peaks. G) Gene ontology (GO) enrichment analysis of differentially expressed genes. H) Venn diagram illustrated 208 overlapping peaks of PROPER/YTHDF2 complex targets identified by m^6^A‐Seq, RNA‐Seq, and RIP‐Seq (data from GSE49339) analysis. I) MeRIP‐Seq displays the distribution of m^6^A peaks and YTHDF2‐binding peaks in *DUSP1* mRNA of PROPER knockdown and control groups in PC‐3 cells. J) The m^6^A‐containing YTHDF2 binding motif at the 3′‐UTR of *DUSP1* mRNA (red, m^6^A motif). K) *In‐slico* RNA‐protein docking analysis displays the crystal structure of YTH domain docking with the conserved A(m^6^A)C of *DUSP1*.

The screening flowchart for the target genes of PROPER/YTHDF2 complex is depicted in Figure S5 (Supporting Information). Initially, we performed MeRIP‐Seq in PC‐3 cells with stable knockdown of PROPER or control using shRNA. The analysis of m^6^A peaks through the MEME algorithm revealed a consensus m^6^A motif RRACH, confirming successful immunoprecipitation (**Figure** **4**A). Further analysis showed that observed m^6^A modifications were mainly present in protein‐coding mRNAs (Figure 4B; Figure S6A, Supporting Information). Consist with previous reports,^[^ ^31^ ^]^ m^6^A peaks were predominantly distributed in 3′‐UTRs, coding sequences (CDSs), and near stop codons (Figure 4C). A comparison of m^6^A abundance between PROPER knockdown and control cells identified 1724 upregulated m^6^A peaks and 1726 downregulated m^6^A peaks (Figure 4D; Figure S6B and Table S7, Supporting Information). These differential peaks were predominantly located in 3′‐UTRs (Figure 4E). However, there was no significant difference in global m^6^A levels between PROPER knockdown and control cells, as detected by dot blot assays (Figure S6C, Supporting Information).

Next, we analyzed the correlation between m^6^A modification and corresponding gene expression. The m^6^A peak data was plotted against the RNA‐Seq data (MeRIP‐Seq input library) of gene expression (Figure 4F). We identified a total of 432 genes with differential m^6^A‐modification and corresponding expression change (Table S8, Supporting Information). These genes were primarily associated with “Immune system process”, “Cytoplasm”, and “Protein binding” based on gene ontology (GO) analysis (Figure 4G). Combining this with the YTHDF2 RIP‐Seq data,^[^ ^32^ ^]^ we identified 208 common peaks corresponding to 175 genes (Figure 4H), of which 138 genes were upregulated and 37 genes were downregulated (Figure S6D, Supporting Information). We next used photoactivatable ribonucleoside‐enhanced crosslinking and immunoprecipitation sequencing (PAR‐CLIP‐Seq) data to explore any direct interactions within the above peaks between YTHDF2 and its targeted transcripts with adenosine methylation (Figure 4; and Table S9, Supporting Information). To further narrow down the target genes that may be regulated by the PROPER/YTHDF2, we knocked down PROPER or YTHDF2 and found that only the expression of *DUSP1* showed a significant increase that consistent with the MeRIP‐Seq results and the YTHDF2 RIP‐Seq data (Table S9, Supporting Information). We observed the presence of m^6^A‐containing YTH binding motif in the 3′‐UTR of *DUSP1*, which was within a hypermethylated m^6^A peak upon PROPER knockdown (Figure 4I,J). Alignment with CLIP‐Seq data from multiple other cells constantly indicated the abundance of methylated adenosine bases in the 3′‐UTR of *DUSP1* mRNA (Figure S6E, Supporting Information). Furthermore, In‐slico RNA‐protein docking analysis confirmed the docking capability of the crystal structure of YTH‐YTHDF2 with the conserved A(m^6^A)C of *DUSP1* (Figure 4K). These results strongly indicate that the m^6^A‐modified *DUSP1* is an intriguing and potential regulatory target gene of the PROPER/YTHDF2 complex.

### 2.6 The PROPER/YTHDF2 Complex Mediates *DUSP1* Downregulation in an m^6^A‐Dependent Manner

**Figure 5:**
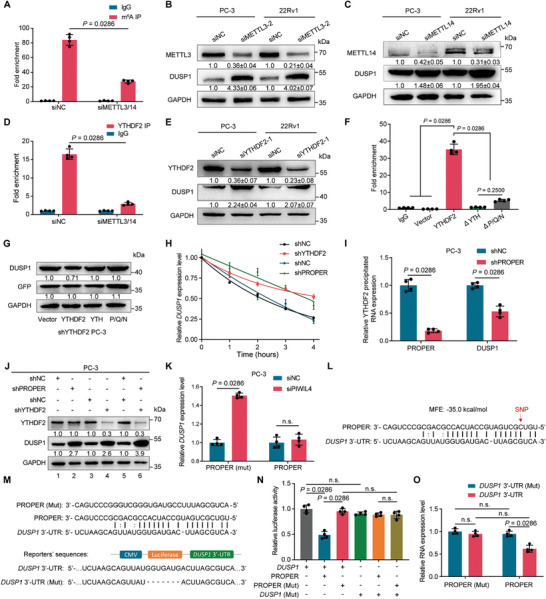
The PROPER/YTHDF2 complex mediates *DUSP1* downregulation. A) Gene‐specific MeRIP‐qPCR validation of the m^6^A levels at *DUSP1* in PC‐3 cells. Data are presented as mean ± s.d. (*n* = 4). The data were normalized to the input levels. *P* values were calculated using unpaired two‐sided Mann‐Whitney *U*‐tests. B,C) Knockdown of METTL3 and METTL14 on the expression of DUSP1. Quantification of blotting intensity for indicated proteins is shown below protein bands (the one in NC is set as 1.0 after normalization with GAPDH blotting). D) METTL3/14 knockdown impaired the interaction between YTHDF2 and *DUSP1* by RIP‐qPCR. The data were normalized to the input levels. *P* values were calculated using unpaired two‐sided Mann‐Whitney *U*‐tests. E) The effects of YTHDF2 knockdown on the expression of DUSP1. Quantification of blotting intensity for indicated proteins is shown below protein bands (the one in NC is set as 1.0 after normalization with GAPDH blotting). F) RIP‐qPCR analysis of *DUSP1* immunoprecipitated using an anti‐FLAG antibody in PC‐3 cells expressing the indicated YTHDF2 plasmids. The data were normalized to the input levels. Data are presented as mean ± s.d. (*n* = 4). *P* values were calculated using unpaired two‐sided Mann‐Whitney *U*‐tests. G) DUSP1 protein levels in cells co‐expressing the YTHDF2 shRNA and the YTHDF2‐truncated plasmids. Quantification of blotting intensity for indicated proteins is shown below protein bands (the one in Vector is set as 1.0 after normalization with GAPDH blotting). H) RNA decay assays indicate the effects of PROPER and YTHDF2 knockdown on the stability of *DUSP1* RNA. I) RIP‐qPCR analysis of *DUSP1* and PROPER immunoprecipitated by YTHDF2. Data are presented as mean ± s.d. (*n* = 4). *P* values were calculated using unpaired two‐sided Mann‐Whitney *U*‐tests. J) Immunoblotting of DUSP1 protein expression upon knockdown of PROPER and/or YTHDF2. Quantification of blotting intensity for indicated proteins is shown below protein bands (the one in line 1 is set as 1.0 after normalization with GAPDH blotting). K) The effects of PIWIL4 knockdown coupled with PROPER (Mut) transfection on the expression of *DUSP1* in PC‐3 cells. Data are presented as mean ± s.d. (*n* = 4). n.s., not significant; *P* values were calculated using unpaired two‐sided Mann‐Whitney *U*‐tests. L) Targeting sites prediction of PROPER within the 3′‐UTRs of *DUSP1* using the RNAhybrid. M) Predicted PEOPER regulatory elements at the 3′‐UTRs of *DUSP1* and the synthetic PROPER, mutated PROPER, and wild‐type and mutated 3′‐UTR Renilla luciferase reporters. N) Reporter gene assays evaluate the functional consequence of the predicted piRNA:mRNA interactions. The reporters were co‐transfected along with cognate chemically synthesized piRNAs (containing phosphate at the 5′ terminus and 2′‐O‐methylation at the 3′ terminus) or mutant versions. Data are presented as mean ± s.d. (*n* = 4). n.s., not significant; *P* values were calculated using unpaired two‐sided Mann‐Whitney *U*‐tests. O) RT‐qPCR analysis of reporter mRNAs. Data are presented as mean ± s.d. (*n* = 4); n.s., not significant; *P* values were calculated using unpaired two‐sided Mann‐Whitney *U*‐tests.

The m^6^A modification is mainly catalyzed by the heterodimer of methyltransferase‐like 3 (METTL3) and methyltransferase‐like 14 (METTL14).^[^ ^33^ ^]^ We observed a significant decrease in overall RNA m^6^A levels upon knockdown of METTL3/METTL14, as detected by dot blot assays (Figure S7A‐D, Supporting Information). Using MeRIP‐qPCR assay, we found that the enrichment of m^6^A in *DUSP1* was drastically reduced upon METTL3/METTL14 knockdown (*P* = 0.0286) (Figure 5A), which led to increased expression levels of DUSP1 (Figure 5B,C; Figure S7E,F, Supporting Information). Moreover, the interaction between YTHDF2 and *DUSP1* was also remarkably impaired upon METTL3/14 knockdown (Figure 5D). Consistently, knockdown of YTHDF2 also resulted in a substantial increase in *DUSP1* level (Figure 5E; Figure S7G, Supporting Information). Next, we expressed truncated versions of YTHDF2 in PCa cells and found that deleting either the YTH domain or the P/Q/N abolished the binding ability of YTHDF2 to *DUSP1* (Figure 5F). Additionally, DUSP1 protein levels were significantly downregulated in cells co‐expressing the YTHDF2 shRNA and the full‐length YTHDF2 overexpression plasmids (Figure 5G). These results suggest that the interaction between the PROPER/YTHDF2 complex and *DUSP1* is regulated via an m^6^A‐dependent manner.

Considering that MeRIP‐Seq assays demonstrated increased m^6^A modification and expression of *DUSP1* after PROPER knockdown, we hypothesized that the highly expressed PROPER in PCa might synergize with YTHDF2 and facilitate its recognition of m^6^A‐modified *DUSP1*, leading to its degradation. We performed RNA decay assays and found that knockdown of PROPER or YTHDF2 reduced the stability of *DUSP1* (Figure 5H). Moreover, knockdown of PROPER followed by YTHDF2/PROPER complex isolation in a RIP assay using YTHDF2 antibody revealed a significant attenuation of the interaction between YTHDF2 and *DUSP1* (Figure 5I; Figure S7H, Supporting Information), resulting in increased *DUSP1* expression levels (Figure S7I,J, Supporting Information). This effect was further enhanced when PROPER and YTHDF2 were simultaneously knocked down (Figure 5J). In contrast, ectopic expression of PROPER profoundly reduced endogenous *DUSP1* levels (Figure S7K, Supporting Information). Since we had previously demonstrated the binding of PROPER with PIWIL4 protein (Figure 1C; Figure S1G, Supporting Information) and the effects of PIWIL4 knockdown in suppressing PROPER expression (Figure S1H,I, Supporting Information), we found that PIWIL4 knockdown coupled with PROPERmut transfection marginally affected the *DUSP1* level, likely due to the absence of endogenous PROPER caused by PIWIL4 knockdown (Figure 5K).

Next, we investigated the mechanism by which PROPER guides YTHDF2 to recognize m^6^A modification at the 3′‐UTR of *DUSP1*, thereby leading to *DUSP1* downregulation. A previous study reported the involvement of piRNAs in instructing massive mRNA elimination in spermatids, similar to the action of miRNA sponges in somatic cells.^[^ ^34^ ^]^ Therefore, we speculated that the function of PROPER might be related to sequences in the 3′‐UTR of *DUSP1*. Using the RNAhybrid, a tool for finding the minimum free energy (MFE) hybridization of a long and a short RNA,^[^ ^35^ ^]^ we identified potential targeting sites of PROPER within the 3′‐UTR of *DUSP1* with an MFE of −35.0 kcal mol^−1^ (Figure 5L). We performed a reporter gene assay to evaluate the functional consequence of the predicted piRNA:mRNA interactions (Figure 5M). Co‐transfection of the reporters with cognate chemically synthesized piRNAs resulted in significant repression of DUSP1 reporters but not the mutant versions by co‐transfected PROPER (Figure 5N). RT‐qPCR analysis of the reporter mRNAs confirmed piRNA‐targeting dependent responses, indicating that piRNAs may induce mRNA degradation (Figure 5O). Taken together, these results suggest that PROPER not only couples with YTHDF2 to promote m^6^A‐dependent deadenylation and decay of *DUSP1* but also triggers these effects through imperfect base‐pairing with specific sites in the 3′‐UTRs of *DUSP1*.

### 2.7 YBX3 and EIF2S3 are Critical Partners of the PROPER/YTHDF2‐Ediated DUSP1 Decay

Among the identified interacting proteins of PROPER, we also found YBX3 and EIF2S3 that post‐transcriptionally controls mRNA stability or translation (Figure 3B; Figure S8A,B, and Table S6, Supporting Information). The RNA pull‐down assay confirmed the interaction of YBX3 and EIF2S3 with PROPER (Figure S8C, Supporting Information). In addition, immunofluorescence analysis in PCa tissues showed colocalization of PROPER with YBX3 and EIF2S3 (Figure S8D, Supporting Information).

**Figure 6:**
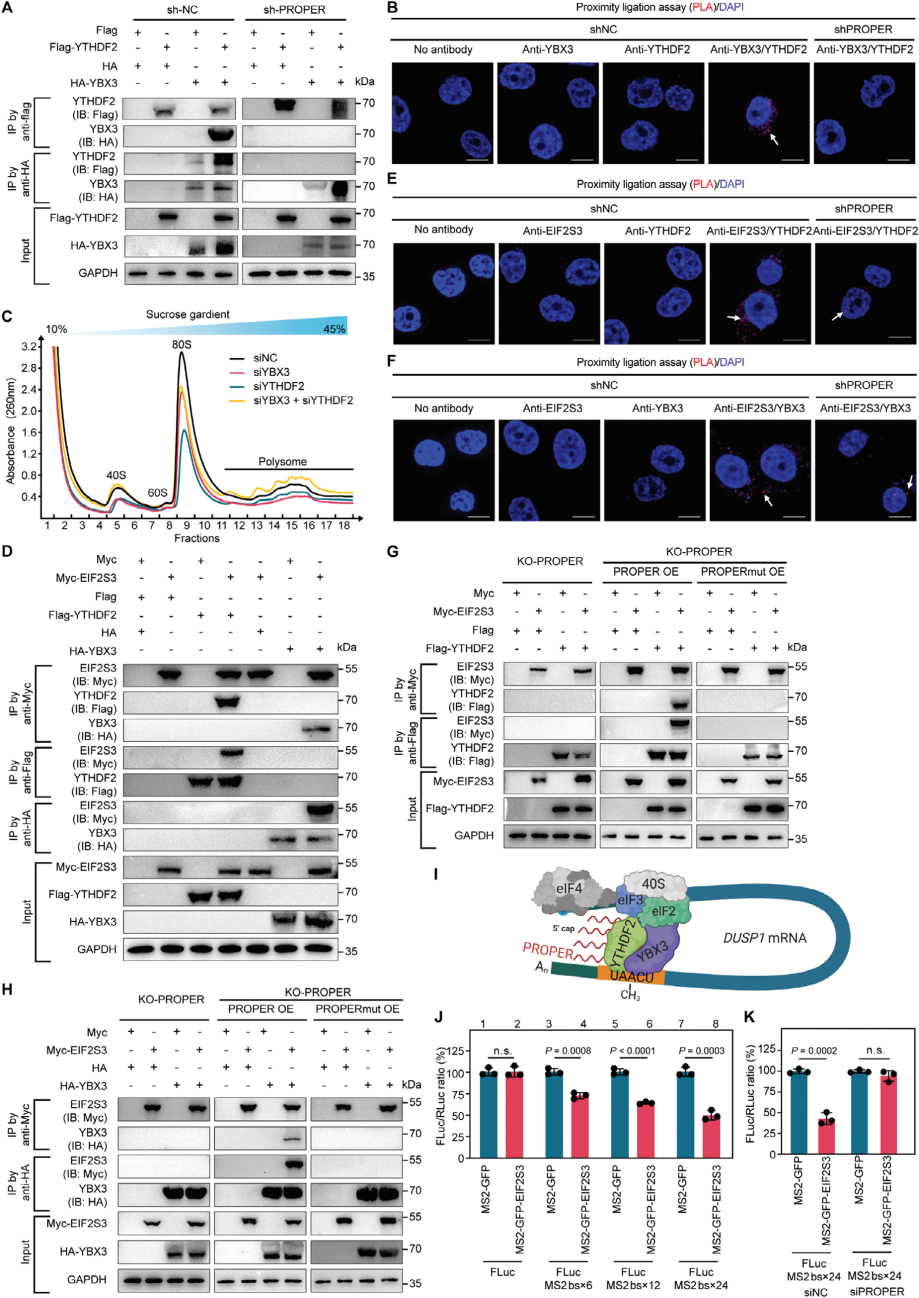
YBX3 and EIF2S3 are required for the PROPER/YTHDF2 complex mediated *DUSP1* circularization and translational suppression. A) Lysates prepared from PC‐3 and 22Rv1 cells were hybridized with biotinylated PROPER probe and subject to RNA pull‐down assays. The remaining lysates were subject to western blotting with antibodies against YBX3 and EIF2S3. B) Proximity ligation assay (PLA) demonstrate the interaction between YBX3 and YTHDF2 in PC‐3 cells; (*n* = 4). C) Polysome profiles and polysome‐to‐monosome (P/M) ratio changes upon YBX3 knockdown, YTHDF2 knockdown, YBX3 and YTHDF2 knockdown together, and control PC‐3 cells; (*n* = 4). D) Co‐IP assays of the interaction between Myc‐EIF2S3 and Flag‐YTHDF2 or HA‐YBX3 in PC‐3 cells. E) PLA demonstrate the interaction between EIF2S3 and YTHDF2 in PC‐3 cells; (*n* = 4). F) PLA demonstrate the interaction between EIF2S3 and YBX3 in PC‐3 cells; (*n* = 4). G) Myc‐EIF2S3 Co‐IP with Flag‐YTHDF2 in PROPER knockout, and PROPER knockout with ectopically expressed PROPER/PROPERmut of PC‐3 cells. H) Myc‐EIF2S3 Co‐IP with HA‐YBX3 in PROPER knockout, and PROPER knockout with ectopically expressed PROPER/PROPERmut of PC‐3 cells. I) Schematic diagram illustrating the mechanism that PROPER functions as a rivet to enhance EIF2S3‐YTHDF2/YBX3 interaction. J) EIF2S3 tethered at the 3′‐UTR of mRNA augments translation of an upstream reporter gene by MS2 tethering assay. Data are presented as mean ± s.d. (*n* = 3); n.s., not significant; *P* values were calculated using unpaired two‐tailed Student's *t*‐tests. K) EIF2S3 mediated *DUSP1* mRNA circularization and translation was inhibited by PORPER. Data are presented as mean ± s.d. (*n* = 3); n.s., not significant; *P* values were calculated using unpaired two‐tailed Student's *t*‐tests.

A recent report established YBX3 as a translation repressor of m^6^A‐modified transcripts.^[^ ^36^ ^]^ A previous study has reported that YBX3 primarily binds to mRNAs toward their 3′‐UTR.^[^ ^37^ ^]^ Available eCLIP‐Seq data of YBX3‐RNA complexes also showed the distribution of multiple YBX3‐binding peaks across the 3′‐UTR of *DUSP1* (Figure S8E, Supporting Information). Notably, co‐Immunoprecipitation (Co‐IP) assays indicated that YBX3 could interact with YTHDF2 (**Figure** **6**A; Figure S8F, Supporting Information), which was further confirmed by an in situ proximity ligation assay (PLA), demonstrating a specific protein interaction between YBX3 and YTHDF2 in the cell cytoplasm in a PROPER‐dependent manner (Figure 6B; Figure S8G, Supporting Information). Therefore, we hypothesized that YBX3 might serve as a cofactor of YTHDF2 in recognizing the m^6^A modification of *DUSP1* and regulating translation efficiency. To confirm this, we conducted RIP assays with the YBX3 antibody and revealed that the recognition of YBX3 to *DUSP1* m^6^A sites was dependent on PROPER (Figure S8H,I, Supporting Information). Notably, polysome profiling followed by RT‐qPCR further demonstrated that more *DUSP1* was present in the polysome‐bound fractions in YBX3 and YTHDF2‐depleted cells compared to YTHDF2 or YBX3‐depleted cells and the controls (Figure 6C; Figure S8J,K, Supporting Information). These results suggest that YBX3 acted as a cofactor of YTHDF2 in suppressing translation efficiency.

EIF2S3, a subunit of the eukaryotic initiation factor 2 (eIF2), is critical in eukaryotic translation initiation involved in the rate‐limiting step in protein synthesis and translation deregulation, which is known to be important in cancer.^[^ ^38^ ^]^ Our Co‐IP assay confirmed the protein interaction between EIF2S3 and YTHDF2/YBX3 (Figure 6D; Figure S8L,M, Supporting Information). Furthermore, the PLA assay revealed the proximity of EIF2S3‐YTHDF2 and EIF2S3‐YBX3 in living cells, and their interactions were markedly weakened after PROPER knockdown (Figure 6E,F; Figure S8N,O, Supporting Information).

### 2.8 PROPER Promotes the EIF2S3‐YTHDF2/YBX3 Complex‐Mediated *DUSP1* Circularization and Translational Suppression

Notably, PROPER depletion significantly decreased the EIF2S3‐YTHDF2/YBX3 interaction, while ectopic expression of PROPER, but not PROPERmut, rescued the effect (Figure 6G,H), suggesting that these interactions depend on PROPER. Therefore, we propose that PROPER is a crucial member of the EIF2S3‐YTHDF2/YBX3 complex and functions as a “rivet” binding to EIF2S3 and enhancing their interaction (Figure 6I). Next, we performed MS2 tethering assays to evaluate the effect of PROPER on mRNA circularization. A dual‐luciferase vector was constructed, which included a Firefly reporter and a Renilla luciferase controlling for different transfection efficiencies (Figure S8P,Q, Supporting Information). Consequently, the translation efficiency decreased when EIF2S3 was tethered to the 5′‐UTR of reporter mRNA (Figure 6J,K), indicating that PROPER promoted *DUSP1* circularization and translational suppression. Collectively, these results indicate that the role of PROPER in translation suppression is to serve as a component of the translation initiation complex, promoting the EIF2S3‐YTHDF2/YBX3 complex‐mediated *DUSP1* circularization and translational suppression.

### 2.9 PROPER Regulates DUSP1 Expression that Contributes to PCa Progression

**Figure 7:**
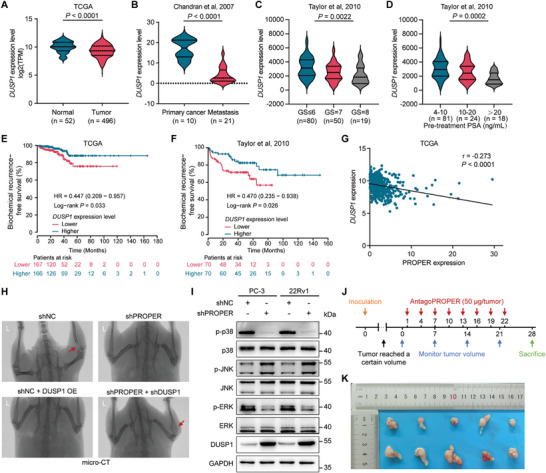
PROPER regulates DUSP1 expression that contributes to PCa progression. A,B) *DUSP1* downregulated in primary PCa tissues (A) and metastasis PCa (B). *P* values were calculated using unpaired two‐tailed Student's *t*‐tests. C,D) *DUSP1* downregulated in patients with high Gleason score (C) and high pre‐treatment PSA (D); GS, Gleason score. *P* values were calculated using one‐way ANOVA. E,F) Lower levels of *DUSP1* correlate with increased risk of biochemical recurrence in two cohorts of PCa patients. *P* values were assessed by a log rank test. G) Spearman's rank correlation coefficient analysis between PROPER and *DUSP1* expression in tumors from TCGA database. H) Representative Micro‐CT images of the hindlimbs of mice 9 weeks after inoculation with the PC‐3 cells. I) Western blotting analysis of the regulations of PROPER on p38 MAPK pathway. J) Timeline schematic for treatment of mouse tumors with antagoPROPER. Colored arrows indicate the times when different events occurred. K) Inhibitory effects of intra‐tumor administration of antagoPROPER on xenograft growth. Shown are pictures of tumors with (upper) or without (bottom) injection of antagoPROPER (*n* = 5).

To explore whether *DUSP1* is associated with human PCa progression, we performed clinical correlation analyses in multiple independent clinical PCa datasets. The results indicated that *DUSP1* expression levels were greatly downregulated in primary and metastatic prostate tumors compared to the normal prostate gland (**Figure** **7**A,B; Figure S9A–D, Supporting Information). In addition, the downregulation of *DUSP1* significantly correlated with a higher Gleason score, pre‐treatment prostate‐specific antigen (PSA) levels, and tumor progression in patients (Figure 7C,D; Figure S9E, Supporting Information). Moreover, patients with lower mRNA levels of *DUSP1* has an increased risk of postoperative biochemical recurrence and a shorter overall survival time (Figure 7E,F). We also observed that the expression of PROPER was negatively correlated with that of *DUSP1* (Figure 7G). Mechanistically, the role of DUSP1 in PCa progression and its impact on patient outcomes have been widely reported by negatively regulating p38 MAPK signaling pathway.^[^ ^21^, ^39^, ^40^ ^]^ The bone metastasis model of PCa showed that knockdown of PROPER expression or elevation of DUSP1 expression significantly inhibited tumor cell‐induced bone erosion of proximal tibiae compared to the control group (Figure 7H; Figure S9F, Supporting Information). However, DUSP1 knockdown significantly interfered with the inhibitory effect of PROPER knockdown on PCa metastasis (Figure 7H; Figure S9F, Supporting Information). The knockdown of PROPER led to a decrease in the phosphorylation of p38 and ERK (Figure 7I).

To test whether PROPER might be a therapeutic target for PCa treatment, we conducted experimental therapy in mouse subcutaneous xenograft models using antagoPROPER. We injected antagoPROPER or a scramble sequence directly into subcutaneous tumors (Figure 7J). After 5–8 injections, the subcutaneous tumors treated with antagoPROPER showed significantly reduced tumor volume and weight compared to those treated with scramble controls (Figure 7K; Figure S9G–I, Supporting Information). These results imply that targeted inhibition of PROPER expression may inhibit PCa progression.

## 3 Discussion

In the present study, we have identified a PCa risk‐associated SNP, rs17201241, located within an oncogenic piRNA called PROPER, which is associated with malignant phenotypes and aggressiveness of PCa. Mechanistically, PROPER interacts with the m^6^A reading proteins YTHDF2 and YBX3 to form pi‐RISC. This pi‐RISC promotes the degradation and translational suppression of *DUSP1* with the translation initiation factor EIF2S3 in an mRNA‐looping manner. These findings reveal a PROPER‐YTHDF2/YBX3/EIF2S3‐*DUSP1* signaling axis and uncovered a novel post‐transcriptional epigenetic mechanism of piRNA that guides certain mRNA degradation and translation control, based on an m^6^A methylation‐regulated mRNA‐looping.

To date, GWAS‐identified risk‐associated SNPs mainly map to noncoding regions of the genome.^[^ ^41^ ^]^ However, few studies have focused on genetic variants of piRNAs in somatic tissues. Our previous work revealed that rs11776042 in a lncRNA‐derived piRNA, piR‐015551, was significantly associated with a decreased risk of colorectal cancer.^[^ ^42^ ^]^ The present study has revealed for the first time that the SNP rs17201241 resides in a snoRNA‐derived piRNA cluster associated with PCa risk. Numerous functional piRNAs have been reported to be derived from snoRNAs.^[^ ^27^, ^43^ ^]^ By combining piRNA features and sequence alignment analysis of small RNA sequencing data,^[^ ^44^ ^]^ we successfully identified a novel piRNA called PROPER. Understanding the functional implications of PCa risk‐associated SNPs provides new opportunities to elucidate the mechanisms underlying PCa tumorigenesis.^[^ ^45^ ^]^ Through eQTL analysis, RNA‐protein docking analysis, and experimental studies, we confirmed that rs17201241 could affect the maturation of SNORD48, leading to high expression of PROPER in PCa tissues. Our previous work has systematically summarized the expression patterns of piRNAs in somatic tissues and their associations with clinical outcomes.^[^ ^43^ ^]^ In this study, we demonstrated that PROPER is an oncogenic piRNA in association with aggressive PCa.

Biologically, piRNAs were initially identified for their well‐known functions in repressing transposable elements in germline cells.^[^ ^46^ ^]^ Liu and her colleagues also discovered that MIWI/piRNA has a dual function, instructing both massive mRNA elimination and activating mRNA translation during spermiogenesis.^[^ ^34^, ^47^ ^]^ Intriguingly, our results uncovered three piRNA‐binding proteins, YTHDF2, YBX3, and EIF2S3, that are also significantly linked to translational regulation.^[^ ^36^, ^48^ ^]^ Dysregulation of the canonical translation machinery and abnormal activation of translation signaling pathways play critical roles in enhancing rapid translation of oncogenes or inhibiting tumor suppressor genes.^[^ ^49^ ^]^ A closed‐loop model mediated by piRNA and multiple RBPs is proposed to regulate multiple rounds of mRNA translation. This is supported by functional and physical circularization between the capped 5′ terminus and the polyadenylated 3′ terminus of mRNA mediated by the eIF4F‐PABPC1 complex,^[^ ^50^ ^]^ which maybe the critical hub for cancer development.^[^ ^51^, ^52^, ^53^ ^]^ Sun et al. reported a micropeptide, APPLE, in acute myeloid leukemia (AML).^[^ ^54^ ^]^ APPLE enhances the PABPC1‐eIF4G interaction, promoting mRNA circularization and the assembly of the eIF4F initiation complex, thereby supporting a specific pro‐oncogenic translation program. Wei et al. reported a novel methylation‐independent function of METTL3 in the cytoplasm, where METTL3 promotes gastric cancer by upregulating mRNA translation of specific transcripts.^[^ ^55^ ^]^ METTL3 interacts with PABPC1, stabilizing its association with the cap‐binding complex eIF4F, preferentially enhancing the translation of epigenetic factors without m^6^A modifications. However, unlike typical eIF4G–PABPC1‐mediated mRNA looping, we presented the first evidence of this EIF2S3‐YTHDF2/YBX3 loop, which presumably suppresses translation through the piRNA‐guided m^6^A‐modified mRNA degradation mediated by different translation initiation factors and patterns of epigenetic modification.

## 4 Conclusion

Altogether, this study revealed a novel PCa risk‐associated genetic variant, rs17201241, located within the piRNA PROPER. These findings support the notion that PROPER exerts an oncogenic role in PCa by acting as a translation co‐regulator in the EIF2S3‐YTHDF2/YBX3 loop axis. The presence of this piRNA as a driver in cellular malignant transformation highlights the similarities between tumorigenesis and gametogenesis, providing valuable insights into our understanding of tumorigenesis and presenting new opportunities for targeting the translation mechanisms in cancer cells.

## 5 Experimental Section

### Study Subjects

Three GWASs were included in a meta‐analysis, consisting of East Asian (i.e., Chinese and Japanese) and European populations.^[^ ^56^, ^57^, ^58^ ^]^ Briefly, the Chinese GWAS including 1151 cases and 1202 controls was part of the Consortium for Prostate Cancer Genetics (ChinaPCa);^[^ ^8^ ^]^ the cases were recruited from hospital‐based and pathologically diagnosed PCa patients and the cancer‐free controls from the communities or physical examination centers. The Japanese GWAS had 5408 cases and 103939 controls with data and samples available from the BioBank Japan Project (BBJ) that established in the Institute of Medical Science at the University of Tokyo.^[^ ^59^ ^]^ From the BBJ, pathologically proven PCa cases were selected. Control samples from BBJ were from four population‐based prospective cohorts, including the Tohoku University Tohoku Medical Megabank Organization (ToMMo),^[^ ^60^ ^]^ Iwate Medical University Iwate Tohoku Medical Megabank Organization (IMM), the Japan Public Health Center‐based Prospective Study,^[^ ^61^ ^]^ and the Japan Multi‐institutional Collaborative Cohort Study.^[^ ^62^ ^]^ The European GWAS from the Prostate Cancer Association Group to Investigate Cancer Associated Alterations in the Genome (PRACTICAL) is an international prostate cancer consortium that includes 79148 cases and 61106 controls (http://practical.ccge.medschl.cam.ac.uk/).^[^ ^58^ ^]^

All participating studies obtained an informed consent from all participants by following the protocols approved by their institutional ethical committees before enrollment, and the ethical committee at each institute approved the project.^[^ ^56^, ^57^, ^58^ ^]^

### Clinical Specimens

An additional 31 pairs of PCa and adjacent normal tissues were used to analyze RNA expression by RT‐qPCR, and another 90 pairs of PCa and adjacent normal tissues were analyzed using a high‐throughput tissue microarray analysis. The paired normal tissue and blood samples from these 90 patients were collected, and their RNA and DNA were extracted for the expression quantitative trait loci (eQTL) analysis. All the tissues were originally collected from patients who underwent surgical treatment and stored in liquid nitrogen until use. Serum was isolated from blood samples and stored at −80 °C. The present study was approved by the Nanjing Medical University Ethics Committee (Approval Number: IACUC‐2009008), and an informed consent was obtained from all patients and volunteers.

### Polysome Profiling

Cells were treated with 100 µg mL^−1^ cycloheximide (CHX) (ACMEC) at 37 °C for 15 min to inhibits protein synthesis by blocking translation elongation. Subsequently, the cells were washed twice with ice‐cold phosphate‐buffered saline (PBS) containing CHX and collected by cell scraping. Then cells were lysed in lysis buffer (20 mm Tris‐HCl, pH 7.5, 5 mm MgCl_2_, 100 mm KCl, 1% Triton X‐100) supplemented with 50 U mL^−1^ Ribonuclease Inhibitor (Promega), 100× Protease Inhibitor Cocktail EDTA Free (Abcam), 100 µg mL^−1^ CHX. Cell lysates were centrifuged at 15,000 rpm at 4 °C for 10 min and the supernatant was loaded onto a linear 10% to 45% (wt/vol) sucrose gradients followed by ultracentrifugation with a SW40Ti rotor (Beckman) at 35,000 rpm for 3 h. Absorbance at 260 nm was recorded using a BioComp Piston Gradient Fractionator equipped with a Bio‐Rad Econo UV Monitor. Polysome to monosome (P/M) ratios were calculated by comparing the areas under the monosome and polysome peaks. The extracted RNA and protein samples were then used for RT‐qPCR and western blotting analysis, respectively. Relative distribution of mRNA in each fraction was normalized by the total abundance of mRNA in all fractions marked as 100%.

### Surface Sensing of Translation (SUnSET) Assay

SUnSET assays were performed according to previously reported methods to monitor protein synthesis.^[^ ^63^ ^]^ Briefly, cells were incubated with 10 ug mL^−1^ puromycin (InvivoGen) for 30 min at 37 °C, followed by washing with cold‐PBS and scraped into RIPA lysis buffer (Beyotime). Then, puromycin‐containing proteins were analyzed by western blotting using the puromycin antibody (Sigma‐Aldrich).

### Small RNA Library Construction, Sequencing, and Data Analysis

Total RNA was extracted from PC‐3 and 22Rv1 cells using TRIzol reagent (Invitrogen). The concentration and quality of RNA were assessed by a NanoDrop ND‐1000 (NanoDrop) and an Bioanalyzer 2100 (Agilent). Following passing the quality control tests, total RNAs were used for library preparation and sequencing. Briefly, small RNAs of 18–40 nt in length were purified from total RNA by size fractionation using 15% PAGE and sequentially ligated to 5′ and 3′ adaptors, followed by RT‐PCR amplification to produce sequencing libraries using the TruSeq Small RNA Sample Prep Kit (Illumina) according to the manufacturer's protocol. PCR products were gel‐purified and sequenced using Illumina HiSeq 2000 (Illumina). Clean reads were obtained by removing low‐quality reads and adaptor sequences from raw reads. Subsequently, the length distribution of the clean reads and common and specific sequences between these two samples were summarized. Small RNA reads were aligned with piRBase (http://bigdata.ibp.ac.cn/piRBase/) to screen and annotate piRNAs using Bowtie.^[^ ^64^ ^]^

### m^6^A RNA Immunoprecipitation (MeRIP)‐Seq and RNA‐Seq

Total RNA from PC‐3 cells treated with PROPER knockdown and their negative controls was isolated using TRIzol reagent (Invitrogen), and the samples were quantified using NanoDrop ND‐1000 (NanoDrop). The RNA integrity was assessed by Bioanalyzer 2100 (Agilent) with RIN number >7.0, and confirmed by electrophoresis with denaturing agarose gel. Approximately more than 25 µg of total RNA was used to deplete ribosomal RNA according to the protocol of the Epicentre Ribo‐Zero Gold Kit (Illumina). Following purification, the ribosomal‐depleted RNA was fragmented into small pieces using Magnesium RNA Fragmentation Module (NEB) under 86 °C for 7 min. Then the cleaved RNA fragments were incubated for 2 h at 4 °C with m^6^A‐specific antibody (Synaptic Systems) in IP buffer (50 mm Tris‐HCl, 750 mm NaCl and 0.5% Igepal CA‐630). Then the IP RNA was reverse‐transcribed to create the cDNA by SuperScript II Reverse Transcriptase (Invitrogen), which was next used to synthesise U‐labeled second‐stranded DNAs with *E. coli* DNA polymerase I (NEB), RNase H (NEB), and dUTP Solution (Thermo Fisher Scientific). The libraries were subjected to denaturation to obtain single‐stranded DNA molecules and captured on Illumina flow cells. An A‐base was then added to the blunt ends of each strand, preparing them for ligation to the indexed adapters. Each adapter contains a T‐base overhang for ligating the adapter to the A‐tailed fragmented DNA. Single‐ or dual‐index adapters were ligated to the fragments, and size selection was performed with AMPureXP beads. After the heat‐labile UDG enzyme (NEB) treatment of the U‐labeled second‐stranded DNAs, the ligated products were amplified with PCR. The average insert size for the final cDNA library was 300 ± 50 bp. At last, the 2 × 150 bp paired‐end sequencing (PE150) was performed on an illumina Novaseq 6000 (LC‐Bio Technology) following the vendor's recommended protocol.

The fastp software (https://github.com/OpenGene/fastp) was used to remove the reads that contained adaptor contamination, low quality bases and undetermined bases with default parameter. Then sequence quality of IP and Input samples were also verified using fastp. HISAT2 (http://daehwankimlab.github.io/hisat2) was used to map reads to the reference genomeHomo sapiens (Version: v101). Mapped reads of IP and input libraries were provided for R package exomePeak (https://bioconductor.org/packages/exomePeak), which identifies m^6^A peaks with bed or bigwig format that could be adapted for visualization on the IGV software (http://www.igv.org). MEME (http://meme‐suite.org) and HOMER (http://homer.ucsd.edu/homer/motif) were used for de novo and known motif finding followed by localization of the motif with respect to peak summit. Called peaks were annotated by intersection with gene architecture using R package ChIPseeker (https://bioconductor.org/packages/ChIPseeker). Then StringTie (https://ccb.jhu.edu/software/stringtie) was used to perform expression level for all mRNAs from input libraries by calculating FPKM (total exon fragments /mapped reads (millions) × exon length (kB)). The differentially expressed mRNAs were selected with log2 (fold change) >1 or log2 (fold change) < ‐1 and *P* value < 0.05 by R package edgeR (https://bioconductor.org/packages/edgeR).

### CRISPR/Cas9‐Mediated Knockout

For SNORD48 KO, the single guide RNAs (sgRNAs) were designed using the online CRISPR design tool (Red Cotton, Guangzhou, China, https://en.rc‐crispr.com/). The pair of oligos for two targeting sites was annealed and ligated to the YKO‐RP006 vector (Ubigene). The YKO‐RP006‐hRABL6 [gRNA] plasmids containing each target sgRNA sequences were transfected into cells with Lipofectamine 3000 (Thermo Fisher Scientific). After 24–48 h, 1 µg mL^−1^ puromycin (InvivoGen) was added to screen the cells; surviving cells were sorted into 96‐well plates and then expanded into 12‐well plates. Selection of single clones was performed after 2–4 weeks, and the selected SNORD48 KO clones were validated by PCR and Sanger sequencing. The sgRNAs and primers for CRISPR design are shown in Table S10 (Supporting Information).

### Immunoprecipitation (IP)

Cell extraction was performed using IP Lysis Buffer (50 mm Tris‐HCl pH 7.4, 1 mm EDTA, 50 mm NaCl, 1% Triton X‐100) supplemented with Halt Protease Inhibitor Cocktail (Thermo Fisher Scientific), and then incubated with primary antibody overnight at 4 °C. Next day, the antibody‐bound protein of interest in lysis buffer was incubated with 40 µL of Protein A/G Beads (Thermo Fisher Scientific). After three washes with Wash Buffer (0.5 m Tris‐HCl pH 7.4, 1.5 m  NaCl), protein‐bound beads were mixed with 5 × loading buffer (Thermo Fisher Scientific) to the final concentration of 1 × loading buffer and boiled for 10 min at 95 °C. The samples were then stored at 20 °C or ready for sodium dodecyl sulfate‐polyacrylamide gel electrophoresis (SDS‐PAGE). To explore whether protein interaction was mediated by RNA, RNase A (Takara) was added into the cell lysate to the final concentration of 20 mg mL^−1^ and incubated for 30 min at 37 °C before IP procedures.

### Proximity Ligation Assay (PLA) Assay

PC‐3 cells were transfected with plasmids of FLAG‐YTHDF2, HA‐YBX3, and MYC‐EIF2S3. After 48 h, cells were incubated with primary antibodies (two antibodies raised in different species) in blocking solution at 4 °C for 2 h. Cells were then washed in PBS plus 0.1% Tween 20 for 2 × 5 min. Then, cells were incubated with PLA probes (anti‐Rabbit‐PLUS and anti‐Mouse‐MINUS) (Sigma, DUO92101) for 60 min at 37 °C. Cells were washed in 1× Wash Buffer A for 2 × 5 min at 37 °C. Then, dilute the Ligation stock and the Ligase according to the manufacturer's instruction. The mix was applied to cells and incubated for 30 min at 37 °C. Cells were washed in 1x Wash Buffer A for 2 × 2 min. Then, dilute the amplification stock and the polymerase according to the manufacturer's instruction. Then, the mix was applied to to cells and incubated for 100 min at 37 °C. Cells were washed in 1x Wash Buffer A for 2×10 min and wash in 0.01× Wash Buffer B for 1 min. Mount the cells with Duolink In Situ Mounting Medium with DAPI, and cells were observed in a confocal microscope (Carl Zeiss LSM 710).

### MS2 Tethering Assay

The MS2 tethering assay was performed as previously described.^[^ ^65^ ^]^ An artificial polypeptide eIF2S3 fused with MS2 was localized downstream of the reporter gene to generate pMS2‐pEGFP‐C1‐EIF2S3. Firefly luciferase (FLuc) represents a reporter RNA containing the firefly luciferase gene as a reporter. MS2‐binding sites (6, 12, or 24 copies) were inserted into the reporter RNA to generate Fluc‐6×MS2bs, Fluc‐12×MS2bs, and FLuc‐24×MS2bs, respectively. Translation of the reporter was greatly enhanced by the EIF2S3‐MS2 fusion protein. Moreover, EIF2S3‐MS2 tethered at the 3′‐UTR enhanced translation. Dual luciferase assays were performed according to the manufacturer's instructions (Promega). The FLuc activity was normalized to *Renilla* luciferase activity to calculate the different transfection efficiencies.

### Animal Models and In Vivo Imaging

Animal experiments were carried out in compliance with approved protocols and guidelines. To examine the effects of PROPER on subcutaneous xenograft growth, male BALB/c nude mice (5 mice per group) were subcutaneously injected with 0.1 mL of cell suspension containing 2 × 10^6^ cells in the rear flank. When a tumor was palpable, it was measured every other day and its volume was calculated according to the formula volume = length × width^2^ × 0.5.

Subcutaneous xenografts were also treated with antagoPROPER, a synthesized PROPER inhibitor (Ribobio; Table S10, Supporting Information) by intra‐tumor injection. Briefly, when tumors implanted in mouse flanks reached a certain volume, 50 µg of AntagoPROPER in 50 µL phosphate buffered saline was directly injected into the tumor. The tumor was injected with AntagoPROPER NC (Ribobio; Table S10, Supporting Information) as control. Treatment was delivered every three days for 22 days and tumor volume was measured before each injection. Mice were sacrificed at the end of the experiment and the xenografts were stripped and photographed. H&E staining was then performed for histological examination of prostate tumor.

To generate a metastasis model, PC‐3 stable cells (2  × 10^6^) which containing control vector or PROPER shRNA‐expressing vector were intratibial injected into the tibia of BALB/c nude mice (4‐week‐old). The mice were humanely sacrificed after 15 weeks. The tumor mass was removed from the tibia bone to detect its weight, and the body weight and survival time of animals in each group were recorded.

Animal studies were approved by the Institutional Animal Care and Use Committee of Nanjing Medical University.

### Statistical Analysis

Multivariable logistic regression analyses were used to estimate odds ratios (ORs) and the corresponding 95% confidence intervals (CIs) for the associations of candidate SNPs with PCa risk after adjustment for covariates of age and the top principal components. The meta‐analysis of three GWAS dataset was performed using a fixed‐effect model. The heterogeneity of studies was tested by the Cochran's *Q* statistic and *I* ^2^. The correlations between SNP genotypes and gene expression levels were evaluated using a linear regression model.

All quantitative experiments were repeated using at least four independent biological repeats and were presented as mean ± s.d. Statistical comparisons between two groups were accessed by two‐tailed Student's *t*‐test and two‐sided Mann‐Whitney *U*‐test. Details of the statistical analysis for each experiment can be found in the relevant figure legends. Kaplan‐Meier survival data were analyzed using the log‐rank test. All image quantifications for migration and invasion experiments were performed using ImageJ2 (version 10.2). All statistical analyses were performed using RStudio (version 1.0.136) with R (version 3.3.3) and GraphPad Prism 6 software.

### Ethics Approval

The present study was approved by the Nanjing Medical University Ethics Committee (Approval Number: IACUC‐2009008), and an informed consent was obtained from all patients and volunteers.

## Conflict of Interest

The authors declare no conflict of interest.

## Author Contributions

S.B., Z.T.D., and J.Y.X. contributed equally to this work. M.L.W. and G.C. conceived the study. S.B., Z.T.D., and J.Y.X. performed computational work. Z.T.D., L.L.F., and S.L.C. performed experimental work. G.C. and Y.F.C. contributed to sample collection. S.L.C. managed sample recruitment. F.L., M.L.D., Z.D.Z., and G.H.W. contributed to critical reading and revision of the manuscript. All authors participated in interpretation and writing the manuscript.

## Supporting information

Supporting Information

## Data Availability

The data that support the findings of this study are available on request from the corresponding author. The data are not publicly available due to privacy or ethical restrictions.
