# Supplementary material for: piRNA PROPER Suppresses DUSP1 Translation by Targeting N 6‐Methyladenosine‐Mediated RNA Circularization to Promote Oncogenesis of Prostate Cancer
Source: Adv Sci (Weinh). 2024 Jul 4;11(33):2402954. doi: 10.1002/advs.202402954 (PMC11434016; doi:10.1002/advs.202402954)
Supplement: Supplementary file 1 — Supporting Information [file ADVS-11-2402954-s001.docx]

Supporting information

Supplementary Materials and Methods

**Genotyping, imputation, and quality control**

The Chinese GWAS had genotype data generated by the Illumina Human OmniExpress BeadArray, the Japanese GWAS data were generated by the Illumina Human610-Quad BeadChip for cases and HumanHap550v3 BeadChip for controls, and the European genotype data were generated in the UK Biobank using the Affymetrix UK Biobank Axiom Array and the Affymetrix UK BiLEVE Axiom Array. Imputation was performed using the Haplotype Reference Consortium, UK10K and 1000 Genomes Project panels. In addition to quality-control procedures performed in previous studies, SNPs with genotyping rate of < 95%, MAF < 0.05 or *P* < 0.001 in a Hardy-Weinberg Equilibrium test were further removed before imputation analysis.

SnoRNA and piRNA expression data mining

The expression of snoRNAs of PCa were generated by small RNA sequencing data from The Cancer Genome Atlas (TCGA) database.^[1]^ We downloaded snoRNA expression data from the website address: http://bioinfo.life.hust.edu.cn/SNORic/download/. The processing methods and criteria of piRNA expression data obtained from the TCGA database have been described previously.^[2]^ Briefly, only the piRNAs with a count per million mapped reads (CPM) ≥ 1 and those expressed in ≥ 20% of samples were retained for further analyses.

Cell culture

One normal prostate epithelial cell line (RWPE-1) and four PCa cell lines (AR+, 22RV1 and LNCaP; AR−, PC-3 and DU 145) were used. All cell lines were from American Type Culture Collection (ATCC). RWPE-1 cells were maintained in DMEM high glucose medium (Gibco), 22RV1 cells were maintained in DMEM/F-12 medium (Gibco), and PC-3, DU-145 and LNCaP cells were maintained in RPMI-1640 medium (Gibco). All medium were added with 10% fetal bovine serum (Gibco), 1% penicillin/streptomycin (Gibco). Cell cultures were maintained at 37°C in a 5% CO_2_ atmosphere.

RNA isolation and RT-qPCR

Total RNA was extracted by TRIzol Reagent (Invitrogen). RT-qPCR was performed using RT reagent Kit with gDNA Eraser (Takara) and Premix Ex Taq II (Takara). Specially, piRNA was reversed into cDNA by RevertAid First Strand cDNA Synthesis Kit (Thermo Fisher Scientific). The relative levels of gene expression were analyzed by the 2^−ΔΔCT^ method. Nuclear and cytoplasmic fractionation was separated using the PARIS kit (Thermo Fisher Scientific) according to manufacturer’s instructions, followed by RNA extraction and RT-qPCR. All primer sequences are listed in Supplementary Table S10.

**Short interfering RNA (siRNA) and plasmid construction**

PCa cells were transfected with siRNA oligonucleotides (GenePharma) and the transfection effects were verified by RT-qPCR and western blotting. Transfection of siRNAs was performed using Lipofectamine 3000 (Invitrogen) according to the manufacturer’s instructions. All siRNA sequences used are listed in Supplementary Table S10.

Constitutive gene expression was performed by cloning the cDNA of interest into pCDNA3.1 (Thermo Fisher Scientific) or pGC-FU vectors (genechem). The full-length of truncated YTHDF2, EIF2S3 and YBX3 were cloned into pcDNA3.1 vector to express FLAG-YTHDF2, MYC-EIF2S3 and HA-YBX3 proteins; the cDNA of PROPER and SNORD48 were cloned into pGC-FU vector for stable RNA expression. Stable SNORD48 interference and the negative control were performed using small hairpin RNA (shRNA) which was cloned into pGL3-MMLV-LTR-Luc shRNA cloning and Expression Vector (Addgene). The sgRNA and siRNA/shRNA sequences were listed in Supplementary Table S10.

In addition, the fragment in 3′-UTR of *DUSP1* covering the PROPER binding site were cloned into pmirGLO Vector (Promega). In addition, seven nucleotides within the predicted PROPER binding site were mutated in the pmirGLO Vector (Promega). Sequences of constructed plasmids were confirmed by Sanger sequencing. The two plasmids were used to evaluate PROPER activity by inserting piRNA target sites into the 3′-UTR of the Firefly Luciferase Gene.

**Northern blot**

The Northern blot analysis was performed using the NorthernMax-Gly kit (Ambion, Carlsbad, CA, USA). Briefly, 20 μg of RNA were denatured with a glyoxal/DMSO treatment, fractionated in a 1.5% agarose gel, transferred to a nylon membrane and fixed by UV cross-linking. The RNA blot was hybridized at 68 °C overnight with an antisense biotin-labeled RNA probe that was complementary to the target. The bound biotin-labeled probe was detected using the BrightStar BioDetect kit (Ambion) according to the manufacturer’s instructions. A probe that was complementary to the piRNA was used. A 2% agarose gel was used to detect the small RNA fragments. The band intensity was quantified using the Gel-Pro analyzer software (Media Cybernetics, Rockville, MD, USA).

Periodate oxidation and alkaline ß-elimination

Periodate oxidation and alkaline ß-elimination was performed as previously describe.^[3]^ Briefly, total small RNAs were isolated using miRneasy Mini kit (Qiagen) according to the manufacture’s protocol. Each sample was split in two portions, each containing 25µg of RNA and independently treated with either 200 mM of sodium periodate or water, and RNA precipitated by ethanol. As positive control for the assay, a synthetic piRNA sequence (corresponding to one of the most abundant piRNA found in prostate cancer: piR-hsa-1681) modified with the 3′-end 2′-*O*-methylation. As negative control for the assay, a synthetic miRNA sequence (corresponding to one of the most abundant miRNA found in prostate cancer: miR-21) without 3′-end 2′-*O*-methylation modification. All these samples were subject to the periodate treatment as above. The samples and control oligos were finally quantified by qPCR-TaqMan small RNA assay. Note that presence of 3′-end 2′-*O*-methylation in positive control and piRNAs confers them resistance to periodate oxidation and alkaline ß-elimination, in contrast to their depletion in unmethylated negative control.

Western blotting

Using standard protocols, cells were collected and then lysed in RIPA lysis buffer (Thermo Fisher Scientific) supplemented with protease and phosphatase inhibitors (Thermo Fisher Scientific). Membranes were then incubated overnight with primary antibodies against the target genes or GAPDH (Proteintech) at a 1:1,000 dilution. After three washes with TBS-T, immunoreactions were visualized with an HRP-conjugated secondary antibody and enhanced chemiluminescence (ECL) reagents (Thermo Fisher Scientific) according to the manufacturers’ instructions.

**Recombinant protein expression and purification**

Full-length His-purified YTHDF2 protein, GST-purified EIF2S3 proteins, His-purified YBX3 protein were expressed in BL21 (DE3) *E. coli* cells and were purified using Ni-NTA resin (GE Healthcare) followed by gel filtration. The bacteria colony was inoculated in liquid Luria-Bertani (LB) medium, and grew at 37 °C overnight while being shaken at 220-250 rpm. When the OD600 value of bacterial culture reached 0.8, Isopropyl β-D-Thiogalactoside (IPTG) was added to the culture medium at a final concentration of 0.24 mg/mL, and the bacteria were grown at 16 °C for another 24 h. The bacteria cells were harvested by centrifugation at 5000 g for 30 min at 4 °C and frozen at -80 °C until use. The cell pellet was sonicated at 4 °C in lysis buffer, and the mixture was centrifuged at 10,000 g for 30 min at 4 °C. The supernatant was transferred into a dialysis bag and concentrate with PEG6000. The recombinant proteins were purified using Ni-NTA resin (GE Healthcare) according to the manufacturer's instructions. Ultrafiltration centrifuge was used to concentrate the purified proteins. The purified proteins were then treated with RNase A and DNase I at 37 °C and purified again using Ni-NTA resin. The protein concentration was measured by the BCA Protein Assay Kit.

RNA immunoprecipitation (RIP)

The RIP assay was performed according to the recommended conditions using RNA Immunoprecipitation Kit (Sigma-Aldrich). Briefly, 2 × 10^7^ cells were harvested, resuspended in 100 μL RIP Lysis Buffer supplemented with 0.5 μL of protease inhibitor cocktail and 0.25 μL of RNase inhibitor, and kept on ice for 5 min. Antibody or a corresponding control IgG was conjugated to Magnetic Beads Protein A/G by incubation for 30 min, followed by washing three times and incubation with pre-cleared nuclear extraction in RIP Immunoprecipitation Buffer (0.5 M EDTA, 5 µL RNase inhibitor, and 860 µL of RIP Wash Buffer) at 4 °C overnight. After washing the beads total six times with 500 µL of cold RIP Wash Buffer, each immunoprecipitate was re-suspend in 150 µL of proteinase K buffer (117 µL of RIP Wash Buffer, 15 µL of 10% SDS, 18 µL of 10 mg/mL proteinase K) at 55 °C for 30 min with shaking to digest the protein. Input and co-immunoprecipitated RNAs were extracted by TRIzol reagent (Invitrogen) and analyzed by qPCR.

RNA pull-down assay and mass spectrometry analysis

Biotin-labelled RNAs was *in vitro* transcribed with HiScribe™ T7 Quick High Yield RNA Synthesis Kit (NEB) with Biotin-16-UTP (Roche), treated with RNase-free DNase I on column during RNA purification with RNA Clean & Concentrator-25 (Zymo Research). For each sample, 5 μg RNA was mixed with 1 × 10^7^ cell extract and incubated at 4 °C for 1 h, followed by incubating with Dynabeads M-280 Streptavidin (Invitrogen) at 4 °C overnight. After washes, the pull-down complexes were eluted by denaturation in 1 × protein loading buffer for 10 min at 100 °C. The samples were detected by western blotting or proceed to mass spectrometry.

After RNA pull-down, equal amounts of samples pulled down by sense and anti-sense PROPER were loaded on SDS-PAGE gel. Then the gel was stained with Silver Stain Kit (Thermo Fisher Scientific) according to the manufacturer’s instructions. Specific bands were cut and analyzed by LC-MS/MS. Protein identification was retrieved in the human RefSeq protein database (National Center for Biotechnology Information), using Mascot version 2.4.01 (Matrix Science).

Transwell migration and invasion assay

For invasion assays, cell-culture inserts (0.8 μm, Falcon) were coated with collagen type I (10 μg/insert) (BD Biosciences) in molecular-grade water and dried overnight. For migration assays, inserts were not coated but rehydrated with Opti-MEM (Invitrogen) and fibronectin (4 µg/insert) for 2 h, and 40,000 cells per insert were seeded in Opti-MEM. Complete media was used in the lower chamber. Following 24-48 h of migration or invasion, cells were fixed in 4% paraformaldehyde for 30 min, treated with RNase A (Invitrogen), and visualized with SYBR Safe (1:5000, Invitrogen) in PBS. Images were collected with a Nikon Eclipse Ti2 with NIS Elements imaging software (version 5.02) and were analyzed with ImageJ (version 10.2).

Cell proliferation, clonogenicity, migration, and invasion assays

For cell proliferation assays, the resuspended cells were plated in a 96-well plate at a density of 1,000 cells/well and cultured in a complete medium, and the cell numbers were quantified every 24 h with a CCK-8 kit (Dojindo) after 30 min of incubation. For clonogenicity assays, transfected cells were seeded in 6-well plates at a density of 300 cells/well in complete medium, incubated for two weeks, fixed with 10% paraformaldehyde, and stained with 0.05% crystal violet. For the cell migration and invasion transwell assays, 25,000 PC-3 and 22Rv1 cells in 500 μL starvation media were plated on the top chambers of Transwell Clear Polyester Membrane Inserts (Corning) and BioCoat Matrigel Invasion Chambers (Corning), while culture media with 20% FBS was applied on the bottom. After 48-72 h, migrated or invaded cells were stained with crystal violet and counted under a ×20 microscope.

Flow cytometry

Using an FITC Annexin V and propidium iodide (PI) staining kit (BD Bioscience), flow cytometric analyses were performed to detect the apoptosis rate of PCa cells. To detect the cell cycle distribution, cells were stained with PI after 48 h of transfection and detected in a BD FACSAri III Sorter (BD Bioscience).

Sequencing data obtain

YTHDF2 RIP-Seq data were obtained from GSE49339 in the public database Gene Expression Omnibus (GEO). CLIP-Seq data were used to detect the interaction between YTHDF2 and the 3′-UTR of *DUSP1* mRNA in HeLa cells (data from GSE49339, GSE86336), HER 393T cells (data from GSE63753), HEK 393T cells (data from GSE122948), A549 cells (data from GSE71154), and CD8 T cells (data from GSE71154). For PAR-CLIP-Seq, the YTHDF2 PAR-CLIP data were obtained from GSE49339. Distributions of YBX3-binding peaks across 3′-UTR of *DUSP1* in K562 and HepG2 cells were obtained from GSE91844 and GSE177585, respectively.

**Pathway enrichment analysis**

Differentially expressed mRNAs were detected using the limma package v3.48.0.51. Further Gene Ontology (GO) and Kyoto Encyclopedia of Genes and Genomes (KEGG) enrichment analysis was performed based on the Database for Annotation, Visualization, and Integrated Discovery (DAVID) v6.8.

Actinomycin D assay

PC-3 cells with stably expressed shRNAs against PROPER and YTHDF2 or controls were seeded into 6-well plates to get 50% confluency after 24 h. Cells were treated with 5 μg/mL actinomycin D and collected at indicated time points. The total RNA was extracted and analyzed by RT-qPCR.

RNA-protein docking simulations

The 3dRNA tool is a fast and automated method of building 3D RNA structure based on sequence and secondary structure, and it builds 3D RNA structure from the smallest secondary elements (SSEs).^[4, 5]^ RNA-binding protein (RBP) binding affinity is driven largely by RNA sequence and secondary structure.^[6]^ We used the minimum free energy algorithm implemented in 3dRNA to model the possible structures of SNORD48, PROPER and *DUSP1* by identifying optimal folding of a nucleic-acid sequence within a specified energy increment. Thus, the secondary structure with a lower theoretical value of free energy was then selected as a model structure for the 3D structure prediction. Finally, we chose the appropriate 3D structures according to the 3dRNA clusters assembled structures and 3dRNAscore for RNA-protein docking.

The ZDOCK tool offered by the Discovery Studio software is widely used for protein-protein and RNA-protein docking, which can accurately predict 3D structure of RNA-protein complexes.^[7]^ To determine possible interaction of SNORD48 with NOP56 and *DUSP1* (m^6^A modified sequence) with YTHDF2 (YTH-domain), We employed ZDOCK to carry out the in-silico molecular docking with default settings: a 1.2 Å grid step, variable grid size for fitting the size of a protein (considered as a receptor) and RNA (considered as ligand), with a 15° angle step for rotation of the ligand. The residues 385-386, 316, 163 in the NOP56 and the residues Y418, D422, C433, W432, W486, W499 in the YTHDF2 used in the docking procedure were derived from Protein Data Bank entry 7MQ9 and 4RDN, respectively. Then all possible poses were assigned with energy-based ZRANK scores that reranked the docking predictions from ZDOCK according to the potential energy, spatial complementarity, and electric field forces, clustering followed by refinement of best scored docked complexes from three biggest clusters. A lower ZRANK score indicated a superior docking result, and docking results between biomolecules were exported as PDB files.

The catRAPID is an algorithm for estimating the propensity for the RNA-protein binding. By combining secondary structure, hydrogen bonding, and van der Waals forces, the RNA-protein binding can be predicted using this algorithm with a high accuracy.^[8]^

Haematoxylin and eosin (H&E), Immunohistochemistry (IHC)

Tumors were harvested, fixed with 10% formalin, stained with H&E, and mounted with mounting medium. Images were acquired with a Nikon Eclipse Ti fluorescence microscope. H&E staining was utilized to select representative areas in a slide.

IHC was performed to evaluate the protein expression of Ki-67, Masson, and TRAP in tumor and bone metastases tissues of mice. The slides were removed from the paraffin in xylene and then rehydrated with ethanol. The slides were used for antigen retrieval with 0.01 M sodium citrate buffer (pH 6.0) and then incubated with 3% H_2_O_2_ for 15 min at room temperature to reduce the non-specific staining, followed by blocking with 5% bovine serum albumin (BSA) for 60 min. The slides were incubated with primary antibodies overnight at 4 °C. After washing, the slides were incubated with HRP conjugated secondary antibody incubation for 45 min at room temperature. Then, the 3-diaminobenzidine tetra-hydrochloride (DAB) was used to detect the antibody binding, and cells were counterstained with hematoxylin. Both positive and negative controls were included each time. Staining scores were evaluated according to the percentage of positive tumor cells and staining intensity score.

Fluorescent in situ hybridization (FISH) and immunofluorescence staining

FISH of PROPER was performed with DIG-labeled LNA probes (RiboBio). RNA localization and levels were determined with a FISH kit according to the manufacturer’s protocol (RiboBio). Cells were fixed and then incubated with PROPER probe overnight at 37 °C. After washing and sealing, cells were incubated with Cy3-conjugated lgG fraction monoclonal mouse anti-digoxin antibody (Jackson) for 1 h, followed by DAPI (Sigma-Aldrich) staining of the nuclei at room temperature for 10 min. For immunofluorescence staining of YTHDF2, YBX3, and EIF2S3 proteins, cells were harvested and were subsequently incubated with primary antibodies at 4 °C overnight and corresponding AF488-conjugated secondary antibodies (1:1000) (Invitrogen) at room temperature for 2 h. Cell nuclei were counterstained with DAPI (Sigma-Aldrich). Fluorescence was observed using confocal laser scanning microscopy (Carl Zeiss LSM 710).

**Micro-computed tomography (micro-CT) analysis**

After the mice were sacrificed, the tibia of tumor growth was dissected and fixed in 4% paraformaldehyde for micro-CT analysis. The tibia for micro-CT scanning was assessed by using a micro-CT scanner (Skyscan 1176, Bruker). The parameters of scanning setting were as follows: 50 kV, 500 uA, 0.5 mm Al filter, 65 ms exposure, pixel size 35.52 μm, 2 frame averaging, and 0.8-degree rotation step through 180°. A resolution as high as 9 μm was obtained in scanning CT images, and 3D reconstruction was established. Reconstruction of sections was carried out with GPU-based scanner software (NRecon, Bruker). In addition, the grayscale was based on the Hounsfield unit, and the validated calcium standards were scanned as the density reference. The three-dimensional microstructural volumes from the micro-CT scans were analyzed using Skyscan software (CTAn, Bruker). Bone volume ratio (BV/TV, %), trabecular number (Tb.N*), trabecular thickness (Tb.Th*), and trabecular separation (Tb.Sp*) was calculated to assess the bone resorption area of the bone metastasis.

**References:**

1. Gong J, Li Y, Liu C-J, Xiang Y, Li C, Ye Y, et al. A Pan-cancer Analysis of the Expression and Clinical Relevance of Small Nucleolar RNAs in Human Cancer. Cell Reports. 2017;21(7):1968-81.

2. Xin J, Du M, Jiang X, Wu Y, Ben S, Zheng R, et al. Systematic evaluation of the effects of genetic variants on PIWI-interacting RNA expression across 33 cancer types. Nucleic Acids Research. 2021;49(1):90-7.

3. Balaratnam S, West N, Basu S. A piRNA utilizes HILI and HIWI2 mediated pathway to down-regulate ferritin heavy chain 1 mRNA in human somatic cells. Nucleic Acids Research. 2018;46(20):10635-48.

4. Wang J, Mao K, Zhao Y, Zeng C, Xiang J, Zhang Y, et al. Optimization of RNA 3D structure prediction using evolutionary restraints of nucleotide–nucleotide interactions from direct coupling analysis. Nucleic Acids Research. 2017;45(11):6299-309.

5. Zhao Y, Huang Y, Gong Z, Wang Y, Man J, Xiao Y. Automated and fast building of three-dimensional RNA structures. Scientific reports. 2012;2(1):1-6.

6. Lambert N, Robertson A, Jangi M, McGeary S, Sharp PA, Burge CB. RNA Bind-n-Seq: quantitative assessment of the sequence and structural binding specificity of RNA binding proteins. Molecular cell. 2014;54(5):887-900.

7. Iwakiri J, Hamada M, Asai K, Kameda T. Improved accuracy in rna–protein rigid body docking by incorporating force field for molecular dynamics simulation into the scoring function. Journal of Chemical Theory and Computation. 2016;12(9):4688-97.

8. Agostini F, Zanzoni A, Klus P, Marchese D, Cirillo D, Tartaglia GG. cat RAPID omics: a web server for large-scale prediction of protein–RNA interactions. Bioinformatics. 2013;29(22):2928-30.


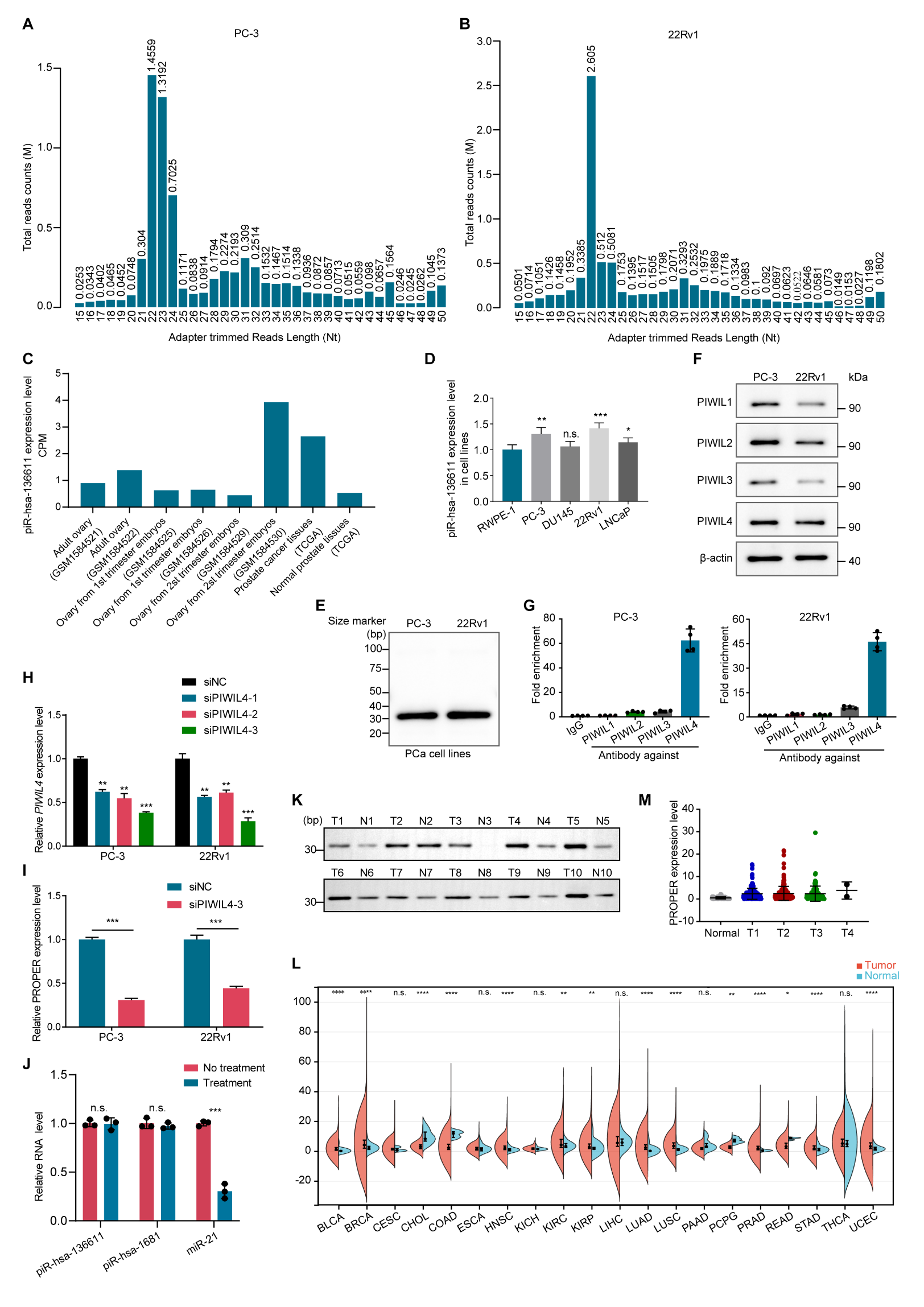


**Supplementary Figure S1. The expression characteristic and structure of piR-hsa-136611**. (A, B) Reads length from small RNA-Seq of PC-3 and 22Rv1 cells. (C) The expression level of piR-hsa-136611 in human prostate tissues (TCGA), human oocytes and early embryos (GEO accession: GSE64942). (D) Relative expression level of piR-hsa-136611 in normal and prostate cancer cell lines by RT-qPCR analysis. n.s., not significant; ^*^*P* < 0.05, ^**^*P* < 0.01, and ^***^*P* < 0.001 by Mann-Whitney *U*-tests. (E) Northern blot of piR-hsa-136611 in prostate cancer cell lines. (F) The expression of PIWI proteins by western blotting through specific antibodies. (G) RIP-qPCR analysis of PROPER immunoprecipitated using an anti-PIWIL1/2/3/4 antibody confirmed the interaction between PIWIL4 and PROPER in PC-3 and 22Rv1 cells. Data are presented as mean ± s.d. (n = 4). (H) RT-qPCR detection of the knockdown efficiency of PIWIL4 by siRNAs in PC-3 and 22Rv1 cells. Data are presented as mean ± s.d. (n = 4); ^**^*P* < 0.01, ^***^*P* < 0.001, and ^****^*P* < 0.0001 by Mann-Whitney *U*-tests. (I) RT-qPCR detection of piR-hsa-136611 expression when PIWIL4 knockdown in PC-3 and 22Rv1 cells. Data are presented as mean ± s.d. (n = 4); ^**^*P* < 0.01, ^***^*P* < 0.001, and ^****^*P* < 0.0001 by Mann-Whitney *U*-tests. (J) RNA relative expression of piR-hsa-136611, piR-hsa-1681, and miR-21 in PC-3 cells upon treatment with sodium periodate (NaIO4) or water and alkaline ß-elimination. Synthetic RNA oligos were used as negative (miR-21, unmethylated) and positive (piR-hsa-1681, 2′-*O*-methylated) controls, respectively. Note that presence of 3′-end 2′-*O*-methylation in piR-hsa-1681 confers them resistance to periodate oxidation and alkaline ß-elimination, in contrast to their depletion in unmethylated miR-21. Data are presented as mean ± s.d. (n = 3); n.s., not significant; ^***^*P* < 0.001 by Mann-Whitney *U*-tests. (K) Northern blot of PROPER in 10 paired prostate cancer and normal tissues. (L) The expression level of piR-hsa-136611 in multiple kinds of cancers. BLCA, Bladder urothelial carcinoma; BRCA, Breast invasive carcinoma; CESC, Cervical squamous cell carcinoma and endocervical adenocarcinoma; CHOL, Cholangio carcinoma; COAD, Colon adenocarcinoma; ESCA, Esophageal carcinoma; HNSC, Head and neck squamous cell carcinoma; KICH, Kidney Chromophobe; KIRC, Kidney renal clear cell carcinoma; KIRP, Kidney renal papillary cell carcinoma; LIHC, Liver hepatocellular carcinoma; LUAD, Lung adenocarcinoma; LUSC, Lung squamous cell carcinoma; PAAD, Pancreatic adenocarcinoma; PCPG, Pheochromocytoma and paraganglioma; PRAD, Prostate adenocarcinoma; READ, Rectum adenocarcinoma; STAD, Stomach adenocarcinoma; THCA, Thyroid carcinoma; UCEC, Uterine corpus endometrial carcinoma. ^*^*P* < 0.05, ^**^*P* < 0.01, ^***^*P* < 0.001, and ^****^*P* < 0.0001 by two-tailed Student’s *t*-test. (M) The expression level of PROPER in different tumor stage from TCGA database.


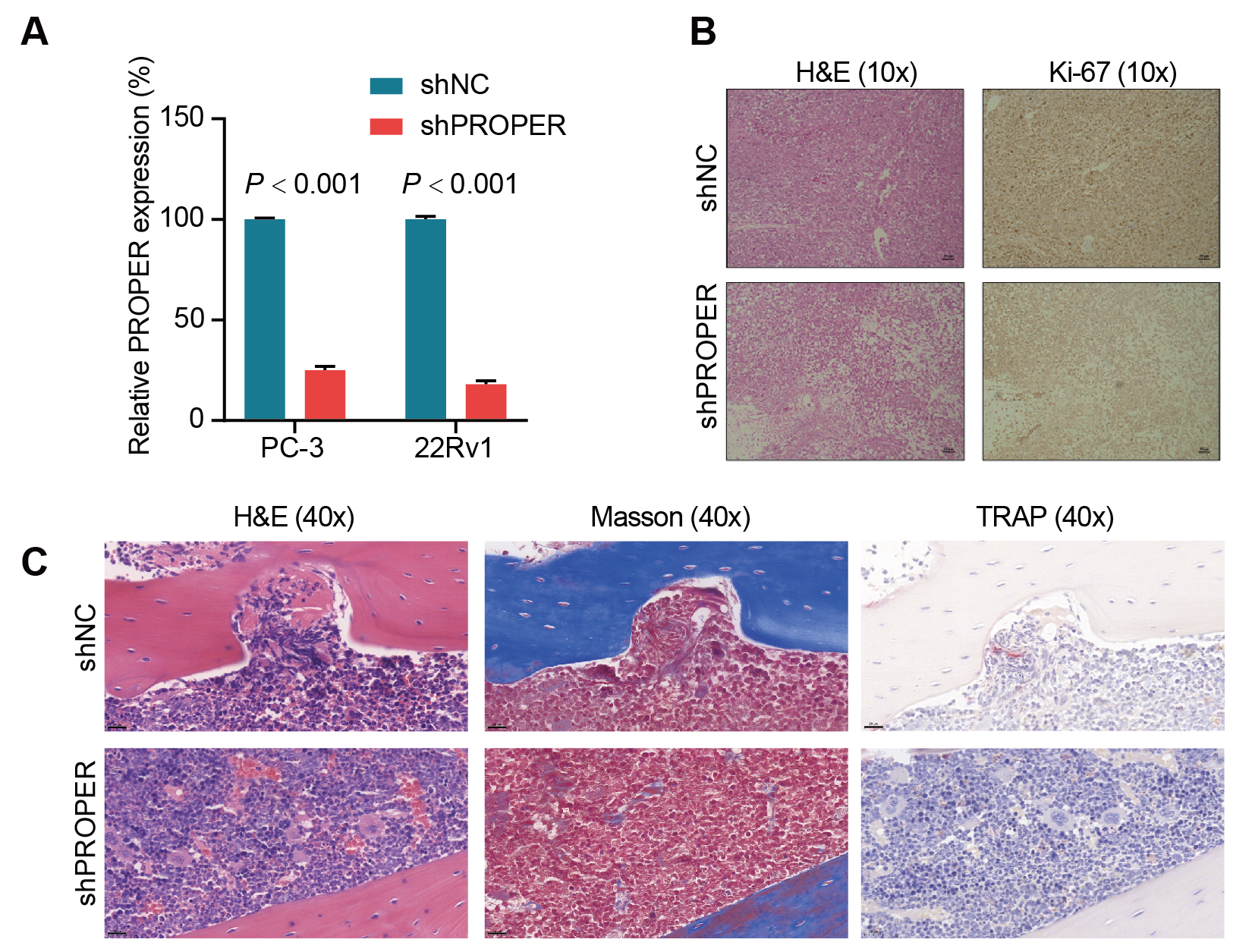


**Supplementary Figure S2. The effects of PROPER on tumor growth in a xenograft** **mouse model.** (A) The efficiency of shRNA-mediated PROPER knockdown was confirmed by RT-qPCR. (B) H&E (left) and Ki-67 (right) immunostaining of tumor tissue from nude mice xenografts; Scale bar, 50 μm. (C) Representative H&E (left) Masson (middle), and TRAP (right)-stained bone metastases tissues produced by the growth of PC-3 cells in the tibia of a nude mouse; Scale bar, 20 μm.


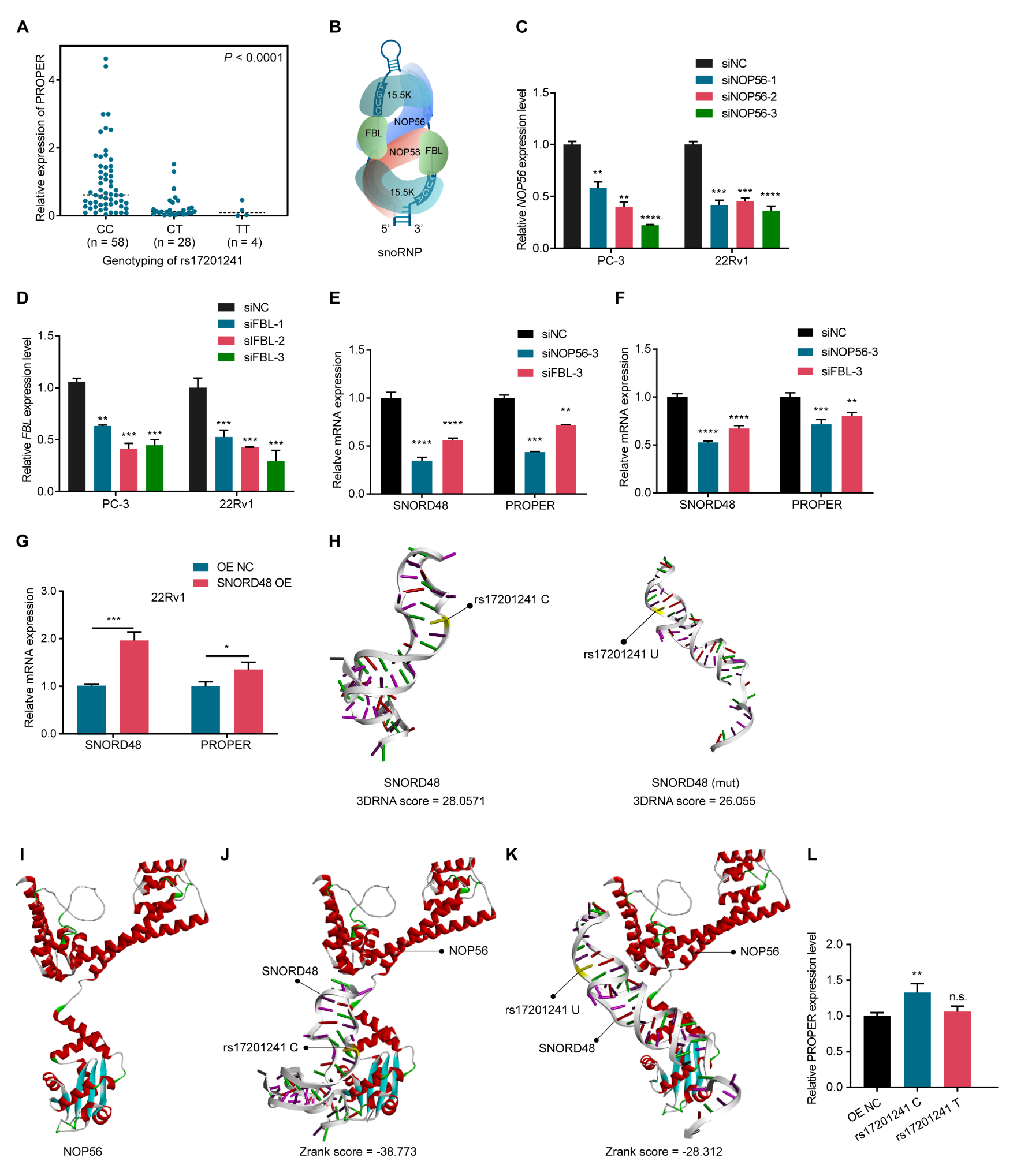


**Supplementary Figure S3. rs17201241 T allele affects SNORD48 mature and PROPER expression**. (A) Expression quantitative trait loci (eQTL) analysis. Linear model *P* values assessed by Matrix eQTL. (B) Fibrillarin, Nop58, Nop56, and 15.8kD proteins associate with C/D box snoRNAs to form a functional snoRNA-ribonucleoprotein complexes (snoRNPs). (C, D) RT-qPCR detection of the knockdown efficiency of NOP56 and FBL by siRNA. (E, F) Knockdown of *FBL* and *NOP56* reduced the expressions of SNORD48 as well as PROPER in PC-3 (E) and 22Rv1 (F) cells detecte by RT-qPCR. (G) Overexpression of SNORD48 significantly increased the expression of PROPER. (H) The 3D structure of SNORD48 that carrying different alleles of rs17201241. (I) The 3D structure of NOP56. (J, K) *In-slico* RNA-protein docking analysis of SNORD48 and NOP56 using ZDOCK tool. (L) The expression level of PROPER in SNORD48 knockdown PC-3 cells transfecting SNORD48 plasmid with different alleles of rs17201241. Data are presented as mean ± s.d. (n = 4); n.s., not significant; ^**^*P* < 0.01 by Mann-Whitney *U*-tests.


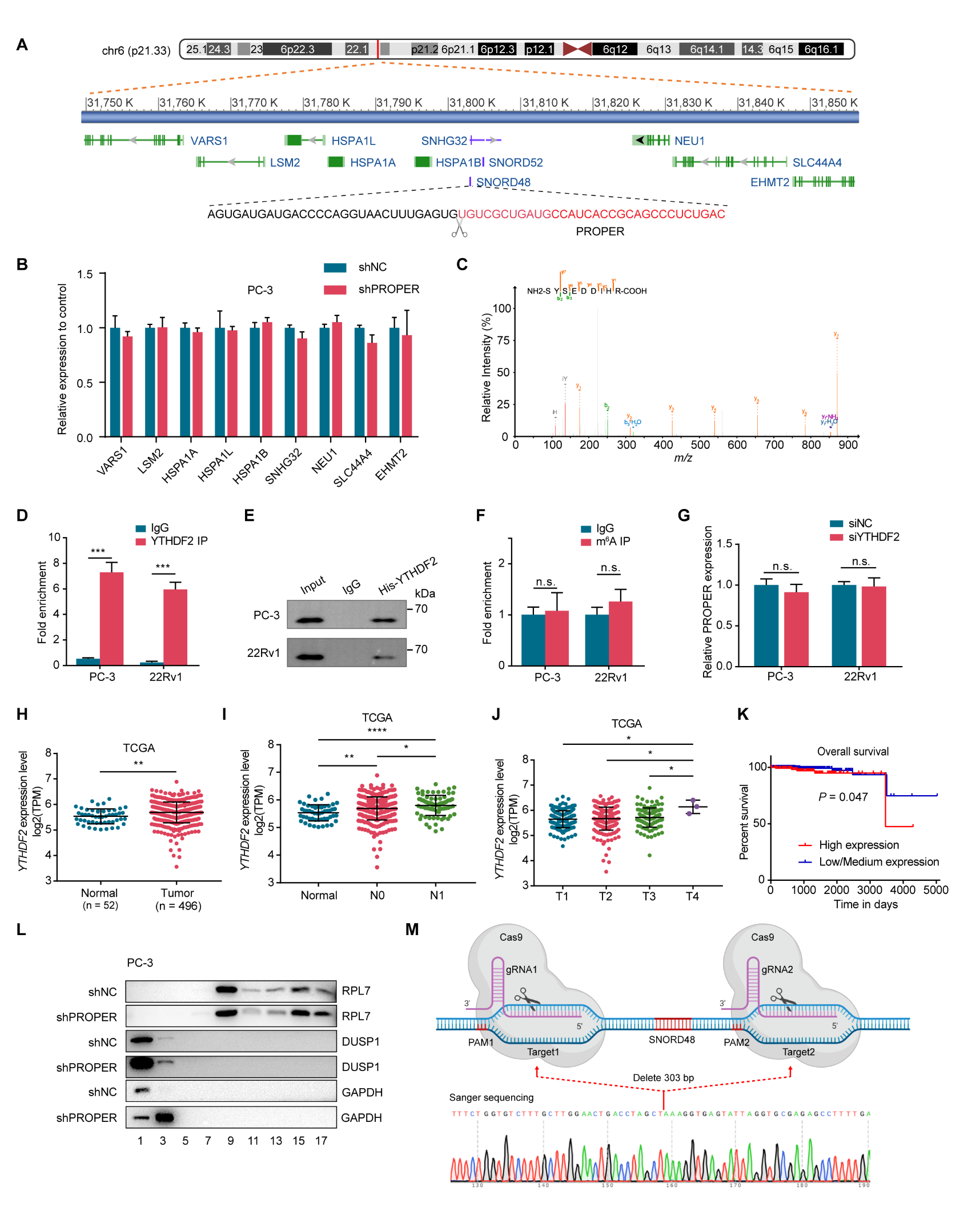


**Supplementary Figure S4. The regulation role of PROPER on repressing mRNA or interacting with proteins.** (A) The genomic physical location of mRNAs within 0.5 M-bases centering the gene generating PROPER. (B) RT-qPCR detection of the effects of PROPER knockdown on the expression of nearby genes. (C) Mass spectrogram of YTHDFs. (D) RIP-qPCR analysis of PROPER immunoprecipitated using an anti-YTHDF2 antibody confirmed the interaction between YTHDF2 and PROPER. The data were normalized to the input levels. Data are presented as mean ± s.d. (n = 4). ^***^*P* < 0.001 by Mann-Whitney *U*-tests. (E) The direct interaction between PROPER and purified His-tagged recombinant YTHDF2 proteins by RNA pull-down assay. (F) RT-qPCR of PROPER after m^6^A methylated RNA immunoprecipitation. n.s., not significant. (G) RT-qPCR of PROPER after YTHDF2 knockdown. n.s., not significant. (H-K) The expression level of *YTHDF2* in different tumor stage and the association with prognosis from TCGA database. ^*^*P* < 0.05, ^**^*P* < 0.01, and ^****^*P* < 0.0001 by two-tailed Student’s *t*-test. (L) Immunoblots of each fraction from polysome profiling in PC-3 as indicated in Figure 3N. RPL7: positive controls for 60S protein. GAPDH: positive control for free protein. (M) The schematic diagram of SNPRD48 knock off by CRISPER/Cas9.


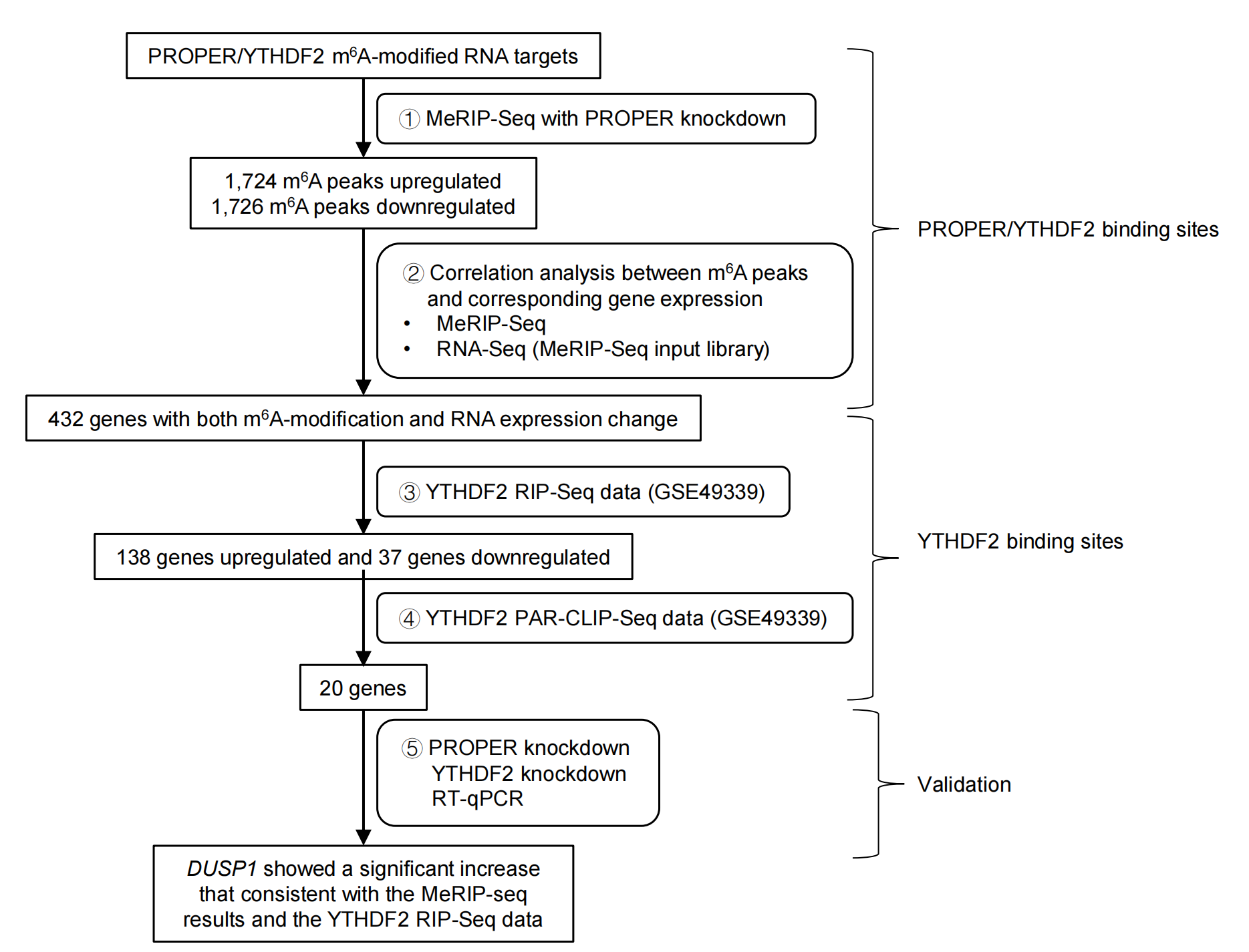


**Supplementary Figure S5.** The screening flowchart for the target genes of PROPER/YTHDF2 complex.


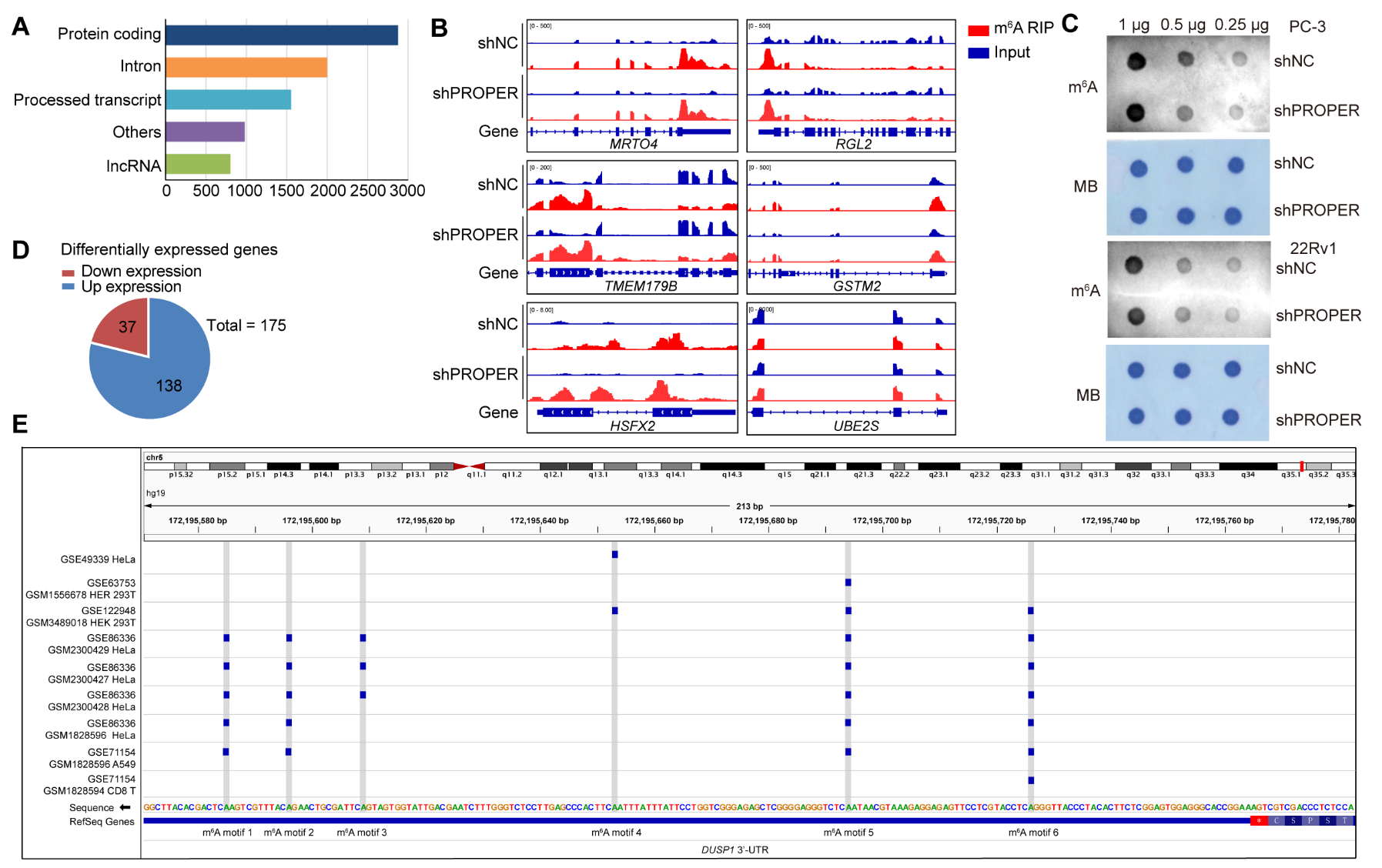


**Supplementary Figure S6. The identification of RNA binding targets of PROPER/YTHDF2 complex.** (A) The number of various RNA species with different m^6^A modifications. (B) MeRIP-Seq displays the distribution of m^6^A peaks and YTHDF2-binding peaks in six representative mRNA of PROPER knockdown and control groups in PC-3 cells. (C) The m^6^A level in PROPER knockdown and control groups of PC-3 and 22Rv1 cells were analyzed by dot blot assay. Methylene blue (MB) staining served as a loading control. (D) The expression changes of 175 target genes of PROPER/YTHDF2 complex identified by m^6^A­­­-Seq, RNA-Seq, and RIP-Seq (data from GSE49339) analysis. (E) CLIP-Seq detected the interaction between YTHDF2 and the 3’-UTR of *DUSP1* mRNA in HeLa cells (data from GSE49339, GSE86336), HER 393T cells (data from GSE63753), HEK 393T cells (data from GSE122948), A549 cells (data from GSE71154), and CD8 T cells (data from GSE71154).


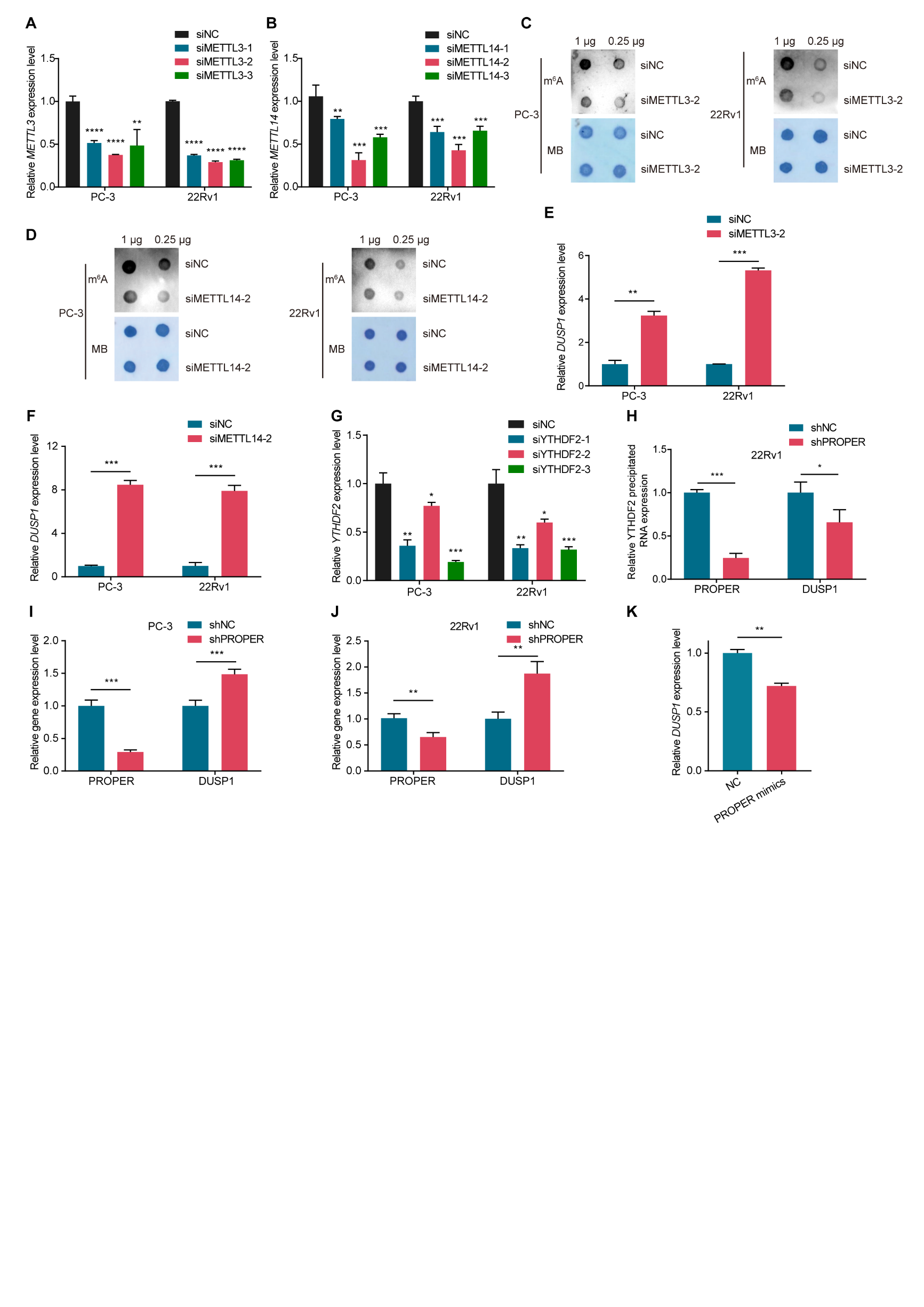


**Supplementary Figure S7. PROPER/YTHDF2 complex mediates *DUSP1* downregulation in an m^6^A dependent manner.** (A, B) RT-qPCR detection of the knockdown efficiency of METTL3 (A) and METTL14 (B) by siRNA. (C, D) The m^6^A level in METTL3 (C) and METTL14 (D) knockdown and control groups of PC-3 and 22Rv1 cells were analyzed by dot blot assay. Methylene blue (MB) staining served as a loading control. (E, F) RT-qPCR detection of the effects of METTL3 (E) or METTL14 (F) knockdown on *DUSP1* expression. (G) RT-qPCR detection of the knockdown efficiency of YTHDF2 by siRNA. (H) RIP-qPCR analysis of PROPER and *DUSP1* immunoprecipitated using an anti-YTHDF2 antibody. (I, J) Effect of PROPER knockdown on *DUSP1* expression in PC-3 (I) and 22Rv1 (J) cells. (K) Effect of PROPER overexpression on endogenous *DUSP1* level. Data are presented as mean ± s.d. (n = 4); ^*^*P* < 0.05, ^**^*P* < 0.01, ^***^*P* < 0.001, ^****^*P* < 0.0001 by Mann-Whitney *U*-tests.


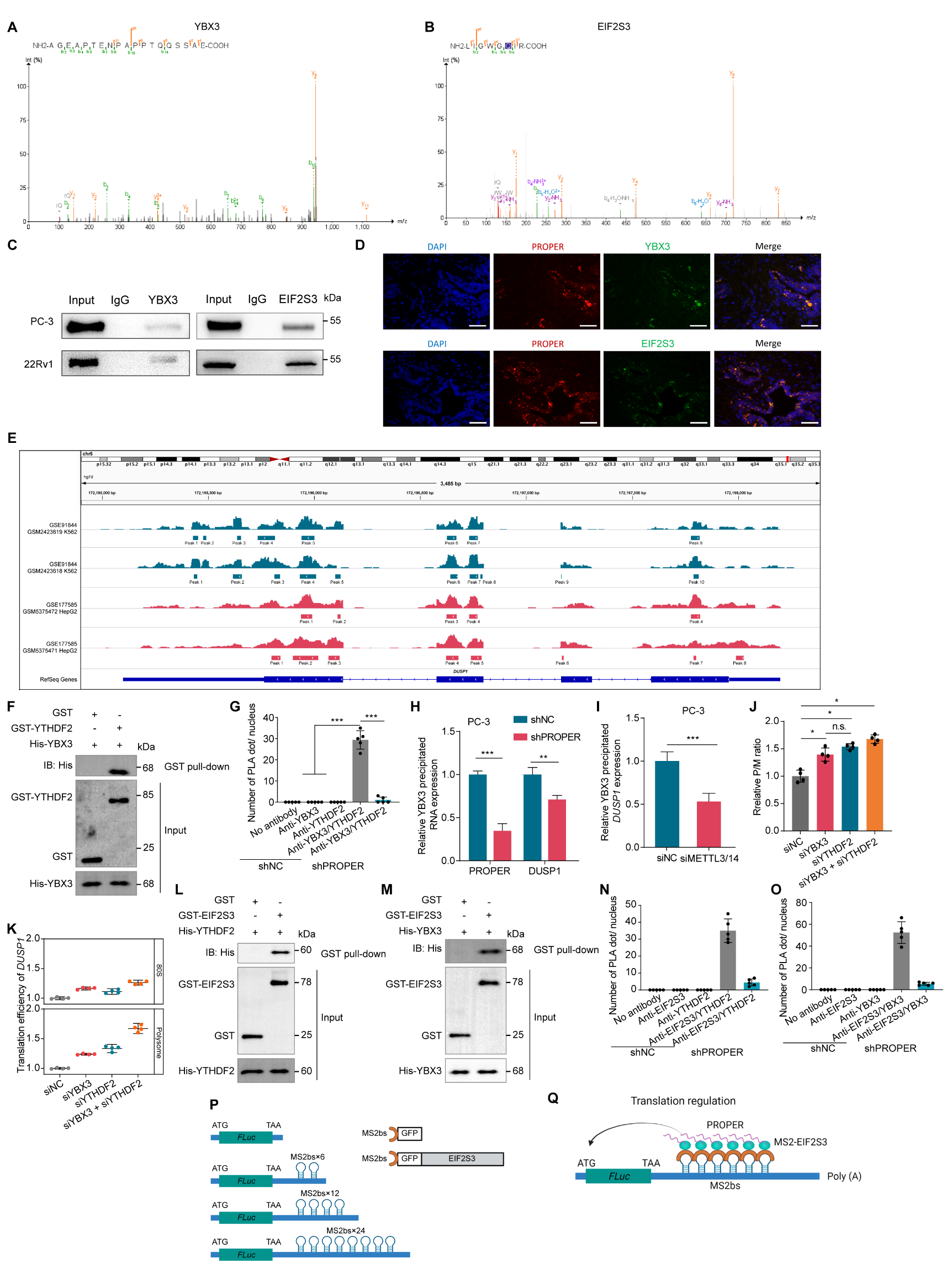


**Supplementary Figure S8. YBX3 and EIF2S3 are key partner of PROPER mediated *DUSP1* decay.** (A, B) Mass spectrogram of YBX3 (A) and EIF2S3 (B). (C) Lysates prepared from PC-3 and 22Rv1 cells were hybridized with biotinylated PROPER probe and subject to RNA pull-down assays. The remaining lysates were subject to western blotting with antibodies against YBX3 and EIF2S3. (D) Prostate tumor sections were stained with DAPI (blue), PROPER (red, by in situ hybridization with Cy3-labed locked nucleic acid probe) and related proteins including YBX3 and EIF2S3 (green, by immunofluorescence). Typical photos showed that expression of PROPER was colocalized with YBX3 (upper) and EIF2S3 (lower). Scale bar, 50 μm. (E) Distributions of YBX3-binding peaks across 3’-UTR of *DUSP1* in K562 cells (data from GSE91844) and HepG2 cells (data from GSE177585). (F) Validation of the interaction between YTHDF2 and YBX3 by western blotting assay. Experiments were performed with GST-purified YTHDF2 protein and His-purified YBX3 protein in PC-3 cells. (G) Number of PLA dot compared to nucleus that reflecting the interaction between YBX3 and YTHDF2 in PC-3 cells. (H) RIP-qPCR analysis of PROPER and *DUSP1* immunoprecipitated using an anti-YBX3 antibody. (I) RIP-qPCR analysis of *DUSP1* immunoprecipitated using an anti-YBX3 antibody with METTL3/14 knockdown. (J, K) YBX3 cooperated with YTHDF2 to mediate translation efficiency of m^6^A modified *DUSP1* (J). We accounted for mRNA level variation by dividing polysome-bound fraction by the non-polysome-bound fraction. Transcript levels are quantified using RT-qPCR (K). (L) Validation of the interaction between EIF2S3 and YTHDF2 by western blotting assay. Experiments were performed with GST-purified EIF2S3 protein and His-purified YTHDF2 protein in PC-3 cells. (M) Validation of the interaction between EIF2S3 and YBX3 by western blotting assay. Experiments were performed with GST-purified EIF2S3 protein and His-purified YBX3 protein in PC-3 cells. (N) Number of PLA dot compared to nucleus that reflecting the interaction between EIF2S3 and YTHDF2 in PC-3 cells. (O) Number of PLA dot compared to nucleus that reflecting the interaction between EIF2S3 and YBX3 in PC-3 cells. (P, Q) Schematic diagram of plasmid construction in MS2 tethering assays (P) and MS2 tethering reporter assay for PROPER in regulating *DUSP1* mRNA circularization (Q). Data are presented as mean ± s.d. (n = 4); n.s., not significant; ^*^*P* < 0.05, ^**^*P* < 0.01, ^***^*P* < 0.001, ^****^*P* < 0.0001 by Mann-Whitney *U*-tests.


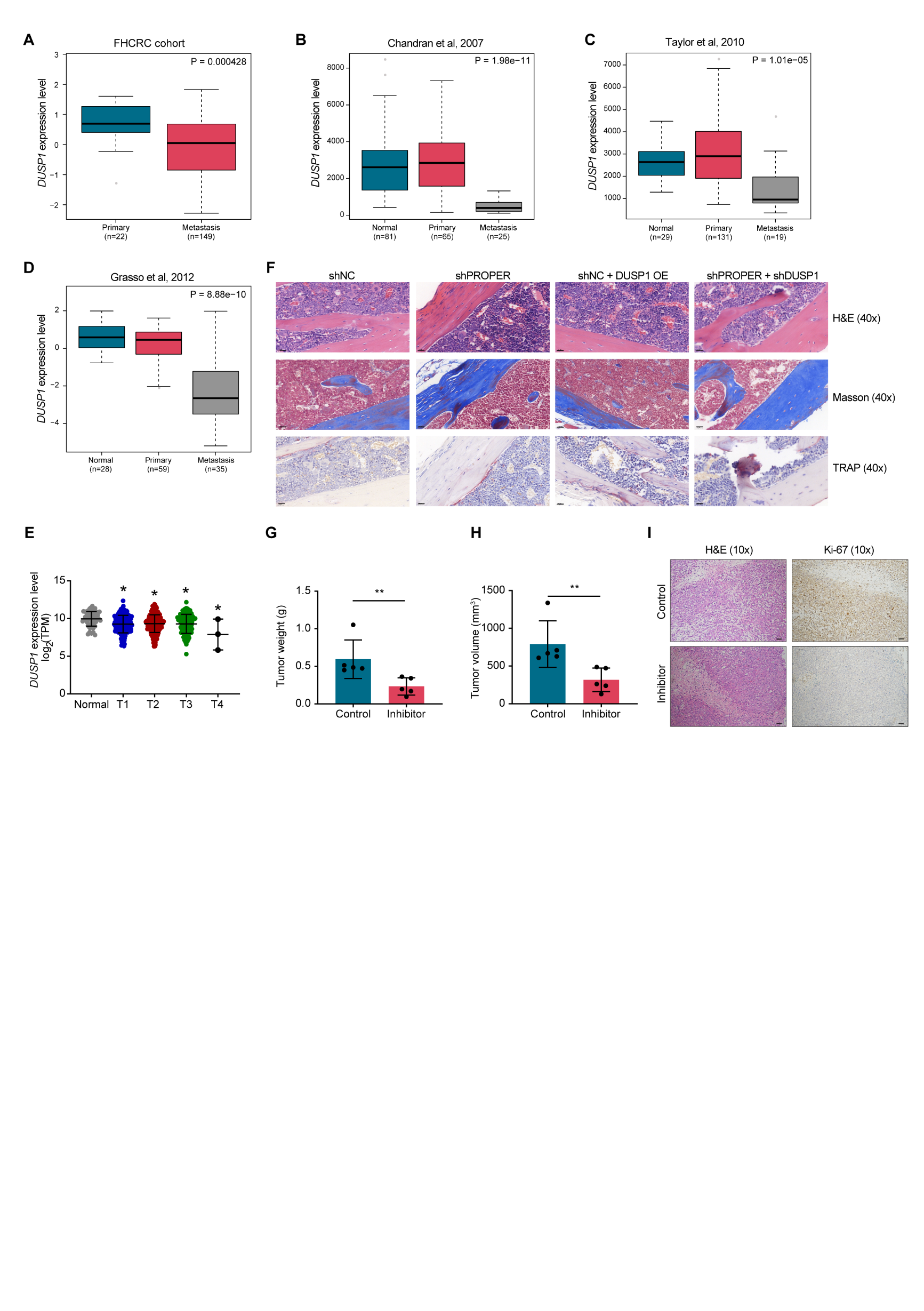


**Supplementary Figure S9. Examination of the role of DUSP1 in prostate cancer development.** (A-D) Expression of *DUSP1* was compared between benign, primary, and metastatic samples in FHCRC (A), Chandran (B), Taylor (C) and Grasso (D) patient cohorts. *P* values were calculated using Student’s *t* test (A) and one-way ANOVA (B-D), respectively. (E) The expression level of *DUSP1* in different tumor stage from TCGA database. ^**^*P* < 0.01 by Student’s *t* test. (F) Representative H&E (up) Masson (middle), and TRAP (down)-stained bone metastases tissues produced by the growth of PC-3 human prostate cancer cells in the tibia of a nude mouse. (G, H) The tumor weight (G) and volume (H) of tumors with or without injection of antagoPROPER. ^**^*P* < 0.01 by Mann-Whitney *U*-tests. (I) H&E and Ki-67 immunostaining of tumor tissue from nude mice xenografts.

**Supplementary Table S1. Summary results of meta-analysis and replication studies for PCa**

| **SNP** | **Allele^a^** | **Chr** | **Position** | **Gene** | **Study** | **Population** | **MAF** | **OR (95% CI)** | ***P* value** | ***P*_het_^b^** |
| --- | --- | --- | --- | --- | --- | --- | --- | --- | --- | --- |
| rs17201241 | T/C | 6 | 31803074 | *SNORD48* | Chinese GWAS | Chinese | 0.067 | 0.70 (0.54-0.91) | 6.54E-04 |  |
|  |  |  |  |  | Japanese GWAS | Japanese | 0.072 | 0.95 (0.88-1.03) | 2.36E-01 |  |
|  |  |  |  |  | PRACTICAL Meta-analysis | European or Asian | 0.064 | 0.94 (0.91-0.98) | 4.89E-04 |  |
|  |  |  |  |  | Meta-analysis of all studies | |  | 0.94 (0.91-0.97) | **7.33E-05** | 0.072 |
| rs7911488 | G/A | 10 | 105154089 | *ATP5MD*, *MIR1307* | Chinese GWAS | Chinese | 0.335 | 0.95 (0.81-1.13) | 5.64E-01 |  |
|  |  |  |  |  | Japanese GWAS | Japanese | 0.360 | 1.00 (0.96-1.04) | 8.93E-01 |  |
|  |  |  |  |  | PRACTICAL Meta-analysis | European or Asian | 0.336 | 1.01 (1.00-1.03) | 8.15E-02 |  |
|  |  |  |  |  | Meta-analysis of all studies | |  | 0.99 (0.97-1.00) | 1.32E-01 | 0.574 |
| rs1059292 | C/T | 11 | 62623051 | *SNHG1*, *SNORD25* | Chinese GWAS | Chinese | 0.141 | 0.90 (0.74-1.08) | 2.52E-01 |  |
|  |  |  |  |  | Japanese GWAS | Japanese | 0.148 | 1.01 (0.96-1.07) | 6.80E-01 |  |
|  |  |  |  |  | PRACTICAL Meta-analysis | European or Asian | 0.052 | 1.00 (0.97-1.04) | 8.71E-01 |  |
|  |  |  |  |  | Meta-analysis of all studies | |  | 1.00 (0.97-1.03) | 8.61E-01 | 0.477 |
| rs78212770 | G/C | 15 | 70371777 | *MIR629*, *TLE3* | Chinese GWAS | Chinese | 0.089 | 0.92 (0.78-1.10) | 3.69E-01 |  |
|  |  |  |  |  | Japanese GWAS | Japanese | 0.184 | 0.99 (0.94-1.05) | 7.35E-01 |  |
|  |  |  |  |  | PRACTICAL Meta-analysis | European or Asian | 0.021 | 1.04 (0.97-1.12) | 2.56E-01 |  |
|  |  |  |  |  | Meta-analysis of all studies | |  | 1.00 (0.95-1.04) | 8.46E-01 | 0.338 |
| rs2102066 | G/A | 16 | 69967093 | *WWP2* | Chinese GWAS | Chinese | 0.030 | 0.83 (0.59-1.18) | 3.03E-01 |  |
|  |  |  |  |  | Japanese GWAS | Japanese | 0.040 | 1.06 (0.95-1.17) | 3.01E-01 |  |
|  |  |  |  |  | PRACTICAL Meta-analysis | European or Asian | 0.265 | 1.01 (0.99-1.03) | 3.95E-01 |  |
|  |  |  |  |  | Meta-analysis of all studies | |  | 0.99 (0.97-1.01) | 3.37E-01 | 0.381 |

MAF, minor allele frequency; GWAS, genome-wide association study; OR, odds ratio; CI, confidence interval; SNP, single-nucleotide polymorphism.

^a^ Effect/non-effect allele.

^b^ *P* value for the heterogeneity.

**Supplementary Table S2. Characteristics of the subjects participated in this study from the ChinaPCa GWAS study**

| **Variables** | **Cases (n = 1151)** | **Controls (n = 1202)** | ***P*** |
| --- | --- | --- | --- |
| Age (years, Mean ± SD) | 71.17 ± 8.22 | 70.69 ± 10.46 | 0.218 |
| PSA^a^ (ng/mL, Mean ± SD) | 160.55 ± 486.55 | 1.11 ± 1.46 | < 0.001 |
| Gleason score |  |  |  |
| <7 | 320 (27.80%) |  |  |
| ≥7 | 831 (72.20%) |  |  |
| T-stage |  |  |  |
| T1 | 154 (14.67%) |  |  |
| T2 | 472 (44.95%) |  |  |
| T3 | 308 (29.33%) |  |  |
| T4 | 116 (11.05%) |  |  |
| TX | 101 |  |  |
| N-stage |  |  |  |
| N0 | 669 (67.51%) |  |  |
| N1 | 322 (32.49%) |  |  |
| NX | 160 |  |  |
| M-stage |  |  |  |
| M0 | 717 (65.72%) |  |  |
| M1 | 374 (34.28%) |  |  |
| MX | 60 |  |  |
| Clinical stage^b^ |  |  |  |
| Aggressive | 854 (74.20%) |  |  |
| Non-aggressive | 297 (25.80%) |  |  |

^a^ Diagnostic PSA level, ng/mL.

^b^ Aggressive disease was defined as PSA > 100 ng/mL, or Gleason score ≥8, or tumor stage T3/T4, or tumor in nodes (N1), or metastasis present (M1), or prostate cancer-associated death.

**Supplementary Table S3. Stratification analyses for the association between rs17201241 variant and prostate cancer risk in dominant model**

| **Variables** | **CC (N, %)** | **CT/TT (N, %)** | **OR (95% CI)** | ***P* value** | ***I*^2^** | ***P*_het_^a^** |
| --- | --- | --- | --- | --- | --- | --- |
| Controls | 1,018 (50.4) | 166 (57.6) | 1.00 |  |  |  |
| Cases | 1,000 (49.6) | 122 (42.4) | 0.70 (0.54-0.91) | 6.54E-03 |  |  |
| PSA^b^ (%) |  |  |  |  | 0% | 0.97 |
| ≤100 | 800 (80.0) | 97 (79.5) | 0.71 (0.54-0.93) | 1.37E-02 |  |  |
| ＞100 | 200 (20.0) | 25 (20.5) | 0.71 (0.45-1.13) | 1.48E-01 |  |  |
| Gleason score (%) | |  |  |  | 0% | 0.73 |
| ＜8 | 622 (62.2) | 79 (64.8) | 0.73 (0.55-0.98) | 3.79E-02 |  |  |
| ≥8 | 378 (37.8) | 43 (35.2) | 0.67 (0.47-0.97) | 3.37E-02 |  |  |
| T stage (%) | |  |  |  | 0% | 0.37 |
| T1&T2 | 545 (59.5) | 67 (63.2) | 0.73 (0.54-1.00) | 5.12E-02 |  |  |
| T3&T4 | 371 (40.5) | 39 (36.8) | 0.59 (0.40-0.86) | 5.56E-03 |  |  |
| N stage (%) | |  |  |  | 34% | 0.22 |
| N0 | 592 (68.3) | 61 (61.6) | 0.60 (0.44-0.82) | 1.56E-03 |  |  |
| N1 | 275 (31.7) | 38 (38.4) | 0.82 (0.56-1.21) | 3.11E-01 |  |  |
| M stage (%) | |  |  |  | 60% | 0.11 |
| M0 | 634 (90.7) | 65 (56.5) | 0.60 (0.44-0.82) | 1.46E-03 |  |  |
| M1 | 314 (9.3) | 50 (43.5) | 0.88 (0.62-1.26) | 4.86E-01 |  |  |
| Clinical stage^c^ (%) | |  |  |  | 0% | 0.97 |
| Non-aggressive | 261 (26.1) | 32 (26.2) | 0.70 (0.46-1.06) | 8.81E-02 |  |  |
| Aggressive | 739 (73.9) | 90 (73.8) | 0.70 (0.53-0.93) | 1.46E-02 |  |  |

OR, odds ratio; CI, confidence interval.

*P* < 0.05 considered to be significant.

^a^ *P* value for the heterogeneity.

^b^ Diagnostic PSA level, ng/mL.

^c^ Aggressive disease was defined as PSA > 100 ng/mL, or Gleason score ≥8, or tumor stage T3/T4, or tumor in nodes (N1), or metastasis present (M1), or prostate cancer-associated death.

**Supplementary Table S4. Small RNA-seq analysis of transcriptome in PC-3 and 22Rv1 cells harboring the SNP rs17201241**

| **Cell line** | **Reads (5'-3')** |
| --- | --- |
| PC-3 | TGTCGCTGATGCCATCACCGCAGCGCTCTGACC |
| PC-3 | TGTCGCTGATGCCATCACCGCAGCGCTCTGACT |
| PC-3 | TGTCGCTGATGCCATCACCGCAGCGCTCTGACG |
| PC-3 | GTGTCGCTGATGCCATCACCGCAGCGCTCTGACC |
| PC-3 | GTAACTCTTGAGTGTGTCGCTGATGCCATCACCGCAGCGCTCTGACC |
| PC-3 | CTCTTGAGTGTGTCGCTGATGCCATCACCGCAGCGCTCTGACC |
| PC-3 | TGTCGCTGATGCCATCACCGCAGCGCTCTGACA |
| PC-3 | GTGTCGCTGATGCCATCACCGCAGCGCTCTGACT |
| PC-3 | GTAACTCTGAGTGTGTCGCTGATGCCATCACCGCAGCGCTCTGACC |
| PC-3 | GTCGCTGATGCCATCACCGCAGCGCTCTGACG |
| PC-3 | GTGTGTCGCTGATGCCATCACCGCAGCGCTCTGACG |
| PC-3 | CGCTGATGCCATCACCGCAGCGCTCTGACT |
| PC-3 | ACTCTTGAGTGTGTCGCTGATGCCATCACCGCAGCGCTCTGACG |
| PC-3 | TAACTCTGAGTGTGTCGCTGATGCCATCACCGCAGCGCTCTGACG |
| PC-3 | GTGTGTCGCTGATGCCATCACCGCAGCGCTCTGACC |
| PC-3 | GTGTCGCTGATGCCATCACCGCAGCGCTCTGACG |
| PC-3 | AGTCGCTGATGCCATCACCGCAGCGCTCTGACT |
| PC-3 | TGTCGCTGATGCCATCACCGCAGCGCTCTGAC |
| PC-3 | GTGTGTCGCTGATGCCATCACCGCAGCGCTCTGACT |
| PC-3 | AACTCTAGAGTGTGTCGCTGATGCCATCACCGCAGCGCTCTGACC |
| PC-3 | TATCGCTGATGCCATCACCGCAGCGCTCTGACT |
| PC-3 | GAGTGTGTCGCTGATGCCATCACCGCAGCGCTCTGACT |
| PC-3 | TCTGAGTGTGTCGCTGATGCCATCACCGCAGCGCTCTGACC |
| PC-3 | GGTAACTCTGAGTGTGTCGCTGATGCCATCACCGCAGCGCTCTGACT |
| PC-3 | CGCTGATGCCATCACCGCAGCGCTCTGACA |
| PC-3 | AACTCTGAGTGTGTCGCTGATGCCATCACCGCAGCGCTCTGACC |
| PC-3 | GTAACTCTGAGTGTGTCGCTGATGCCATCACCGCAGCGCTCTGATT |
| PC-3 | GTGTCGCTGATGCCATCACCGCAGCGCTCTGAGACC |
| PC-3 | GTGACGCTGATGCCATCACCGCAGCGCTCTGACG |
| PC-3 | GTGTCGCTGATGCCATCACCGCAGCGCTCTGACA |
| PC-3 | GTAACTCTGAGTGTGTCGCTGATGCCATCACCGCAGCGCTCTGACCGC |
| PC-3 | AACTCTTGAGTGTGTCGCTGATGCCATCACCGCAGCGCTCTGACC |
| PC-3 | TGCCGCTGATGCCATCACCGCAGCGCTCTGACT |
| PC-3 | CTCTCGAGTGTGTCGCTGATGCCATCACCGCAGCGCTCTGACT |
| PC-3 | GTGTGTCGCTGATGCCATCACCGCAGCGCTCTGACCT |
| PC-3 | TCTTGAGTGTGTCGCTGATGCCATCACCGCAGCGCTCTGACT |
| PC-3 | TGAGTGTGTCGCTGATGCCATCACCGCAGCGCTCTGACT |
| PC-3 | GAGTGTGTCGCTGATGCCATCACCGCAGCGCTCTGACG |
| PC-3 | CTCTTGAGTGTGTCGCTGATGCCATCACCGCAGCGCTCTGACT |
| PC-3 | AACTCTTGAGTGTGTCGCTGATGCCATCACCGCAGCGCTCTGACT |
| PC-3 | GTGTCGCTGATGCCATCACCGCAGCGCTCTGACCGT |
| PC-3 | TGAGTGTGTCGCTGATGCCATCACCGCAGCGCTCTGACC |
| PC-3 | TTGAGTGTGTCGCTGATGCCATCACCGCAGCGCTCTGACT |
| PC-3 | TCTTGAGTGTGTCGCTGATGCCATCACCGCAGCGCTCTGACA |
| PC-3 | **GTCGCTGATGCCATCACCGCAGCGCTCTGACC** |
| PC-3 | GTGTCGCTGATGCCATCACCGCAGCGCTCTGACCGC |
| PC-3 | ACTCTTGAGTGTGTCGCTGATGCCATCACCGCAGCGCTCTGACT |
| PC-3 | GTAACTCTGAGTGTGTCGCTGATGCCATCACCGCAGCGCTCTGACT |
| 22Rv1 | TGTCGCTGATGCCATCACCGCAGCGCTCTGACT |
| 22Rv1 | TGTCGCTGATGCCATCACCGCAGCGCTCTGACC |
| 22Rv1 | TGTCGCTGATGCCATCACCGCAGCGCTCTGACA |
| 22Rv1 | GTAACTCTGAGTGTGTCGCTGATGCCATCACCGCAGCGCTCTGACC |
| 22Rv1 | GTAACTCTGAGTGTGTCGCTGATGCCATCACCGCAGCGCTCTGACT |
| 22Rv1 | GGTAACTCTGAGTGTGTCGCTGATGCCATCACCGCAGCGCTCTGACC |
| 22Rv1 | GGTAACTCTGAGTGTGTCGCTGATGCCATCACCGCAGCGCTCTGACT |
| 22Rv1 | TAACTCTGAGTGTGTCGCTGATGCCATCACCGCAGCGCTCTGACC |
| 22Rv1 | TAACTCTGAGTGTGTCGCTGATGCCATCACCGCAGCGCTCTGACT |
| 22Rv1 | AACTCTGAGTGTGTCGCTGATGCCATCACCGCAGCGCTCTGACC |
| 22Rv1 | **GTCGCTGATGCCATCACCGCAGCGCTCTGACC** |
| 22Rv1 | AACTCTGAGTGTGTCGCTGATGCCATCACCGCAGCGCTCTGACT |
| 22Rv1 | GTCGCTGATGCCATCACCGCAGCGCTCTGACT |
| 22Rv1 | GTAACTCTGAGTGTGTCGCTGATGCCATCACCGCAGCGCTCTGACA |
| 22Rv1 | AACTCTGAGTGTGTCGCTGATGCCATCACCGCAGCGCTCTGACA |
| 22Rv1 | GGTAACTCTGAGTGTGTCGCTGATGCCATCACCGCAGCGCTCTGACA |
| 22Rv1 | GTGTCGCTGATGCCATCACCGCAGCGCTCTGACT |
| 22Rv1 | GTCGCTGATGCCATCACCGCAGCGCTCTGACA |
| 22Rv1 | AGTCGCTGATGCCATCACCGCAGCGCTCTGACT |
| 22Rv1 | GTGTGTCGCTGATGCCATCACCGCAGCGCTCTGACT |
| 22Rv1 | CGTCGCTGATGCCATCACCGCAGCGCTCTGACC |
| 22Rv1 | AACTCTGAGAGTGTCGCTGATGCCATCACCGCAGCGCTCTGACT |
| 22Rv1 | TGTCGCTGATGCCATCACCGCAGCGCTCTGATT |
| 22Rv1 | GTGTCGCTGATGCCATCACCGCAGCGCTCTGACA |
| 22Rv1 | GTAACTCTGAGTGTGTCGCTGATGCCATCACCGCAGCGCTCTGACCG |
| 22Rv1 | GAGTGTGTCGCTGATGCCATCACCGCAGCGCTCTGACC |
| 22Rv1 | CGTCGCTGATGCCATCACCGCAGCGCTCTGACT |
| 22Rv1 | GTGTCGCTGATGCCATCACCGCAGCGCTCTGACC |
| 22Rv1 | CTCTGAGTGTGTCGCTGATGCCATCACCGCAGCGCTCTGACC |
| 22Rv1 | ACTCTGAGTGTGTCGCTGATGCCATCACCGCAGCGCTCTGACT |
| 22Rv1 | GTCGCTGATGCCATCACCGCAGCGCTCTGACG |
| 22Rv1 | GTAACTCTGAATGTGTCGCTGATGCCATCACCGCAGCGCTCTGACT |
| 22Rv1 | CTCTGAGTGTGTCGCTGATGCCATCACCGCAGCGCTCTGACT |
| 22Rv1 | TGTCGCTGATGCCATCACCGCAGCGCTCTGACG |
| 22Rv1 | AAACTCTGAGTGTGTCGCTGATGCCATCACCGCAGCGCTCTGACC |
| 22Rv1 | AACTCTGAGTGCGTCGCTGATGCCATCACCGCAGCGCTCTGACT |
| 22Rv1 | GACTCTGAGTGTGTCGCTGATGCCATCACCGCAGCGCTCTGACA |
| 22Rv1 | GAGTGTGTCGCTGATGCCATCACCGCAGCGCTCTGACT |
| 22Rv1 | AACTCAGAGTGTGTCGCTGATGCCATCACCGCAGCGCTCTGACC |
| 22Rv1 | GTAACTCTGAGTGTGTCGCTGATGCCATCACCGCAGCGCTCTGATT |
| 22Rv1 | GTAACTCTGAGTGCGTCGCTGATGCCATCACCGCAGCGCTCTGACT |
| 22Rv1 | GTAACCCTGAGTGTGTCGCTGATGCCATCACCGCAGCGCTCTGACC |
| 22Rv1 | GGTAATTCTGAGTGTGTCGCTGATGCCATCACCGCAGCGCTCTGACC |
| 22Rv1 | GTCGCTGATGCCATCACCGCAGCGCTCTGATT |
| 22Rv1 | AACTCAGAGTGTGTCGCTGATGCCATCACCGCAGCGCTCTGACT |
| 22Rv1 | GAGTGTGTCGCTGATGCCATCACCGCAGCGCTCTGACA |
| 22Rv1 | CCAGGTAACTCTGAGTGTGTCGCTGATGCCATCACCGCAGCGCTCTGACC |
| 22Rv1 | AACTCTGAGAGTGTCGCTGATGCCATCACCGCAGCGCTCTGACA |
| 22Rv1 | GCAACTCTGAGTGTGTCGCTGATGCCATCACCGCAGCGCTCTGACA |
| 22Rv1 | TGTAACTCTGAGTGTGTCGCTGATGCCATCACCGCAGCGCTCTGACT |
| 22Rv1 | GTAACTCTGAGTGCGTCGCTGATGCCATCACCGCAGCGCTCTGACC |
| 22Rv1 | AACTCTGAGTGTGTCGCTGATGCCATCACCGCAGCGCTCTGACCAAATCG |
| 22Rv1 | GTAACTCTGAGAGTGTCGCTGATGCCATCACCGCAGCGCTCTGACC |
| 22Rv1 | TGTCGCTGATGCCATCACCGCAGCGCTCTGATC |
| 22Rv1 | AACTCTGAGTGTGTCGCTGATGCCATCACCGCAGCGCTCTGACAAGGTCG |
| 22Rv1 | GGTAACTCTGAATGTGTCGCTGATGCCATCACCGCAGCGCTCTGAC |
| 22Rv1 | TGCCGCTGATGCCATCACCGCAGCGCTCTGACC |
| 22Rv1 | TCTCGCTGATGCCATCACCGCAGCGCTCTGACT |
| 22Rv1 | ATGTAACTCTGAGTGTGTCGCTGATGCCATCACCGCAGCGCTCTGACT |
| 22Rv1 | GGTAACTCTGAGTGTGTCGCTGATGCCATCACCGCAGCGCTCTGATT |
| 22Rv1 | GTCGCTGATGCCATCACCGCAGCGCTCTGAAC |
| 22Rv1 | GTAACTCTGAGTGAGTCGCTGATGCCATCACCGCAGCGCTCTGACC |
| 22Rv1 | TCGCTGATGCCATCACCGCAGCGCTCTGACT |
| 22Rv1 | GGAAACTCTGAGTGTGTCGCTGATGCCATCACCGCAGCGCTCTGACT |

**Supplementary Table S5. Characteristics of the subjects from tissue microarray**

| **Variables** | **Cases (n = 90)** |
| --- | --- |
| Age (years, Mean ± SD) | 69.85 ± 7.04 |
| ISUP grade group |  |
| 2 | 15 (17.78%) |
| 3 | 24 (26.67%) |
| 4 | 14 (13.33%) |
| 5 | 37 (42.22%) |
| T-stage |  |
| T2 | 16 (17.78%) |
| T3 | 65 (72.22%) |
| T4 | 9 (10.00%) |
| N-stage |  |
| N0 | 28 (31.11%) |
| N1 | 23 (25.56%) |
| NX | 18 (20.00%) |
| Missing | 21 (23.33%) |
| Clinical stage^a^ |  |
| Aggressive | 77 (85.56%) |
| Non-aggressive | 13 (14.44%) |

ISUP, International Society of Urological Pathology

^a^ Aggressive: ISUP grade group 3 or higher, a stage of T3/T4, N+, or M+.

**Supplementary Table S6. List of the same biotinylated-PROPER pull-down proteins identified by mass spectrometry both in PC-3 and 22Rv1 cells and function prediction of proteins**

|  | **Probe enriched unique peptides** | | | |  |  |  |  |  |
| --- | --- | --- | --- | --- | --- | --- | --- | --- | --- |
| **Gene name** | **PC-3 sense probe** | **PC-3 antisense probe** | **22Rv1 sense probe** | **22Rv1 antisense probe** |  | **Accession** | **Subcellular locations from COMPARTMENTS** | **Gene Ontology (GO) - Molecular Function** | **Gene Ontology (GO) - Biological Process** |
| EIF2S3 | 3 | 0 | 3 | 0 |  | Q2VIR3 | cytosol, extracellular | 1.tRNA binding  2.nucleotide binding  3.translation initiation factor activity  4.GTPase activity  5.protein binding | 1.formation of translation preinitiation complex  2.translation  3.translational initiation  4.positive regulation of translational fidelity |
| PCBP2 | 3 | 0 | 3 | 0 |  | Q15366 | cytosol, nucleus | 1.nucleic acid binding  2.DNA binding  3.single-stranded DNA binding  4.RNA binding  5.mRNA binding | 1.regulation of gene expression  2.mRNA metabolic process  3.viral RNA genome replication  4.proteasome-mediated ubiquitin-dependent protein catabolic process |
| KRT34 | 3 | 0 | 3 | 0 |  | O76011 | cytosol, cytoskeleton | / | 1.epidermis development  2.epithelial cell differentiation  3.intermediate filament organization |
| HNRNPLL | 2 | 0 | 3 | 0 |  | Q8WVV9 | nucleus, cytosol | 1.nucleic acid binding  2.RNA binding  3.mRNA binding  4.protein binding | 1.mRNA processing  2.positive regulation of RNA splicing  3.regulation of RNA splicing |
| PUF60 | 2 | 0 | 3 | 0 |  | Q9UHX1 | nucleus | 1.nucleic acid binding  2.DNA binding  3.RNA binding  4.protein binding  5.identical protein binding | 1.alternative mRNA splicing, via spliceosome  2.regulation of alternative mRNA splicing, via spliceosome  3.mRNA splice site selection  4.mRNA processing  5.apoptotic process |
| RPL13A | 2 | 0 | 2 | 0 |  | Q6NVV1 | cytosol, nucleus | 1.RNA binding 2.mRNA binding 3.structural constituent of ribosome | 1.cytoplasmic translation 2.translation 3.regulation of translation 4.negative regulation of translation 5.cellular response to interferon-gamma |
| YBX3 | 2 | 0 | 2 | 0 |  | P16989 | cytosol, nucleus | 1.RNA polymerase II transcription regulatory  2.region sequence-specific DNA binding 3.nucleic acid binding 4.DNA binding 5.RNA binding 6.mRNA 3'-UTR binding | 1.negative regulation of transcription by RNA polymerase II 2.in utero embryonic development 3.spermatogenesis 4.male gonad development 5.fertilization |
| DEK | 2 | 0 | 2 | 0 |  | P35659 | nucleus, cytosol | 1.DNA binding 2.RNA binding 3.protein binding 4.histone binding | 1.chromatin organization 2.regulation of transcription by RNA polymerase II 3.transcription by RNA polymerase II 4.signal transduction 5.viral genome replication |
| RBM45 | 2 | 0 | 2 | 0 |  | Q8IUH3 | nucleus | 1.nucleic acid binding 2.RNA binding 3.protein binding 4.identical protein binding | 1.ervous system development 2.cell differentiation |
| U17LJ | 2 | 0 | 2 | 0 |  | D6RCP7| | cytosol, endoplasmic reticulum, nucleus | 1.cysteine-type endopeptidase activity 2.thiol-dependent deubiquitinase 3.peptidase activity 4.cysteine-type peptidase activity 5.hydrolase activity | 1.proteolysis 2.ubiquitin-dependent protein catabolic process 3.protein deubiquitination 4.regulation of apoptotic process |
| AP2M1 | 2 | 0 | 2 | 0 |  | Q96CW1 | cytosol, plasma membrane, lysosome, endosome, extracellular | 1.signal sequence binding 2.protein binding 3.lipid binding 4.clathrin adaptor activity 5.transmembrane transporter binding | 1.positive regulation of receptor internalization 2.intracellular protein transport 3.endocytosis 4.vesicle budding from membrane |
| H2B1J | 2 | 0 | 2 | 0 |  | P06899 | nucleus, extracellular | 1.lipopolysaccharide binding 2.DNA binding 3.protein binding 4.protein heterodimerization activity | 1.innate immune response in mucosa 2.nucleosome assembly 3.negative regulation of tumor necrosis factor-mediated signaling pathway 4.antibacterial humoral response 5.killing of cells of another organism |
| SRSF8 | 2 | 0 | 2 | 0 |  | Q9BRL6 | cytosol, nucleus | 1.nucleic acid binding 2.transcription corepressor activity 3.RNA binding 4.protein kinase C binding 5.protein binding | 1.mitotic cell cycle 2.regulation of alternative mRNA splicing, via spliceosome 3.mRNA splicing, via spliceosome 4.mRNA processing 5.RNA splicing |
| YTHDF2 | 2 | 0 | 2 | 0 |  | Q9Y5A9 | cytosol, nucleus, cytoskeleton | 1.RNA binding 2.mRNA binding 3.protein binding 4.C5-methylcytidine-containing RNA binding 5.N6-methyladenosine-containing RNA binding | 1.oocyte maturation 2.immune system process 3.mRNA catabolic process 4.humoral immune response 5.cell cycle |

**Supplementary Table S7. List of differentially m^6^A methylated RNA peaks in PROPER knockdown compared to NC PC-3 cells. (Fold change > 1.5)**

| **Chromosome** | **Peak start** | **Peak end** | **Width** | **Gene ID** | **Transcript type** | **Gene name** | **Peak annotation** | ***P* value** | **m^6^A regulation** |
| --- | --- | --- | --- | --- | --- | --- | --- | --- | --- |
| chr8 | 116851013 | 116851104 | 92 | ENSG00000164754 | protein_coding | RAD21 | 5' UTR | 0.05 | down |
| chr6 | 111266902 | 111267413 | 512 | ENSG00000173214 | processed_transcript | MFSD4B | 3' UTR | 0.05 | down |
| chr22 | 18992429 | 18992819 | 391 | ENSG00000273032 | lncRNA | DGCR5 | Exon | 0.05 | down |
| chr17 | 7709287 | 7709437 | 151 | ENSG00000108947 | protein_coding | EFNB3 | Exon | 0.05 | down |
| chr15 | 40196592 | 40200042 | 3451 | ENSG00000156970 | retained_intron | BUB1B | 3' UTR | 0.05 | down |
| chr1 | 203800197 | 203800528 | 332 | ENSG00000058673 | nonsense_mediated_decay | ZC3H11A | 3' UTR | 0.05 | down |
| chr11 | 1242667 | 1243088 | 422 | ENSG00000275669 | miRNA | MIR6744 | Exon | 0.05 | down |
| chr20 | 32431653 | 32432423 | 771 | ENSG00000171456 | retained_intron | ASXL1 | Exon | 0.05 | down |
| chr6 | 43338412 | 43339371 | 960 | ENSG00000171467 | protein_coding | ZNF318 | Exon | 0.05 | down |
| chr17 | 4538825 | 4539035 | 211 | ENSG00000183018 | retained_intron | SPNS2 | 3' UTR | 0.05 | down |
| chr12 | 22625523 | 22625991 | 469 | ENSG00000139163 | nonsense_mediated_decay | ETNK1 | 3' UTR | 0.05 | down |
| chr17 | 67825532 | 67825743 | 212 | ENSG00000171634 | protein_coding | BPTF | 5' UTR | 0.05 | down |
| chr11 | 124884106 | 124887789 | 3684 | ENSG00000254568 | lncRNA | AP003501 | Exon | 0.05 | down |
| chr1 | 225497442 | 225501223 | 3782 | ENSG00000154380 | retained_intron | ENAH | 3' UTR | 0.05 | down |
| chr3 | 177023205 | 177023626 | 422 | ENSG00000177565 | processed_transcript | TBL1XR1 | 3' UTR | 0.05 | up |
| chr17 | 7315589 | 7317322 | 1734 | ENSG00000132522 | retained_intron | GPS2 | 3' UTR | 0.05 | up |
| chr3 | 157159127 | 157159308 | 182 | ENSG00000163660 | retained_intron | CCNL1 | Exon | 0.05 | up |
| chr7 | 143351633 | 143352083 | 451 | ENSG00000272619 | lncRNA | AC093673 | 3' UTR | 0.05 | up |
| chr1 | 184693862 | 184702872 | 9011 | ENSG00000116406 | processed_transcript | EDEM3 | 3' UTR | 0.05 | up |
| chr2 | 99162137 | 99163067 | 931 | ENSG00000144182 | protein_coding | LIPT1 | Exon | 0.05 | up |
| X | 47144799 | 47145038 | 240 | ENSG00000147123 | protein_coding | NDUFB11 | 5' UTR | 0.05 | up |
| chr14 | 21498755 | 21499025 | 271 | ENSG00000092203 | retained_intron | TOX4 | 3' UTR | 0.05 | up |
| chr5 | 103177918 | 103183274 | 5357 | ENSG00000145725 | protein_coding | PPIP5K2 | Exon | 0.05 | up |
| chr16 | 50035983 | 50036432 | 450 | ENSG00000205423 | protein_coding | CNEP1R1 | 3' UTR | 0.05 | up |
| chr12 | 113816766 | 113817003 | 238 | ENSG00000122965 | processed_transcript | RBM19 | 3' UTR | 0.05 | up |
| chr11 | 70384922 | 70385162 | 241 | ENSG00000131626 | protein_coding | PPFIA1 | 3' UTR | 0.05 | up |
| chr16 | 69365391 | 69365482 | 92 | ENSG00000132604 | retained_intron | TERF2 | 3' UTR | 0.05 | up |
| chr8 | 98136573 | 98136906 | 334 | ENSG00000104356 | protein_coding | POP1 | Exon | 0.05 | up |
| chr7 | 32992723 | 32995058 | 2336 | ENSG00000122642 | processed_transcript | FKBP9 | Exon | 0.05 | up |
| chr19 | 40572128 | 40572279 | 152 | ENSG00000160460 | retained_intron | SPTBN4 | 3' UTR | 0.05 | up |
| chr11 | 111879305 | 111879365 | 61 | ENSG00000258529 | protein_coding | AP001781 | 5' UTR | 0.05 | up |
| chr19 | 54107297 | 54107358 | 62 | ENSG00000170906 | processed_transcript | NDUFA3 | 3' UTR | 0.05 | down |
| chr1 | 178441583 | 178443120 | 1538 | ENSG00000075391 | protein_coding | RASAL2 | Exon | 0.05 | down |
| chr2 | 179109519 | 179109999 | 481 | ENSG00000187231 | nonsense_mediated_decay | SESTD1 | 3' UTR | 0.05 | down |
| chr13 | 76881821 | 76882332 | 512 | ENSG00000178695 | protein_coding | KCTD12 | 3' UTR | 0.05 | down |
| chr3 | 122740327 | 122741143 | 817 | ENSG00000169087 | processed_transcript | HSPBAP1 | 3' UTR | 0.05 | down |
| chr14 | 53043399 | 53043760 | 362 | ENSG00000100523 | processed_transcript | DDHD1 | 3' UTR | 0.05 | down |
| chr16 | 75568295 | 75568355 | 61 | ENSG00000034713 | protein_coding | GABARAPL2 | 3' UTR | 0.05 | down |
| chr12 | 30799921 | 30800311 | 391 | ENSG00000285517 | lncRNA | AC010198 | Exon | 0.05 | down |
| chr11 | 66563703 | 66563793 | 91 | ENSG00000174080 | retained_intron | CTSF | 3' UTR | 0.05 | down |
| chr2 | 162077356 | 162077651 | 296 | ENSG00000231445 | processed_pseudogene | TIMM8AP1 | Exon | 0.05 | down |
| chr6 | 43037616 | 43037707 | 92 | ENSG00000240625 | misc_RNA | RN7SL403P | 3' UTR | 0.05 | down |
| chr3 | 47273622 | 47277215 | 3594 | ENSG00000088727 | processed_transcript | KIF9 | 3' UTR | 0.05 | down |
| chr16 | 10942210 | 10942481 | 272 | ENSG00000182108 | protein_coding | DEXI | 3' UTR | 0.05 | down |
| chr19 | 32709339 | 32712118 | 2780 | ENSG00000173809 | protein_coding | TDRD12 | 3' UTR | 0.05 | down |
| chr12 | 64145759 | 64146240 | 482 | ENSG00000196935 | retained_intron | SRGAP1 | 3' UTR | 0.05 | down |
| chr5 | 173889994 | 173890444 | 451 | ENSG00000113742 | processed_transcript | CPEB4 | Exon | 0.05 | down |
| chr2 | 99196537 | 99196598 | 62 | ENSG00000185414 | nonsense_mediated_decay | MRPL30 | 3' UTR | 0.05 | down |
| X | 49250191 | 49250251 | 61 | ENSG00000101997 | processed_transcript | CCDC22 | Exon | 0.05 | down |
| chr7 | 152176438 | 152180794 | 4357 | ENSG00000055609 | protein_coding | KMT2C | Exon | 0.05 | down |
| chr19 | 58455202 | 58455801 | 600 | ENSG00000249471 | protein_coding | ZNF324B | Exon | 0.05 | down |
| chr21 | 36749914 | 36750334 | 421 | ENSG00000224269 | lncRNA | AP000697 | 3' UTR | 0.05 | up |
| chr16 | 68695876 | 68696155 | 280 | ENSG00000062038 | processed_transcript | CDH3 | 3' UTR | 0.05 | up |
| chr3 | 49316748 | 49318386 | 1639 | ENSG00000114316 | processed_transcript | USP4 | 3' UTR | 0.05 | up |
| chr10 | 133295386 | 133295977 | 592 | ENSG00000235245 | lncRNA | AL360181 | Exon | 0.05 | up |
| chr6 | 149919041 | 149919774 | 734 | ENSG00000203722 | nonsense_mediated_decay | RAET1G | Exon | 0.05 | up |
| chr20 | 56638658 | 56638837 | 180 | ENSG00000087510 | protein_coding | TFAP2C | 3' UTR | 0.05 | up |
| chr4 | 118279635 | 118279875 | 241 | ENSG00000275994 | snoRNA | SNORA24 | Exon | 0.05 | up |
| chr17 | 16566603 | 16567909 | 1307 | ENSG00000141040 | protein_coding | ZNF287 | 5' UTR | 0.05 | up |
| chr11 | 47293914 | 47295577 | 1664 | ENSG00000110514 | protein_coding | MADD | 5' UTR | 0.05 | up |
| chr2 | 46582715 | 46583433 | 719 | ENSG00000119729 | retained_intron | RHOQ | 3' UTR | 0.05 | up |
| chr4 | 84733432 | 84735076 | 1645 | ENSG00000163625 | protein_coding | WDFY3 | Exon | 0.05 | up |
| chr3 | 47169176 | 47175857 | 6682 | ENSG00000227398 | lncRNA | KIF9-AS1 | Exon | 0.05 | up |
| chr6 | 100864117 | 100867991 | 3875 | ENSG00000112249 | retained_intron | ASCC3 | Exon | 0.05 | up |
| chr6 | 111265973 | 111266694 | 722 | ENSG00000173214 | processed_transcript | MFSD4B | 3' UTR | 0.05 | up |
| chr19 | 48807331 | 48807721 | 391 | ENSG00000105552 | retained_intron | BCAT2 | Exon | 0.05 | up |
| chr21 | 46300446 | 46300776 | 331 | ENSG00000160298 | processed_transcript | C21orf58 | 3' UTR | 0.05 | up |
| chr1 | 19679439 | 19679829 | 391 | ENSG00000158748 | protein_coding | HTR6 | 3' UTR | 0.05 | up |
| chr1 | 173886034 | 173886365 | 332 | ENSG00000185278 | protein_coding | ZBTB37 | 3' UTR | 0.05 | up |
| chr9 | 15478547 | 15485951 | 7405 | ENSG00000164985 | processed_transcript | PSIP1 | Exon | 0.05 | up |
| chr2 | 178571421 | 178571602 | 182 | ENSG00000155657 | protein_coding | TTN | Exon | 0.05 | up |
| chr12 | 116109798 | 116109948 | 151 | ENSG00000123066 | retained_intron | MED13L | 3' UTR | 0.05 | down |
| chr15 | 68212058 | 68212358 | 301 | ENSG00000128973 | retained_intron | CLN6 | Exon | 0.05 | down |
| chr21 | 36369076 | 36369796 | 721 | ENSG00000159256 | nonsense_mediated_decay | MORC3 | 5' UTR | 0.05 | down |
| chr10 | 60029026 | 60029507 | 482 | ENSG00000151150 | retained_intron | ANK3 | 3' UTR | 0.05 | down |
| chr15 | 82817934 | 82823259 | 5326 | ENSG00000156232 | retained_intron | WHAMM | Exon | 0.05 | down |
| chr14 | 96285183 | 96289493 | 4311 | ENSG00000066739 | retained_intron | ATG2B | 3' UTR | 0.05 | down |
| chr3 | 114079360 | 114080036 | 677 | ENSG00000151576 | retained_intron | QTRT2 | Exon | 0.05 | down |
| chr16 | 89521411 | 89521892 | 482 | ENSG00000197912 | retained_intron | SPG7 | Exon | 0.05 | down |
| chr8 | 94387068 | 94393810 | 6743 | ENSG00000197275 | retained_intron | RAD54B | 3' UTR | 0.05 | down |
| chr5 | 181233742 | 181233922 | 181 | ENSG00000146063 | processed_transcript | TRIM41 | 3' UTR | 0.05 | down |
| chr1 | 63651659 | 63651720 | 62 | ENSG00000079739 | processed_transcript | PGM1 | Exon | 0.05 | down |
| chr11 | 65538804 | 65538925 | 122 | ENSG00000168056 | protein_coding | LTBP3 | 3' UTR | 0.05 | down |
| chr6 | 116238962 | 116244257 | 5296 | ENSG00000111817 | protein_coding | DSE | 3' UTR | 0.05 | down |
| chr5 | 136178842 | 136179741 | 900 | ENSG00000113658 | nonsense_mediated_decay | SMAD5 | 3' UTR | 0.05 | down |
| chr7 | 149861505 | 149862492 | 988 | ENSG00000273419 | lncRNA | AC004877 | Exon | 0.05 | down |
| chr17 | 17248932 | 17260353 | 11422 | ENSG00000141030 | protein_coding | COPS3 | 3' UTR | 0.05 | down |
| chr20 | 50889215 | 50889785 | 571 | ENSG00000101126 | protein_coding | ADNP | 3' UTR | 0.05 | down |
| chr19 | 1228100 | 1228431 | 332 | ENSG00000118046 | protein_coding | STK11 | 3' UTR | 0.05 | up |
| chr2 | 200871025 | 200877595 | 6571 | ENSG00000240344 | retained_intron | PPIL3 | 3' UTR | 0.05 | up |
| chr2 | 223756272 | 223756781 | 510 | ENSG00000152056 | protein_coding | AP1S3 | 3' UTR | 0.05 | up |
| chr4 | 25790544 | 25818225 | 27682 | ENSG00000091490 | protein_coding | SEL1L3 | Exon | 0.05 | up |
| chr11 | 110435924 | 110462819 | 26896 | ENSG00000137714 | protein_coding | FDX1 | 3' UTR | 0.05 | up |
| chr2 | 219279850 | 219280031 | 182 | ENSG00000135924 | retained_intron | DNAJB2 | Exon | 0.05 | up |
| chr19 | 40778245 | 40779109 | 865 | ENSG00000167578 | protein_coding | RAB4B | 5' UTR | 0.05 | up |
| chr12 | 76846658 | 76849616 | 2959 | ENSG00000186908 | retained_intron | ZDHHC17 | Exon | 0.05 | up |
| chr8 | 66921950 | 66922374 | 425 | ENSG00000254341 | snoRNA | SNORD87 | Exon | 0.05 | up |
| chr6 | 158502866 | 158503286 | 421 | ENSG00000146433 | protein_coding | TMEM181 | Exon | 0.05 | up |
| chr1 | 74705779 | 74706078 | 300 | ENSG00000116791 | processed_transcript | CRYZ | 3' UTR | 0.05 | up |
| chr2 | 27369062 | 27369562 | 501 | ENSG00000115211 | nonsense_mediated_decay | EIF2B4 | 3' UTR | 0.05 | up |
| chr12 | 48837354 | 48837715 | 362 | ENSG00000174243 | retained_intron | DDX23 | Exon | 0.05 | up |
| chr5 | 80140725 | 80141146 | 422 | ENSG00000164300 | retained_intron | SERINC5 | 3' UTR | 0.05 | up |
| chr1 | 3778618 | 3778828 | 211 | ENSG00000130764 | nonsense_mediated_decay | LRRC47 | 3' UTR | 0.05 | up |
| chr10 | 114951099 | 114959886 | 8788 | ENSG00000165832 | processed_transcript | TRUB1 | Exon | 0.05 | up |
| chr6 | 7568523 | 7569210 | 688 | ENSG00000096696 | retained_intron | DSP | Exon | 0.05 | up |
| chr17 | 44299663 | 44299813 | 151 | ENSG00000267750 | lncRNA | RUNDC3A-AS1 | Exon | 0.05 | up |
| chr21 | 6116515 | 6116966 | 452 | ENSG00000275993 | retained_intron | SIK1B | Exon | 0.05 | down |
| chr15 | 55205362 | 55205692 | 331 | ENSG00000137876 | processed_transcript | RSL24D1 | 3' UTR | 0.05 | down |
| chr16 | 19075726 | 19078178 | 2453 | ENSG00000167186 | processed_transcript | COQ7 | 3' UTR | 0.05 | down |
| chr3 | 186582023 | 186597103 | 15081 | ENSG00000283390 | processed_pseudogene | AC068631 | 3' UTR | 0.05 | down |
| chr12 | 57767082 | 57768986 | 1905 | ENSG00000111012 | retained_intron | CYP27B1 | 3' UTR | 0.05 | down |
| chr3 | 196270448 | 196287199 | 16752 | ENSG00000161217 | protein_coding | PCYT1A | 3' UTR | 0.05 | down |
| chr8 | 98949036 | 98949515 | 480 | ENSG00000164920 | protein_coding | OSR2 | Exon | 0.05 | down |
| chr15 | 79457414 | 79463112 | 5699 | ENSG00000169330 | nonsense_mediated_decay | MINAR1 | Exon | 0.05 | down |
| chr5 | 90810324 | 90815625 | 5302 | ENSG00000164199 | retained_intron | ADGRV1 | Exon | 0.05 | down |
| chr6 | 34284118 | 34284299 | 182 | ENSG00000220583 | processed_pseudogene | RPL35P2 | 3' UTR | 0.05 | down |
| chr8 | 61659835 | 61660315 | 481 | ENSG00000198363 | retained_intron | ASPH | 3' UTR | 0.05 | down |
| chr2 | 134866523 | 134867690 | 1168 | ENSG00000224043 | lncRNA | CCNT2-AS1 | Exon | 0.05 | down |
| chr2 | 231108407 | 231109270 | 864 | ENSG00000135914 | protein_coding | HTR2B | 3' UTR | 0.05 | down |
| chr19 | 18637024 | 18659784 | 22761 | ENSG00000167487 | protein_coding | KLHL26 | 3' UTR | 0.05 | down |
| chr19 | 38922247 | 38930129 | 7883 | ENSG00000104835 | protein_coding | SARS2 | 3' UTR | 0.05 | down |
| chr19 | 58257299 | 58258222 | 924 | ENSG00000268516 | lncRNA | AC020915 | Exon | 0.05 | down |
| chr15 | 65119791 | 65132970 | 13180 | ENSG00000090470 | protein_coding | PDCD7 | Exon | 0.05 | down |
| chr16 | 3137201 | 3137471 | 271 | ENSG00000085644 | protein_coding | ZNF213 | 5' UTR | 0.05 | down |
| chr17 | 54936656 | 54937386 | 731 | ENSG00000141198 | retained_intron | TOM1L1 | 3' UTR | 0.05 | down |
| chr14 | 73058531 | 73058801 | 271 | ENSG00000119707 | protein_coding | RBM25 | 5' UTR | 0.05 | down |
| chr9 | 73166111 | 73167685 | 1575 | ENSG00000135046 | processed_transcript | ANXA1 | Exon | 0.05 | down |
| chr16 | 89711855 | 89712064 | 210 | ENSG00000261373 | lncRNA | VPS9D1-AS1 | Exon | 0.05 | down |
| chr19 | 56141099 | 56142381 | 1283 | ENSG00000167685 | processed_transcript | ZNF444 | 5' UTR | 0.05 | down |
| chr12 | 6327686 | 6328446 | 761 | ENSG00000008323 | protein_coding | PLEKHG6 | 3' UTR | 0.05 | down |
| chr11 | 74611534 | 74628937 | 17404 | ENSG00000077514 | protein_coding | POLD3 | 5' UTR | 0.05 | down |
| chr12 | 76025806 | 76026077 | 272 | ENSG00000139289 | protein_coding | PHLDA1 | 3' UTR | 0.05 | up |
| chr10 | 124760264 | 124765733 | 5470 | ENSG00000203791 | nonsense_mediated_decay | EEF1AKMT2 | 3' UTR | 0.05 | up |
| chr11 | 33541804 | 33542314 | 511 | ENSG00000110427 | protein_coding | KIAA1549L | Exon | 0.05 | up |
| chr4 | 88036301 | 88043289 | 6989 | ENSG00000118762 | protein_coding | PKD2 | 5' UTR | 0.05 | up |
| chr9 | 134031292 | 134034606 | 3315 | ENSG00000235106 | processed_transcript | BRD3OS | 3' UTR | 0.05 | up |
| chr12 | 122261381 | 122263686 | 2306 | ENSG00000139719 | nonsense_mediated_decay | VPS33A | 3' UTR | 0.05 | up |
| X | 108088723 | 108091549 | 2827 | ENSG00000101843 | protein_coding | PSMD10 | 5' UTR | 0.05 | up |
| chr1 | 180989990 | 181005346 | 15357 | ENSG00000135823 | processed_transcript | STX6 | 5' UTR | 0.05 | up |
| chr21 | 29062845 | 29064976 | 2132 | ENSG00000156261 | retained_intron | CCT8 | Exon | 0.05 | up |
| chr12 | 62592618 | 62593159 | 542 | ENSG00000061987 | retained_intron | MON2 | 3' UTR | 0.05 | up |
| chr16 | 56730117 | 56740770 | 10654 | ENSG00000102900 | protein_coding | NUP93 | 5' UTR | 0.05 | up |
| chr10 | 125812264 | 125816206 | 3943 | ENSG00000188690 | nonsense_mediated_decay | UROS | Exon | 0.05 | up |
| chr3 | 9448528 | 9449528 | 1001 | ENSG00000168137 | processed_transcript | SETD5 | 3' UTR | 0.05 | up |
| chr7 | 48275255 | 48276336 | 1082 | ENSG00000179869 | processed_transcript | ABCA13 | Exon | 0.05 | up |
| chr13 | 110869411 | 110869502 | 92 | ENSG00000255874 | lncRNA | PRECSIT | Exon | 0.04 | down |
| chr3 | 138704461 | 138707396 | 2936 | ENSG00000051382 | retained_intron | PIK3CB | 3' UTR | 0.04 | down |
| chr3 | 96814580 | 96814761 | 182 | ENSG00000080224 | protein_coding | EPHA6 | 5' UTR | 0.04 | down |
| chr4 | 68330302 | 68337139 | 6838 | ENSG00000083896 | retained_intron | YTHDC1 | Exon | 0.04 | down |
| chr12 | 96268325 | 96268506 | 182 | ENSG00000111145 | processed_transcript | ELK3 | 3' UTR | 0.04 | down |
| chr1 | 10459878 | 10460269 | 392 | ENSG00000160049 | nonsense_mediated_decay | DFFA | 3' UTR | 0.04 | down |
| chr17 | 40630753 | 40631660 | 908 | ENSG00000073584 | retained_intron | SMARCE1 | 3' UTR | 0.04 | down |
| chr9 | 129507369 | 129507610 | 242 | ENSG00000204054 | lncRNA | LINC00963 | Exon | 0.04 | down |
| chr17 | 50635870 | 50643594 | 7725 | ENSG00000108846 | nonsense_mediated_decay | ABCC3 | 3' UTR | 0.04 | down |
| chr14 | 24190218 | 24190339 | 122 | ENSG00000259522 | nonsense_mediated_decay | AL136295 | 3' UTR | 0.04 | down |
| chr6 | 29888679 | 29889443 | 765 | ENSG00000206341 | unprocessed_pseudogene | HLA-H | Exon | 0.04 | down |
| chr21 | 36751951 | 36752252 | 302 | ENSG00000224269 | lncRNA | AP000697 | 3' UTR | 0.04 | down |
| chr11 | 65866237 | 65867294 | 1058 | ENSG00000172638 | nonsense_mediated_decay | EFEMP2 | 3' UTR | 0.04 | down |
| chr14 | 75002920 | 75003220 | 301 | ENSG00000119718 | protein_coding | EIF2B2 | 5' UTR | 0.04 | down |
| chr11 | 47617967 | 47618295 | 329 | ENSG00000109919 | processed_transcript | MTCH2 | 3' UTR | 0.04 | down |
| chr15 | 40698785 | 40709097 | 10313 | ENSG00000051180 | nonsense_mediated_decay | RAD51 | 3' UTR | 0.04 | down |
| chr3 | 113532554 | 113532855 | 302 | ENSG00000072858 | retained_intron | SIDT1 | 5' UTR | 0.04 | up |
| chr11 | 72098103 | 72098163 | 61 | ENSG00000149357 | protein_coding | LAMTOR1 | 3' UTR | 0.04 | up |
| chr17 | 2318911 | 2323161 | 4251 | ENSG00000167720 | protein_coding | SRR | 5' UTR | 0.04 | up |
| chr9 | 132328183 | 132329743 | 1561 | ENSG00000107290 | protein_coding | SETX | Exon | 0.04 | up |
| chr10 | 68756450 | 68760886 | 4437 | ENSG00000060339 | protein_coding | CCAR1 | 3' UTR | 0.04 | up |
| chr3 | 149982773 | 149982982 | 210 | ENSG00000224831 | processed_pseudogene | TMEM183B | Exon | 0.04 | up |
| X | 37442185 | 37453846 | 11662 | ENSG00000241607 | unprocessed_pseudogene | AC245096 | 3' UTR | 0.04 | up |
| chr2 | 232791363 | 232794935 | 3573 | ENSG00000204120 | nonsense_mediated_decay | GIGYF2 | 3' UTR | 0.04 | up |
| chr11 | 72095496 | 72095675 | 180 | ENSG00000184154 | processed_transcript | LRTOMT | 3' UTR | 0.04 | up |
| chr14 | 70742458 | 70742579 | 122 | ENSG00000006432 | retained_intron | MAP3K9 | Exon | 0.04 | up |
| chr1 | 146995244 | 146995484 | 241 | ENSG00000268043 | protein_coding | NBPF12 | 3' UTR | 0.04 | up |
| chr7 | 130184220 | 130185366 | 1147 | ENSG00000146842 | retained_intron | TMEM209 | Exon | 0.04 | up |
| chr21 | 39296652 | 39296983 | 332 | ENSG00000185658 | retained_intron | BRWD1 | 3' UTR | 0.04 | up |
| chr11 | 118478039 | 118481848 | 3810 | ENSG00000118058 | processed_transcript | KMT2A | Exon | 0.04 | up |
| chr15 | 43184373 | 43185141 | 769 | ENSG00000166946 | retained_intron | CCNDBP1 | Exon | 0.04 | up |
| chr12 | 98605809 | 98606259 | 451 | ENSG00000075415 | retained_intron | SLC25A3 | 3' UTR | 0.04 | up |
| chr16 | 85079141 | 85079621 | 481 | ENSG00000135709 | protein_coding | KIAA0513 | 3' UTR | 0.04 | up |
| chr7 | 99908593 | 99908773 | 181 | ENSG00000146833 | retained_intron | TRIM4 | Exon | 0.04 | up |
| chr5 | 124648650 | 124649011 | 362 | ENSG00000168916 | processed_transcript | ZNF608 | Exon | 0.04 | up |
| chr19 | 52016752 | 52017627 | 876 | ENSG00000197619 | protein_coding | ZNF615 | 3' UTR | 0.04 | up |
| chr1 | 93136770 | 93137280 | 511 | ENSG00000143033 | processed_transcript | MTF2 | 3' UTR | 0.04 | up |
| chr1 | 153628970 | 153629211 | 242 | ENSG00000160678 | protein_coding | S100A1 | Exon | 0.04 | down |
| chr17 | 76559231 | 76559292 | 62 | ENSG00000274091 | snoRNA | SNORD1C | Exon | 0.04 | down |
| chr1 | 44104488 | 44105058 | 571 | ENSG00000230615 | lncRNA | AL139220 | Exon | 0.04 | down |
| chr2 | 25926687 | 25926867 | 181 | ENSG00000084731 | processed_transcript | KIF3C | 3' UTR | 0.04 | down |
| chr16 | 70575750 | 70575931 | 182 | ENSG00000189091 | processed_transcript | SF3B3 | 3' UTR | 0.04 | down |
| chr2 | 97834630 | 97834848 | 219 | ENSG00000075568 | retained_intron | TMEM131 | Exon | 0.04 | down |
| chr17 | 15973936 | 15974026 | 91 | ENSG00000170425 | retained_intron | ADORA2B | Exon | 0.04 | down |
| chr17 | 42580888 | 42581128 | 241 | ENSG00000131470 | protein_coding | PSMC3IP | 3' UTR | 0.04 | down |
| chr8 | 76681298 | 76683832 | 2535 | ENSG00000091656 | protein_coding | ZFHX4 | 5' UTR | 0.04 | down |
| chr16 | 89918026 | 89918233 | 208 | ENSG00000258839 | protein_coding | MC1R | 5' UTR | 0.04 | down |
| chr17 | 1780921 | 1781773 | 853 | ENSG00000186532 | retained_intron | SMYD4 | 3' UTR | 0.04 | down |
| chr14 | 73111615 | 73119918 | 8304 | ENSG00000119707 | retained_intron | RBM25 | 3' UTR | 0.04 | down |
| chr19 | 43593276 | 43595408 | 2133 | ENSG00000167378 | protein_coding | IRGQ | 5' UTR | 0.04 | down |
| chr8 | 123328247 | 123337765 | 9519 | ENSG00000283172 | miRNA | MIR548D1 | 3' UTR | 0.04 | down |
| chr15 | 89326733 | 89327117 | 385 | ENSG00000275101 | miRNA | MIR6766 | 3' UTR | 0.04 | down |
| chr17 | 783097 | 783365 | 269 | ENSG00000171861 | protein_coding | MRM3 | Exon | 0.04 | up |
| chr5 | 149628809 | 149632394 | 3586 | ENSG00000183111 | retained_intron | ARHGEF37 | 3' UTR | 0.04 | up |
| chr17 | 42674372 | 42675937 | 1566 | ENSG00000068137 | processed_transcript | PLEKHH3 | Exon | 0.04 | up |
| chr2 | 169813133 | 169821153 | 8021 | ENSG00000138382 | retained_intron | METTL5 | 3' UTR | 0.04 | up |
| X | 123885825 | 123907290 | 21466 | ENSG00000101966 | processed_transcript | XIAP | 3' UTR | 0.04 | up |
| chr20 | 62295402 | 62296063 | 662 | ENSG00000130703 | retained_intron | OSBPL2 | 3' UTR | 0.04 | up |
| chr11 | 130139638 | 130140572 | 935 | ENSG00000084234 | retained_intron | APLP2 | 3' UTR | 0.04 | up |
| chr19 | 18589980 | 18590100 | 121 | ENSG00000006015 | retained_intron | REX1BD | Exon | 0.04 | up |
| chr21 | 29046974 | 29050130 | 3157 | ENSG00000156256 | retained_intron | USP16 | Exon | 0.04 | up |
| chr19 | 24036773 | 24037194 | 422 | ENSG00000268362 | lncRNA | AC092279 | Exon | 0.04 | up |
| chr12 | 42446725 | 42447325 | 601 | ENSG00000134283 | TEC | PPHLN1 | 3' UTR | 0.04 | up |
| chr15 | 74890064 | 74890155 | 92 | ENSG00000178802 | protein_coding | MPI | 5' UTR | 0.04 | up |
| chr1 | 43452708 | 43452828 | 121 | ENSG00000178922 | retained_intron | HYI | Exon | 0.04 | up |
| chr2 | 88628230 | 88628500 | 271 | ENSG00000234028 | lncRNA | EIF2AK3-DT | Exon | 0.04 | up |
| chr10 | 67956878 | 67964939 | 8062 | ENSG00000226318 | processed_pseudogene | RPS3AP38 | 3' UTR | 0.04 | up |
| chr2 | 39012137 | 39013911 | 1775 | ENSG00000115904 | retained_intron | SOS1 | Exon | 0.04 | up |
| chr5 | 150302475 | 150302745 | 271 | ENSG00000183876 | protein_coding | ARSI | 5' UTR | 0.04 | up |
| X | 85091878 | 85092238 | 361 | ENSG00000155008 | protein_coding | APOOL | 3' UTR | 0.04 | up |
| chr6 | 144835870 | 144836383 | 514 | ENSG00000152818 | processed_transcript | UTRN | Exon | 0.04 | up |
| chr1 | 9583285 | 9583436 | 152 | ENSG00000188807 | protein_coding | TMEM201 | 3' UTR | 0.04 | down |
| chr7 | 22991134 | 23014100 | 22967 | ENSG00000122591 | protein_coding | FAM126A | 5' UTR | 0.04 | down |
| chr6 | 145686128 | 145686916 | 789 | ENSG00000112425 | protein_coding | EPM2A | 3' UTR | 0.04 | down |
| chr19 | 34683157 | 34684181 | 1025 | ENSG00000089335 | retained_intron | ZNF302 | 3' UTR | 0.04 | down |
| chr15 | 39600693 | 39618129 | 17437 | ENSG00000150667 | nonsense_mediated_decay | FSIP1 | 3' UTR | 0.04 | down |
| chr2 | 241725998 | 241726239 | 242 | ENSG00000168395 | processed_transcript | ING5 | 3' UTR | 0.04 | down |
| chr6 | 109145361 | 109146828 | 1468 | ENSG00000183137 | protein_coding | CEP57L1 | 5' UTR | 0.04 | down |
| chr8 | 120538347 | 120538917 | 571 | ENSG00000172167 | processed_transcript | MTBP | 3' UTR | 0.04 | down |
| chr3 | 159886302 | 159886393 | 92 | ENSG00000151967 | retained_intron | SCHIP1 | Exon | 0.04 | down |
| chr12 | 31087716 | 31087837 | 122 | ENSG00000013573 | retained_intron | DDX11 | Exon | 0.04 | down |
| chr5 | 157304249 | 157304340 | 92 | ENSG00000055163 | retained_intron | CYFIP2 | 3' UTR | 0.04 | down |
| chr3 | 58409000 | 58413132 | 4133 | ENSG00000168297 | protein_coding | PXK | 3' UTR | 0.04 | down |
| chr2 | 162075093 | 162077822 | 2730 | ENSG00000231445 | processed_pseudogene | TIMM8AP1 | Exon | 0.04 | down |
| chr19 | 3613541 | 3613811 | 271 | ENSG00000226800 | lncRNA | CACTIN-AS1 | Exon | 0.04 | down |
| chr8 | 42534748 | 42536425 | 1678 | ENSG00000168575 | nonsense_mediated_decay | SLC20A2 | 5' UTR | 0.04 | down |
| chr22 | 30662066 | 30662842 | 777 | ENSG00000288153 | unprocessed_pseudogene | AC003072 | 3' UTR | 0.04 | down |
| chr4 | 76131271 | 76134357 | 3087 | ENSG00000138750 | retained_intron | NUP54 | 3' UTR | 0.04 | up |
| chr7 | 83960690 | 83963267 | 2578 | ENSG00000075213 | protein_coding | SEMA3A | 3' UTR | 0.04 | up |
| chr14 | 44963057 | 44964374 | 1318 | ENSG00000198718 | retained_intron | TOGARAM1 | Exon | 0.04 | up |
| chr3 | 23499629 | 23532562 | 32934 | ENSG00000182247 | protein_coding | UBE2E2 | Exon | 0.04 | up |
| chr3 | 184964510 | 184982553 | 18044 | ENSG00000156931 | retained_intron | VPS8 | Exon | 0.04 | up |
| chr14 | 96520173 | 96526095 | 5923 | ENSG00000090060 | retained_intron | PAPOLA | Exon | 0.04 | up |
| chr16 | 74468960 | 74469171 | 212 | ENSG00000090863 | retained_intron | GLG1 | 3' UTR | 0.04 | up |
| chr11 | 73647675 | 73647826 | 152 | ENSG00000021300 | retained_intron | PLEKHB1 | 5' UTR | 0.04 | up |
| chr5 | 54519286 | 54519707 | 422 | ENSG00000178996 | protein_coding | SNX18 | Exon | 0.04 | up |
| chr2 | 85318109 | 85318230 | 122 | ENSG00000152291 | protein_coding | TGOLN2 | 3' UTR | 0.04 | up |
| chr13 | 94574501 | 94574831 | 331 | ENSG00000088451 | retained_intron | TGDS | 3' UTR | 0.04 | up |
| X | 155941956 | 155942046 | 91 | ENSG00000124334 | protein_coding | IL9R | 3' UTR | 0.04 | up |
| chr9 | 89361926 | 89362317 | 392 | ENSG00000187764 | protein_coding | SEMA4D | 3' UTR | 0.04 | up |
| chr9 | 92310683 | 92311183 | 501 | ENSG00000198000 | retained_intron | NOL8 | 3' UTR | 0.04 | up |
| chr9 | 75066779 | 75066930 | 152 | ENSG00000106733 | processed_transcript | NMRK1 | 3' UTR | 0.04 | up |
| chr17 | 4789249 | 4789877 | 629 | ENSG00000182327 | protein_coding | GLTPD2 | Exon | 0.04 | up |
| chr2 | 132644870 | 132645081 | 212 | ENSG00000150551 | nonsense_mediated_decay | LYPD1 | 3' UTR | 0.04 | down |
| chr2 | 175993207 | 175994238 | 1032 | ENSG00000144320 | protein_coding | LNPK | Exon | 0.04 | down |
| chr9 | 137051018 | 137051280 | 263 | ENSG00000054179 | retained_intron | ENTPD2 | Exon | 0.04 | down |
| chr17 | 82458730 | 82458955 | 226 | ENSG00000141562 | protein_coding | NARF | 5' UTR | 0.04 | down |
| chr22 | 31101755 | 31102541 | 787 | ENSG00000183963 | retained_intron | SMTN | Exon | 0.04 | down |
| chr16 | 25031891 | 25032691 | 801 | ENSG00000262587 | transcribed_unprocessed_pseudogene | AC133552 | Exon | 0.04 | down |
| chr10 | 86936406 | 86937065 | 660 | ENSG00000173269 | processed_transcript | MMRN2 | 3' UTR | 0.04 | down |
| chr16 | 4339327 | 4339388 | 62 | ENSG00000126603 | protein_coding | GLIS2 | 3' UTR | 0.04 | down |
| chr16 | 89324282 | 89324962 | 681 | ENSG00000261253 | lncRNA | AC137932 | Exon | 0.04 | down |
| chr14 | 60723781 | 60724351 | 571 | ENSG00000100625 | protein_coding | SIX4 | 5' UTR | 0.04 | down |
| chr17 | 38491459 | 38491639 | 181 | ENSG00000275832 | retained_intron | ARHGAP23 | Exon | 0.04 | down |
| chr2 | 167868947 | 167870296 | 1350 | ENSG00000172318 | protein_coding | B3GALT1 | 3' UTR | 0.04 | down |
| chr3 | 157459939 | 157489170 | 29232 | ENSG00000197415 | processed_transcript | VEPH1 | 3' UTR | 0.04 | down |
| chr19 | 40396851 | 40397661 | 811 | ENSG00000105227 | nonsense_mediated_decay | PRX | 3' UTR | 0.04 | down |
| chr19 | 48443852 | 48444691 | 840 | ENSG00000105447 | protein_coding | GRWD1 | 3' UTR | 0.04 | up |
| chr12 | 56181504 | 56181791 | 288 | ENSG00000139613 | retained_intron | SMARCC2 | 3' UTR | 0.04 | up |
| chr1 | 155900229 | 155904311 | 4083 | ENSG00000143622 | retained_intron | RIT1 | 3' UTR | 0.04 | up |
| chr15 | 38484568 | 38484928 | 361 | ENSG00000286786 | lncRNA | AC116158 | 3' UTR | 0.04 | up |
| chr5 | 31526324 | 31526475 | 152 | ENSG00000113360 | protein_coding | DROSHA | Exon | 0.04 | up |
| chr9 | 132242549 | 132242879 | 331 | ENSG00000196358 | processed_transcript | NTNG2 | 3' UTR | 0.04 | up |
| chr9 | 34649479 | 34649600 | 122 | ENSG00000213930 | processed_transcript | GALT | 3' UTR | 0.04 | up |
| chr1 | 182877578 | 182877728 | 151 | ENSG00000135829 | processed_transcript | DHX9 | Exon | 0.04 | up |
| chr10 | 128107860 | 128108400 | 541 | ENSG00000148773 | processed_transcript | MKI67 | Exon | 0.04 | up |
| chr6 | 18237392 | 18244624 | 7233 | ENSG00000124795 | retained_intron | DEK | 3' UTR | 0.04 | up |
| chr14 | 73963309 | 73963640 | 332 | ENSG00000119723 | processed_transcript | COQ6 | 3' UTR | 0.04 | up |
| chr19 | 44235918 | 44236188 | 271 | ENSG00000131115 | processed_transcript | ZNF227 | Exon | 0.04 | up |
| chr20 | 46725083 | 46725264 | 182 | ENSG00000197496 | processed_transcript | SLC2A10 | Exon | 0.04 | down |
| chr3 | 183020217 | 183022457 | 2241 | ENSG00000078070 | retained_intron | MCCC1 | 3' UTR | 0.04 | down |
| chr6 | 42652560 | 42658106 | 5547 | ENSG00000206848 | snRNA | RNU6-890P | Exon | 0.04 | down |
| chr17 | 31863512 | 31873279 | 9768 | ENSG00000108651 | protein_coding | UTP6 | 3' UTR | 0.04 | down |
| chr16 | 31120723 | 31120904 | 182 | ENSG00000278133 | lncRNA | AC135050 | Exon | 0.04 | down |
| chr12 | 8095602 | 8095842 | 241 | ENSG00000089818 | retained_intron | NECAP1 | 3' UTR | 0.04 | down |
| chr18 | 5243755 | 5244295 | 541 | ENSG00000263753 | lncRNA | LINC00667 | Exon | 0.04 | down |
| chr16 | 71474933 | 71476252 | 1320 | ENSG00000157429 | protein_coding | ZNF19 | 3' UTR | 0.04 | down |
| chr11 | 47326905 | 47327116 | 212 | ENSG00000110514 | retained_intron | MADD | Exon | 0.04 | down |
| chr17 | 76570618 | 76570767 | 150 | ENSG00000070731 | nonsense_mediated_decay | ST6GALNAC2 | Exon | 0.04 | down |
| chr6 | 28124608 | 28129577 | 4970 | ENSG00000196812 | protein_coding | ZSCAN16 | 5' UTR | 0.04 | down |
| chr10 | 100993264 | 100993654 | 391 | ENSG00000107816 | protein_coding | LZTS2 | 3' UTR | 0.04 | down |
| chr17 | 61945660 | 61946965 | 1306 | ENSG00000108510 | retained_intron | MED13 | 3' UTR | 0.04 | down |
| chr11 | 59800940 | 59801240 | 301 | ENSG00000166900 | protein_coding | STX3 | 3' UTR | 0.04 | down |
| chr1 | 45494045 | 45494225 | 181 | ENSG00000070759 | protein_coding | TESK2 | 3' UTR | 0.04 | down |
| X | 53415054 | 53421900 | 6847 | ENSG00000072501 | protein_coding | SMC1A | Exon | 0.04 | down |
| chr1 | 52083502 | 52086342 | 2841 | ENSG00000134717 | processed_transcript | BTF3L4 | 3' UTR | 0.04 | down |
| chr1 | 10431233 | 10451405 | 20173 | ENSG00000175279 | retained_intron | CENPS | 3' UTR | 0.04 | down |
| chr5 | 179614177 | 179614358 | 182 | ENSG00000169045 | retained_intron | HNRNPH1 | 3' UTR | 0.04 | down |
| chr9 | 114359624 | 114362430 | 2807 | ENSG00000106948 | processed_transcript | AKNA | Exon | 0.04 | down |
| chr9 | 128178425 | 128178486 | 62 | ENSG00000148337 | processed_transcript | CIZ1 | Exon | 0.04 | up |
| chr3 | 122528043 | 122531913 | 3871 | ENSG00000114030 | protein_coding | KPNA1 | 3' UTR | 0.04 | up |
| chr8 | 23071376 | 23074459 | 3084 | ENSG00000253616 | lncRNA | AC107959 | Exon | 0.04 | up |
| chr1 | 1490223 | 1490493 | 271 | ENSG00000160072 | retained_intron | ATAD3B | 3' UTR | 0.04 | up |
| chr16 | 23522403 | 23523034 | 632 | ENSG00000103365 | protein_coding | GGA2 | 3' UTR | 0.04 | up |
| chr20 | 32444438 | 32444678 | 241 | ENSG00000197183 | protein_coding | NOL4L | 3' UTR | 0.04 | up |
| chr3 | 27633243 | 27633720 | 478 | ENSG00000271943 | processed_transcript | AC098614 | Exon | 0.04 | up |
| chr20 | 36892869 | 36893107 | 239 | ENSG00000101347 | nonsense_mediated_decay | SAMHD1 | 3' UTR | 0.04 | up |
| chr2 | 69937270 | 69938512 | 1243 | ENSG00000059728 | processed_transcript | MXD1 | 3' UTR | 0.04 | up |
| chr2 | 178855367 | 178865842 | 10476 | ENSG00000163492 | processed_transcript | CCDC141 | Exon | 0.04 | up |
| chr6 | 127286856 | 127287787 | 932 | ENSG00000118518 | protein_coding | RNF146 | 3' UTR | 0.04 | up |
| chr20 | 46348528 | 46348829 | 302 | ENSG00000080189 | processed_transcript | SLC35C2 | 3' UTR | 0.04 | up |
| chr6 | 56482405 | 56482766 | 362 | ENSG00000151914 | retained_intron | DST | Exon | 0.04 | up |
| chr11 | 85711145 | 85714595 | 3451 | ENSG00000137501 | protein_coding | SYTL2 | 3' UTR | 0.04 | up |
| chr5 | 69320248 | 69320459 | 212 | ENSG00000183323 | processed_transcript | CCDC125 | 5' UTR | 0.04 | up |
| chr8 | 143867543 | 143867963 | 421 | ENSG00000261150 | protein_coding | EPPK1 | Exon | 0.04 | up |
| chr19 | 12272901 | 12273380 | 480 | ENSG00000197857 | nonsense_mediated_decay | ZNF44 | Exon | 0.04 | up |
| chr22 | 41694066 | 41694336 | 271 | ENSG00000184208 | transcribed_unitary_pseudogene | C22orf46 | Exon | 0.04 | up |
| chr20 | 1377505 | 1377716 | 212 | ENSG00000229728 | lncRNA | AL136531 | Exon | 0.04 | up |
| chr11 | 106098161 | 106098400 | 240 | ENSG00000149313 | retained_intron | AASDHPPT | 3' UTR | 0.04 | down |
| chr2 | 106806367 | 106806578 | 212 | ENSG00000238250 | lncRNA | ST6GAL2-IT1 | 3' UTR | 0.04 | down |
| chr11 | 18278846 | 18279416 | 571 | ENSG00000110756 | protein_coding | HPS5 | 3' UTR | 0.04 | down |
| X | 154886378 | 154886792 | 415 | ENSG00000277203 | protein_coding | F8A1 | Exon | 0.04 | down |
| chr17 | 4731515 | 4732071 | 557 | ENSG00000161920 | protein_coding | MED11 | 3' UTR | 0.04 | down |
| chr12 | 100059100 | 100059866 | 767 | ENSG00000111647 | retained_intron | UHRF1BP1L | Exon | 0.04 | down |
| chr17 | 46548608 | 46548878 | 271 | ENSG00000238083 | retained_intron | LRRC37A2 | Exon | 0.04 | down |
| chr4 | 5811734 | 5812243 | 510 | ENSG00000072840 | processed_transcript | EVC | 3' UTR | 0.04 | down |
| chr15 | 44405398 | 44414338 | 8941 | ENSG00000166734 | processed_transcript | GOLM2 | Exon | 0.04 | down |
| chr16 | 46696168 | 46697945 | 1778 | ENSG00000091651 | retained_intron | ORC6 | 3' UTR | 0.04 | down |
| chr10 | 102854271 | 102869821 | 15551 | ENSG00000166275 | retained_intron | BORCS7 | 3' UTR | 0.04 | down |
| chr17 | 67893413 | 67911993 | 18581 | ENSG00000171634 | retained_intron | BPTF | 3' UTR | 0.04 | down |
| chr19 | 3762681 | 3765208 | 2528 | ENSG00000183617 | protein_coding | MRPL54 | 5' UTR | 0.04 | down |
| chr8 | 1958011 | 1958641 | 631 | ENSG00000253696 | lncRNA | KBTBD11-OT1 | 3' UTR | 0.04 | down |
| chr15 | 64700572 | 64703251 | 2680 | ENSG00000180304 | protein_coding | OAZ2 | 5' UTR | 0.04 | down |
| chr19 | 47382014 | 47382375 | 362 | ENSG00000134815 | nonsense_mediated_decay | DHX34 | 3' UTR | 0.04 | up |
| chr14 | 67023652 | 67058805 | 35154 | ENSG00000171723 | retained_intron | GPHN | Exon | 0.04 | up |
| chr2 | 74494198 | 74494529 | 332 | ENSG00000115282 | protein_coding | TTC31 | 3' UTR | 0.04 | up |
| chr2 | 25143773 | 25145171 | 1399 | ENSG00000084710 | protein_coding | EFR3B | 3' UTR | 0.04 | up |
| chr15 | 82826761 | 82830994 | 4234 | ENSG00000156232 | retained_intron | WHAMM | Exon | 0.04 | up |
| chr5 | 139880963 | 139904363 | 23401 | ENSG00000158458 | processed_transcript | NRG2 | Exon | 0.04 | up |
| X | 132217012 | 132217341 | 330 | ENSG00000232160 | lncRNA | RAP2C-AS1 | 5' UTR | 0.04 | up |
| chr4 | 102631608 | 102633267 | 1660 | ENSG00000109323 | retained_intron | MANBA | 3' UTR | 0.04 | up |
| chr8 | 73046865 | 73047434 | 570 | ENSG00000253636 | lncRNA | AC022893 | 3' UTR | 0.04 | up |
| chr12 | 4352293 | 4352923 | 631 | ENSG00000078237 | processed_transcript | TIGAR | 3' UTR | 0.04 | up |
| chr1 | 151424194 | 151424375 | 182 | ENSG00000143442 | retained_intron | POGZ | 3' UTR | 0.04 | up |
| chr17 | 75784844 | 75785114 | 271 | ENSG00000132478 | nonsense_mediated_decay | UNK | Exon | 0.04 | up |
| chr5 | 112864460 | 112864881 | 422 | ENSG00000153037 | retained_intron | SRP19 | 3' UTR | 0.04 | up |
| chr9 | 129110754 | 129111189 | 436 | ENSG00000095321 | protein_coding | CRAT | 5' UTR | 0.04 | up |
| chr3 | 47586434 | 47586584 | 151 | ENSG00000114646 | protein_coding | CSPG5 | 3' UTR | 0.04 | up |
| chr19 | 55635488 | 55641104 | 5617 | ENSG00000171425 | protein_coding | ZNF581 | 5' UTR | 0.04 | up |
| chr7 | 21908204 | 21911703 | 3500 | ENSG00000164649 | retained_intron | CDCA7L | Exon | 0.04 | up |
| chr21 | 34101046 | 34101286 | 241 | ENSG00000243927 | retained_intron | MRPS6 | 3' UTR | 0.04 | up |
| chr16 | 22483128 | 22483308 | 181 | ENSG00000237296 | processed_transcript | SMG1P1 | Exon | 0.04 | up |
| chr21 | 6119815 | 6121205 | 1391 | ENSG00000275993 | retained_intron | SIK1B | Exon | 0.04 | up |
| chr11 | 64994452 | 64996928 | 2477 | ENSG00000168062 | protein_coding | BATF2 | 5' UTR | 0.04 | up |
| chr4 | 158706761 | 158708665 | 1905 | ENSG00000271817 | snoRNA | U3 | 3' UTR | 0.04 | up |
| X | 153738175 | 153738382 | 208 | ENSG00000185825 | protein_coding | BCAP31 | Exon | 0.04 | up |
| chr6 | 16301330 | 16301510 | 181 | ENSG00000124788 | protein_coding | ATXN1 | 3' UTR | 0.04 | up |
| chr9 | 130581202 | 130581472 | 271 | ENSG00000107164 | processed_transcript | FUBP3 | 3' UTR | 0.04 | down |
| chr1 | 36172048 | 36172139 | 92 | ENSG00000116871 | retained_intron | MAP7D1 | Exon | 0.04 | down |
| X | 123614131 | 123619672 | 5542 | ENSG00000125676 | processed_transcript | THOC2 | 3' UTR | 0.04 | down |
| chr3 | 40487758 | 40488119 | 362 | ENSG00000177873 | protein_coding | ZNF619 | 3' UTR | 0.04 | down |
| chr2 | 240563913 | 240564481 | 569 | ENSG00000188542 | processed_transcript | DUSP28 | 3' UTR | 0.04 | down |
| chr2 | 55667902 | 55679778 | 11877 | ENSG00000138035 | nonsense_mediated_decay | PNPT1 | 3' UTR | 0.04 | down |
| chr5 | 141187160 | 141187578 | 419 | ENSG00000177839 | protein_coding | PCDHB9 | 5' UTR | 0.04 | down |
| chr14 | 20455609 | 20455819 | 211 | ENSG00000100823 | protein_coding | APEX1 | 5' UTR | 0.04 | down |
| X | 56991394 | 56992713 | 1320 | ENSG00000204271 | nonsense_mediated_decay | SPIN3 | 3' UTR | 0.04 | down |
| chr17 | 3924648 | 3925248 | 601 | ENSG00000074370 | retained_intron | ATP2A3 | 3' UTR | 0.04 | down |
| chr7 | 90414523 | 90414734 | 212 | ENSG00000157224 | processed_transcript | CLDN12 | 3' UTR | 0.04 | down |
| chr15 | 31374848 | 31375059 | 212 | ENSG00000169926 | processed_transcript | KLF13 | 3' UTR | 0.04 | down |
| chr3 | 101594315 | 101594465 | 151 | ENSG00000081154 | retained_intron | PCNP | 3' UTR | 0.04 | down |
| chr11 | 66520818 | 66521237 | 420 | ENSG00000174483 | retained_intron | BBS1 | Exon | 0.04 | down |
| chr16 | 89873599 | 89873720 | 122 | ENSG00000141002 | protein_coding | TCF25 | 5' UTR | 0.04 | down |
| chr2 | 108685930 | 108686409 | 480 | ENSG00000169756 | protein_coding | LIMS1 | 3' UTR | 0.04 | down |
| chr11 | 119028485 | 119028546 | 62 | ENSG00000137700 | retained_intron | SLC37A4 | Exon | 0.04 | down |
| chr5 | 107427210 | 107506191 | 78982 | ENSG00000184349 | protein_coding | EFNA5 | Exon | 0.04 | down |
| chr14 | 24430803 | 24430954 | 152 | ENSG00000139899 | protein_coding | CBLN3 | 5' UTR | 0.04 | down |
| chr18 | 13730629 | 13740187 | 9559 | ENSG00000101654 | protein_coding | RNMT | 5' UTR | 0.04 | down |
| chr8 | 22114628 | 22115048 | 421 | ENSG00000168453 | retained_intron | HR | 3' UTR | 0.04 | down |
| X | 41230591 | 41232951 | 2361 | ENSG00000124486 | retained_intron | USP9X | 3' UTR | 0.04 | down |
| chr7 | 2607182 | 2607333 | 152 | ENSG00000106012 | processed_transcript | IQCE | 3' UTR | 0.04 | up |
| chr9 | 98255228 | 98255559 | 332 | ENSG00000095383 | protein_coding | TBC1D2 | 5' UTR | 0.04 | up |
| chr1 | 169477510 | 169485585 | 8076 | ENSG00000117479 | protein_coding | SLC19A2 | Exon | 0.04 | up |
| chr8 | 79766627 | 79766868 | 242 | ENSG00000164683 | processed_transcript | HEY1 | 3' UTR | 0.04 | up |
| chr15 | 74481064 | 74481304 | 241 | ENSG00000260103 | transcribed_processed_pseudogene | AC012435 | Exon | 0.04 | up |
| chr11 | 108299743 | 108302869 | 3127 | ENSG00000149311 | nonsense_mediated_decay | ATM | 3' UTR | 0.04 | up |
| chr3 | 111721595 | 111722853 | 1259 | ENSG00000240891 | processed_transcript | PLCXD2 | 3' UTR | 0.04 | up |
| chr20 | 2657972 | 2658393 | 422 | ENSG00000101361 | processed_transcript | NOP56 | 3' UTR | 0.04 | up |
| chr10 | 13209185 | 13209906 | 722 | ENSG00000272055 | snRNA | RNU6-6P | 3' UTR | 0.04 | up |
| chr7 | 94909898 | 94910618 | 721 | ENSG00000158528 | protein_coding | PPP1R9A | 5' UTR | 0.04 | up |
| chr20 | 5557995 | 5561500 | 3506 | ENSG00000125772 | processed_transcript | GPCPD1 | 3' UTR | 0.04 | up |
| chr1 | 113913366 | 113913816 | 451 | ENSG00000118655 | nonsense_mediated_decay | DCLRE1B | 3' UTR | 0.04 | up |
| chr21 | 46431324 | 46432062 | 739 | ENSG00000160299 | processed_transcript | PCNT | Exon | 0.04 | up |
| chr17 | 15562213 | 15562781 | 569 | ENSG00000175106 | protein_coding | TVP23C | 5' UTR | 0.04 | up |
| chr1 | 15566121 | 15566272 | 152 | ENSG00000116138 | processed_transcript | DNAJC16 | 3' UTR | 0.04 | down |
| chr1 | 232982832 | 232983043 | 212 | ENSG00000135778 | processed_transcript | NTPCR | 3' UTR | 0.04 | down |
| chr20 | 2482685 | 2482955 | 271 | ENSG00000088876 | retained_intron | ZNF343 | 3' UTR | 0.04 | down |
| chr11 | 2921552 | 2921703 | 152 | ENSG00000110628 | retained_intron | SLC22A18 | Exon | 0.04 | down |
| X | 71254906 | 71255116 | 211 | ENSG00000147130 | protein_coding | ZMYM3 | 5' UTR | 0.04 | down |
| chr20 | 49891972 | 49892242 | 271 | ENSG00000197818 | processed_transcript | SLC9A8 | 3' UTR | 0.04 | down |
| chr12 | 68759200 | 68760181 | 982 | ENSG00000175782 | processed_transcript | SLC35E3 | 3' UTR | 0.04 | down |
| chr3 | 51974272 | 51974422 | 151 | ENSG00000114779 | retained_intron | ABHD14B | 5' UTR | 0.04 | down |
| chr22 | 42509053 | 42510814 | 1762 | ENSG00000172250 | retained_intron | SERHL | Exon | 0.04 | down |
| chr19 | 57443361 | 57444379 | 1019 | ENSG00000276449 | lncRNA | AC004076 | 5' UTR | 0.04 | down |
| chr7 | 73829157 | 73829636 | 480 | ENSG00000189143 | protein_coding | CLDN4 | 5' UTR | 0.04 | down |
| chr12 | 71697493 | 71699218 | 1726 | ENSG00000139291 | protein_coding | TMEM19 | 3' UTR | 0.04 | down |
| chr11 | 62153019 | 62153169 | 151 | ENSG00000149503 | processed_transcript | INCENP | 3' UTR | 0.04 | down |
| chr11 | 64823560 | 64824430 | 871 | ENSG00000171219 | processed_transcript | CDC42BPG | 3' UTR | 0.04 | down |
| chr4 | 185399626 | 185399776 | 151 | ENSG00000109771 | protein_coding | LRP2BP | 3' UTR | 0.04 | down |
| chr22 | 23263512 | 23264229 | 718 | ENSG00000230701 | processed_pseudogene | FBXW4P1 | Exon | 0.04 | down |
| chr1 | 27350458 | 27350758 | 301 | ENSG00000142765 | retained_intron | SYTL1 | Exon | 0.04 | down |
| chr1 | 84490390 | 84495955 | 5566 | ENSG00000122432 | processed_transcript | SPATA1 | Exon | 0.04 | down |
| chr14 | 96555938 | 96562622 | 6685 | ENSG00000090060 | retained_intron | PAPOLA | 3' UTR | 0.04 | down |
| chr2 | 130347579 | 130347848 | 270 | ENSG00000136718 | retained_intron | IMP4 | 3' UTR | 0.04 | down |
| chr11 | 19711731 | 19712235 | 505 | ENSG00000270607 | lncRNA | AC009549 | Exon | 0.04 | up |
| chr20 | 34776321 | 34792458 | 16138 | ENSG00000198646 | protein_coding | NCOA6 | 5' UTR | 0.04 | up |
| chr17 | 7861153 | 7861811 | 659 | ENSG00000280046 | TEC | AC104581 | 3' UTR | 0.04 | up |
| chr15 | 77113601 | 77114620 | 1020 | ENSG00000140391 | protein_coding | TSPAN3 | 3' UTR | 0.04 | up |
| chr11 | 62740004 | 62740293 | 290 | ENSG00000162222 | retained_intron | TTC9C | 3' UTR | 0.04 | up |
| chr14 | 93183209 | 93183418 | 210 | ENSG00000165943 | protein_coding | MOAP1 | Exon | 0.04 | up |
| chr11 | 8715815 | 8717966 | 2152 | ENSG00000166444 | retained_intron | DENND2B | 5' UTR | 0.04 | up |
| chr12 | 48965638 | 48966266 | 629 | ENSG00000169884 | retained_intron | WNT10B | 3' UTR | 0.04 | up |
| chr10 | 101060205 | 101060739 | 535 | ENSG00000273162 | lncRNA | AL133215 | Exon | 0.04 | up |
| chr1 | 25243989 | 25244200 | 212 | ENSG00000117616 | processed_transcript | RSRP1 | 3' UTR | 0.04 | up |
| chr11 | 77813680 | 77820777 | 7098 | ENSG00000048649 | protein_coding | RSF1 | 5' UTR | 0.04 | up |
| X | 84105794 | 84106433 | 640 | ENSG00000072133 | processed_transcript | RPS6KA6 | Exon | 0.04 | up |
| chr12 | 106106437 | 106138745 | 32309 | ENSG00000074590 | protein_coding | NUAK1 | 5' UTR | 0.04 | up |
| chr8 | 78572921 | 78598436 | 25516 | ENSG00000171033 | protein_coding | PKIA | 5' UTR | 0.04 | up |
| chr6 | 28325904 | 28327421 | 1518 | ENSG00000235109 | processed_transcript | ZSCAN31 | 3' UTR | 0.04 | up |
| chr18 | 74158380 | 74158799 | 420 | ENSG00000075336 | protein_coding | TIMM21 | 3' UTR | 0.04 | up |
| chr2 | 111344145 | 111346848 | 2704 | ENSG00000172965 | lncRNA | MIR4435-2HG | Exon | 0.04 | up |
| chr1 | 10660464 | 10660645 | 182 | ENSG00000130940 | retained_intron | CASZ1 | Exon | 0.04 | up |
| chr14 | 54902586 | 54902677 | 92 | ENSG00000131979 | protein_coding | GCH1 | 5' UTR | 0.04 | up |
| chr3 | 126611046 | 126615448 | 4403 | ENSG00000197763 | processed_transcript | TXNRD3 | Exon | 0.04 | down |
| chr13 | 51251749 | 51252228 | 480 | ENSG00000150510 | protein_coding | FAM124A | Exon | 0.04 | down |
| chr7 | 158692870 | 158693433 | 564 | ENSG00000146918 | retained_intron | NCAPG2 | Exon | 0.04 | down |
| chr19 | 42156959 | 42157227 | 269 | ENSG00000259436 | lncRNA | AC010247 | Exon | 0.04 | down |
| chr13 | 37024667 | 37025270 | 604 | ENSG00000102710 | retained_intron | SUPT20H | Exon | 0.04 | down |
| chr16 | 1641156 | 1655245 | 14090 | ENSG00000007545 | retained_intron | CRAMP1 | 3' UTR | 0.04 | down |
| chr22 | 35287406 | 35288371 | 966 | ENSG00000100281 | retained_intron | HMGXB4 | 3' UTR | 0.04 | down |
| chr13 | 45008837 | 45028036 | 19200 | ENSG00000133114 | processed_transcript | GPALPP1 | 3' UTR | 0.04 | down |
| chr2 | 68655292 | 68655862 | 571 | ENSG00000169621 | protein_coding | APLF | 3' UTR | 0.04 | down |
| chr6 | 37257890 | 37282274 | 24385 | ENSG00000172738 | protein_coding | TMEM217 | 5' UTR | 0.04 | up |
| chr1 | 156738143 | 156738377 | 235 | ENSG00000143314 | processed_transcript | MRPL24 | Exon | 0.04 | up |
| chr19 | 57357800 | 57358580 | 781 | ENSG00000152433 | nonsense_mediated_decay | ZNF547 | 3' UTR | 0.04 | up |
| chr5 | 173890742 | 173891405 | 664 | ENSG00000113742 | protein_coding | CPEB4 | Exon | 0.04 | up |
| chr15 | 55340451 | 55355553 | 15103 | ENSG00000069943 | nonsense_mediated_decay | PIGB | 3' UTR | 0.04 | up |
| chr15 | 58771550 | 58771970 | 421 | ENSG00000128923 | protein_coding | MINDY2 | Exon | 0.04 | up |
| chr1 | 204549871 | 204550082 | 212 | ENSG00000198625 | protein_coding | MDM4 | 3' UTR | 0.04 | up |
| chr7 | 102566712 | 102566862 | 151 | ENSG00000168255 | processed_transcript | POLR2J3 | 3' UTR | 0.04 | up |
| chr14 | 73963320 | 73963651 | 332 | ENSG00000187097 | protein_coding | ENTPD5 | 3' UTR | 0.04 | up |
| chr18 | 24449899 | 24450934 | 1036 | ENSG00000154059 | nonsense_mediated_decay | IMPACT | 3' UTR | 0.04 | up |
| chr6 | 13604648 | 13604888 | 241 | ENSG00000225921 | protein_coding | NOL7 | 3' UTR | 0.04 | up |
| chr3 | 197042709 | 197042980 | 272 | ENSG00000163975 | protein_coding | MELTF | 3' UTR | 0.04 | down |
| chr22 | 42278215 | 42278605 | 391 | ENSG00000182057 | lncRNA | OGFRP1 | Exon | 0.04 | down |
| X | 40064501 | 40068013 | 3513 | ENSG00000183337 | protein_coding | BCOR | Exon | 0.04 | down |
| chr7 | 94527170 | 94533685 | 6516 | ENSG00000127995 | nonsense_mediated_decay | CASD1 | 3' UTR | 0.04 | down |
| chr4 | 1942386 | 1942627 | 242 | ENSG00000109685 | processed_transcript | NSD2 | 3' UTR | 0.04 | down |
| chr7 | 27173975 | 27174306 | 332 | ENSG00000253293 | protein_coding | HOXA10 | Exon | 0.04 | down |
| chr4 | 55428861 | 55429461 | 601 | ENSG00000134852 | retained_intron | CLOCK | 3' UTR | 0.04 | down |
| chr11 | 64300855 | 64303828 | 2974 | ENSG00000219435 | retained_intron | CATSPERZ | Exon | 0.04 | down |
| chr7 | 128739381 | 128747681 | 8301 | ENSG00000128595 | protein_coding | CALU | 5' UTR | 0.04 | down |
| chr16 | 22324549 | 22324640 | 92 | ENSG00000058600 | retained_intron | POLR3E | 3' UTR | 0.04 | down |
| chr11 | 43673119 | 43815486 | 142368 | ENSG00000149084 | nonsense_mediated_decay | HSD17B12 | 3' UTR | 0.04 | down |
| chr21 | 14373414 | 14375798 | 2385 | ENSG00000155304 | protein_coding | HSPA13 | 3' UTR | 0.04 | down |
| chr17 | 44396658 | 44397108 | 451 | ENSG00000005961 | protein_coding | ITGA2B | 3' UTR | 0.04 | down |
| chr7 | 29920971 | 29921301 | 331 | ENSG00000136193 | protein_coding | SCRN1 | 3' UTR | 0.04 | up |
| chr2 | 120220080 | 120220350 | 271 | ENSG00000226479 | protein_coding | TMEM185B | 3' UTR | 0.04 | up |
| chr6 | 128089774 | 128184604 | 94831 | ENSG00000152894 | processed_transcript | PTPRK | 3' UTR | 0.04 | up |
| chr15 | 89903311 | 89903857 | 547 | ENSG00000250021 | processed_transcript | ARPIN-AP3S2 | Exon | 0.04 | up |
| chr1 | 243504296 | 243505077 | 782 | ENSG00000117020 | processed_transcript | AKT3 | 3' UTR | 0.04 | up |
| chr1 | 178008137 | 178022667 | 14531 | ENSG00000254154 | lncRNA | CRYZL2P-SEC16B | Exon | 0.04 | up |
| chr17 | 83084945 | 83085303 | 359 | ENSG00000176845 | protein_coding | METRNL | 5' UTR | 0.04 | up |
| chr20 | 58991605 | 58991786 | 182 | ENSG00000101158 | processed_transcript | NELFCD | Exon | 0.04 | up |
| chr19 | 24046230 | 24047773 | 1544 | ENSG00000268362 | lncRNA | AC092279 | 5' UTR | 0.04 | up |
| chr5 | 55956053 | 55960420 | 4368 | ENSG00000134352 | retained_intron | IL6ST | 3' UTR | 0.04 | up |
| chr10 | 12237228 | 12237513 | 286 | ENSG00000151465 | protein_coding | CDC123 | Exon | 0.04 | up |
| chr2 | 9556365 | 9556717 | 353 | ENSG00000271855 | lncRNA | AC073195 | Exon | 0.04 | up |
| chr19 | 57552513 | 57553023 | 511 | ENSG00000121406 | protein_coding | ZNF549 | 3' UTR | 0.04 | up |
| chr2 | 173811104 | 173811392 | 289 | ENSG00000270460 | lncRNA | AC106900 | Exon | 0.04 | up |
| chr8 | 143867539 | 143867960 | 422 | ENSG00000261150 | protein_coding | EPPK1 | Exon | 0.04 | up |
| chr1 | 6455038 | 6455368 | 331 | ENSG00000187017 | protein_coding | ESPN | Exon | 0.04 | down |
| chr11 | 90211259 | 90211440 | 182 | ENSG00000110172 | protein_coding | CHORDC1 | 3' UTR | 0.04 | down |
| chr19 | 7525372 | 7525552 | 181 | ENSG00000090674 | retained_intron | MCOLN1 | Exon | 0.04 | down |
| chr1 | 92636257 | 92693848 | 57592 | ENSG00000067208 | protein_coding | EVI5 | Exon | 0.04 | down |
| chr7 | 35890706 | 35898517 | 7812 | ENSG00000122545 | protein_coding | SEPTIN7 | 3' UTR | 0.04 | down |
| chr3 | 55467301 | 55467601 | 301 | ENSG00000114251 | processed_transcript | WNT5A | 3' UTR | 0.04 | down |
| chr10 | 31521069 | 31523934 | 2866 | ENSG00000223834 | lncRNA | AL161935 | 3' UTR | 0.04 | down |
| chr9 | 136844474 | 136844773 | 300 | ENSG00000232434 | protein_coding | AJM1 | Exon | 0.04 | down |
| chr3 | 100455811 | 100456259 | 449 | ENSG00000181458 | protein_coding | TMEM45A | 3' UTR | 0.04 | down |
| chr2 | 223756989 | 223757468 | 480 | ENSG00000152056 | protein_coding | AP1S3 | 3' UTR | 0.04 | down |
| chr1 | 32386767 | 32393713 | 6947 | ENSG00000160058 | retained_intron | BSDC1 | Exon | 0.04 | down |
| chr16 | 15594596 | 15595406 | 811 | ENSG00000166783 | retained_intron | MARF1 | 3' UTR | 0.04 | down |
| chr6 | 82240675 | 82247754 | 7080 | ENSG00000005700 | protein_coding | IBTK | 5' UTR | 0.04 | down |
| chr17 | 58312184 | 58312694 | 511 | ENSG00000005379 | protein_coding | TSPOAP1 | Exon | 0.04 | down |
| chr1 | 147630101 | 147642345 | 12245 | ENSG00000162836 | retained_intron | ACP6 | 3' UTR | 0.04 | down |
| chr11 | 112082542 | 112083562 | 1021 | ENSG00000204370 | nonsense_mediated_decay | SDHD | 3' UTR | 0.04 | down |
| chr19 | 57191499 | 57191770 | 272 | ENSG00000083844 | protein_coding | ZNF264 | 5' UTR | 0.04 | down |
| chr17 | 31995620 | 31999273 | 3654 | ENSG00000178691 | retained_intron | SUZ12 | 3' UTR | 0.04 | down |
| chr16 | 28723127 | 28726319 | 3193 | ENSG00000184110 | retained_intron | EIF3C | Exon | 0.04 | down |
| chr11 | 77332760 | 77336279 | 3520 | ENSG00000149269 | retained_intron | PAK1 | 3' UTR | 0.04 | down |
| chr1 | 38864370 | 38867595 | 3226 | ENSG00000116954 | protein_coding | RRAGC | 3' UTR | 0.04 | down |
| chr15 | 66708218 | 66716427 | 8210 | ENSG00000137834 | nonsense_mediated_decay | SMAD6 | Exon | 0.04 | up |
| chr16 | 731714 | 731835 | 122 | ENSG00000103245 | retained_intron | CIAO3 | Exon | 0.04 | up |
| chr6 | 33457156 | 33457246 | 91 | ENSG00000213588 | protein_coding | ZBTB9 | 3' UTR | 0.04 | up |
| chr3 | 179203544 | 179204588 | 1045 | ENSG00000121879 | retained_intron | PIK3CA | Exon | 0.04 | up |
| chr5 | 137953448 | 137954419 | 972 | ENSG00000031003 | retained_intron | FAM13B | Exon | 0.04 | up |
| chr7 | 152315137 | 152358663 | 43527 | ENSG00000055609 | retained_intron | KMT2C | Exon | 0.04 | up |
| chr11 | 74491910 | 74493220 | 1311 | ENSG00000175536 | protein_coding | LIPT2 | 3' UTR | 0.04 | up |
| chr9 | 35399680 | 35400397 | 718 | ENSG00000198722 | protein_coding | UNC13B | 3' UTR | 0.04 | up |
| chr22 | 39091730 | 39092855 | 1126 | ENSG00000225720 | processed_transcript | AL031846 | Exon | 0.04 | up |
| chr10 | 49517111 | 49525080 | 7970 | ENSG00000225830 | processed_transcript | ERCC6 | Exon | 0.04 | up |
| chr1 | 41011726 | 41011787 | 62 | ENSG00000281207 | lncRNA | SLFNL1-AS1 | 3' UTR | 0.04 | up |
| chr14 | 75764718 | 75771793 | 7076 | ENSG00000119685 | protein_coding | TTLL5 | Exon | 0.04 | up |
| chr19 | 29615561 | 29615801 | 241 | ENSG00000105171 | retained_intron | POP4 | 3' UTR | 0.04 | up |
| chr8 | 140520544 | 140520635 | 92 | ENSG00000123908 | processed_transcript | AGO2 | 3' UTR | 0.04 | up |
| chr14 | 44999440 | 45006333 | 6894 | ENSG00000198718 | protein_coding | TOGARAM1 | Exon | 0.04 | up |
| chr12 | 30978515 | 30978665 | 151 | ENSG00000226472 | processed_transcript | AC008013 | Exon | 0.04 | up |
| chr19 | 38329381 | 38329681 | 301 | ENSG00000099338 | protein_coding | CATSPERG | 3' UTR | 0.04 | up |
| chr15 | 40532150 | 40534430 | 2281 | ENSG00000128891 | processed_transcript | CCDC32 | 3' UTR | 0.04 | up |
| chr10 | 112450427 | 112450668 | 242 | ENSG00000151532 | processed_transcript | VTI1A | Exon | 0.04 | up |
| chr15 | 65624572 | 65624813 | 242 | ENSG00000074621 | protein_coding | SLC24A1 | Exon | 0.04 | up |
| chr6 | 75623929 | 75634830 | 10902 | ENSG00000112701 | retained_intron | SENP6 | 3' UTR | 0.04 | up |
| chr2 | 47068265 | 47068474 | 210 | ENSG00000225187 | lncRNA | AC073283 | Exon | 0.03 | down |
| chr2 | 196135471 | 196135771 | 301 | ENSG00000081320 | protein_coding | STK17B | 3' UTR | 0.03 | down |
| chr10 | 101550839 | 101550990 | 152 | ENSG00000166167 | processed_transcript | BTRC | 3' UTR | 0.03 | down |
| chr14 | 92641319 | 92644535 | 3217 | ENSG00000100599 | processed_transcript | RIN3 | Exon | 0.03 | down |
| chr16 | 89595471 | 89595908 | 438 | ENSG00000178773 | protein_coding | CPNE7 | 3' UTR | 0.03 | down |
| chr1 | 52685045 | 52685792 | 748 | ENSG00000162377 | processed_transcript | COA7 | 3' UTR | 0.03 | down |
| chr17 | 59946620 | 59947639 | 1020 | ENSG00000108443 | processed_transcript | RPS6KB1 | 3' UTR | 0.03 | down |
| chr3 | 9476965 | 9477176 | 212 | ENSG00000168137 | retained_intron | SETD5 | 3' UTR | 0.03 | down |
| chr1 | 77811305 | 77811633 | 329 | ENSG00000219201 | processed_pseudogene | AC138392 | Exon | 0.03 | down |
| chr19 | 44665474 | 44666162 | 689 | ENSG00000279095 | TEC | AC243964 | 3' UTR | 0.03 | down |
| chr19 | 57853831 | 57856021 | 2191 | ENSG00000198466 | retained_intron | ZNF587 | Exon | 0.03 | down |
| chr15 | 40936499 | 40938356 | 1858 | ENSG00000128917 | retained_intron | DLL4 | 3' UTR | 0.03 | down |
| chr15 | 44348885 | 44382196 | 33312 | ENSG00000166734 | processed_transcript | GOLM2 | 3' UTR | 0.03 | down |
| chr19 | 43661456 | 43667282 | 5827 | ENSG00000011422 | retained_intron | PLAUR | Exon | 0.03 | down |
| chr12 | 68841521 | 68842929 | 1409 | ENSG00000135679 | processed_transcript | MDM2 | 3' UTR | 0.03 | up |
| chr1 | 23796197 | 23796378 | 182 | ENSG00000117308 | protein_coding | GALE | Exon | 0.03 | up |
| chr6 | 30486441 | 30486531 | 91 | ENSG00000236603 | processed_pseudogene | RANP1 | Exon | 0.03 | up |
| chr4 | 151099653 | 151099774 | 122 | ENSG00000145425 | protein_coding | RPS3A | 5' UTR | 0.03 | up |
| chr7 | 101815993 | 101913170 | 97178 | ENSG00000257923 | protein_coding | CUX1 | 5' UTR | 0.03 | up |
| chr1 | 247099991 | 247101752 | 1762 | ENSG00000188295 | protein_coding | ZNF669 | 3' UTR | 0.03 | up |
| chr14 | 34862104 | 34874843 | 12740 | ENSG00000198604 | nonsense_mediated_decay | BAZ1A | 5' UTR | 0.03 | up |
| chr6 | 151357690 | 151357870 | 181 | ENSG00000131016 | processed_transcript | AKAP12 | 3' UTR | 0.03 | up |
| chr15 | 90206571 | 90206967 | 397 | ENSG00000225193 | transcribed_processed_pseudogene | RPS12P26 | Exon | 0.03 | up |
| chr9 | 128431597 | 128432006 | 410 | ENSG00000273186 | lncRNA | AL359091 | Exon | 0.03 | up |
| chr15 | 25338054 | 25373542 | 35489 | ENSG00000114062 | protein_coding | UBE3A | Exon | 0.03 | up |
| chr5 | 96763207 | 96775183 | 11977 | ENSG00000153113 | retained_intron | CAST | 3' UTR | 0.03 | up |
| chr5 | 140458730 | 140459182 | 453 | ENSG00000131503 | retained_intron | ANKHD1 | 5' UTR | 0.03 | up |
| chr3 | 69959350 | 69965315 | 5966 | ENSG00000187098 | processed_transcript | MITF | 3' UTR | 0.03 | up |
| chr8 | 124313836 | 124314017 | 182 | ENSG00000164983 | protein_coding | TMEM65 | 3' UTR | 0.03 | up |
| chr1 | 1757024 | 1757115 | 92 | ENSG00000008130 | retained_intron | NADK | Exon | 0.03 | up |
| chr5 | 95583000 | 95583151 | 152 | ENSG00000164291 | nonsense_mediated_decay | ARSK | 3' UTR | 0.03 | up |
| chr1 | 206401567 | 206405125 | 3559 | ENSG00000266028 | retained_intron | SRGAP2 | Exon | 0.03 | up |
| chr17 | 58322949 | 58323358 | 410 | ENSG00000005379 | retained_intron | TSPOAP1 | Exon | 0.03 | up |
| chr5 | 179607338 | 179607459 | 122 | ENSG00000176783 | processed_transcript | RUFY1 | Exon | 0.03 | up |
| chr3 | 52221199 | 52221440 | 242 | ENSG00000280003 | TEC | AC097637 | 3' UTR | 0.03 | down |
| chr3 | 52221200 | 52221440 | 241 | ENSG00000280003 | TEC | AC097637 | 3' UTR | 0.03 | down |
| chr17 | 80208790 | 80209151 | 362 | ENSG00000181045 | protein_coding | SLC26A11 | 3' UTR | 0.03 | down |
| chr15 | 99973474 | 99974524 | 1051 | ENSG00000140470 | retained_intron | ADAMTS17 | 3' UTR | 0.03 | down |
| chr2 | 161224707 | 161232771 | 8065 | ENSG00000136560 | protein_coding | TANK | 3' UTR | 0.03 | down |
| chr16 | 11255006 | 11255366 | 361 | ENSG00000185338 | protein_coding | SOCS1 | Exon | 0.03 | down |
| chr8 | 65718615 | 65721906 | 3292 | ENSG00000272155 | lncRNA | AC055822 | 3' UTR | 0.03 | down |
| chr12 | 123758006 | 123758576 | 571 | ENSG00000185344 | retained_intron | ATP6V0A2 | 3' UTR | 0.03 | down |
| chr9 | 113536828 | 113565052 | 28225 | ENSG00000138835 | protein_coding | RGS3 | 3' UTR | 0.03 | down |
| chr3 | 146517067 | 146517247 | 181 | ENSG00000188313 | processed_transcript | PLSCR1 | 3' UTR | 0.03 | down |
| chr16 | 735016 | 735525 | 510 | ENSG00000103253 | processed_transcript | HAGHL | Exon | 0.03 | down |
| chr1 | 10457090 | 10457751 | 662 | ENSG00000160049 | nonsense_mediated_decay | DFFA | 3' UTR | 0.03 | down |
| X | 70420198 | 70420677 | 480 | ENSG00000130055 | protein_coding | GDPD2 | 3' UTR | 0.03 | down |
| chr6 | 124948080 | 124963165 | 15086 | ENSG00000146373 | protein_coding | RNF217 | Exon | 0.03 | down |
| chr15 | 22867440 | 22867621 | 182 | ENSG00000273749 | protein_coding | CYFIP1 | 3' UTR | 0.03 | up |
| chr20 | 49028538 | 49033582 | 5045 | ENSG00000124207 | protein_coding | CSE1L | 3' UTR | 0.03 | up |
| chr1 | 22864903 | 22865114 | 212 | ENSG00000133216 | processed_transcript | EPHB2 | Exon | 0.03 | up |
| X | 72128257 | 72129721 | 1465 | ENSG00000242732 | protein_coding | RTL5 | 3' UTR | 0.03 | up |
| chr5 | 150296819 | 150297595 | 777 | ENSG00000183876 | protein_coding | ARSI | 3' UTR | 0.03 | up |
| chr5 | 134959079 | 134960725 | 1647 | ENSG00000152705 | processed_transcript | CATSPER3 | 3' UTR | 0.03 | up |
| chr10 | 31317588 | 31318637 | 1050 | ENSG00000148516 | retained_intron | ZEB1 | Exon | 0.03 | up |
| chr1 | 32797772 | 32797953 | 182 | ENSG00000134684 | processed_transcript | YARS1 | 3' UTR | 0.03 | up |
| chr6 | 34279738 | 34279979 | 242 | ENSG00000220583 | processed_pseudogene | RPL35P2 | 3' UTR | 0.03 | up |
| chr11 | 65423985 | 65424118 | 134 | ENSG00000277599 | misc_RNA | AP000944 | Exon | 0.03 | up |
| chr11 | 68024897 | 68025288 | 392 | ENSG00000255306 | lncRNA | AC004923 | Exon | 0.03 | up |
| chr12 | 119679992 | 119680159 | 168 | ENSG00000111725 | processed_transcript | PRKAB1 | Exon | 0.03 | up |
| X | 80288907 | 80310116 | 21210 | ENSG00000278530 | transcribed_unitary_pseudogene | CHMP1B2P | Exon | 0.03 | up |
| chr3 | 44916096 | 44916396 | 301 | ENSG00000163812 | processed_transcript | ZDHHC3 | 3' UTR | 0.03 | up |
| chr5 | 180810254 | 180810495 | 242 | ENSG00000131446 | processed_transcript | MGAT1 | Exon | 0.03 | up |
| chr8 | 39136713 | 39151519 | 14807 | ENSG00000197140 | retained_intron | ADAM32 | 5' UTR | 0.03 | up |
| chr17 | 29589461 | 29593964 | 4504 | ENSG00000108262 | protein_coding | GIT1 | 5' UTR | 0.03 | up |
| chr8 | 143429142 | 143429533 | 392 | ENSG00000182759 | processed_transcript | MAFA | 3' UTR | 0.03 | up |
| chr1 | 180093622 | 180094162 | 541 | ENSG00000135837 | protein_coding | CEP350 | Exon | 0.03 | down |
| chr3 | 119582253 | 119586311 | 4059 | ENSG00000144843 | protein_coding | ADPRH | Exon | 0.03 | down |
| chr15 | 25713781 | 25714022 | 242 | ENSG00000206190 | processed_transcript | ATP10A | 3' UTR | 0.03 | down |
| chr4 | 39032653 | 39032862 | 210 | ENSG00000121895 | processed_transcript | TMEM156 | 5' UTR | 0.03 | down |
| chr1 | 9928685 | 9928956 | 272 | ENSG00000162441 | retained_intron | LZIC | 3' UTR | 0.03 | down |
| chr1 | 40281367 | 40290678 | 9312 | ENSG00000084073 | retained_intron | ZMPSTE24 | 3' UTR | 0.03 | down |
| X | 70297166 | 70302057 | 4892 | ENSG00000090889 | processed_transcript | KIF4A | Exon | 0.03 | down |
| chr12 | 62560519 | 62561089 | 571 | ENSG00000061987 | retained_intron | MON2 | 3' UTR | 0.03 | down |
| chr9 | 34371282 | 34371913 | 632 | ENSG00000164976 | protein_coding | MYORG | Exon | 0.03 | down |
| chr3 | 56633121 | 56633751 | 631 | ENSG00000163946 | retained_intron | TASOR | Exon | 0.03 | down |
| chr4 | 38044404 | 38051924 | 7521 | ENSG00000065882 | protein_coding | TBC1D1 | Exon | 0.03 | down |
| chr6 | 160106005 | 160106515 | 511 | ENSG00000197081 | processed_transcript | IGF2R | 3' UTR | 0.03 | down |
| chr7 | 137885436 | 137905748 | 20313 | ENSG00000182158 | protein_coding | CREB3L2 | 3' UTR | 0.03 | up |
| chr8 | 37765718 | 37766372 | 655 | ENSG00000147471 | protein_coding | PLPBP | 3' UTR | 0.03 | up |
| chr3 | 45959395 | 45964351 | 4957 | ENSG00000163820 | protein_coding | FYCO1 | Exon | 0.03 | up |
| chr19 | 50278267 | 50281704 | 3438 | ENSG00000105357 | retained_intron | MYH14 | 5' UTR | 0.03 | up |
| chr11 | 70487300 | 70487511 | 212 | ENSG00000162105 | protein_coding | SHANK2 | Exon | 0.03 | up |
| chr15 | 77109285 | 77110125 | 841 | ENSG00000140391 | protein_coding | TSPAN3 | 3' UTR | 0.03 | up |
| chr16 | 87392432 | 87392583 | 152 | ENSG00000140941 | retained_intron | MAP1LC3B | Exon | 0.03 | down |
| chr7 | 11102652 | 11103132 | 481 | ENSG00000106443 | processed_transcript | PHF14 | 3' UTR | 0.03 | down |
| chr13 | 102862170 | 102862829 | 660 | ENSG00000134899 | processed_transcript | ERCC5 | 3' UTR | 0.03 | down |
| chr13 | 102862183 | 102862842 | 660 | ENSG00000134899 | processed_transcript | ERCC5 | 3' UTR | 0.03 | down |
| chr4 | 41650398 | 41661449 | 11052 | ENSG00000064042 | processed_transcript | LIMCH1 | Exon | 0.03 | down |
| chr3 | 172786565 | 172802863 | 16299 | ENSG00000114346 | protein_coding | ECT2 | Exon | 0.03 | down |
| chr8 | 127855164 | 127859902 | 4739 | ENSG00000249859 | lncRNA | PVT1 | Exon | 0.03 | down |
| chr2 | 230059188 | 230067160 | 7973 | ENSG00000199400 | misc_RNA | RNY4P19 | 5' UTR | 0.03 | down |
| chr17 | 44734099 | 44741428 | 7330 | ENSG00000161692 | retained_intron | DBF4B | 3' UTR | 0.03 | down |
| chr15 | 100573646 | 100576646 | 3001 | ENSG00000140471 | nonsense_mediated_decay | LINS1 | Exon | 0.03 | down |
| chr12 | 47745369 | 47745670 | 302 | ENSG00000079337 | retained_intron | RAPGEF3 | 3' UTR | 0.03 | down |
| chr11 | 119232552 | 119271801 | 39250 | ENSG00000110395 | protein_coding | CBL | Exon | 0.03 | down |
| chr20 | 1565063 | 1565423 | 361 | ENSG00000101307 | protein_coding | SIRPB1 | 3' UTR | 0.03 | down |
| chr1 | 215620164 | 215620881 | 718 | ENSG00000136636 | processed_transcript | KCTD3 | 3' UTR | 0.03 | down |
| chr17 | 2477725 | 2511891 | 34167 | ENSG00000127804 | protein_coding | METTL16 | 5' UTR | 0.03 | down |
| chr1 | 205241594 | 205241804 | 211 | ENSG00000133069 | processed_transcript | TMCC2 | Exon | 0.03 | down |
| chr15 | 100881128 | 100881399 | 272 | ENSG00000184254 | protein_coding | ALDH1A3 | 3' UTR | 0.03 | down |
| chr3 | 170129135 | 170129436 | 302 | ENSG00000173889 | protein_coding | PHC3 | Exon | 0.03 | down |
| chr2 | 118107153 | 118109052 | 1900 | ENSG00000125629 | processed_transcript | INSIG2 | 3' UTR | 0.03 | up |
| chr17 | 47975226 | 47975613 | 388 | ENSG00000108465 | retained_intron | CDK5RAP3 | 3' UTR | 0.03 | up |
| chr1 | 52033975 | 52055191 | 21217 | ENSG00000117862 | protein_coding | TXNDC12 | 3' UTR | 0.03 | up |
| chr3 | 15635645 | 15642164 | 6520 | ENSG00000169814 | protein_coding | BTD | 3' UTR | 0.03 | up |
| chr14 | 91458207 | 91461406 | 3200 | ENSG00000100796 | retained_intron | PPP4R3A | 3' UTR | 0.03 | up |
| chr10 | 67887447 | 67891431 | 3985 | ENSG00000096717 | protein_coding | SIRT1 | 5' UTR | 0.03 | up |
| chr3 | 197050627 | 197065522 | 14896 | ENSG00000075711 | retained_intron | DLG1 | 3' UTR | 0.03 | up |
| chr11 | 108250884 | 108253905 | 3022 | ENSG00000149311 | processed_transcript | ATM | Exon | 0.03 | up |
| chr16 | 68311003 | 68311184 | 182 | ENSG00000132600 | protein_coding | PRMT7 | 5' UTR | 0.03 | up |
| chr2 | 44321130 | 44321538 | 409 | ENSG00000138078 | protein_coding | PREPL | 3' UTR | 0.03 | up |
| chr9 | 62532453 | 62532682 | 230 | ENSG00000212951 | unprocessed_pseudogene | BX005266 | Exon | 0.03 | up |
| chr5 | 140807485 | 140808415 | 931 | ENSG00000204967 | protein_coding | PCDHA4 | Exon | 0.03 | up |
| chr6 | 34279703 | 34280003 | 301 | ENSG00000225339 | retained_intron | AL354740 | Exon | 0.03 | up |
| chr15 | 55317272 | 55318923 | 1652 | ENSG00000225973 | protein_coding | PIGBOS1 | 3' UTR | 0.03 | up |
| chr5 | 55160286 | 55160526 | 241 | ENSG00000164294 | retained_intron | GPX8 | Exon | 0.03 | up |
| chr6 | 73241313 | 73241523 | 211 | ENSG00000135314 | protein_coding | KHDC1 | 3' UTR | 0.03 | up |
| chr12 | 29171639 | 29223822 | 52184 | ENSG00000064763 | protein_coding | FAR2 | 5' UTR | 0.03 | up |
| chr12 | 106978387 | 106978778 | 392 | ENSG00000151135 | processed_transcript | TMEM263 | Exon | 0.03 | up |
| chr9 | 67249116 | 67249176 | 61 | ENSG00000276386 | unprocessed_pseudogene | CNTNAP3P2 | Exon | 0.03 | up |
| chr13 | 67224862 | 67225193 | 332 | ENSG00000184226 | protein_coding | PCDH9 | 3' UTR | 0.03 | down |
| chr21 | 43808949 | 43809488 | 540 | ENSG00000215458 | lncRNA | AATBC | Exon | 0.03 | down |
| chr17 | 57078381 | 57079181 | 801 | ENSG00000263004 | lncRNA | AC007114 | Exon | 0.03 | down |
| chr7 | 157004900 | 157005798 | 899 | ENSG00000130675 | protein_coding | MNX1 | 3' UTR | 0.03 | down |
| chr14 | 24315818 | 24316298 | 481 | ENSG00000213903 | protein_coding | LTB4R | 5' UTR | 0.03 | down |
| chr10 | 68646213 | 68651862 | 5650 | ENSG00000213025 | processed_pseudogene | COX20P1 | Exon | 0.03 | down |
| chr16 | 11721622 | 11730716 | 9095 | ENSG00000153066 | nonsense_mediated_decay | TXNDC11 | Exon | 0.03 | down |
| chr19 | 2728429 | 2729327 | 899 | ENSG00000261342 | lncRNA | AC006538 | Exon | 0.03 | down |
| chr13 | 42302763 | 42303663 | 901 | ENSG00000023516 | protein_coding | AKAP11 | Exon | 0.03 | down |
| chr19 | 55601760 | 55601970 | 211 | ENSG00000171443 | protein_coding | ZNF524 | 5' UTR | 0.03 | down |
| chr11 | 134153554 | 134156889 | 3336 | ENSG00000151503 | processed_transcript | NCAPD3 | Exon | 0.03 | down |
| chr15 | 50934663 | 50950097 | 15435 | ENSG00000259378 | processed_pseudogene | DCAF13P3 | 3' UTR | 0.03 | down |
| chr7 | 90403461 | 90513391 | 109931 | ENSG00000058091 | protein_coding | CDK14 | 3' UTR | 0.03 | down |
| chr14 | 104821769 | 104822250 | 482 | ENSG00000258701 | lncRNA | LINC00638 | Exon | 0.03 | down |
| chr1 | 150943964 | 150945098 | 1135 | ENSG00000143379 | retained_intron | SETDB1 | 3' UTR | 0.03 | up |
| chr4 | 144995307 | 145033340 | 38034 | ENSG00000164162 | protein_coding | ANAPC10 | 3' UTR | 0.03 | up |
| chr12 | 4352285 | 4352943 | 659 | ENSG00000078237 | processed_transcript | TIGAR | 3' UTR | 0.03 | up |
| chr12 | 132731888 | 132734556 | 2669 | ENSG00000176915 | protein_coding | ANKLE2 | 5' UTR | 0.03 | up |
| chr16 | 84123783 | 84124113 | 331 | ENSG00000103160 | processed_transcript | HSDL1 | 3' UTR | 0.03 | up |
| chr1 | 212051523 | 212051722 | 200 | ENSG00000220749 | processed_pseudogene | RPL21P28 | Exon | 0.03 | up |
| X | 119619361 | 119619511 | 151 | ENSG00000186416 | protein_coding | NKRF | 3' UTR | 0.03 | up |
| chr22 | 31282941 | 31283271 | 331 | ENSG00000100100 | retained_intron | PIK3IP1 | 3' UTR | 0.03 | up |
| chr14 | 23521059 | 23521848 | 790 | ENSG00000136367 | processed_transcript | ZFHX2 | 3' UTR | 0.03 | up |
| chr4 | 8232792 | 8233392 | 601 | ENSG00000125089 | retained_intron | SH3TC1 | 3' UTR | 0.03 | up |
| chr12 | 114679904 | 114680144 | 241 | ENSG00000135111 | retained_intron | TBX3 | Exon | 0.03 | up |
| chr5 | 177476660 | 177476990 | 331 | ENSG00000113758 | protein_coding | DBN1 | Exon | 0.03 | up |
| chr1 | 145978699 | 145978848 | 150 | ENSG00000121851 | retained_intron | POLR3GL | 3' UTR | 0.03 | up |
| X | 3606194 | 3606584 | 391 | ENSG00000207332 | snRNA | RNU6-146P | 3' UTR | 0.03 | up |
| chr8 | 11319827 | 11322763 | 2937 | ENSG00000177710 | protein_coding | SLC35G5 | 3' UTR | 0.03 | down |
| chr2 | 230909852 | 230910419 | 568 | ENSG00000135898 | protein_coding | GPR55 | 3' UTR | 0.03 | down |
| chr11 | 47214643 | 47215003 | 361 | ENSG00000134574 | protein_coding | DDB2 | 5' UTR | 0.03 | down |
| X | 10589890 | 10620511 | 30622 | ENSG00000101871 | protein_coding | MID1 | 5' UTR | 0.03 | down |
| chr17 | 77098282 | 77098793 | 512 | ENSG00000284250 | miRNA | MIR6516 | Exon | 0.03 | down |
| chr3 | 51664502 | 51664803 | 302 | ENSG00000164081 | protein_coding | TEX264 | 3' UTR | 0.03 | down |
| chr6 | 99431787 | 99432323 | 537 | ENSG00000228506 | lncRNA | AL513550 | Exon | 0.03 | down |
| chr7 | 155738079 | 155738260 | 182 | ENSG00000184863 | protein_coding | RBM33 | 3' UTR | 0.03 | down |
| chr4 | 6996333 | 7001283 | 4951 | ENSG00000132405 | protein_coding | TBC1D14 | 5' UTR | 0.03 | down |
| chr6 | 148546098 | 148549234 | 3137 | ENSG00000111961 | retained_intron | SASH1 | 3' UTR | 0.03 | down |
| chr19 | 5823831 | 5824131 | 301 | ENSG00000171119 | protein_coding | NRTN | 5' UTR | 0.03 | up |
| chr1 | 113119338 | 113124738 | 5401 | ENSG00000198799 | processed_transcript | LRIG2 | 3' UTR | 0.03 | up |
| chr12 | 101405826 | 101407125 | 1300 | ENSG00000120805 | protein_coding | ARL1 | 5' UTR | 0.03 | up |
| chr19 | 57419640 | 57421133 | 1494 | ENSG00000186272 | retained_intron | ZNF17 | 3' UTR | 0.03 | up |
| chr7 | 27172661 | 27173376 | 716 | ENSG00000253293 | processed_transcript | HOXA10 | Exon | 0.03 | up |
| chr1 | 241631904 | 241632204 | 301 | ENSG00000203668 | processed_transcript | CHML | 3' UTR | 0.03 | up |
| chr17 | 15642755 | 15642936 | 182 | ENSG00000221926 | protein_coding | TRIM16 | 5' UTR | 0.03 | up |
| chr5 | 140807488 | 140808414 | 927 | ENSG00000204967 | protein_coding | PCDHA4 | Exon | 0.03 | up |
| chr19 | 52290137 | 52291367 | 1231 | ENSG00000198464 | protein_coding | ZNF480 | 3' UTR | 0.03 | up |
| chr19 | 10335883 | 10338769 | 2887 | ENSG00000076662 | protein_coding | ICAM3 | 3' UTR | 0.03 | up |
| chr9 | 15470693 | 15471233 | 541 | ENSG00000164985 | protein_coding | PSIP1 | 3' UTR | 0.03 | up |
| chr5 | 157803699 | 157806055 | 2357 | ENSG00000113282 | retained_intron | CLINT1 | Exon | 0.03 | up |
| chr17 | 82024106 | 82024167 | 62 | ENSG00000169689 | protein_coding | CENPX | 5' UTR | 0.03 | up |
| chr1 | 109275450 | 109275541 | 92 | ENSG00000143126 | processed_transcript | CELSR2 | 3' UTR | 0.03 | up |
| X | 91835569 | 91877205 | 41637 | ENSG00000102290 | protein_coding | PCDH11X | Exon | 0.03 | up |
| chr4 | 77028746 | 77031426 | 2681 | ENSG00000138758 | processed_transcript | SEPTIN11 | 3' UTR | 0.03 | up |
| chr11 | 83180472 | 83180623 | 152 | ENSG00000165494 | retained_intron | PCF11 | Exon | 0.03 | up |
| chr3 | 139444341 | 139444521 | 181 | ENSG00000248932 | lncRNA | AC046134 | Exon | 0.03 | down |
| chr17 | 1711880 | 1712209 | 330 | ENSG00000185561 | protein_coding | TLCD2 | Exon | 0.03 | down |
| chr4 | 99061939 | 99062090 | 152 | ENSG00000164024 | protein_coding | METAP1 | 3' UTR | 0.03 | down |
| chr20 | 5967505 | 5967958 | 454 | ENSG00000231961 | processed_pseudogene | AL035461 | Exon | 0.03 | down |
| chr10 | 102854288 | 102860622 | 6335 | ENSG00000166275 | retained_intron | BORCS7 | Exon | 0.03 | down |
| chr16 | 19035680 | 19038031 | 2352 | ENSG00000170537 | processed_transcript | TMC7 | Exon | 0.03 | down |
| chr3 | 141291595 | 141293241 | 1647 | ENSG00000155893 | processed_transcript | PXYLP1 | 3' UTR | 0.03 | down |
| chr19 | 52928970 | 52942601 | 13632 | ENSG00000213801 | processed_transcript | ZNF321P | Exon | 0.03 | down |
| chr6 | 36969673 | 36969793 | 121 | ENSG00000137409 | processed_transcript | MTCH1 | 3' UTR | 0.03 | down |
| chr19 | 43591567 | 43591867 | 301 | ENSG00000167378 | processed_transcript | IRGQ | 3' UTR | 0.03 | down |
| chr6 | 87674919 | 87675277 | 359 | ENSG00000135334 | processed_transcript | AKIRIN2 | 3' UTR | 0.03 | down |
| chr11 | 118505988 | 118507540 | 1553 | ENSG00000118058 | protein_coding | KMT2A | Exon | 0.03 | down |
| chr7 | 17793696 | 17794356 | 661 | ENSG00000071189 | processed_transcript | SNX13 | 3' UTR | 0.03 | down |
| chr4 | 94451916 | 94455496 | 3581 | ENSG00000163110 | protein_coding | PDLIM5 | 3' UTR | 0.03 | down |
| chr17 | 50975024 | 50976615 | 1592 | ENSG00000008294 | retained_intron | SPAG9 | Exon | 0.03 | up |
| chr19 | 44955063 | 44955718 | 656 | ENSG00000104853 | protein_coding | CLPTM1 | 5' UTR | 0.03 | up |
| chr19 | 54456693 | 54456814 | 122 | ENSG00000167615 | protein_coding | LENG8 | Exon | 0.03 | up |
| chr20 | 63738718 | 63740398 | 1681 | ENSG00000125520 | protein_coding | SLC2A4RG | 3' UTR | 0.03 | up |
| chr22 | 19179472 | 19179892 | 421 | ENSG00000100075 | retained_intron | SLC25A1 | 3' UTR | 0.03 | up |
| chr15 | 61917413 | 61919395 | 1983 | ENSG00000129003 | retained_intron | VPS13C | 3' UTR | 0.03 | up |
| chr15 | 70892106 | 70892433 | 328 | ENSG00000129028 | protein_coding | THAP10 | 5' UTR | 0.03 | up |
| chr3 | 48260831 | 48268046 | 7216 | ENSG00000164048 | processed_transcript | ZNF589 | 3' UTR | 0.03 | up |
| chr5 | 149545382 | 149545782 | 401 | ENSG00000241112 | processed_pseudogene | RPL29P14 | Exon | 0.03 | up |
| chr2 | 190691296 | 190691417 | 122 | ENSG00000138386 | processed_transcript | NAB1 | 3' UTR | 0.03 | up |
| chr8 | 109334353 | 109334503 | 151 | ENSG00000120533 | processed_transcript | ENY2 | 5' UTR | 0.03 | up |
| chr13 | 102838659 | 102840038 | 1380 | ENSG00000134899 | nonsense_mediated_decay | ERCC5 | 3' UTR | 0.03 | up |
| chr11 | 83180376 | 83184083 | 3708 | ENSG00000165494 | retained_intron | PCF11 | Exon | 0.03 | up |
| chr7 | 77768702 | 77773345 | 4644 | ENSG00000187257 | protein_coding | RSBN1L | Exon | 0.03 | up |
| chr6 | 43526229 | 43526380 | 152 | ENSG00000124571 | retained_intron | XPO5 | Exon | 0.03 | up |
| chr19 | 37185765 | 37186663 | 899 | ENSG00000245680 | retained_intron | ZNF585B | 3' UTR | 0.03 | down |
| chr13 | 45033266 | 45033536 | 271 | ENSG00000133114 | processed_transcript | GPALPP1 | 3' UTR | 0.03 | down |
| chr3 | 175096823 | 175124448 | 27626 | ENSG00000177694 | processed_transcript | NAALADL2 | Exon | 0.03 | down |
| chr9 | 128308059 | 128308149 | 91 | ENSG00000167112 | processed_transcript | TRUB2 | 3' UTR | 0.03 | down |
| chr3 | 11643752 | 11671309 | 27558 | ENSG00000144560 | protein_coding | VGLL4 | 5' UTR | 0.03 | down |
| chr5 | 43609259 | 43613055 | 3797 | ENSG00000112992 | protein_coding | NNT | 3' UTR | 0.03 | down |
| chr20 | 5967532 | 5967955 | 424 | ENSG00000231961 | processed_pseudogene | AL035461 | Exon | 0.03 | down |
| chr1 | 153983200 | 153983470 | 271 | ENSG00000143545 | processed_transcript | RAB13 | Exon | 0.03 | down |
| chr3 | 184135067 | 184135337 | 271 | ENSG00000145191 | retained_intron | EIF2B5 | Exon | 0.03 | down |
| X | 77828051 | 77828261 | 211 | ENSG00000085224 | protein_coding | ATRX | 3' UTR | 0.03 | down |
| chr17 | 68247015 | 68247492 | 478 | ENSG00000265100 | lncRNA | AC005332 | Exon | 0.03 | down |
| chr3 | 50336408 | 50336588 | 181 | ENSG00000068028 | protein_coding | RASSF1 | Exon | 0.03 | down |
| chr1 | 11295030 | 11296049 | 1020 | ENSG00000120942 | protein_coding | UBIAD1 | 3' UTR | 0.03 | down |
| chr1 | 6463821 | 6464358 | 538 | ENSG00000215788 | retained_intron | TNFRSF25 | Exon | 0.03 | down |
| chr2 | 9407553 | 9408377 | 825 | ENSG00000119185 | retained_intron | ITGB1BP1 | 3' UTR | 0.03 | down |
| chr1 | 43448774 | 43449375 | 602 | ENSG00000274975 | miRNA | MIR6735 | Exon | 0.03 | down |
| chr6 | 154832936 | 154860894 | 27959 | ENSG00000146426 | processed_transcript | TIAM2 | 3' UTR | 0.03 | down |
| chr14 | 70370983 | 70371911 | 929 | ENSG00000133983 | processed_transcript | COX16 | 3' UTR | 0.03 | down |
| chr12 | 49336536 | 49337188 | 653 | ENSG00000186897 | protein_coding | C1QL4 | 5' UTR | 0.03 | down |
| X | 10117226 | 10122634 | 5409 | ENSG00000073464 | protein_coding | CLCN4 | Exon | 0.03 | up |
| chr4 | 6824224 | 6824524 | 301 | ENSG00000170871 | processed_transcript | KIAA0232 | 5' UTR | 0.03 | up |
| chr3 | 50570404 | 50570585 | 182 | ENSG00000114735 | nonsense_mediated_decay | HEMK1 | Exon | 0.03 | up |
| chr9 | 128683803 | 128685208 | 1406 | ENSG00000119335 | protein_coding | SET | 5' UTR | 0.03 | up |
| chr12 | 7210076 | 7210227 | 152 | ENSG00000139197 | nonsense_mediated_decay | PEX5 | 3' UTR | 0.03 | up |
| chr5 | 140458727 | 140459179 | 453 | ENSG00000131503 | retained_intron | ANKHD1 | 5' UTR | 0.03 | up |
| chr10 | 43557179 | 43567982 | 10804 | ENSG00000196793 | protein_coding | ZNF239 | 5' UTR | 0.03 | up |
| X | 93671676 | 93672301 | 626 | ENSG00000186310 | protein_coding | NAP1L3 | 3' UTR | 0.03 | up |
| chr9 | 134844032 | 134844543 | 512 | ENSG00000130635 | retained_intron | COL5A1 | 3' UTR | 0.03 | up |
| chr3 | 195696455 | 195698782 | 2328 | ENSG00000242086 | lncRNA | MUC20-OT1 | Exon | 0.03 | up |
| chr12 | 68841945 | 68842384 | 440 | ENSG00000256664 | processed_pseudogene | AC025423 | 3' UTR | 0.03 | up |
| chr17 | 39405873 | 39406174 | 302 | ENSG00000108306 | protein_coding | FBXL20 | 3' UTR | 0.03 | up |
| chr16 | 67082266 | 67093533 | 11268 | ENSG00000067955 | retained_intron | CBFB | 3' UTR | 0.03 | up |
| chr20 | 13617009 | 13630101 | 13093 | ENSG00000089123 | protein_coding | TASP1 | 5' UTR | 0.03 | up |
| chr5 | 140824473 | 140824803 | 331 | ENSG00000204965 | protein_coding | PCDHA5 | 3' UTR | 0.03 | down |
| chr10 | 95874073 | 95874614 | 542 | ENSG00000226688 | lncRNA | ENTPD1-AS1 | Exon | 0.03 | down |
| chr15 | 60393155 | 60393246 | 92 | ENSG00000182718 | protein_coding | ANXA2 | 5' UTR | 0.03 | down |
| chr4 | 75527080 | 75527381 | 302 | ENSG00000174796 | protein_coding | THAP6 | 3' UTR | 0.03 | down |
| chr21 | 39199089 | 39199420 | 332 | ENSG00000185658 | retained_intron | BRWD1 | 3' UTR | 0.03 | down |
| chr1 | 184791023 | 184791353 | 331 | ENSG00000135842 | protein_coding | NIBAN1 | 3' UTR | 0.03 | down |
| chr12 | 54239448 | 54239539 | 92 | ENSG00000283803 | miRNA | MIR3198-2 | 3' UTR | 0.03 | down |
| chr2 | 86195675 | 86196049 | 375 | ENSG00000132305 | protein_coding | IMMT | Exon | 0.03 | down |
| chr1 | 64691593 | 64692311 | 719 | ENSG00000158966 | retained_intron | CACHD1 | 3' UTR | 0.03 | down |
| chr16 | 67932651 | 67932802 | 152 | ENSG00000141086 | retained_intron | CTRL | Exon | 0.03 | down |
| chr16 | 22287029 | 22287600 | 572 | ENSG00000103319 | processed_transcript | EEF2K | 3' UTR | 0.03 | down |
| chr19 | 56624505 | 56624955 | 451 | ENSG00000268182 | protein_coding | SMIM17 | 3' UTR | 0.03 | down |
| X | 45063424 | 45068697 | 5274 | ENSG00000147050 | retained_intron | KDM6A | 3' UTR | 0.03 | down |
| chr16 | 10942198 | 10942468 | 271 | ENSG00000182108 | protein_coding | DEXI | 5' UTR | 0.03 | down |
| chr17 | 4271497 | 4271767 | 271 | ENSG00000185722 | nonsense_mediated_decay | ANKFY1 | 3' UTR | 0.03 | down |
| chr11 | 64359267 | 64359565 | 299 | ENSG00000162302 | protein_coding | RPS6KA4 | Exon | 0.03 | down |
| chr12 | 56424866 | 56428290 | 3425 | ENSG00000111602 | retained_intron | TIMELESS | Exon | 0.03 | down |
| chr8 | 143309418 | 143310272 | 855 | ENSG00000184428 | retained_intron | TOP1MT | 3' UTR | 0.03 | down |
| chr12 | 1491737 | 1492697 | 961 | ENSG00000249628 | lncRNA | LINC00942 | 3' UTR | 0.03 | up |
| chr15 | 70058243 | 70058334 | 92 | ENSG00000140332 | retained_intron | TLE3 | Exon | 0.03 | up |
| chr3 | 11601957 | 11604379 | 2423 | ENSG00000144560 | protein_coding | VGLL4 | 5' UTR | 0.03 | up |
| chr11 | 64987974 | 64988933 | 960 | ENSG00000168062 | protein_coding | BATF2 | 3' UTR | 0.03 | up |
| chr16 | 46922308 | 46922399 | 92 | ENSG00000166123 | retained_intron | GPT2 | Exon | 0.03 | up |
| chr16 | 970827 | 981168 | 10342 | ENSG00000103227 | protein_coding | LMF1 | 5' UTR | 0.03 | up |
| chr2 | 42704196 | 42708900 | 4705 | ENSG00000057935 | retained_intron | MTA3 | 3' UTR | 0.03 | up |
| chr16 | 67943301 | 67944071 | 771 | ENSG00000213398 | protein_coding | LCAT | 3' UTR | 0.03 | up |
| chr15 | 48474530 | 48481745 | 7216 | ENSG00000166147 | nonsense_mediated_decay | FBN1 | Exon | 0.03 | up |
| chr3 | 158691148 | 158691742 | 595 | ENSG00000168827 | protein_coding | GFM1 | 3' UTR | 0.03 | up |
| chr15 | 65258630 | 65258810 | 181 | ENSG00000138617 | nonsense_mediated_decay | PARP16 | 3' UTR | 0.03 | up |
| chr8 | 213279 | 213593 | 315 | ENSG00000223508 | transcribed_processed_pseudogene | RPL23AP53 | Exon | 0.03 | up |
| chr16 | 89517994 | 89518445 | 452 | ENSG00000197912 | processed_transcript | SPG7 | Exon | 0.03 | up |
| chr1 | 149941677 | 149941947 | 271 | ENSG00000014914 | protein_coding | MTMR11 | 3' UTR | 0.03 | up |
| chr10 | 29409580 | 29410380 | 801 | ENSG00000224597 | processed_transcript | SVIL-AS1 | Exon | 0.03 | up |
| X | 93671673 | 93672299 | 627 | ENSG00000186310 | protein_coding | NAP1L3 | 3' UTR | 0.03 | up |
| chr13 | 95970194 | 95985028 | 14835 | ENSG00000102595 | processed_transcript | UGGT2 | 3' UTR | 0.03 | up |
| chr1 | 58681986 | 58689069 | 7084 | ENSG00000162601 | processed_transcript | MYSM1 | 3' UTR | 0.03 | up |
| chr2 | 96272823 | 96273064 | 242 | ENSG00000144021 | retained_intron | CIAO1 | 3' UTR | 0.03 | up |
| chr19 | 4543491 | 4543972 | 482 | ENSG00000167680 | retained_intron | SEMA6B | 3' UTR | 0.03 | up |
| chr15 | 84573022 | 84578346 | 5325 | ENSG00000259728 | processed_transcript | LINC00933 | Exon | 0.03 | up |
| chr6 | 28326068 | 28326717 | 650 | ENSG00000235109 | processed_transcript | ZSCAN31 | 3' UTR | 0.03 | up |
| chr5 | 1038348 | 1038676 | 329 | ENSG00000145506 | retained_intron | NKD2 | 3' UTR | 0.03 | up |
| chr21 | 33772146 | 33774396 | 2251 | ENSG00000205726 | retained_intron | ITSN1 | 3' UTR | 0.03 | up |
| chr6 | 73424097 | 73424516 | 420 | ENSG00000164430 | retained_intron | CGAS | 3' UTR | 0.03 | down |
| chr12 | 51786699 | 51789309 | 2611 | ENSG00000196876 | processed_transcript | SCN8A | 3' UTR | 0.03 | down |
| chr1 | 243164637 | 243169273 | 4637 | ENSG00000143702 | processed_transcript | CEP170 | Exon | 0.03 | down |
| chr9 | 14314337 | 14314548 | 212 | ENSG00000147862 | protein_coding | NFIB | 5' UTR | 0.03 | down |
| chr6 | 32045219 | 32045340 | 122 | ENSG00000168477 | protein_coding | TNXB | 5' UTR | 0.03 | down |
| chr7 | 66640719 | 66640960 | 242 | ENSG00000243335 | protein_coding | KCTD7 | 3' UTR | 0.03 | down |
| chr17 | 7858400 | 7883260 | 24861 | ENSG00000280046 | TEC | AC104581 | 5' UTR | 0.03 | down |
| chr1 | 145746359 | 145746540 | 182 | ENSG00000265491 | retained_intron | RNF115 | 3' UTR | 0.03 | down |
| chr1 | 1615375 | 1615615 | 241 | ENSG00000197530 | protein_coding | MIB2 | Exon | 0.03 | down |
| chr16 | 69693762 | 69695498 | 1737 | ENSG00000102908 | retained_intron | NFAT5 | 3' UTR | 0.03 | down |
| chr14 | 92039565 | 92040896 | 1332 | ENSG00000100815 | protein_coding | TRIP11 | 5' UTR | 0.03 | down |
| chr17 | 76559890 | 76560221 | 332 | ENSG00000199961 | snoRNA | SNORD1B | Exon | 0.03 | up |
| chr17 | 7861144 | 7861743 | 600 | ENSG00000280046 | TEC | AC104581 | 3' UTR | 0.03 | up |
| chr3 | 152463200 | 152463980 | 781 | ENSG00000152601 | retained_intron | MBNL1 | 3' UTR | 0.03 | up |
| chr17 | 47053917 | 47054247 | 331 | ENSG00000262879 | lncRNA | AC005670 | Exon | 0.03 | up |
| chr12 | 55716036 | 55716368 | 333 | ENSG00000135424 | nonsense_mediated_decay | ITGA7 | 5' UTR | 0.03 | up |
| chr19 | 30004547 | 30007610 | 3064 | ENSG00000105176 | processed_transcript | URI1 | 3' UTR | 0.03 | up |
| chr15 | 78935028 | 78937149 | 2122 | ENSG00000103811 | retained_intron | CTSH | 3' UTR | 0.03 | up |
| chr16 | 3300697 | 3301627 | 931 | ENSG00000140993 | processed_transcript | TIGD7 | 5' UTR | 0.03 | up |
| chr9 | 37879518 | 37879668 | 151 | ENSG00000122696 | protein_coding | SLC25A51 | Exon | 0.03 | up |
| chr10 | 122084118 | 122084538 | 421 | ENSG00000138162 | processed_transcript | TACC2 | Exon | 0.03 | up |
| chr13 | 44538520 | 44539818 | 1299 | ENSG00000102804 | processed_transcript | TSC22D1 | 3' UTR | 0.03 | up |
| chr6 | 70525321 | 70525862 | 542 | ENSG00000082269 | retained_intron | FAM135A | 3' UTR | 0.03 | down |
| chr15 | 42739568 | 42739899 | 332 | ENSG00000140326 | protein_coding | CDAN1 | 3' UTR | 0.03 | down |
| chr11 | 119023051 | 119023111 | 61 | ENSG00000196655 | processed_transcript | TRAPPC4 | Exon | 0.03 | down |
| chr1 | 224954997 | 224961176 | 6180 | ENSG00000185842 | protein_coding | DNAH14 | Exon | 0.03 | down |
| chr5 | 149984945 | 149985186 | 242 | ENSG00000155850 | protein_coding | SLC26A2 | 3' UTR | 0.03 | down |
| chr2 | 199271535 | 199272494 | 960 | ENSG00000119042 | retained_intron | SATB2 | 3' UTR | 0.03 | down |
| chr1 | 150995309 | 150995575 | 267 | ENSG00000143363 | processed_transcript | PRUNE1 | 3' UTR | 0.03 | down |
| chr11 | 126015228 | 126017165 | 1938 | ENSG00000064309 | nonsense_mediated_decay | CDON | Exon | 0.03 | down |
| chr7 | 80217337 | 80217698 | 362 | ENSG00000127955 | retained_intron | GNAI1 | 3' UTR | 0.03 | down |
| chr8 | 81829491 | 81840270 | 10780 | ENSG00000104497 | protein_coding | SNX16 | 5' UTR | 0.03 | down |
| chr20 | 32186476 | 32187164 | 689 | ENSG00000235217 | processed_transcript | TSPY26P | Exon | 0.03 | down |
| chr2 | 61032098 | 61049080 | 16983 | ENSG00000162928 | protein_coding | PEX13 | 3' UTR | 0.03 | down |
| chr7 | 92517407 | 92518194 | 788 | ENSG00000127980 | nonsense_mediated_decay | PEX1 | Exon | 0.03 | down |
| chr7 | 12231895 | 12232315 | 421 | ENSG00000106460 | retained_intron | TMEM106B | 3' UTR | 0.03 | down |
| chr11 | 66336115 | 66336721 | 607 | ENSG00000174791 | protein_coding | RIN1 | 5' UTR | 0.03 | up |
| chr16 | 89738961 | 89739213 | 253 | ENSG00000187741 | retained_intron | FANCA | Exon | 0.03 | up |
| chr17 | 61399872 | 61400113 | 242 | ENSG00000121068 | nonsense_mediated_decay | TBX2 | 5' UTR | 0.03 | up |
| chr1 | 92333419 | 92345891 | 12473 | ENSG00000122484 | retained_intron | RPAP2 | Exon | 0.03 | up |
| chr12 | 111452471 | 111452741 | 271 | ENSG00000204842 | retained_intron | ATXN2 | 3' UTR | 0.03 | up |
| chr4 | 7759192 | 7759403 | 212 | ENSG00000196526 | retained_intron | AFAP1 | 3' UTR | 0.03 | up |
| chr20 | 63282854 | 63282915 | 62 | ENSG00000101199 | processed_transcript | ARFGAP1 | Exon | 0.03 | up |
| chr16 | 82148820 | 82151306 | 2487 | ENSG00000261235 | lncRNA | AC092142 | 3' UTR | 0.03 | up |
| chr2 | 71363990 | 71364201 | 212 | ENSG00000075292 | processed_transcript | ZNF638 | 5' UTR | 0.03 | up |
| chr1 | 113614605 | 113642368 | 27764 | ENSG00000232499 | processed_pseudogene | AL391058 | Exon | 0.03 | up |
| chr12 | 65758120 | 65758384 | 265 | ENSG00000241749 | transcribed_processed_pseudogene | RPSAP52 | Exon | 0.03 | up |
| chr13 | 94398619 | 94403472 | 4854 | ENSG00000183098 | protein_coding | GPC6 | 3' UTR | 0.03 | up |
| chr8 | 123502169 | 123502469 | 301 | ENSG00000156804 | retained_intron | FBXO32 | 3' UTR | 0.03 | up |
| chr2 | 135621595 | 135631889 | 10295 | ENSG00000048991 | processed_transcript | R3HDM1 | Exon | 0.03 | up |
| chr16 | 30919408 | 30920306 | 899 | ENSG00000260852 | lncRNA | FBXL19-AS1 | Exon | 0.03 | down |
| chr7 | 102250366 | 102250547 | 182 | ENSG00000257923 | retained_intron | CUX1 | 3' UTR | 0.03 | down |
| chr2 | 39736596 | 39744415 | 7820 | ENSG00000138050 | retained_intron | THUMPD2 | 3' UTR | 0.03 | down |
| chr14 | 37622086 | 37622356 | 271 | ENSG00000139865 | protein_coding | TTC6 | 5' UTR | 0.03 | down |
| chr21 | 43416368 | 43416579 | 212 | ENSG00000142178 | retained_intron | SIK1 | 3' UTR | 0.03 | down |
| chr1 | 153747343 | 153747433 | 91 | ENSG00000143624 | protein_coding | INTS3 | Exon | 0.03 | down |
| chr17 | 47181535 | 47188884 | 7350 | ENSG00000004897 | nonsense_mediated_decay | CDC27 | Exon | 0.03 | down |
| chr19 | 44665471 | 44666158 | 688 | ENSG00000279095 | TEC | AC243964 | 3' UTR | 0.03 | down |
| chr4 | 185399620 | 185399680 | 61 | ENSG00000186352 | protein_coding | ANKRD37 | Exon | 0.03 | down |
| chr14 | 31106739 | 31109401 | 2663 | ENSG00000092148 | retained_intron | HECTD1 | Exon | 0.03 | down |
| chr8 | 89769897 | 89780401 | 10505 | ENSG00000104312 | retained_intron | RIPK2 | 3' UTR | 0.03 | down |
| chr1 | 213240930 | 213242307 | 1378 | ENSG00000136643 | processed_transcript | RPS6KC1 | Exon | 0.03 | down |
| chr1 | 156212466 | 156212796 | 331 | ENSG00000260238 | protein_coding | PMF1-BGLAP | 3' UTR | 0.03 | down |
| chr1 | 10158354 | 10161224 | 2871 | ENSG00000130939 | nonsense_mediated_decay | UBE4B | Exon | 0.03 | up |
| chr7 | 135361944 | 135384360 | 22417 | ENSG00000080802 | processed_transcript | CNOT4 | 3' UTR | 0.03 | up |
| chr17 | 35352164 | 35353131 | 968 | ENSG00000172716 | protein_coding | SLFN11 | 3' UTR | 0.03 | up |
| chr12 | 122261399 | 122263673 | 2275 | ENSG00000139719 | nonsense_mediated_decay | VPS33A | 3' UTR | 0.03 | up |
| chr2 | 95108212 | 95109990 | 1779 | ENSG00000144029 | retained_intron | MRPS5 | Exon | 0.03 | up |
| chr1 | 51878013 | 51878727 | 715 | ENSG00000078618 | protein_coding | NRDC | 5' UTR | 0.03 | up |
| chr1 | 11789619 | 11789740 | 122 | ENSG00000177000 | protein_coding | MTHFR | 3' UTR | 0.03 | up |
| chr8 | 86473106 | 86473286 | 181 | ENSG00000176623 | protein_coding | RMDN1 | 3' UTR | 0.03 | up |
| chr11 | 103283052 | 103286342 | 3291 | ENSG00000187240 | nonsense_mediated_decay | DYNC2H1 | Exon | 0.03 | up |
| chr1 | 70354584 | 70354674 | 91 | ENSG00000118454 | protein_coding | ANKRD13C | 5' UTR | 0.03 | up |
| X | 101101101 | 101102407 | 1307 | ENSG00000102384 | protein_coding | CENPI | Exon | 0.03 | up |
| chr1 | 214332131 | 214332312 | 182 | ENSG00000143499 | protein_coding | SMYD2 | Exon | 0.03 | down |
| chr13 | 109785173 | 109785564 | 392 | ENSG00000185950 | protein_coding | IRS2 | Exon | 0.03 | down |
| chr16 | 70377265 | 70377506 | 242 | ENSG00000285710 | lncRNA | AC012184 | 3' UTR | 0.03 | down |
| chr18 | 722487 | 722698 | 212 | ENSG00000132199 | protein_coding | ENOSF1 | 3' UTR | 0.03 | down |
| chr2 | 165684069 | 165684399 | 331 | ENSG00000178662 | protein_coding | CSRNP3 | 3' UTR | 0.03 | down |
| chr16 | 30526977 | 30527337 | 361 | ENSG00000278922 | TEC | AC002310 | Exon | 0.03 | down |
| chr2 | 237094128 | 237096695 | 2568 | ENSG00000198612 | nonsense_mediated_decay | COPS8 | 3' UTR | 0.03 | down |
| chr6 | 41906008 | 41906248 | 241 | ENSG00000164663 | protein_coding | USP49 | 3' UTR | 0.03 | down |
| chr17 | 47945564 | 47945744 | 181 | ENSG00000263798 | lncRNA | AC018521 | 3' UTR | 0.03 | down |
| chr19 | 48954844 | 48955083 | 240 | ENSG00000087088 | protein_coding | BAX | 5' UTR | 0.03 | down |
| chr7 | 64369389 | 64375584 | 6196 | ENSG00000287985 | lncRNA | AC073270 | Exon | 0.03 | up |
| chr10 | 22319916 | 22320306 | 391 | ENSG00000168283 | protein_coding | BMI1 | 3' UTR | 0.03 | up |
| chr11 | 73353162 | 73356315 | 3154 | ENSG00000110237 | retained_intron | ARHGEF17 | Exon | 0.03 | up |
| chr14 | 74812774 | 74821330 | 8557 | ENSG00000119596 | protein_coding | YLPM1 | 3' UTR | 0.03 | up |
| chr16 | 71864920 | 71879962 | 15043 | ENSG00000102984 | protein_coding | ZNF821 | 3' UTR | 0.03 | up |
| chr19 | 14717710 | 14719494 | 1785 | ENSG00000160961 | retained_intron | ZNF333 | 3' UTR | 0.03 | up |
| chr2 | 24826267 | 24826717 | 451 | ENSG00000271936 | lncRNA | AC012073 | Exon | 0.03 | up |
| chr12 | 110429987 | 110430223 | 237 | ENSG00000258011 | processed_pseudogene | HMGA1P3 | Exon | 0.03 | up |
| chr12 | 7202701 | 7203549 | 849 | ENSG00000139197 | nonsense_mediated_decay | PEX5 | Exon | 0.03 | up |
| chr11 | 9961997 | 9968512 | 6516 | ENSG00000133812 | retained_intron | SBF2 | Exon | 0.03 | up |
| chr6 | 125276693 | 125276903 | 211 | ENSG00000111906 | processed_transcript | HDDC2 | Exon | 0.03 | up |
| chr15 | 55355277 | 55355548 | 272 | ENSG00000260916 | protein_coding | CCPG1 | 3' UTR | 0.03 | up |
| chr8 | 17643854 | 17644035 | 182 | ENSG00000129422 | processed_transcript | MTUS1 | 3' UTR | 0.03 | down |
| chr8 | 17643861 | 17644041 | 181 | ENSG00000104213 | processed_transcript | PDGFRL | Exon | 0.03 | down |
| chr3 | 121714865 | 121716985 | 2121 | ENSG00000173230 | protein_coding | GOLGB1 | 3' UTR | 0.03 | down |
| chr22 | 37887293 | 37887593 | 301 | ENSG00000100129 | retained_intron | EIF3L | Exon | 0.03 | down |
| chr12 | 753628 | 753929 | 302 | ENSG00000060237 | protein_coding | WNK1 | Exon | 0.03 | down |
| chr3 | 49658119 | 49661098 | 2980 | ENSG00000164062 | protein_coding | APEH | Exon | 0.03 | down |
| chr6 | 34284123 | 34284304 | 182 | ENSG00000225339 | lncRNA | AL354740 | Exon | 0.03 | down |
| chr1 | 156676568 | 156676749 | 182 | ENSG00000132688 | protein_coding | NES | Exon | 0.03 | down |
| chr19 | 40319924 | 40320612 | 689 | ENSG00000160392 | protein_coding | C19orf47 | 3' UTR | 0.03 | down |
| chr17 | 80221213 | 80221483 | 271 | ENSG00000181045 | processed_transcript | SLC26A11 | 5' UTR | 0.03 | down |
| chr16 | 81028637 | 81028938 | 302 | ENSG00000261061 | lncRNA | AC092718 | 3' UTR | 0.03 | down |
| chr7 | 98206522 | 98207092 | 571 | ENSG00000180535 | protein_coding | BHLHA15 | 3' UTR | 0.03 | down |
| chr6 | 57147181 | 57150854 | 3674 | ENSG00000112200 | retained_intron | ZNF451 | Exon | 0.03 | down |
| chr3 | 196234403 | 196234943 | 541 | ENSG00000161217 | retained_intron | PCYT1A | 3' UTR | 0.03 | down |
| chr11 | 116843401 | 116844212 | 812 | ENSG00000160584 | protein_coding | SIK3 | 3' UTR | 0.03 | down |
| chr16 | 89738948 | 89739218 | 271 | ENSG00000158805 | retained_intron | ZNF276 | 3' UTR | 0.03 | up |
| chr17 | 8288653 | 8288939 | 287 | ENSG00000108961 | protein_coding | RANGRF | 5' UTR | 0.03 | up |
| chr22 | 41205281 | 41208330 | 3050 | ENSG00000100395 | retained_intron | L3MBTL2 | 5' UTR | 0.03 | up |
| chr15 | 22867445 | 22867625 | 181 | ENSG00000140157 | retained_intron | NIPA2 | 3' UTR | 0.03 | up |
| chr6 | 152974926 | 152983430 | 8505 | ENSG00000112029 | protein_coding | FBXO5 | 5' UTR | 0.03 | up |
| chr2 | 178392515 | 178396145 | 3631 | ENSG00000223960 | lncRNA | CHROMR | 3' UTR | 0.03 | up |
| chr1 | 3837473 | 3837594 | 122 | ENSG00000116198 | processed_transcript | CEP104 | 3' UTR | 0.03 | up |
| chr14 | 55175913 | 55180744 | 4832 | ENSG00000126787 | protein_coding | DLGAP5 | Exon | 0.03 | up |
| chr1 | 28510056 | 28510266 | 211 | ENSG00000180198 | protein_coding | RCC1 | 5' UTR | 0.03 | up |
| chr1 | 45607865 | 45608195 | 331 | ENSG00000132780 | processed_transcript | NASP | 3' UTR | 0.03 | up |
| chr3 | 122410213 | 122410453 | 241 | ENSG00000272758 | lncRNA | AC083798 | 3' UTR | 0.03 | down |
| chr19 | 54108866 | 54109227 | 362 | ENSG00000170906 | processed_transcript | NDUFA3 | Exon | 0.03 | down |
| chr1 | 101085959 | 101087244 | 1286 | ENSG00000233184 | lncRNA | AC093157 | Exon | 0.03 | down |
| chr10 | 93323409 | 93323500 | 92 | ENSG00000138119 | retained_intron | MYOF | Exon | 0.03 | down |
| chr5 | 661385 | 661835 | 451 | ENSG00000171368 | protein_coding | TPPP | 3' UTR | 0.03 | down |
| chr14 | 50322602 | 50322842 | 241 | ENSG00000125375 | retained_intron | DMAC2L | 3' UTR | 0.03 | down |
| chr11 | 30330923 | 30331372 | 450 | ENSG00000152219 | processed_transcript | ARL14EP | 5' UTR | 0.03 | down |
| chr17 | 34980511 | 34980798 | 288 | ENSG00000005156 | protein_coding | LIG3 | 5' UTR | 0.03 | down |
| chr20 | 20505533 | 20513248 | 7716 | ENSG00000188559 | protein_coding | RALGAPA2 | 5' UTR | 0.03 | down |
| chr7 | 38754923 | 38772598 | 17676 | ENSG00000006715 | retained_intron | VPS41 | Exon | 0.03 | down |
| chr16 | 1365782 | 1366322 | 541 | ENSG00000059145 | processed_transcript | UNKL | 3' UTR | 0.03 | down |
| chr17 | 8288630 | 8288900 | 271 | ENSG00000108961 | protein_coding | RANGRF | 3' UTR | 0.03 | up |
| chr16 | 67163624 | 67164240 | 617 | ENSG00000135722 | retained_intron | FBXL8 | 3' UTR | 0.03 | up |
| chr5 | 139391876 | 139392325 | 450 | ENSG00000170476 | nonsense_mediated_decay | MZB1 | 3' UTR | 0.03 | up |
| chr19 | 40214975 | 40215575 | 601 | ENSG00000130758 | retained_intron | MAP3K10 | 3' UTR | 0.03 | up |
| chr9 | 137615061 | 137615360 | 300 | ENSG00000197070 | processed_transcript | ARRDC1 | 3' UTR | 0.03 | up |
| chr11 | 43892044 | 43897318 | 5275 | ENSG00000213693 | processed_pseudogene | SEC14L1P1 | Exon | 0.03 | up |
| chr2 | 238098396 | 238098636 | 241 | ENSG00000144488 | protein_coding | ESPNL | 3' UTR | 0.03 | up |
| chr9 | 113203019 | 113206114 | 3096 | ENSG00000119321 | processed_transcript | FKBP15 | Exon | 0.03 | up |
| chr8 | 58145935 | 58146445 | 511 | ENSG00000169122 | processed_transcript | FAM110B | 5' UTR | 0.03 | up |
| chr2 | 222930665 | 222933110 | 2446 | ENSG00000123983 | retained_intron | ACSL3 | 3' UTR | 0.03 | up |
| chr2 | 110545142 | 110545589 | 448 | ENSG00000183054 | retained_intron | RGPD6 | 3' UTR | 0.03 | up |
| chr3 | 113327620 | 113330268 | 2649 | ENSG00000206530 | nonsense_mediated_decay | CFAP44 | Exon | 0.02 | down |
| chr16 | 89543353 | 89543594 | 242 | ENSG00000197912 | retained_intron | SPG7 | Exon | 0.02 | down |
| chr7 | 105457172 | 105457473 | 302 | ENSG00000091127 | retained_intron | PUS7 | 3' UTR | 0.02 | down |
| chr11 | 129924968 | 129929419 | 4452 | ENSG00000170325 | protein_coding | PRDM10 | Exon | 0.02 | down |
| chr11 | 123071816 | 123072116 | 301 | ENSG00000109971 | protein_coding | HSPA8 | 3' UTR | 0.02 | down |
| chr16 | 69691843 | 69692234 | 392 | ENSG00000102908 | retained_intron | NFAT5 | 3' UTR | 0.02 | down |
| chr12 | 68855854 | 68856633 | 780 | ENSG00000135678 | nonsense_mediated_decay | CPM | 3' UTR | 0.02 | down |
| chr19 | 53443299 | 53444223 | 925 | ENSG00000160336 | protein_coding | ZNF761 | Exon | 0.02 | down |
| chr19 | 52928951 | 52952824 | 23874 | ENSG00000213801 | processed_transcript | ZNF321P | 3' UTR | 0.02 | down |
| chr4 | 76114986 | 76115493 | 508 | ENSG00000138750 | retained_intron | NUP54 | 3' UTR | 0.02 | down |
| chr3 | 127037121 | 127037392 | 272 | ENSG00000114554 | processed_transcript | PLXNA1 | 3' UTR | 0.02 | down |
| chr6 | 154832924 | 154833674 | 751 | ENSG00000146426 | processed_transcript | TIAM2 | 3' UTR | 0.02 | down |
| chr2 | 84927057 | 84966626 | 39570 | ENSG00000287625 | lncRNA | AC022210 | Exon | 0.02 | down |
| chr12 | 132748175 | 132748941 | 767 | ENSG00000176915 | processed_transcript | ANKLE2 | Exon | 0.02 | down |
| chr7 | 100351452 | 100351900 | 449 | ENSG00000272752 | lncRNA | STAG3L5P-PVRIG2P-PILRB | Exon | 0.02 | up |
| chr11 | 68891275 | 68891514 | 240 | ENSG00000197345 | protein_coding | MRPL21 | 3' UTR | 0.02 | up |
| X | 19358927 | 19359868 | 942 | ENSG00000131828 | processed_transcript | PDHA1 | 3' UTR | 0.02 | up |
| chr5 | 67164174 | 67165402 | 1229 | ENSG00000069020 | protein_coding | MAST4 | Exon | 0.02 | up |
| chr16 | 58038370 | 58040178 | 1809 | ENSG00000102996 | protein_coding | MMP15 | Exon | 0.02 | up |
| chr10 | 67922647 | 67932601 | 9955 | ENSG00000148634 | processed_transcript | HERC4 | 3' UTR | 0.02 | up |
| chr1 | 235437472 | 235448393 | 10922 | ENSG00000284770 | retained_intron | TBCE | 3' UTR | 0.02 | up |
| chr17 | 5408903 | 5416563 | 7661 | ENSG00000108559 | protein_coding | NUP88 | 3' UTR | 0.02 | up |
| X | 53082366 | 53082607 | 242 | ENSG00000184205 | retained_intron | TSPYL2 | 5' UTR | 0.02 | up |
| chr17 | 35353760 | 35360079 | 6320 | ENSG00000172716 | protein_coding | SLFN11 | Exon | 0.02 | up |
| chr2 | 176131499 | 176131829 | 331 | ENSG00000175879 | protein_coding | HOXD8 | 3' UTR | 0.02 | up |
| chr8 | 73324621 | 73324742 | 122 | ENSG00000121039 | retained_intron | RDH10 | 3' UTR | 0.02 | up |
| chr5 | 35878524 | 35878854 | 331 | ENSG00000168685 | retained_intron | IL7R | 3' UTR | 0.02 | up |
| chr6 | 31465268 | 31465598 | 331 | ENSG00000206337 | lncRNA | HCP5 | Exon | 0.02 | up |
| chr19 | 1470878 | 1471328 | 451 | ENSG00000115266 | protein_coding | APC2 | 3' UTR | 0.02 | down |
| chr16 | 67680500 | 67680830 | 331 | ENSG00000141098 | retained_intron | GFOD2 | 3' UTR | 0.02 | down |
| chr5 | 172336264 | 172336355 | 92 | ENSG00000174705 | processed_transcript | SH3PXD2B | 3' UTR | 0.02 | down |
| chr18 | 62577100 | 62577520 | 421 | ENSG00000141664 | nonsense_mediated_decay | ZCCHC2 | 3' UTR | 0.02 | down |
| chr17 | 37613782 | 37619823 | 6042 | ENSG00000274620 | miRNA | MIR378J | 3' UTR | 0.02 | down |
| chr11 | 65569659 | 65570010 | 352 | ENSG00000260233 | lncRNA | ZNRD2-AS1 | Exon | 0.02 | down |
| chr6 | 109481099 | 109483159 | 2061 | ENSG00000112365 | protein_coding | ZBTB24 | 5' UTR | 0.02 | down |
| chr2 | 3545641 | 3549066 | 3426 | ENSG00000171865 | retained_intron | RNASEH1 | 3' UTR | 0.02 | down |
| chr1 | 44103538 | 44104377 | 840 | ENSG00000230615 | lncRNA | AL139220 | Exon | 0.02 | down |
| chr2 | 74880405 | 74880525 | 121 | ENSG00000159399 | retained_intron | HK2 | Exon | 0.02 | down |
| chr19 | 38922241 | 38945076 | 22836 | ENSG00000104835 | protein_coding | SARS2 | 3' UTR | 0.02 | down |
| chr11 | 93793694 | 93793995 | 302 | ENSG00000042429 | retained_intron | MED17 | 3' UTR | 0.02 | down |
| chr14 | 61321883 | 61322183 | 301 | ENSG00000027075 | protein_coding | PRKCH | 5' UTR | 0.02 | down |
| chr19 | 4798272 | 4799653 | 1382 | ENSG00000141965 | protein_coding | FEM1A | 3' UTR | 0.02 | up |
| chr22 | 19978961 | 19979172 | 212 | ENSG00000099889 | retained_intron | ARVCF | Exon | 0.02 | up |
| chr1 | 89583287 | 89593100 | 9814 | ENSG00000231999 | lncRNA | LRRC8C-DT | 3' UTR | 0.02 | up |
| chr12 | 44388146 | 44389343 | 1198 | ENSG00000139173 | processed_transcript | TMEM117 | 3' UTR | 0.02 | up |
| chr14 | 24210475 | 24211826 | 1352 | ENSG00000254505 | retained_intron | CHMP4A | 3' UTR | 0.02 | up |
| chr5 | 55937877 | 55938238 | 362 | ENSG00000134352 | retained_intron | IL6ST | 3' UTR | 0.02 | up |
| chr12 | 29536215 | 29568745 | 32531 | ENSG00000133687 | processed_transcript | TMTC1 | 3' UTR | 0.02 | up |
| chr20 | 5942445 | 5943576 | 1132 | ENSG00000089195 | processed_transcript | TRMT6 | Exon | 0.02 | up |
| chr14 | 61776234 | 61792863 | 16630 | ENSG00000279633 | TEC | AL137918 | Exon | 0.02 | up |
| chr1 | 146994675 | 146994885 | 211 | ENSG00000268043 | protein_coding | NBPF12 | 3' UTR | 0.02 | up |
| chr17 | 61388586 | 61388827 | 242 | ENSG00000141376 | retained_intron | BCAS3 | Exon | 0.02 | up |
| chr16 | 78115074 | 78130116 | 15043 | ENSG00000186153 | processed_transcript | WWOX | 3' UTR | 0.02 | up |
| chr11 | 43882713 | 43882774 | 62 | ENSG00000166199 | processed_transcript | ALKBH3 | Exon | 0.02 | up |
| X | 73994833 | 73995073 | 241 | ENSG00000225470 | lncRNA | JPX | Exon | 0.02 | down |
| chr14 | 55633335 | 55637805 | 4471 | ENSG00000126777 | nonsense_mediated_decay | KTN1 | 5' UTR | 0.02 | down |
| chr2 | 237762633 | 237762964 | 332 | ENSG00000124831 | retained_intron | LRRFIP1 | Exon | 0.02 | down |
| chr16 | 58116088 | 58116614 | 527 | ENSG00000070761 | retained_intron | CFAP20 | Exon | 0.02 | down |
| chr6 | 99280811 | 99281351 | 541 | ENSG00000146267 | processed_transcript | FAXC | 3' UTR | 0.02 | down |
| chr6 | 43340794 | 43354712 | 13919 | ENSG00000171467 | protein_coding | ZNF318 | Exon | 0.02 | down |
| chr17 | 42955833 | 42956132 | 300 | ENSG00000266967 | nonsense_mediated_decay | AARSD1 | 3' UTR | 0.02 | down |
| chr12 | 132704729 | 132714912 | 10184 | ENSG00000247077 | protein_coding | PGAM5 | 3' UTR | 0.02 | down |
| chr19 | 54156892 | 54159721 | 2830 | ENSG00000105617 | protein_coding | LENG1 | 5' UTR | 0.02 | up |
| chr11 | 68745042 | 68750200 | 5159 | ENSG00000132749 | retained_intron | TESMIN | Exon | 0.02 | up |
| chr5 | 9549928 | 9550314 | 387 | ENSG00000239112 | snoRNA | SNORD123 | Exon | 0.02 | up |
| chr3 | 19983769 | 19984278 | 510 | ENSG00000144566 | retained_intron | RAB5A | 3' UTR | 0.02 | up |
| chr5 | 42718463 | 42719722 | 1260 | ENSG00000112964 | processed_transcript | GHR | 3' UTR | 0.02 | up |
| chr4 | 65414354 | 65490493 | 76140 | ENSG00000285711 | lncRNA | LINC02835 | Exon | 0.02 | up |
| chr17 | 82188321 | 82194197 | 5877 | ENSG00000176155 | retained_intron | CCDC57 | 3' UTR | 0.02 | up |
| chr2 | 6849012 | 6849342 | 331 | ENSG00000225964 | lncRNA | NRIR | 3' UTR | 0.02 | up |
| chr20 | 10637683 | 10637924 | 242 | ENSG00000101384 | retained_intron | JAG1 | 3' UTR | 0.02 | up |
| chr7 | 27792132 | 27803169 | 11038 | ENSG00000106052 | protein_coding | TAX1BP1 | 3' UTR | 0.02 | up |
| chr15 | 84202426 | 84202547 | 122 | ENSG00000225151 | processed_transcript | GOLGA2P7 | Exon | 0.02 | up |
| chr4 | 122743450 | 122744226 | 777 | ENSG00000181004 | protein_coding | BBS12 | 3' UTR | 0.02 | up |
| chr7 | 77920416 | 77922723 | 2308 | ENSG00000006576 | retained_intron | PHTF2 | Exon | 0.02 | up |
| chr6 | 125889429 | 125889849 | 421 | ENSG00000111912 | processed_transcript | NCOA7 | Exon | 0.02 | down |
| chr18 | 55227430 | 55228150 | 721 | ENSG00000196628 | protein_coding | TCF4 | 3' UTR | 0.02 | down |
| chr14 | 20365786 | 20366027 | 242 | ENSG00000129566 | protein_coding | TEP1 | 3' UTR | 0.02 | down |
| chr8 | 123239802 | 123239982 | 181 | ENSG00000189376 | retained_intron | C8orf76 | Exon | 0.02 | down |
| chr13 | 95021452 | 95043434 | 21983 | ENSG00000125257 | retained_intron | ABCC4 | 3' UTR | 0.02 | down |
| chr1 | 15661245 | 15661846 | 602 | ENSG00000215695 | protein_coding | RSC1A1 | 3' UTR | 0.02 | down |
| chr2 | 38295720 | 38296230 | 511 | ENSG00000119787 | retained_intron | ATL2 | 3' UTR | 0.02 | down |
| chr15 | 88621336 | 88621516 | 181 | ENSG00000181026 | retained_intron | AEN | 5' UTR | 0.02 | down |
| chr22 | 17689096 | 17695861 | 6766 | ENSG00000099968 | processed_transcript | BCL2L13 | 5' UTR | 0.02 | down |
| chr17 | 2041750 | 2042021 | 272 | ENSG00000287553 | lncRNA | AC090617 | 3' UTR | 0.02 | down |
| chr11 | 66537332 | 66537873 | 542 | ENSG00000174165 | processed_transcript | ZDHHC24 | 3' UTR | 0.02 | down |
| chr10 | 97401200 | 97401345 | 146 | ENSG00000052749 | protein_coding | RRP12 | Exon | 0.02 | up |
| chr19 | 13765993 | 13766054 | 62 | ENSG00000037757 | retained_intron | MRI1 | Exon | 0.02 | up |
| chr15 | 24955014 | 24962154 | 7141 | ENSG00000214265 | nonsense_mediated_decay | AC124312 | 5' UTR | 0.02 | up |
| chr1 | 112673440 | 112674927 | 1488 | ENSG00000155363 | protein_coding | MOV10 | 5' UTR | 0.02 | up |
| chr3 | 18348461 | 18349540 | 1080 | ENSG00000182568 | retained_intron | SATB1 | 3' UTR | 0.02 | up |
| chr17 | 36599100 | 36599549 | 450 | ENSG00000278535 | retained_intron | DHRS11 | Exon | 0.02 | up |
| chr4 | 67722232 | 67722922 | 691 | ENSG00000248049 | lncRNA | UBA6-AS1 | Exon | 0.02 | up |
| chr16 | 67638786 | 67638937 | 152 | ENSG00000159753 | nonsense_mediated_decay | CARMIL2 | 3' UTR | 0.02 | up |
| chr3 | 73064055 | 73065269 | 1215 | ENSG00000163605 | protein_coding | PPP4R2 | 3' UTR | 0.02 | up |
| chr12 | 122231452 | 122231783 | 332 | ENSG00000284934 | protein_coding | AC048338 | 3' UTR | 0.02 | up |
| chr19 | 10291747 | 10292271 | 525 | ENSG00000105376 | retained_intron | ICAM5 | Exon | 0.02 | up |
| chr13 | 79368973 | 79376342 | 7370 | ENSG00000139746 | processed_transcript | RBM26 | Exon | 0.02 | up |
| chr5 | 69313370 | 69313872 | 503 | ENSG00000213830 | processed_pseudogene | CFL1P5 | Exon | 0.02 | up |
| chr11 | 65242037 | 65242458 | 422 | ENSG00000014138 | nonsense_mediated_decay | POLA2 | Exon | 0.02 | up |
| chr5 | 140808801 | 140809220 | 420 | ENSG00000204967 | protein_coding | PCDHA4 | Exon | 0.02 | up |
| chr1 | 148748952 | 148749423 | 472 | ENSG00000177144 | protein_coding | NUDT4B | 5' UTR | 0.02 | up |
| chr16 | 30200862 | 30201087 | 226 | ENSG00000261052 | protein_coding | SULT1A3 | Exon | 0.02 | down |
| chr10 | 125748652 | 125748863 | 212 | ENSG00000107938 | retained_intron | EDRF1 | Exon | 0.02 | down |
| chr19 | 51964996 | 51965505 | 510 | ENSG00000256683 | protein_coding | ZNF350 | Exon | 0.02 | down |
| chr6 | 43618963 | 43619383 | 421 | ENSG00000124688 | protein_coding | MAD2L1BP | 3' UTR | 0.02 | down |
| chr5 | 5461846 | 5462086 | 241 | ENSG00000164151 | retained_intron | ICE1 | Exon | 0.02 | down |
| chr19 | 57864130 | 57864310 | 181 | ENSG00000198466 | processed_transcript | ZNF587 | 3' UTR | 0.02 | down |
| chr2 | 106805767 | 106805888 | 122 | ENSG00000238250 | lncRNA | ST6GAL2-IT1 | 3' UTR | 0.02 | down |
| chr12 | 122507202 | 122507912 | 711 | ENSG00000111011 | retained_intron | RSRC2 | Exon | 0.02 | down |
| chr6 | 34286138 | 34286409 | 272 | ENSG00000225339 | lncRNA | AL354740 | Exon | 0.02 | down |
| chr6 | 34286141 | 34286411 | 271 | ENSG00000220583 | processed_pseudogene | RPL35P2 | 3' UTR | 0.02 | down |
| chr5 | 138290734 | 138303663 | 12930 | ENSG00000158402 | protein_coding | CDC25C | 3' UTR | 0.02 | down |
| chr1 | 89714254 | 89715124 | 871 | ENSG00000171488 | processed_transcript | LRRC8C | 3' UTR | 0.02 | down |
| chr3 | 44659745 | 44660791 | 1047 | ENSG00000272077 | lncRNA | AC124045 | 3' UTR | 0.02 | down |
| chr1 | 241630019 | 241631247 | 1229 | ENSG00000203668 | processed_transcript | CHML | 3' UTR | 0.02 | down |
| chr17 | 42659283 | 42659640 | 358 | ENSG00000037042 | protein_coding | TUBG2 | 5' UTR | 0.02 | up |
| chr21 | 45294296 | 45295047 | 752 | ENSG00000223768 | lncRNA | LINC00205 | Exon | 0.02 | up |
| chr8 | 80053173 | 80066906 | 13734 | ENSG00000076554 | retained_intron | TPD52 | 3' UTR | 0.02 | up |
| chr4 | 2744764 | 2745483 | 720 | ENSG00000168884 | retained_intron | TNIP2 | Exon | 0.02 | up |
| chr5 | 90518722 | 90525072 | 6351 | ENSG00000176018 | protein_coding | LYSMD3 | 3' UTR | 0.02 | up |
| chr2 | 11177794 | 11178392 | 599 | ENSG00000162976 | processed_transcript | SLC66A3 | 3' UTR | 0.02 | up |
| chr2 | 206046570 | 206056905 | 10336 | ENSG00000114933 | protein_coding | INO80D | 5' UTR | 0.02 | up |
| chr16 | 16321073 | 16321313 | 241 | ENSG00000183889 | processed_transcript | AC138969 | Exon | 0.02 | up |
| chr12 | 110128961 | 110134988 | 6028 | ENSG00000122970 | nonsense_mediated_decay | IFT81 | Exon | 0.02 | up |
| chr9 | 94568471 | 94568952 | 482 | ENSG00000231806 | lncRNA | PCAT7 | Exon | 0.02 | up |
| chr15 | 50354958 | 50355049 | 92 | ENSG00000244879 | lncRNA | GABPB1-AS1 | Exon | 0.02 | up |
| chr13 | 110614538 | 110614836 | 299 | ENSG00000275880 | lncRNA | AL139385 | Exon | 0.02 | up |
| chr17 | 41514674 | 41514855 | 182 | ENSG00000171346 | retained_intron | KRT15 | Exon | 0.02 | up |
| chr4 | 128107946 | 128108247 | 302 | ENSG00000138709 | nonsense_mediated_decay | LARP1B | 3' UTR | 0.02 | down |
| chr16 | 11797736 | 11798275 | 540 | ENSG00000277369 | lncRNA | AC010654 | Exon | 0.02 | down |
| chr2 | 203193803 | 203201625 | 7823 | ENSG00000204196 | processed_pseudogene | RPL12P16 | Exon | 0.02 | down |
| chr1 | 99688835 | 99689972 | 1138 | ENSG00000099260 | retained_intron | PALMD | 3' UTR | 0.02 | down |
| chr6 | 29630013 | 29630553 | 541 | ENSG00000204681 | nonsense_mediated_decay | GABBR1 | Exon | 0.02 | down |
| chr1 | 233054279 | 233054579 | 301 | ENSG00000135749 | retained_intron | PCNX2 | Exon | 0.02 | down |
| chr17 | 76087691 | 76087752 | 62 | ENSG00000182473 | retained_intron | EXOC7 | 3' UTR | 0.02 | down |
| chr4 | 74158229 | 74160303 | 2075 | ENSG00000269559 | lncRNA | AC093677 | 5' UTR | 0.02 | down |
| chr10 | 12120827 | 12121935 | 1109 | ENSG00000181192 | retained_intron | DHTKD1 | 3' UTR | 0.02 | down |
| chr7 | 123541760 | 123545661 | 3902 | ENSG00000128609 | retained_intron | NDUFA5 | 3' UTR | 0.02 | down |
| chr22 | 24541382 | 24541622 | 241 | ENSG00000138867 | processed_transcript | GUCD1 | 3' UTR | 0.02 | down |
| chr16 | 31489559 | 31489680 | 122 | ENSG00000260740 | lncRNA | AC026471 | 3' UTR | 0.02 | up |
| chr20 | 31547527 | 31548081 | 555 | ENSG00000101294 | processed_transcript | HM13 | 3' UTR | 0.02 | up |
| chr3 | 47083808 | 47097963 | 14156 | ENSG00000181555 | processed_transcript | SETD2 | 3' UTR | 0.02 | up |
| chr7 | 27197288 | 27197617 | 330 | ENSG00000106031 | processed_transcript | HOXA13 | 3' UTR | 0.02 | up |
| chr14 | 37172124 | 37172539 | 416 | ENSG00000183032 | protein_coding | SLC25A21 | Exon | 0.02 | up |
| chr19 | 47260360 | 47266319 | 5960 | ENSG00000105321 | retained_intron | CCDC9 | Exon | 0.02 | up |
| chr21 | 33543037 | 33546500 | 3464 | ENSG00000159131 | protein_coding | GART | 5' UTR | 0.02 | up |
| chr16 | 56683987 | 56684196 | 210 | ENSG00000187193 | nonsense_mediated_decay | MT1X | 3' UTR | 0.02 | up |
| chr5 | 473822 | 475271 | 1450 | ENSG00000225138 | lncRNA | SLC9A3-AS1 | Exon | 0.02 | up |
| chr16 | 58683959 | 58684200 | 242 | ENSG00000103042 | nonsense_mediated_decay | SLC38A7 | 5' UTR | 0.02 | up |
| chr17 | 4965894 | 4966513 | 620 | ENSG00000091640 | processed_transcript | SPAG7 | 5' UTR | 0.02 | up |
| chr15 | 25079057 | 25079149 | 93 | ENSG00000207137 | snoRNA | SNORD116-13 | Exon | 0.02 | up |
| chr1 | 42747322 | 42747443 | 122 | ENSG00000117385 | retained_intron | P3H1 | Exon | 0.02 | up |
| chr7 | 131502478 | 131502659 | 182 | ENSG00000128567 | retained_intron | PODXL | 3' UTR | 0.02 | up |
| chr7 | 102375883 | 102380603 | 4721 | ENSG00000128563 | protein_coding | PRKRIP1 | 5' UTR | 0.02 | up |
| chr19 | 34221508 | 34221748 | 241 | ENSG00000257103 | retained_intron | LSM14A | Exon | 0.02 | up |
| chr19 | 54094697 | 54095204 | 508 | ENSG00000170909 | protein_coding | OSCAR | 3' UTR | 0.02 | up |
| chr6 | 27452109 | 27452556 | 448 | ENSG00000096654 | protein_coding | ZNF184 | Exon | 0.02 | up |
| chr19 | 1782254 | 1782644 | 391 | ENSG00000130270 | nonsense_mediated_decay | ATP8B3 | 3' UTR | 0.02 | up |
| chr17 | 40630748 | 40631018 | 271 | ENSG00000073584 | retained_intron | SMARCE1 | 3' UTR | 0.02 | down |
| chr17 | 59107132 | 59107341 | 210 | ENSG00000224738 | lncRNA | AC099850 | Exon | 0.02 | down |
| chr11 | 72290555 | 72290915 | 361 | ENSG00000162129 | processed_transcript | CLPB | 3' UTR | 0.02 | down |
| chr5 | 141422034 | 141423772 | 1739 | ENSG00000253873 | protein_coding | PCDHGA11 | 3' UTR | 0.02 | down |
| chr19 | 1438357 | 1438595 | 239 | ENSG00000115268 | protein_coding | RPS15 | 5' UTR | 0.02 | down |
| chr19 | 13099152 | 13099603 | 452 | ENSG00000104903 | retained_intron | LYL1 | 3' UTR | 0.02 | down |
| chr3 | 50349030 | 50349091 | 62 | ENSG00000114388 | retained_intron | NPRL2 | Exon | 0.02 | down |
| chr1 | 39743287 | 39745414 | 2128 | ENSG00000084072 | processed_transcript | PPIE | 3' UTR | 0.02 | down |
| chr19 | 36489678 | 36490774 | 1097 | ENSG00000186017 | protein_coding | ZNF566 | Exon | 0.02 | up |
| chr11 | 118471768 | 118472908 | 1141 | ENSG00000118058 | retained_intron | KMT2A | 5' UTR | 0.02 | up |
| chr3 | 12489960 | 12503653 | 13694 | ENSG00000154743 | retained_intron | TSEN2 | Exon | 0.02 | up |
| chr14 | 102050625 | 102050806 | 182 | ENSG00000197102 | retained_intron | DYNC1H1 | 3' UTR | 0.02 | up |
| chr1 | 223795937 | 223799948 | 4012 | ENSG00000143514 | nonsense_mediated_decay | TP53BP2 | 3' UTR | 0.02 | up |
| chr5 | 132486512 | 132559294 | 72783 | ENSG00000113522 | protein_coding | RAD50 | 3' UTR | 0.02 | up |
| chr6 | 31541908 | 31542149 | 242 | ENSG00000198563 | retained_intron | DDX39B | 3' UTR | 0.02 | up |
| chr8 | 144999584 | 145000437 | 854 | ENSG00000196922 | transcribed_unprocessed_pseudogene | ZNF252P | Exon | 0.02 | up |
| chr19 | 51581877 | 51587728 | 5852 | ENSG00000105497 | retained_intron | ZNF175 | Exon | 0.02 | up |
| chr12 | 45849746 | 45850486 | 741 | ENSG00000189079 | protein_coding | ARID2 | Exon | 0.02 | up |
| chr12 | 103980544 | 103980949 | 406 | ENSG00000139372 | nonsense_mediated_decay | TDG | 5' UTR | 0.02 | up |
| chr15 | 72586136 | 72586436 | 301 | ENSG00000283798 | miRNA | MIR630 | 3' UTR | 0.02 | up |
| chr3 | 49642677 | 49643007 | 331 | ENSG00000164061 | retained_intron | BSN | Exon | 0.02 | up |
| chr2 | 113205653 | 113205774 | 122 | ENSG00000189223 | lncRNA | PAX8-AS1 | 3' UTR | 0.02 | down |
| chr4 | 52602003 | 52610556 | 8554 | ENSG00000109189 | retained_intron | USP46 | 3' UTR | 0.02 | down |
| chr19 | 7683668 | 7683878 | 211 | ENSG00000181029 | protein_coding | TRAPPC5 | 3' UTR | 0.02 | down |
| chr15 | 84201827 | 84201918 | 92 | ENSG00000225151 | processed_transcript | GOLGA2P7 | Exon | 0.02 | down |
| chr12 | 68841519 | 68842918 | 1400 | ENSG00000135679 | processed_transcript | MDM2 | 3' UTR | 0.02 | down |
| chr21 | 44416730 | 44425272 | 8543 | ENSG00000142185 | protein_coding | TRPM2 | Exon | 0.02 | down |
| chr10 | 8074095 | 8074513 | 419 | ENSG00000107485 | processed_transcript | GATA3 | 3' UTR | 0.02 | down |
| chr11 | 60935422 | 60935603 | 182 | ENSG00000006118 | protein_coding | TMEM132A | 3' UTR | 0.02 | down |
| chr13 | 52461085 | 52465565 | 4481 | ENSG00000136108 | protein_coding | CKAP2 | 3' UTR | 0.02 | down |
| chr10 | 75401703 | 75401764 | 62 | ENSG00000165655 | protein_coding | ZNF503 | 5' UTR | 0.02 | down |
| chr4 | 2965259 | 2966219 | 961 | ENSG00000125388 | protein_coding | GRK4 | 3' UTR | 0.02 | down |
| chr1 | 2181853 | 2182511 | 659 | ENSG00000182873 | lncRNA | PRKCZ-AS1 | Exon | 0.02 | down |
| chr17 | 57680311 | 57680641 | 331 | ENSG00000153944 | protein_coding | MSI2 | 3' UTR | 0.02 | down |
| X | 100832783 | 100833383 | 601 | ENSG00000101811 | protein_coding | CSTF2 | Exon | 0.02 | up |
| chr2 | 238098362 | 238098633 | 272 | ENSG00000144488 | protein_coding | ESPNL | 3' UTR | 0.02 | up |
| chr5 | 6746245 | 6746396 | 152 | ENSG00000112941 | retained_intron | TENT4A | Exon | 0.02 | up |
| chr1 | 56494760 | 56495090 | 331 | ENSG00000284686 | nonsense_mediated_decay | AC119674 | 3' UTR | 0.02 | up |
| chr17 | 46196375 | 46196636 | 262 | ENSG00000214401 | lncRNA | KANSL1-AS1 | Exon | 0.02 | up |
| chr7 | 96120638 | 96120758 | 121 | ENSG00000004864 | processed_transcript | SLC25A13 | 3' UTR | 0.02 | up |
| chr1 | 222687031 | 222693839 | 6809 | ENSG00000186063 | processed_transcript | AIDA | Exon | 0.02 | up |
| chr2 | 207581792 | 207582123 | 332 | ENSG00000144401 | processed_transcript | METTL21A | 3' UTR | 0.02 | down |
| chr15 | 75349140 | 75349410 | 271 | ENSG00000140398 | retained_intron | NEIL1 | Exon | 0.02 | down |
| chr17 | 45514190 | 45517990 | 3801 | ENSG00000214425 | processed_transcript | LRRC37A4P | Exon | 0.02 | down |
| chr12 | 122950778 | 122951788 | 1011 | ENSG00000150967 | protein_coding | ABCB9 | 5' UTR | 0.02 | down |
| chr14 | 102037948 | 102038339 | 392 | ENSG00000197102 | retained_intron | DYNC1H1 | Exon | 0.02 | down |
| chr2 | 231108727 | 231109088 | 362 | ENSG00000173692 | processed_transcript | PSMD1 | Exon | 0.02 | down |
| chr6 | 7339246 | 7339306 | 61 | ENSG00000220472 | processed_pseudogene | AL139095 | Exon | 0.02 | down |
| chr7 | 128654431 | 128654722 | 292 | ENSG00000243679 | processed_pseudogene | AC018638 | Exon | 0.02 | down |
| chr12 | 49842897 | 49843106 | 210 | ENSG00000186666 | protein_coding | BCDIN3D | 5' UTR | 0.02 | down |
| chr20 | 44589798 | 44598726 | 8929 | ENSG00000168734 | protein_coding | PKIG | 5' UTR | 0.02 | down |
| chr5 | 175527852 | 175528423 | 572 | ENSG00000164466 | retained_intron | SFXN1 | 3' UTR | 0.02 | down |
| chr16 | 3408380 | 3409364 | 985 | ENSG00000103343 | protein_coding | ZNF174 | 3' UTR | 0.02 | down |
| chr8 | 98156352 | 98158276 | 1925 | ENSG00000104356 | retained_intron | POP1 | 3' UTR | 0.02 | up |
| chr17 | 61406469 | 61406770 | 302 | ENSG00000121068 | processed_transcript | TBX2 | Exon | 0.02 | up |
| chr19 | 19507017 | 19507257 | 241 | ENSG00000186010 | nonsense_mediated_decay | NDUFA13 | 3' UTR | 0.02 | up |
| chr11 | 64859810 | 64859871 | 62 | ENSG00000110047 | retained_intron | EHD1 | Exon | 0.02 | up |
| chr21 | 46235132 | 46235373 | 242 | ENSG00000160285 | protein_coding | LSS | 3' UTR | 0.02 | up |
| chr11 | 108140078 | 108142623 | 2546 | ENSG00000075239 | retained_intron | ACAT1 | 3' UTR | 0.02 | up |
| chr15 | 63623765 | 63623856 | 92 | ENSG00000103657 | nonsense_mediated_decay | HERC1 | 3' UTR | 0.02 | up |
| chr16 | 18829501 | 18830032 | 532 | ENSG00000157106 | retained_intron | SMG1 | Exon | 0.02 | up |
| chr6 | 28085564 | 28085804 | 241 | ENSG00000197279 | protein_coding | ZNF165 | Exon | 0.02 | up |
| chr8 | 12722861 | 12723221 | 361 | ENSG00000154359 | retained_intron | LONRF1 | 3' UTR | 0.02 | down |
| chr7 | 3821493 | 3951735 | 130243 | ENSG00000146555 | protein_coding | SDK1 | Exon | 0.02 | down |
| chr2 | 232582139 | 232584423 | 2285 | ENSG00000237126 | lncRNA | AC073254 | Exon | 0.02 | down |
| chr19 | 54122890 | 54123220 | 331 | ENSG00000237017 | lncRNA | AC245052 | Exon | 0.02 | down |
| chr12 | 125140604 | 125140934 | 331 | ENSG00000279233 | TEC | AC122688 | Exon | 0.02 | down |
| chr3 | 171460701 | 171461897 | 1197 | ENSG00000279673 | TEC | AC092919 | Exon | 0.02 | down |
| chr11 | 69367335 | 69367726 | 392 | ENSG00000260877 | lncRNA | AP005233 | Exon | 0.02 | down |
| chr14 | 102980873 | 102983618 | 2746 | ENSG00000198752 | protein_coding | CDC42BPB | Exon | 0.02 | down |
| chr10 | 70150819 | 70151000 | 182 | ENSG00000156521 | protein_coding | TYSND1 | 3' UTR | 0.02 | up |
| chr16 | 84060689 | 84061229 | 541 | ENSG00000140943 | retained_intron | MBTPS1 | Exon | 0.02 | up |
| chr4 | 150817177 | 150828489 | 11313 | ENSG00000198589 | protein_coding | LRBA | Exon | 0.02 | up |
| chr16 | 53934005 | 53935708 | 1704 | ENSG00000140718 | protein_coding | FTO | 3' UTR | 0.02 | up |
| chr1 | 155288354 | 155288803 | 450 | ENSG00000143630 | processed_transcript | HCN3 | 3' UTR | 0.02 | up |
| X | 48993545 | 48997986 | 4442 | ENSG00000068400 | nonsense_mediated_decay | GRIPAP1 | 5' UTR | 0.02 | up |
| chr6 | 31762430 | 31762581 | 152 | ENSG00000255152 | nonsense_mediated_decay | MSH5-SAPCD1 | 3' UTR | 0.02 | up |
| chr10 | 120906775 | 120907425 | 651 | ENSG00000120008 | processed_transcript | WDR11 | 3' UTR | 0.02 | up |
| chr14 | 39399519 | 39399759 | 241 | ENSG00000259100 | processed_pseudogene | AL157791 | 3' UTR | 0.02 | up |
| chr4 | 87140693 | 87141024 | 332 | ENSG00000172493 | retained_intron | AFF1 | 3' UTR | 0.02 | down |
| chr5 | 14871567 | 14871718 | 152 | ENSG00000154122 | nonsense_mediated_decay | ANKH | 5' UTR | 0.02 | down |
| chr8 | 492478 | 494526 | 2049 | ENSG00000180190 | processed_transcript | TDRP | Exon | 0.02 | down |
| chr11 | 108510801 | 108511311 | 511 | ENSG00000178202 | protein_coding | POGLUT3 | Exon | 0.02 | down |
| chr7 | 27143384 | 27143681 | 298 | ENSG00000106004 | protein_coding | HOXA5 | 5' UTR | 0.02 | down |
| chr16 | 228053 | 228294 | 242 | ENSG00000007392 | protein_coding | LUC7L | 3' UTR | 0.02 | down |
| chr16 | 66943310 | 66963199 | 19890 | ENSG00000172828 | protein_coding | CES3 | 3' UTR | 0.02 | down |
| chr1 | 1625839 | 1626199 | 361 | ENSG00000197530 | nonsense_mediated_decay | MIB2 | Exon | 0.02 | down |
| chr19 | 35499135 | 35499931 | 797 | ENSG00000161249 | retained_intron | DMKN | 3' UTR | 0.02 | down |
| chr10 | 119455114 | 119455593 | 480 | ENSG00000198873 | retained_intron | GRK5 | 3' UTR | 0.02 | down |
| chr11 | 61362343 | 61362801 | 459 | ENSG00000149483 | retained_intron | TMEM138 | 5' UTR | 0.02 | down |
| chr14 | 21357302 | 21358043 | 742 | ENSG00000092201 | retained_intron | SUPT16H | Exon | 0.02 | down |
| chr8 | 23403971 | 23429299 | 25329 | ENSG00000134013 | protein_coding | LOXL2 | 3' UTR | 0.02 | down |
| chr16 | 50316454 | 50317085 | 632 | ENSG00000166164 | protein_coding | BRD7 | 3' UTR | 0.02 | down |
| chr17 | 5027110 | 5027501 | 392 | ENSG00000129250 | processed_transcript | KIF1C | 3' UTR | 0.02 | down |
| chr1 | 241867021 | 241879157 | 12137 | ENSG00000174371 | protein_coding | EXO1 | Exon | 0.02 | down |
| chr17 | 41853281 | 41854990 | 1710 | ENSG00000259623 | lncRNA | AC125257 | 3' UTR | 0.02 | down |
| chr22 | 38822397 | 38823125 | 729 | ENSG00000221890 | protein_coding | NPTXR | 3' UTR | 0.02 | up |
| chr6 | 49463428 | 49492311 | 28884 | ENSG00000031691 | protein_coding | CENPQ | 3' UTR | 0.02 | up |
| chr1 | 171568300 | 171574936 | 6637 | ENSG00000117523 | protein_coding | PRRC2C | Exon | 0.02 | up |
| chr10 | 122086396 | 122087146 | 751 | ENSG00000138162 | processed_transcript | TACC2 | Exon | 0.02 | up |
| chr5 | 149002686 | 149003286 | 601 | ENSG00000169247 | nonsense_mediated_decay | SH3TC2 | 3' UTR | 0.02 | up |
| chr3 | 42224449 | 42224750 | 302 | ENSG00000222872 | snRNA | RNU4-78P | 3' UTR | 0.02 | up |
| chr12 | 53486651 | 53486950 | 300 | ENSG00000139625 | retained_intron | MAP3K12 | Exon | 0.02 | up |
| chr6 | 27019922 | 27020252 | 331 | ENSG00000224843 | lncRNA | LINC00240 | Exon | 0.02 | up |
| chr9 | 106924201 | 106924472 | 272 | ENSG00000148143 | processed_transcript | ZNF462 | Exon | 0.02 | up |
| chr8 | 143866760 | 143867180 | 421 | ENSG00000261150 | protein_coding | EPPK1 | Exon | 0.02 | up |
| chr3 | 168043395 | 168095235 | 51841 | ENSG00000173905 | retained_intron | GOLIM4 | Exon | 0.02 | up |
| chr14 | 37401865 | 37423635 | 21771 | ENSG00000151338 | TEC | MIPOL1 | Exon | 0.02 | down |
| chr19 | 10315776 | 10316015 | 240 | ENSG00000167807 | nonsense_mediated_decay | AC011511 | 5' UTR | 0.02 | down |
| chr11 | 9807863 | 9808104 | 242 | ENSG00000246273 | lncRNA | SBF2-AS1 | Exon | 0.02 | down |
| chr1 | 197142737 | 197143308 | 572 | ENSG00000066279 | protein_coding | ASPM | Exon | 0.02 | down |
| chr16 | 50316457 | 50317087 | 631 | ENSG00000121281 | retained_intron | ADCY7 | 3' UTR | 0.02 | down |
| chr10 | 28611862 | 28614747 | 2886 | ENSG00000095787 | retained_intron | WAC | 3' UTR | 0.02 | down |
| chr15 | 84622014 | 84622373 | 360 | ENSG00000276278 | lncRNA | AC048382 | Exon | 0.02 | down |
| chr14 | 73716379 | 73716710 | 332 | ENSG00000176903 | protein_coding | PNMA1 | 3' UTR | 0.02 | down |
| chr15 | 58596932 | 58597622 | 691 | ENSG00000137845 | retained_intron | ADAM10 | 3' UTR | 0.02 | down |
| chr3 | 107808762 | 107809841 | 1080 | ENSG00000114439 | protein_coding | BBX | 3' UTR | 0.02 | down |
| chr15 | 45023146 | 45035846 | 12701 | ENSG00000140263 | protein_coding | SORD | 3' UTR | 0.02 | down |
| chr19 | 4254405 | 4267624 | 13220 | ENSG00000105248 | protein_coding | YJU2 | Exon | 0.02 | down |
| chr22 | 38729468 | 38729619 | 152 | ENSG00000100226 | nonsense_mediated_decay | GTPBP1 | Exon | 0.02 | down |
| chr15 | 75018840 | 75019079 | 240 | ENSG00000198794 | protein_coding | SCAMP5 | 3' UTR | 0.02 | up |
| chr5 | 90627351 | 90627862 | 512 | ENSG00000164199 | retained_intron | ADGRV1 | Exon | 0.02 | up |
| chr19 | 4180861 | 4182536 | 1676 | ENSG00000077463 | protein_coding | SIRT6 | 5' UTR | 0.02 | up |
| chr20 | 49889188 | 49889518 | 331 | ENSG00000197818 | processed_transcript | SLC9A8 | 3' UTR | 0.02 | up |
| chr11 | 128972631 | 128973410 | 780 | ENSG00000134909 | retained_intron | ARHGAP32 | 3' UTR | 0.02 | up |
| chr6 | 30340512 | 30341872 | 1361 | ENSG00000241370 | protein_coding | RPP21 | 3' UTR | 0.02 | up |
| chr8 | 125363314 | 125367120 | 3807 | ENSG00000156831 | processed_transcript | NSMCE2 | 3' UTR | 0.02 | up |
| chr16 | 16321095 | 16321306 | 212 | ENSG00000183889 | processed_transcript | AC138969 | Exon | 0.02 | up |
| chr16 | 31120195 | 31120574 | 380 | ENSG00000278133 | lncRNA | AC135050 | Exon | 0.02 | up |
| chr1 | 40271985 | 40280219 | 8235 | ENSG00000084073 | retained_intron | ZMPSTE24 | 3' UTR | 0.02 | up |
| chr6 | 149578903 | 149580604 | 1702 | ENSG00000055211 | protein_coding | GINM1 | Exon | 0.02 | up |
| chr17 | 46196403 | 46196635 | 233 | ENSG00000214401 | lncRNA | KANSL1-AS1 | Exon | 0.02 | up |
| chr20 | 28565109 | 28583030 | 17922 | ENSG00000282826 | transcribed_unprocessed_pseudogene | FRG1CP | Exon | 0.02 | down |
| chr11 | 59107214 | 59109856 | 2643 | ENSG00000189057 | retained_intron | FAM111B | 5' UTR | 0.02 | down |
| chr16 | 1655910 | 1656952 | 1043 | ENSG00000007545 | retained_intron | CRAMP1 | 3' UTR | 0.02 | down |
| chr7 | 156958829 | 156962171 | 3343 | ENSG00000146909 | retained_intron | NOM1 | Exon | 0.02 | down |
| chr4 | 27008778 | 27017726 | 8949 | ENSG00000109689 | retained_intron | STIM2 | Exon | 0.02 | down |
| chr1 | 202861753 | 202873635 | 11883 | ENSG00000234996 | processed_transcript | AC098934 | Exon | 0.02 | down |
| chr16 | 89971758 | 89972236 | 479 | ENSG00000177946 | transcribed_unitary_pseudogene | CENPBD1 | 5' UTR | 0.02 | down |
| chr7 | 92325985 | 92343182 | 17198 | ENSG00000001629 | nonsense_mediated_decay | ANKIB1 | 3' UTR | 0.02 | down |
| chr2 | 85537960 | 85538284 | 325 | ENSG00000286532 | lncRNA | PARTICL | Exon | 0.02 | down |
| chr12 | 109088246 | 109089681 | 1436 | ENSG00000189046 | protein_coding | ALKBH2 | 3' UTR | 0.02 | down |
| chr12 | 132704629 | 132704985 | 357 | ENSG00000247077 | protein_coding | PGAM5 | 3' UTR | 0.02 | down |
| chr19 | 4046207 | 4046807 | 601 | ENSG00000268670 | lncRNA | AC016586 | 3' UTR | 0.02 | up |
| chr16 | 1984876 | 1985055 | 180 | ENSG00000196408 | protein_coding | NOXO1 | Exon | 0.02 | up |
| chr7 | 44768654 | 44768745 | 92 | ENSG00000122515 | retained_intron | ZMIZ2 | 3' UTR | 0.02 | up |
| chr11 | 123627414 | 123627504 | 91 | ENSG00000023171 | retained_intron | GRAMD1B | 3' UTR | 0.02 | up |
| chr19 | 54449036 | 54449637 | 602 | ENSG00000167615 | protein_coding | LENG8 | 5' UTR | 0.02 | up |
| chr2 | 72465189 | 72492333 | 27145 | ENSG00000144036 | processed_transcript | EXOC6B | Exon | 0.02 | up |
| chr16 | 88862356 | 88862686 | 331 | ENSG00000167515 | retained_intron | TRAPPC2L | 3' UTR | 0.02 | up |
| chr3 | 128893594 | 128895338 | 1745 | ENSG00000177646 | processed_transcript | ACAD9 | 3' UTR | 0.02 | up |
| chr12 | 112075744 | 112087705 | 11962 | ENSG00000111300 | protein_coding | NAA25 | 3' UTR | 0.02 | up |
| chr8 | 67043078 | 67043438 | 361 | ENSG00000121022 | processed_transcript | COPS5 | 3' UTR | 0.02 | up |
| chr4 | 62818197 | 62818436 | 240 | ENSG00000180673 | processed_pseudogene | EXOC5P1 | Exon | 0.02 | up |
| chr13 | 95757701 | 95760064 | 2364 | ENSG00000276809 | lncRNA | AL138955 | Exon | 0.02 | up |
| chr16 | 3306394 | 3306635 | 242 | ENSG00000162086 | processed_transcript | ZNF75A | Exon | 0.02 | down |
| chr1 | 148532193 | 148532344 | 152 | ENSG00000274020 | lncRNA | LINC01138 | 3' UTR | 0.02 | down |
| chr6 | 32188746 | 32188897 | 152 | ENSG00000204304 | retained_intron | PBX2 | Exon | 0.02 | down |
| chr6 | 21231985 | 21232195 | 211 | ENSG00000145996 | processed_transcript | CDKAL1 | 3' UTR | 0.02 | down |
| chr12 | 112029021 | 112033469 | 4449 | ENSG00000111300 | retained_intron | NAA25 | 3' UTR | 0.02 | down |
| chr1 | 155868124 | 155868634 | 511 | ENSG00000132718 | protein_coding | SYT11 | Exon | 0.02 | down |
| chr21 | 26841303 | 26841513 | 211 | ENSG00000154734 | retained_intron | ADAMTS1 | Exon | 0.02 | down |
| chr1 | 100975524 | 100975944 | 421 | ENSG00000162695 | protein_coding | SLC30A7 | 3' UTR | 0.02 | down |
| chr7 | 74386487 | 74388823 | 2337 | ENSG00000106665 | retained_intron | CLIP2 | Exon | 0.02 | down |
| chr16 | 16131812 | 16131933 | 122 | ENSG00000103222 | retained_intron | ABCC1 | Exon | 0.02 | down |
| chr19 | 16549836 | 16550047 | 212 | ENSG00000127526 | processed_transcript | SLC35E1 | 3' UTR | 0.02 | down |
| chr16 | 70365103 | 70366235 | 1133 | ENSG00000168872 | retained_intron | DDX19A | 3' UTR | 0.02 | down |
| chr17 | 2387600 | 2388196 | 597 | ENSG00000070444 | retained_intron | MNT | 3' UTR | 0.02 | up |
| chr10 | 3134565 | 3144988 | 10424 | ENSG00000107959 | processed_transcript | PITRM1 | 3' UTR | 0.02 | up |
| chr16 | 590269 | 616677 | 26409 | ENSG00000197562 | nonsense_mediated_decay | RAB40C | 3' UTR | 0.02 | up |
| chr5 | 38990983 | 38996847 | 5865 | ENSG00000164327 | retained_intron | RICTOR | Exon | 0.02 | up |
| chr16 | 53324117 | 53325047 | 931 | ENSG00000177200 | nonsense_mediated_decay | CHD9 | 3' UTR | 0.02 | up |
| chr1 | 29097859 | 29109530 | 11672 | ENSG00000159023 | retained_intron | EPB41 | 3' UTR | 0.02 | up |
| chr19 | 57977501 | 57977771 | 271 | ENSG00000177025 | protein_coding | C19orf18 | 3' UTR | 0.02 | up |
| chr17 | 28909474 | 28909715 | 242 | ENSG00000109118 | processed_transcript | PHF12 | 3' UTR | 0.02 | down |
| chr19 | 1205807 | 1205928 | 122 | ENSG00000118046 | protein_coding | STK11 | 5' UTR | 0.02 | down |
| chr21 | 41445458 | 41449751 | 4294 | ENSG00000157601 | retained_intron | MX1 | Exon | 0.02 | down |
| chr7 | 127582990 | 127584281 | 1292 | ENSG00000179562 | protein_coding | GCC1 | Exon | 0.02 | down |
| chr1 | 26876191 | 26876581 | 391 | ENSG00000142751 | retained_intron | GPN2 | 3' UTR | 0.02 | down |
| chr1 | 224419562 | 224429312 | 9751 | ENSG00000162923 | processed_transcript | WDR26 | 3' UTR | 0.02 | down |
| chr1 | 28282013 | 28282461 | 449 | ENSG00000130766 | protein_coding | SESN2 | 3' UTR | 0.02 | up |
| chr19 | 10469091 | 10469570 | 480 | ENSG00000065989 | retained_intron | PDE4A | 3' UTR | 0.02 | up |
| chr19 | 50289552 | 50301839 | 12288 | ENSG00000105357 | retained_intron | MYH14 | 3' UTR | 0.02 | up |
| chr17 | 37084991 | 37085321 | 331 | ENSG00000278540 | protein_coding | ACACA | 3' UTR | 0.02 | up |
| chr14 | 23476065 | 23477229 | 1165 | ENSG00000129460 | retained_intron | NGDN | Exon | 0.02 | up |
| chr16 | 89420074 | 89422226 | 2153 | ENSG00000261692 | lncRNA | AC092120 | Exon | 0.02 | up |
| chr3 | 131383147 | 131383537 | 391 | ENSG00000198585 | protein_coding | NUDT16 | 3' UTR | 0.02 | up |
| chr3 | 179378341 | 179378551 | 211 | ENSG00000171109 | retained_intron | MFN1 | 3' UTR | 0.02 | up |
| chr4 | 27017845 | 27019530 | 1686 | ENSG00000109689 | retained_intron | STIM2 | 3' UTR | 0.02 | up |
| chr7 | 105875110 | 105876514 | 1405 | ENSG00000146776 | protein_coding | ATXN7L1 | 5' UTR | 0.02 | up |
| chr14 | 50159543 | 50159994 | 452 | ENSG00000100485 | protein_coding | SOS2 | Exon | 0.02 | up |
| chr8 | 143866734 | 143867184 | 451 | ENSG00000261150 | protein_coding | EPPK1 | Exon | 0.02 | up |
| X | 52065821 | 52066559 | 739 | ENSG00000187243 | retained_intron | MAGED4B | Exon | 0.02 | up |
| chr16 | 14975167 | 14976890 | 1724 | ENSG00000179889 | protein_coding | PDXDC1 | 5' UTR | 0.02 | up |
| chr14 | 58266804 | 58267015 | 212 | ENSG00000257621 | lncRNA | PSMA3-AS1 | Exon | 0.02 | up |
| chr16 | 28487937 | 28488328 | 392 | ENSG00000188603 | retained_intron | CLN3 | Exon | 0.02 | down |
| chr7 | 107202661 | 107203021 | 361 | ENSG00000272072 | lncRNA | AC004492 | 3' UTR | 0.02 | down |
| chr9 | 134158828 | 134159098 | 271 | ENSG00000273473 | lncRNA | BX649601 | 3' UTR | 0.02 | down |
| chr6 | 85611401 | 85611581 | 181 | ENSG00000271793 | nonsense_mediated_decay | AL589666 | 3' UTR | 0.02 | down |
| chr16 | 89971781 | 89972232 | 452 | ENSG00000177946 | transcribed_unitary_pseudogene | CENPBD1 | 5' UTR | 0.02 | down |
| chr17 | 82293715 | 82294313 | 599 | ENSG00000260563 | lncRNA | AC132872 | Exon | 0.02 | down |
| chr7 | 4770631 | 4770782 | 152 | ENSG00000242802 | nonsense_mediated_decay | AP5Z1 | 3' UTR | 0.02 | down |
| chr2 | 68130655 | 68131777 | 1123 | ENSG00000243667 | protein_coding | WDR92 | 3' UTR | 0.02 | down |
| chr10 | 24714343 | 24722487 | 8145 | ENSG00000107863 | protein_coding | ARHGAP21 | 5' UTR | 0.02 | down |
| chr2 | 58221986 | 58241283 | 19298 | ENSG00000115392 | protein_coding | FANCL | 5' UTR | 0.02 | down |
| chr9 | 127943299 | 127943390 | 92 | ENSG00000167106 | retained_intron | FAM102A | Exon | 0.02 | down |
| chr2 | 61017614 | 61018742 | 1129 | ENSG00000162927 | retained_intron | PUS10 | 3' UTR | 0.02 | up |
| chr11 | 12138439 | 12161475 | 23037 | ENSG00000133816 | protein_coding | MICAL2 | 5' UTR | 0.02 | up |
| chr10 | 29458547 | 29462325 | 3779 | ENSG00000197321 | processed_transcript | SVIL | Exon | 0.02 | up |
| chr2 | 223877138 | 223877407 | 270 | ENSG00000085449 | processed_transcript | WDFY1 | 3' UTR | 0.02 | up |
| chr6 | 150061261 | 150061860 | 600 | ENSG00000131019 | protein_coding | ULBP3 | 3' UTR | 0.02 | up |
| chr3 | 33796069 | 33796400 | 332 | ENSG00000271643 | lncRNA | AC112220 | Exon | 0.02 | up |
| chr15 | 99136322 | 99136743 | 422 | ENSG00000103852 | retained_intron | TTC23 | 3' UTR | 0.02 | up |
| chr17 | 17219760 | 17220240 | 481 | ENSG00000154803 | retained_intron | FLCN | Exon | 0.02 | down |
| chr15 | 41838829 | 41838920 | 92 | ENSG00000243708 | protein_coding | PLA2G4B | 5' UTR | 0.02 | down |
| chr3 | 45417560 | 45421277 | 3718 | ENSG00000011376 | protein_coding | LARS2 | 3' UTR | 0.02 | down |
| chr7 | 98878515 | 98881208 | 2694 | ENSG00000196367 | protein_coding | TRRAP | 5' UTR | 0.02 | down |
| chr1 | 10431209 | 10440187 | 8979 | ENSG00000175279 | retained_intron | CENPS | 3' UTR | 0.02 | down |
| chr16 | 2501454 | 2501814 | 361 | ENSG00000162065 | processed_transcript | TBC1D24 | 3' UTR | 0.02 | down |
| chr3 | 25601149 | 25609309 | 8161 | ENSG00000077097 | processed_transcript | TOP2B | 3' UTR | 0.02 | down |
| chr2 | 151802889 | 151806924 | 4036 | ENSG00000162980 | processed_transcript | ARL5A | 3' UTR | 0.02 | down |
| chr16 | 48354198 | 48354648 | 451 | ENSG00000102910 | retained_intron | LONP2 | 3' UTR | 0.02 | down |
| chr3 | 23967954 | 23978020 | 10067 | ENSG00000174738 | retained_intron | NR1D2 | 3' UTR | 0.02 | down |
| chr15 | 91002155 | 91005425 | 3271 | ENSG00000184056 | retained_intron | VPS33B | 3' UTR | 0.02 | up |
| chr19 | 1473890 | 1474040 | 151 | ENSG00000119559 | retained_intron | C19orf25 | 3' UTR | 0.02 | up |
| chr3 | 51662692 | 51671260 | 8569 | ENSG00000164081 | protein_coding | TEX264 | 3' UTR | 0.02 | up |
| chr19 | 19570234 | 19570654 | 421 | ENSG00000105717 | retained_intron | PBX4 | 3' UTR | 0.02 | up |
| chr7 | 105062193 | 105063474 | 1282 | ENSG00000005483 | retained_intron | KMT2E | 5' UTR | 0.02 | up |
| chr16 | 84760226 | 84764116 | 3891 | ENSG00000103194 | retained_intron | USP10 | 3' UTR | 0.02 | up |
| chr6 | 49554892 | 49555253 | 362 | ENSG00000197261 | retained_intron | C6orf141 | 3' UTR | 0.02 | up |
| chr1 | 183935847 | 183936626 | 780 | ENSG00000198756 | processed_transcript | COLGALT2 | 3' UTR | 0.02 | up |
| chr5 | 141863942 | 141864782 | 841 | ENSG00000156453 | processed_transcript | PCDH1 | 3' UTR | 0.02 | up |
| chr7 | 93101517 | 93102177 | 661 | ENSG00000205413 | protein_coding | SAMD9 | Exon | 0.02 | down |
| chr7 | 5477442 | 5477652 | 211 | ENSG00000207973 | miRNA | MIR589 | 3' UTR | 0.02 | down |
| chr17 | 76570616 | 76570766 | 151 | ENSG00000070731 | nonsense_mediated_decay | ST6GALNAC2 | 3' UTR | 0.02 | down |
| chr6 | 136278063 | 136278424 | 362 | ENSG00000029363 | nonsense_mediated_decay | BCLAF1 | Exon | 0.02 | down |
| chr14 | 35273452 | 35273813 | 362 | ENSG00000100902 | protein_coding | PSMA6 | 3' UTR | 0.02 | down |
| chr4 | 1930751 | 1939421 | 8671 | ENSG00000109685 | processed_transcript | NSD2 | Exon | 0.02 | down |
| chr17 | 58083418 | 58087087 | 3670 | ENSG00000264364 | protein_coding | DYNLL2 | 5' UTR | 0.02 | down |
| chr17 | 81702042 | 81702432 | 391 | ENSG00000262049 | lncRNA | AC139530 | Exon | 0.02 | down |
| chr16 | 23480758 | 23491738 | 10981 | ENSG00000103365 | processed_transcript | GGA2 | 3' UTR | 0.02 | up |
| chr11 | 73228137 | 73234313 | 6177 | ENSG00000260401 | lncRNA | AP002761 | 5' UTR | 0.02 | up |
| chr16 | 8811114 | 8842305 | 31192 | ENSG00000140650 | processed_transcript | PMM2 | 3' UTR | 0.02 | up |
| MT | 12366 | 12753 | 388 | ENSG00000198786 | protein_coding | MT-ND5 | Exon | 0.02 | up |
| chr18 | 57600864 | 57601104 | 241 | ENSG00000134440 | retained_intron | NARS1 | 3' UTR | 0.02 | up |
| chr1 | 183515932 | 183517651 | 1720 | ENSG00000116698 | processed_transcript | SMG7 | 5' UTR | 0.02 | up |
| chr12 | 117968090 | 117968541 | 452 | ENSG00000171435 | protein_coding | KSR2 | 5' UTR | 0.02 | up |
| chr8 | 56301290 | 56304013 | 2724 | ENSG00000170786 | protein_coding | SDR16C5 | 3' UTR | 0.02 | up |
| chr21 | 41376861 | 41377884 | 1024 | ENSG00000183486 | protein_coding | MX2 | 5' UTR | 0.02 | up |
| chr2 | 70031710 | 70031891 | 182 | ENSG00000179818 | lncRNA | PCBP1-AS1 | Exon | 0.02 | down |
| chr17 | 18781926 | 18782166 | 241 | ENSG00000171928 | protein_coding | TVP23B | 5' UTR | 0.02 | down |
| chr2 | 156329972 | 156330151 | 180 | ENSG00000153234 | protein_coding | NR4A2 | 5' UTR | 0.02 | down |
| chr1 | 201468939 | 201469237 | 299 | ENSG00000174307 | protein_coding | PHLDA3 | 5' UTR | 0.02 | down |
| chr17 | 48591818 | 48592174 | 357 | ENSG00000120093 | protein_coding | HOXB3 | 3' UTR | 0.02 | down |
| chr4 | 1812107 | 1812408 | 302 | ENSG00000168924 | retained_intron | LETM1 | 3' UTR | 0.02 | down |
| chr15 | 80137583 | 80137914 | 332 | ENSG00000086666 | retained_intron | ZFAND6 | 3' UTR | 0.02 | down |
| chr9 | 20907237 | 20923671 | 16435 | ENSG00000188352 | protein_coding | FOCAD | Exon | 0.02 | down |
| chr22 | 47175310 | 47175699 | 390 | ENSG00000280312 | TEC | Z83836 | 3' UTR | 0.02 | up |
| chr1 | 42931150 | 42931301 | 152 | ENSG00000117394 | retained_intron | SLC2A1 | Exon | 0.02 | up |
| chr9 | 134031292 | 134031742 | 451 | ENSG00000169925 | processed_transcript | BRD3 | 3' UTR | 0.02 | up |
| chr1 | 114567915 | 114575746 | 7832 | ENSG00000116752 | protein_coding | BCAS2 | 3' UTR | 0.02 | up |
| chr12 | 110386368 | 110386608 | 241 | ENSG00000196510 | retained_intron | ANAPC7 | 3' UTR | 0.02 | up |
| chr12 | 112162077 | 112162348 | 272 | ENSG00000173064 | processed_transcript | HECTD4 | 3' UTR | 0.02 | up |
| chr6 | 7287958 | 7288109 | 152 | ENSG00000124783 | processed_transcript | SSR1 | 3' UTR | 0.02 | up |
| chr4 | 158713131 | 158719201 | 6071 | ENSG00000171497 | nonsense_mediated_decay | PPID | 3' UTR | 0.02 | up |
| chr16 | 22483118 | 22483526 | 409 | ENSG00000237296 | processed_transcript | SMG1P1 | Exon | 0.02 | up |
| chr16 | 89870929 | 89871079 | 151 | ENSG00000141002 | nonsense_mediated_decay | TCF25 | 3' UTR | 0.02 | up |
| chr17 | 56791971 | 56795138 | 3168 | ENSG00000214226 | retained_intron | C17orf67 | 3' UTR | 0.02 | up |
| chr1 | 244844728 | 244845027 | 300 | ENSG00000203667 | retained_intron | COX20 | 3' UTR | 0.02 | up |
| chr4 | 99900710 | 99906092 | 5383 | ENSG00000164031 | nonsense_mediated_decay | DNAJB14 | 3' UTR | 0.02 | up |
| chr1 | 214657372 | 214663763 | 6392 | ENSG00000117724 | processed_transcript | CENPF | Exon | 0.02 | up |
| chr8 | 91066727 | 91067357 | 631 | ENSG00000253738 | lncRNA | OTUD6B-AS1 | Exon | 0.02 | down |
| chr2 | 230909712 | 230910102 | 391 | ENSG00000230385 | lncRNA | AC012507 | Exon | 0.02 | down |
| chr14 | 71307277 | 71311271 | 3995 | ENSG00000259146 | lncRNA | AC005476 | Exon | 0.02 | down |
| chr19 | 57544572 | 57547344 | 2773 | ENSG00000251369 | protein_coding | ZNF550 | 3' UTR | 0.02 | down |
| chr16 | 75109526 | 75110036 | 511 | ENSG00000186187 | retained_intron | ZNRF1 | 3' UTR | 0.02 | down |
| chr14 | 35273436 | 35273825 | 390 | ENSG00000100902 | protein_coding | PSMA6 | 3' UTR | 0.02 | down |
| chr22 | 39078940 | 39079239 | 300 | ENSG00000239713 | retained_intron | APOBEC3G | Exon | 0.02 | up |
| chr5 | 107380239 | 107381376 | 1138 | ENSG00000184349 | processed_transcript | EFNA5 | 3' UTR | 0.02 | up |
| chr15 | 77114769 | 77133684 | 18916 | ENSG00000140391 | protein_coding | TSPAN3 | Exon | 0.02 | up |
| chr2 | 169081530 | 169095897 | 14368 | ENSG00000073737 | protein_coding | DHRS9 | 3' UTR | 0.02 | up |
| chr7 | 92457085 | 92457804 | 720 | ENSG00000244055 | lncRNA | AC007566 | 3' UTR | 0.02 | up |
| chr1 | 40514517 | 40515057 | 541 | ENSG00000227278 | lncRNA | AL603839 | Exon | 0.02 | up |
| chr1 | 236551273 | 236551963 | 691 | ENSG00000116977 | retained_intron | LGALS8 | 3' UTR | 0.02 | up |
| chr6 | 3177333 | 3177537 | 205 | ENSG00000216819 | unprocessed_pseudogene | TUBB2BP1 | Exon | 0.02 | down |
| chr20 | 46710129 | 46714517 | 4389 | ENSG00000197496 | processed_transcript | SLC2A10 | 3' UTR | 0.02 | down |
| chr5 | 177619088 | 177627393 | 8306 | ENSG00000246596 | processed_transcript | AC139795 | Exon | 0.02 | down |
| chr2 | 14649344 | 14650244 | 901 | ENSG00000162981 | processed_transcript | LRATD1 | Exon | 0.02 | down |
| chr5 | 141216044 | 141216733 | 690 | ENSG00000187372 | protein_coding | PCDHB13 | 3' UTR | 0.02 | down |
| chr12 | 122207661 | 122207840 | 180 | ENSG00000184047 | retained_intron | DIABLO | 3' UTR | 0.02 | down |
| chr19 | 16549830 | 16550040 | 211 | ENSG00000127526 | processed_transcript | SLC35E1 | 3' UTR | 0.02 | down |
| chr2 | 218633342 | 218633985 | 644 | ENSG00000115556 | retained_intron | PLCD4 | Exon | 0.02 | up |
| chr20 | 44303585 | 44303765 | 181 | ENSG00000197296 | protein_coding | FITM2 | 3' UTR | 0.02 | up |
| chr11 | 68050739 | 68052254 | 1516 | ENSG00000255031 | lncRNA | AP002807 | 3' UTR | 0.02 | up |
| chr16 | 22075495 | 22076095 | 601 | ENSG00000185716 | retained_intron | MOSMO | 3' UTR | 0.02 | up |
| chr3 | 120781965 | 120782383 | 419 | ENSG00000213371 | processed_pseudogene | NAP1L1P3 | 3' UTR | 0.02 | up |
| chr1 | 93553106 | 93553406 | 301 | ENSG00000137942 | retained_intron | FNBP1L | 3' UTR | 0.02 | up |
| chr19 | 58305408 | 58306380 | 973 | ENSG00000142396 | protein_coding | ERVK3-1 | 3' UTR | 0.02 | up |
| chr15 | 43813012 | 43817321 | 4310 | ENSG00000140259 | retained_intron | MFAP1 | Exon | 0.02 | up |
| chr6 | 142751618 | 142752247 | 630 | ENSG00000010818 | processed_transcript | HIVEP2 | 3' UTR | 0.02 | up |
| chr20 | 10412595 | 10412836 | 242 | ENSG00000285723 | protein_coding | AL034430 | Exon | 0.02 | up |
| chr3 | 47120269 | 47121259 | 991 | ENSG00000181555 | protein_coding | SETD2 | 5' UTR | 0.02 | up |
| chr10 | 11501168 | 11532255 | 31088 | ENSG00000148429 | protein_coding | USP6NL | 5' UTR | 0.02 | up |
| chr16 | 3651524 | 3651945 | 422 | ENSG00000213918 | protein_coding | DNASE1 | 5' UTR | 0.02 | up |
| X | 102884148 | 102884508 | 361 | ENSG00000239407 | lncRNA | Z68871 | Exon | 0.02 | up |
| chr17 | 39405881 | 39406233 | 353 | ENSG00000266469 | lncRNA | AC005288 | Exon | 0.02 | up |
| chr17 | 28604934 | 28605205 | 272 | ENSG00000167524 | retained_intron | RSKR | 3' UTR | 0.02 | down |
| chr19 | 52881724 | 52890245 | 8522 | ENSG00000182986 | protein_coding | ZNF320 | Exon | 0.02 | down |
| chr7 | 75537909 | 75539337 | 1429 | ENSG00000127957 | processed_transcript | PMS2P3 | 3' UTR | 0.02 | down |
| chr19 | 58126367 | 58127147 | 781 | ENSG00000121413 | protein_coding | ZSCAN18 | 3' UTR | 0.02 | down |
| chr10 | 45624634 | 45640293 | 15660 | ENSG00000172671 | processed_transcript | ZFAND4 | 3' UTR | 0.02 | down |
| chr1 | 110222855 | 110224444 | 1590 | ENSG00000116396 | protein_coding | KCNC4 | Exon | 0.02 | down |
| chr17 | 47946830 | 47947954 | 1125 | ENSG00000264019 | lncRNA | AC018521 | Exon | 0.02 | up |
| chr7 | 123749307 | 123750057 | 751 | ENSG00000272686 | lncRNA | WASL-DT | Exon | 0.02 | up |
| chr15 | 43186138 | 43188567 | 2430 | ENSG00000166946 | retained_intron | CCNDBP1 | Exon | 0.02 | up |
| chr11 | 62673176 | 62673476 | 301 | ENSG00000204922 | protein_coding | UQCC3 | 3' UTR | 0.02 | up |
| chr3 | 9841411 | 9841920 | 510 | ENSG00000156990 | retained_intron | RPUSD3 | 3' UTR | 0.02 | up |
| chr14 | 24433006 | 24435611 | 2606 | ENSG00000100441 | retained_intron | KHNYN | 5' UTR | 0.02 | up |
| X | 154396937 | 154397325 | 389 | ENSG00000280195 | lncRNA | AC245140 | Exon | 0.02 | up |
| chr9 | 19230733 | 19276418 | 45686 | ENSG00000137145 | retained_intron | DENND4C | 5' UTR | 0.02 | up |
| chr22 | 35740530 | 35740771 | 242 | ENSG00000100320 | retained_intron | RBFOX2 | 3' UTR | 0.02 | up |
| chr7 | 35695693 | 35695904 | 212 | ENSG00000271122 | lncRNA | AC018647 | Exon | 0.02 | up |
| chr3 | 15255071 | 15255642 | 572 | ENSG00000131370 | protein_coding | SH3BP5 | 3' UTR | 0.02 | up |
| chr2 | 230385238 | 230388702 | 3465 | ENSG00000185404 | retained_intron | SP140L | Exon | 0.01 | down |
| chr10 | 62196613 | 62196854 | 242 | ENSG00000182010 | protein_coding | RTKN2 | 3' UTR | 0.01 | down |
| chr14 | 74066749 | 74068893 | 2145 | ENSG00000119711 | protein_coding | ALDH6A1 | 5' UTR | 0.01 | down |
| chr6 | 29726727 | 29727110 | 384 | ENSG00000239257 | transcribed_processed_pseudogene | RPL23AP1 | Exon | 0.01 | down |
| chr14 | 70370205 | 70370475 | 271 | ENSG00000133983 | processed_transcript | COX16 | 3' UTR | 0.01 | down |
| chr16 | 11869564 | 11870283 | 720 | ENSG00000261560 | lncRNA | AC007216 | 3' UTR | 0.01 | down |
| chr11 | 117206701 | 117206912 | 212 | ENSG00000280143 | TEC | AP000892 | 3' UTR | 0.01 | down |
| chr2 | 108753873 | 108755227 | 1355 | ENSG00000153201 | retained_intron | RANBP2 | Exon | 0.01 | down |
| chr1 | 1402533 | 1405219 | 2687 | ENSG00000264293 | misc_RNA | RN7SL657P | 3' UTR | 0.01 | down |
| chr17 | 67342479 | 67345701 | 3223 | ENSG00000197170 | retained_intron | PSMD12 | 3' UTR | 0.01 | down |
| chr16 | 68023260 | 68023470 | 211 | ENSG00000167264 | retained_intron | DUS2 | 5' UTR | 0.01 | down |
| chr16 | 1808942 | 1809362 | 421 | ENSG00000063854 | processed_transcript | HAGH | 3' UTR | 0.01 | down |
| chr19 | 48481924 | 48482314 | 391 | ENSG00000105443 | processed_transcript | CYTH2 | 3' UTR | 0.01 | down |
| chr1 | 43451934 | 43452054 | 121 | ENSG00000178922 | retained_intron | HYI | Exon | 0.01 | down |
| chr1 | 16157066 | 16157300 | 235 | ENSG00000227959 | lncRNA | AL451042 | Exon | 0.01 | down |
| chr12 | 26922699 | 26928821 | 6123 | ENSG00000064102 | protein_coding | INTS13 | Exon | 0.01 | down |
| chr20 | 5773442 | 5863946 | 90505 | ENSG00000171984 | protein_coding | SHLD1 | 3' UTR | 0.01 | down |
| X | 154402223 | 154402523 | 301 | ENSG00000013563 | retained_intron | DNASE1L1 | 3' UTR | 0.01 | up |
| chr11 | 94869425 | 94871210 | 1786 | ENSG00000166025 | retained_intron | AMOTL1 | 3' UTR | 0.01 | up |
| chr1 | 220879460 | 220879850 | 391 | ENSG00000136630 | protein_coding | HLX | 5' UTR | 0.01 | up |
| chr20 | 21326517 | 21330630 | 4114 | ENSG00000088930 | protein_coding | XRN2 | Exon | 0.01 | up |
| chr1 | 155234145 | 155234325 | 181 | ENSG00000236675 | unprocessed_pseudogene | MTX1P1 | Exon | 0.01 | down |
| chr12 | 55832997 | 55833268 | 272 | ENSG00000182796 | processed_transcript | TMEM198B | Exon | 0.01 | down |
| chr13 | 40982002 | 41019293 | 37292 | ENSG00000120690 | nonsense_mediated_decay | ELF1 | 5' UTR | 0.01 | down |
| chr2 | 27023356 | 27023567 | 212 | ENSG00000084764 | protein_coding | MAPRE3 | Exon | 0.01 | down |
| chr7 | 72948149 | 72948330 | 182 | ENSG00000106133 | processed_transcript | NSUN5P2 | Exon | 0.01 | down |
| chr1 | 15943726 | 15944145 | 420 | ENSG00000116809 | processed_transcript | ZBTB17 | Exon | 0.01 | down |
| chr16 | 2289470 | 2292170 | 2701 | ENSG00000167972 | retained_intron | ABCA3 | Exon | 0.01 | down |
| chr17 | 28759213 | 28762042 | 2830 | ENSG00000173065 | protein_coding | FAM222B | 3' UTR | 0.01 | down |
| chr7 | 27110208 | 27110747 | 540 | ENSG00000105997 | protein_coding | HOXA3 | 5' UTR | 0.01 | down |
| chr17 | 75812240 | 75812331 | 92 | ENSG00000132478 | nonsense_mediated_decay | UNK | 3' UTR | 0.01 | up |
| chr10 | 50343124 | 50418158 | 75035 | ENSG00000198964 | protein_coding | SGMS1 | 3' UTR | 0.01 | up |
| chr15 | 99384751 | 99386419 | 1669 | ENSG00000168904 | processed_transcript | LRRC28 | 3' UTR | 0.01 | up |
| chr17 | 7903466 | 7904110 | 645 | ENSG00000170004 | nonsense_mediated_decay | CHD3 | Exon | 0.01 | up |
| chr15 | 68176493 | 68181451 | 4959 | ENSG00000033800 | processed_transcript | PIAS1 | Exon | 0.01 | up |
| chr2 | 74361153 | 74361214 | 62 | ENSG00000204843 | retained_intron | DCTN1 | 3' UTR | 0.01 | up |
| chr21 | 44077732 | 44079841 | 2110 | ENSG00000160218 | retained_intron | TRAPPC10 | 3' UTR | 0.01 | up |
| chr3 | 172295507 | 172307079 | 11573 | ENSG00000075420 | retained_intron | FNDC3B | Exon | 0.01 | up |
| chr17 | 40474899 | 40475289 | 391 | ENSG00000279806 | TEC | AC018629 | Exon | 0.01 | up |
| chr19 | 58009373 | 58009793 | 421 | ENSG00000176593 | lncRNA | AC008969 | Exon | 0.01 | up |
| chr10 | 73253908 | 73254349 | 442 | ENSG00000227540 | lncRNA | DNAJC9-AS1 | Exon | 0.01 | up |
| chr1 | 244844714 | 244845014 | 301 | ENSG00000153187 | retained_intron | HNRNPU | 3' UTR | 0.01 | up |
| chr10 | 49982189 | 49988186 | 5998 | ENSG00000204149 | processed_transcript | AGAP6 | 3' UTR | 0.01 | up |
| chr7 | 66423304 | 66423515 | 212 | ENSG00000231234 | transcribed_processed_pseudogene | SKP1P1 | Exon | 0.01 | up |
| chr8 | 30547476 | 30549514 | 2039 | ENSG00000157110 | processed_transcript | RBPMS | 3' UTR | 0.01 | up |
| chr6 | 42048540 | 42048690 | 151 | ENSG00000112576 | processed_transcript | CCND3 | 5' UTR | 0.01 | up |
| chr11 | 33054639 | 33056712 | 2074 | ENSG00000176148 | protein_coding | TCP11L1 | Exon | 0.01 | up |
| chr15 | 45484636 | 45486618 | 1983 | ENSG00000104154 | processed_transcript | SLC30A4 | 3' UTR | 0.01 | up |
| chr2 | 113454745 | 113457154 | 2410 | ENSG00000136682 | retained_intron | CBWD2 | 3' UTR | 0.01 | down |
| chr12 | 112168684 | 112169561 | 878 | ENSG00000173064 | retained_intron | HECTD4 | Exon | 0.01 | down |
| chr14 | 99465705 | 99465796 | 92 | ENSG00000183576 | processed_transcript | SETD3 | Exon | 0.01 | down |
| chr12 | 119685880 | 119686271 | 392 | ENSG00000122966 | retained_intron | CIT | 3' UTR | 0.01 | down |
| chr16 | 75566374 | 75566631 | 258 | ENSG00000034713 | protein_coding | GABARAPL2 | 5' UTR | 0.01 | down |
| chr19 | 12669572 | 12669901 | 330 | ENSG00000123154 | nonsense_mediated_decay | WDR83 | 3' UTR | 0.01 | down |
| chr16 | 69647093 | 69647454 | 362 | ENSG00000102908 | nonsense_mediated_decay | NFAT5 | 3' UTR | 0.01 | up |
| chr1 | 19327338 | 19327945 | 608 | ENSG00000040487 | protein_coding | SLC66A1 | 3' UTR | 0.01 | up |
| chr7 | 93886140 | 93886826 | 687 | ENSG00000105825 | protein_coding | TFPI2 | 3' UTR | 0.01 | up |
| chr7 | 141662388 | 141665031 | 2644 | ENSG00000270157 | lncRNA | AC004918 | 3' UTR | 0.01 | up |
| chr5 | 109781548 | 109794178 | 12631 | ENSG00000112893 | processed_transcript | MAN2A1 | Exon | 0.01 | up |
| chr19 | 1505021 | 1505202 | 182 | ENSG00000185761 | processed_transcript | ADAMTSL5 | 3' UTR | 0.01 | up |
| chr20 | 62158784 | 62158935 | 152 | ENSG00000184402 | protein_coding | SS18L1 | 5' UTR | 0.01 | up |
| chr19 | 41771852 | 41772092 | 241 | ENSG00000086548 | protein_coding | CEACAM6 | 3' UTR | 0.01 | up |
| chr2 | 42331619 | 42331829 | 211 | ENSG00000143924 | processed_transcript | EML4 | 3' UTR | 0.01 | up |
| chr11 | 33103150 | 33108413 | 5264 | ENSG00000176102 | nonsense_mediated_decay | CSTF3 | Exon | 0.01 | up |
| chr13 | 51766255 | 51766765 | 511 | ENSG00000102796 | retained_intron | DHRS12 | 3' UTR | 0.01 | up |
| chr19 | 27803595 | 27804002 | 408 | ENSG00000267264 | processed_pseudogene | AC006504 | Exon | 0.01 | down |
| chr9 | 79571952 | 79572553 | 602 | ENSG00000106829 | protein_coding | TLE4 | 5' UTR | 0.01 | down |
| chr15 | 79413112 | 79413832 | 721 | ENSG00000169330 | protein_coding | MINAR1 | 3' UTR | 0.01 | down |
| chr1 | 110210852 | 110211453 | 602 | ENSG00000116396 | retained_intron | KCNC4 | 5' UTR | 0.01 | down |
| chr7 | 139829176 | 139829356 | 181 | ENSG00000059377 | nonsense_mediated_decay | TBXAS1 | 5' UTR | 0.01 | down |
| chr5 | 72905550 | 72909398 | 3849 | ENSG00000083312 | retained_intron | TNPO1 | 3' UTR | 0.01 | down |
| chr11 | 108678344 | 108689014 | 10671 | ENSG00000178105 | protein_coding | DDX10 | Exon | 0.01 | down |
| chr8 | 140605856 | 140626573 | 20718 | ENSG00000123908 | protein_coding | AGO2 | 5' UTR | 0.01 | down |
| chr19 | 1068381 | 1068651 | 271 | ENSG00000180448 | protein_coding | ARHGAP45 | Exon | 0.01 | down |
| chr15 | 49134321 | 49137376 | 3056 | ENSG00000166200 | retained_intron | COPS2 | Exon | 0.01 | up |
| chr12 | 57128572 | 57129022 | 451 | ENSG00000123384 | protein_coding | LRP1 | 5' UTR | 0.01 | up |
| chr2 | 73266577 | 73269007 | 2431 | ENSG00000163013 | retained_intron | FBXO41 | Exon | 0.01 | up |
| chr15 | 77478366 | 77479484 | 1119 | ENSG00000140382 | protein_coding | HMG20A | 3' UTR | 0.01 | up |
| chr6 | 33448906 | 33451514 | 2609 | ENSG00000197283 | processed_transcript | SYNGAP1 | 3' UTR | 0.01 | up |
| chr1 | 239386564 | 239386955 | 392 | ENSG00000133019 | processed_transcript | CHRM3 | 5' UTR | 0.01 | up |
| chr1 | 75750511 | 75750885 | 375 | ENSG00000117054 | processed_transcript | ACADM | 3' UTR | 0.01 | up |
| chr3 | 158096998 | 158097446 | 449 | ENSG00000168779 | protein_coding | SHOX2 | 3' UTR | 0.01 | up |
| chr6 | 167970195 | 167970615 | 421 | ENSG00000130396 | processed_transcript | AFDN | 3' UTR | 0.01 | up |
| chr18 | 75286146 | 75286386 | 241 | ENSG00000179981 | retained_intron | TSHZ1 | Exon | 0.01 | down |
| chr5 | 5464480 | 5465169 | 690 | ENSG00000164151 | retained_intron | ICE1 | Exon | 0.01 | down |
| chr16 | 64944381 | 64944742 | 362 | ENSG00000140937 | processed_transcript | CDH11 | 3' UTR | 0.01 | down |
| chr17 | 28604936 | 28605206 | 271 | ENSG00000167524 | retained_intron | RSKR | 3' UTR | 0.01 | down |
| chr19 | 46999322 | 46999563 | 242 | ENSG00000160007 | retained_intron | ARHGAP35 | Exon | 0.01 | down |
| chr4 | 932222 | 932343 | 122 | ENSG00000178950 | protein_coding | GAK | 5' UTR | 0.01 | down |
| chr2 | 151802883 | 151806947 | 4065 | ENSG00000162980 | processed_transcript | ARL5A | 3' UTR | 0.01 | down |
| chr8 | 143293092 | 143295788 | 2697 | ENSG00000185730 | protein_coding | ZNF696 | 3' UTR | 0.01 | down |
| chr7 | 102454489 | 102454879 | 391 | ENSG00000161036 | protein_coding | LRWD1 | 3' UTR | 0.01 | down |
| chr7 | 90384974 | 90405783 | 20810 | ENSG00000157224 | protein_coding | CLDN12 | 3' UTR | 0.01 | down |
| chr8 | 30566302 | 30570789 | 4488 | ENSG00000157110 | processed_transcript | RBPMS | 3' UTR | 0.01 | down |
| chr17 | 29566380 | 29567037 | 658 | ENSG00000168792 | protein_coding | ABHD15 | 5' UTR | 0.01 | down |
| chr12 | 122207662 | 122207843 | 182 | ENSG00000184047 | retained_intron | DIABLO | 3' UTR | 0.01 | down |
| chr16 | 1779492 | 1780421 | 930 | ENSG00000095906 | retained_intron | NUBP2 | 3' UTR | 0.01 | down |
| chr20 | 44751145 | 44751924 | 780 | ENSG00000276223 | lncRNA | AL118522 | 3' UTR | 0.01 | down |
| chr15 | 70052728 | 70053118 | 391 | ENSG00000140332 | retained_intron | TLE3 | Exon | 0.01 | up |
| chr1 | 178897737 | 178916893 | 19157 | ENSG00000116191 | retained_intron | RALGPS2 | 3' UTR | 0.01 | up |
| chr10 | 99875906 | 99876207 | 302 | ENSG00000107554 | retained_intron | DNMBP | 3' UTR | 0.01 | up |
| chr1 | 91020703 | 91021183 | 481 | ENSG00000122482 | protein_coding | ZNF644 | Exon | 0.01 | up |
| chr1 | 149959712 | 149961053 | 1342 | ENSG00000264522 | retained_intron | OTUD7B | Exon | 0.01 | up |
| chr1 | 175004645 | 175004916 | 272 | ENSG00000116161 | retained_intron | CACYBP | 5' UTR | 0.01 | up |
| chr6 | 30326352 | 30326622 | 271 | ENSG00000204599 | protein_coding | TRIM39 | Exon | 0.01 | up |
| chr9 | 86346406 | 86353204 | 6799 | ENSG00000083223 | protein_coding | TUT7 | 5' UTR | 0.01 | up |
| chr14 | 64744466 | 64744647 | 182 | ENSG00000126822 | processed_transcript | PLEKHG3 | 3' UTR | 0.01 | down |
| chr17 | 81527566 | 81527776 | 211 | ENSG00000186765 | protein_coding | FSCN2 | Exon | 0.01 | down |
| chr16 | 50034638 | 50035716 | 1079 | ENSG00000205423 | protein_coding | CNEP1R1 | 3' UTR | 0.01 | down |
| chr5 | 119392979 | 119394268 | 1290 | ENSG00000145779 | protein_coding | TNFAIP8 | 3' UTR | 0.01 | down |
| chr5 | 69235406 | 69255689 | 20284 | ENSG00000134058 | processed_transcript | CDK7 | 3' UTR | 0.01 | down |
| chr4 | 7031956 | 7032226 | 271 | ENSG00000173013 | protein_coding | CCDC96 | Exon | 0.01 | down |
| chr3 | 10006403 | 10007482 | 1080 | ENSG00000206567 | lncRNA | AC022007 | Exon | 0.01 | down |
| chr5 | 90501927 | 90513425 | 11499 | ENSG00000164199 | protein_coding | ADGRV1 | 3' UTR | 0.01 | down |
| chr12 | 112938443 | 112938593 | 151 | ENSG00000111331 | protein_coding | OAS3 | 5' UTR | 0.01 | up |
| chr8 | 143541677 | 143542095 | 419 | ENSG00000275558 | misc_RNA | RN7SKP175 | Exon | 0.01 | up |
| chr14 | 102225278 | 102225937 | 660 | ENSG00000080823 | processed_transcript | MOK | 3' UTR | 0.01 | up |
| chr11 | 83212959 | 83213677 | 719 | ENSG00000241020 | processed_pseudogene | AP000873 | 3' UTR | 0.01 | up |
| chr1 | 92323474 | 92323925 | 452 | ENSG00000122484 | retained_intron | RPAP2 | Exon | 0.01 | up |
| chr6 | 31762172 | 31762526 | 355 | ENSG00000255152 | nonsense_mediated_decay | MSH5-SAPCD1 | 3' UTR | 0.01 | up |
| chr11 | 9729426 | 9732654 | 3229 | ENSG00000133789 | nonsense_mediated_decay | SWAP70 | 3' UTR | 0.01 | down |
| chr3 | 172762773 | 172764361 | 1589 | ENSG00000114346 | protein_coding | ECT2 | Exon | 0.01 | down |
| chr16 | 29928275 | 29928575 | 301 | ENSG00000247735 | lncRNA | AC120114 | Exon | 0.01 | down |
| X | 154430078 | 154430288 | 211 | ENSG00000071553 | protein_coding | ATP6AP1 | 3' UTR | 0.01 | down |
| chr2 | 11214877 | 11216201 | 1325 | ENSG00000134318 | retained_intron | ROCK2 | 3' UTR | 0.01 | down |
| chr2 | 95412940 | 95413090 | 151 | ENSG00000115042 | retained_intron | FAHD2A | 3' UTR | 0.01 | down |
| chr11 | 66667923 | 66668374 | 452 | ENSG00000173933 | processed_transcript | RBM4 | 3' UTR | 0.01 | down |
| chr4 | 176328441 | 176328681 | 241 | ENSG00000129128 | processed_transcript | SPCS3 | 3' UTR | 0.01 | down |
| chr9 | 88475824 | 88476153 | 330 | ENSG00000130045 | processed_transcript | NXNL2 | 3' UTR | 0.01 | down |
| chr16 | 71894465 | 71903244 | 8780 | ENSG00000102984 | processed_transcript | ZNF821 | 5' UTR | 0.01 | down |
| chr16 | 3606623 | 3611606 | 4984 | ENSG00000188827 | protein_coding | SLX4 | 5' UTR | 0.01 | down |
| chr1 | 243164304 | 243165893 | 1590 | ENSG00000143702 | protein_coding | CEP170 | Exon | 0.01 | down |
| chr7 | 72948151 | 72948356 | 206 | ENSG00000285886 | lncRNA | AC211476 | 3' UTR | 0.01 | down |
| chr11 | 119168506 | 119169116 | 611 | ENSG00000160703 | processed_transcript | NLRX1 | 5' UTR | 0.01 | down |
| chr3 | 49654493 | 49656292 | 1800 | ENSG00000164061 | retained_intron | BSN | Exon | 0.01 | up |
| chr2 | 74491106 | 74491711 | 606 | ENSG00000115282 | retained_intron | TTC31 | 3' UTR | 0.01 | up |
| chr2 | 74135399 | 74145199 | 9801 | ENSG00000163170 | retained_intron | BOLA3 | 3' UTR | 0.01 | up |
| chr8 | 30571866 | 30572256 | 391 | ENSG00000157110 | processed_transcript | RBPMS | 3' UTR | 0.01 | up |
| chr16 | 29901589 | 29901827 | 239 | ENSG00000174939 | protein_coding | ASPHD1 | 5' UTR | 0.01 | up |
| chr3 | 15255110 | 15255619 | 510 | ENSG00000224660 | lncRNA | SH3BP5-AS1 | Exon | 0.01 | up |
| chr1 | 172393870 | 172397192 | 3323 | ENSG00000135845 | processed_transcript | PIGC | Exon | 0.01 | up |
| chr20 | 3777769 | 3778185 | 417 | ENSG00000101222 | processed_transcript | SPEF1 | 3' UTR | 0.01 | down |
| chr10 | 17157515 | 17159176 | 1662 | ENSG00000107614 | processed_transcript | TRDMT1 | 3' UTR | 0.01 | down |
| chr18 | 31042664 | 31042815 | 152 | ENSG00000134762 | protein_coding | DSC3 | 5' UTR | 0.01 | down |
| chr3 | 124969987 | 124970766 | 780 | ENSG00000173706 | processed_transcript | HEG1 | 3' UTR | 0.01 | down |
| chr19 | 39435126 | 39435543 | 418 | ENSG00000105193 | protein_coding | RPS16 | Exon | 0.01 | down |
| chr3 | 123914641 | 123930894 | 16254 | ENSG00000175455 | retained_intron | CCDC14 | 3' UTR | 0.01 | down |
| chr10 | 103917513 | 103918154 | 642 | ENSG00000107960 | protein_coding | STN1 | 5' UTR | 0.01 | down |
| chr22 | 42518690 | 42519796 | 1107 | ENSG00000189306 | protein_coding | RRP7A | 5' UTR | 0.01 | down |
| chr13 | 98519266 | 98519447 | 182 | ENSG00000102572 | protein_coding | STK24 | Exon | 0.01 | down |
| chr10 | 63622700 | 63623060 | 361 | ENSG00000286373 | lncRNA | AC022387 | 3' UTR | 0.01 | down |
| chr8 | 91018466 | 91020170 | 1705 | ENSG00000155099 | protein_coding | PIP4P2 | 3' UTR | 0.01 | down |
| chr15 | 88459500 | 88465645 | 6146 | ENSG00000259494 | nonsense_mediated_decay | MRPL46 | 3' UTR | 0.01 | down |
| chr8 | 23021422 | 23021663 | 242 | ENSG00000245025 | lncRNA | AC107959 | 3' UTR | 0.01 | down |
| chr1 | 2586579 | 2587100 | 522 | ENSG00000157870 | protein_coding | PRXL2B | 5' UTR | 0.01 | down |
| chr2 | 206152467 | 206159246 | 6780 | ENSG00000023228 | protein_coding | NDUFS1 | 5' UTR | 0.01 | up |
| chr12 | 8096051 | 8096472 | 422 | ENSG00000284697 | lncRNA | AC006511 | 3' UTR | 0.01 | up |
| chr22 | 38992508 | 38992778 | 271 | ENSG00000179750 | nonsense_mediated_decay | APOBEC3B | 3' UTR | 0.01 | up |
| chr17 | 45135674 | 45135825 | 152 | ENSG00000181513 | protein_coding | ACBD4 | 5' UTR | 0.01 | up |
| chr1 | 201955532 | 201957630 | 2099 | ENSG00000134375 | processed_transcript | TIMM17A | Exon | 0.01 | up |
| chr12 | 110136787 | 110162384 | 25598 | ENSG00000122970 | processed_transcript | IFT81 | 3' UTR | 0.01 | up |
| chr12 | 57056482 | 57056693 | 212 | ENSG00000166866 | processed_transcript | MYO1A | 3' UTR | 0.01 | up |
| chr7 | 41687590 | 41687800 | 211 | ENSG00000122641 | processed_transcript | INHBA | 3' UTR | 0.01 | down |
| chr2 | 120984499 | 120986303 | 1805 | ENSG00000074047 | nonsense_mediated_decay | GLI2 | 3' UTR | 0.01 | down |
| chr12 | 57525677 | 57525828 | 152 | ENSG00000166987 | nonsense_mediated_decay | MBD6 | Exon | 0.01 | down |
| chr19 | 56222218 | 56223822 | 1605 | ENSG00000131848 | retained_intron | ZSCAN5A | 5' UTR | 0.01 | down |
| chr16 | 58680060 | 58681507 | 1448 | ENSG00000103042 | nonsense_mediated_decay | SLC38A7 | 5' UTR | 0.01 | down |
| chr5 | 6756324 | 6756864 | 541 | ENSG00000112941 | retained_intron | TENT4A | 3' UTR | 0.01 | down |
| chr2 | 159229603 | 159231491 | 1889 | ENSG00000115183 | retained_intron | TANC1 | 3' UTR | 0.01 | down |
| chr19 | 49665141 | 49665890 | 750 | ENSG00000126453 | protein_coding | BCL2L12 | 5' UTR | 0.01 | down |
| chr10 | 73168238 | 73192622 | 24385 | ENSG00000122882 | processed_transcript | ECD | 5' UTR | 0.01 | up |
| chr2 | 218399427 | 218399887 | 461 | ENSG00000144579 | protein_coding | CTDSP1 | 5' UTR | 0.01 | up |
| chr20 | 44498415 | 44499195 | 781 | ENSG00000132824 | protein_coding | SERINC3 | 3' UTR | 0.01 | up |
| chr1 | 145573510 | 145573840 | 331 | ENSG00000272150 | transcribed_unprocessed_pseudogene | NBPF25P | Exon | 0.01 | up |
| chr1 | 109928866 | 109930064 | 1199 | ENSG00000184371 | processed_transcript | CSF1 | 3' UTR | 0.01 | up |
| chr1 | 110947817 | 110950047 | 2231 | ENSG00000121931 | protein_coding | LRIF1 | 3' UTR | 0.01 | up |
| chr1 | 183938270 | 183938988 | 719 | ENSG00000198756 | processed_transcript | COLGALT2 | 3' UTR | 0.01 | up |
| chr1 | 206470475 | 206470536 | 62 | ENSG00000263528 | retained_intron | IKBKE | 5' UTR | 0.01 | up |
| chr17 | 65529547 | 65529996 | 450 | ENSG00000168646 | retained_intron | AXIN2 | 3' UTR | 0.01 | up |
| chr19 | 57444438 | 57445217 | 780 | ENSG00000276449 | lncRNA | AC004076 | Exon | 0.01 | down |
| chr1 | 161519012 | 161519373 | 362 | ENSG00000143226 | processed_transcript | FCGR2A | 3' UTR | 0.01 | down |
| chr17 | 75703286 | 75703497 | 212 | ENSG00000161526 | retained_intron | SAP30BP | 3' UTR | 0.01 | down |
| chr17 | 47056015 | 47056645 | 631 | ENSG00000262879 | lncRNA | AC005670 | Exon | 0.01 | down |
| chr7 | 44058093 | 44058482 | 390 | ENSG00000136279 | protein_coding | DBNL | 3' UTR | 0.01 | up |
| chr12 | 6492121 | 6492929 | 809 | ENSG00000111639 | protein_coding | MRPL51 | 3' UTR | 0.01 | up |
| chr19 | 14155699 | 14169539 | 13841 | ENSG00000072071 | protein_coding | ADGRL1 | Exon | 0.01 | up |
| chr14 | 52719009 | 52721628 | 2620 | ENSG00000100519 | processed_transcript | PSMC6 | Exon | 0.01 | up |
| chr21 | 33520994 | 33524102 | 3109 | ENSG00000159131 | processed_transcript | GART | 3' UTR | 0.01 | up |
| chr21 | 33534723 | 33535285 | 563 | ENSG00000159131 | processed_transcript | GART | Exon | 0.01 | up |
| chr3 | 96349759 | 96349996 | 238 | ENSG00000243547 | processed_pseudogene | HNRNPKP4 | Exon | 0.01 | up |
| chr7 | 2590013 | 2594958 | 4946 | ENSG00000106012 | retained_intron | IQCE | 3' UTR | 0.01 | up |
| chr6 | 107958699 | 107959747 | 1049 | ENSG00000272476 | lncRNA | AL024507 | Exon | 0.01 | up |
| chr3 | 112536492 | 112536673 | 182 | ENSG00000144848 | retained_intron | ATG3 | Exon | 0.01 | up |
| chr12 | 48679791 | 48679911 | 121 | ENSG00000139620 | nonsense_mediated_decay | KANSL2 | Exon | 0.01 | up |
| chr7 | 98317280 | 98355100 | 37821 | ENSG00000272950 | lncRNA | AC093799 | Exon | 0.01 | up |
| chr10 | 73253878 | 73272930 | 19053 | ENSG00000182180 | protein_coding | MRPS16 | 3' UTR | 0.01 | up |
| chr6 | 154733437 | 154733678 | 242 | ENSG00000213079 | protein_coding | SCAF8 | 5' UTR | 0.01 | up |
| chr6 | 143824031 | 143824361 | 331 | ENSG00000257065 | nonsense_mediated_decay | AL049844 | 3' UTR | 0.01 | down |
| chr5 | 436642 | 437212 | 571 | ENSG00000063438 | retained_intron | AHRR | 3' UTR | 0.01 | down |
| chr5 | 436641 | 437212 | 572 | ENSG00000063438 | retained_intron | AHRR | 3' UTR | 0.01 | down |
| chr19 | 12764863 | 12765154 | 292 | ENSG00000095066 | retained_intron | HOOK2 | 3' UTR | 0.01 | down |
| chr15 | 50490380 | 50493276 | 2897 | ENSG00000138592 | retained_intron | USP8 | Exon | 0.01 | down |
| chr3 | 37251421 | 37287536 | 36116 | ENSG00000144674 | protein_coding | GOLGA4 | 5' UTR | 0.01 | down |
| chr15 | 75932695 | 75933145 | 451 | ENSG00000167196 | protein_coding | FBXO22 | 3' UTR | 0.01 | down |
| chr11 | 118633282 | 118633553 | 272 | ENSG00000019144 | retained_intron | PHLDB1 | Exon | 0.01 | down |
| chr5 | 108842975 | 108924683 | 81709 | ENSG00000151422 | protein_coding | FER | 3' UTR | 0.01 | down |
| chr2 | 218659122 | 218659875 | 754 | ENSG00000074582 | protein_coding | BCS1L | 5' UTR | 0.01 | down |
| chr3 | 52292290 | 52293160 | 871 | ENSG00000168237 | retained_intron | GLYCTK | 3' UTR | 0.01 | down |
| chr15 | 57707550 | 57712072 | 4523 | ENSG00000255529 | processed_transcript | POLR2M | 3' UTR | 0.01 | down |
| chr1 | 151342257 | 151342768 | 512 | ENSG00000143390 | retained_intron | RFX5 | Exon | 0.01 | down |
| chr5 | 154957112 | 154960024 | 2913 | ENSG00000082515 | processed_transcript | MRPL22 | 3' UTR | 0.01 | up |
| chr1 | 90916644 | 90918275 | 1632 | ENSG00000122482 | processed_transcript | ZNF644 | 3' UTR | 0.01 | up |
| chr7 | 5420221 | 5423122 | 2902 | ENSG00000188365 | lncRNA | AC092171 | Exon | 0.01 | up |
| chr1 | 87098426 | 87105102 | 6677 | ENSG00000267272 | lncRNA | LINC01140 | 3' UTR | 0.01 | up |
| chr18 | 12427083 | 12431377 | 4295 | ENSG00000267108 | lncRNA | AP001029 | 3' UTR | 0.01 | up |
| chr14 | 20699437 | 20699828 | 392 | ENSG00000258818 | protein_coding | RNASE4 | 3' UTR | 0.01 | up |
| chr2 | 161308454 | 161308635 | 182 | ENSG00000115233 | retained_intron | PSMD14 | 5' UTR | 0.01 | up |
| chr5 | 123023427 | 123023696 | 270 | ENSG00000263432 | misc_RNA | RN7SL689P | 3' UTR | 0.01 | up |
| chr15 | 39949206 | 39955717 | 6512 | ENSG00000128829 | protein_coding | EIF2AK4 | Exon | 0.01 | up |
| chr1 | 156290118 | 156290598 | 481 | ENSG00000198715 | processed_transcript | GLMP | 3' UTR | 0.01 | up |
| chr2 | 27471968 | 27472268 | 301 | ENSG00000138002 | retained_intron | IFT172 | 3' UTR | 0.01 | up |
| chr18 | 63390058 | 63390209 | 152 | ENSG00000119541 | processed_transcript | VPS4B | 3' UTR | 0.01 | down |
| chr1 | 6522034 | 6522185 | 152 | ENSG00000171680 | protein_coding | PLEKHG5 | 3' UTR | 0.01 | down |
| chr7 | 76980746 | 76990319 | 9574 | ENSG00000265479 | lncRNA | DTX2P1-UPK3BP1-PMS2P11 | Exon | 0.01 | down |
| chr7 | 76980734 | 77002234 | 21501 | ENSG00000265479 | lncRNA | DTX2P1-UPK3BP1-PMS2P11 | Exon | 0.01 | down |
| chr16 | 2975677 | 2976057 | 381 | ENSG00000127564 | retained_intron | PKMYT1 | Exon | 0.01 | down |
| chr18 | 3447649 | 3449765 | 2117 | ENSG00000177426 | protein_coding | TGIF1 | 5' UTR | 0.01 | down |
| chr5 | 61159519 | 61162260 | 2742 | ENSG00000251279 | lncRNA | SMIM15-AS1 | 3' UTR | 0.01 | down |
| chr16 | 67163616 | 67164242 | 627 | ENSG00000135722 | retained_intron | FBXL8 | 3' UTR | 0.01 | up |
| chr15 | 89648515 | 89649015 | 501 | ENSG00000166813 | nonsense_mediated_decay | KIF7 | 3' UTR | 0.01 | up |
| chr11 | 119092774 | 119092894 | 121 | ENSG00000256269 | retained_intron | HMBS | 3' UTR | 0.01 | up |
| chr18 | 12978805 | 12982761 | 3957 | ENSG00000085415 | retained_intron | SEH1L | Exon | 0.01 | up |
| chr9 | 122971943 | 122986329 | 14387 | ENSG00000011454 | retained_intron | RABGAP1 | 3' UTR | 0.01 | up |
| chr3 | 32526362 | 32526843 | 482 | ENSG00000144635 | retained_intron | DYNC1LI1 | 3' UTR | 0.01 | up |
| chr1 | 147651364 | 147651725 | 362 | ENSG00000162836 | retained_intron | ACP6 | Exon | 0.01 | up |
| chr5 | 146482593 | 146492975 | 10383 | ENSG00000113649 | retained_intron | TCERG1 | Exon | 0.01 | up |
| chr19 | 1505040 | 1505853 | 814 | ENSG00000185761 | processed_transcript | ADAMTSL5 | 3' UTR | 0.01 | up |
| chr2 | 127052713 | 127053044 | 332 | ENSG00000136717 | retained_intron | BIN1 | Exon | 0.01 | up |
| chr6 | 32979589 | 32979740 | 152 | ENSG00000204256 | retained_intron | BRD2 | Exon | 0.01 | up |
| chr6 | 169663716 | 169664214 | 499 | ENSG00000184465 | processed_transcript | WDR27 | 3' UTR | 0.01 | up |
| chr11 | 66667473 | 66667714 | 242 | ENSG00000173933 | processed_transcript | RBM4 | 3' UTR | 0.01 | up |
| chr12 | 130790373 | 130790523 | 151 | ENSG00000111450 | retained_intron | STX2 | 3' UTR | 0.01 | down |
| chr22 | 19233241 | 19234530 | 1290 | ENSG00000070371 | retained_intron | CLTCL1 | Exon | 0.01 | down |
| chr1 | 15933360 | 15934081 | 722 | ENSG00000065526 | processed_transcript | SPEN | Exon | 0.01 | down |
| chr3 | 142780857 | 142784980 | 4124 | ENSG00000144935 | retained_intron | TRPC1 | Exon | 0.01 | down |
| chr17 | 44323993 | 44324174 | 182 | ENSG00000013306 | retained_intron | SLC25A39 | 5' UTR | 0.01 | down |
| chr1 | 47299885 | 47301682 | 1798 | ENSG00000123473 | retained_intron | STIL | Exon | 0.01 | down |
| chr5 | 154902604 | 154907724 | 5121 | ENSG00000082516 | retained_intron | GEMIN5 | Exon | 0.01 | down |
| chr8 | 38969503 | 38970044 | 542 | ENSG00000169499 | processed_transcript | PLEKHA2 | 3' UTR | 0.01 | up |
| chr6 | 142752845 | 142753803 | 959 | ENSG00000010818 | processed_transcript | HIVEP2 | 3' UTR | 0.01 | up |
| chr7 | 101238670 | 101238820 | 151 | ENSG00000106404 | protein_coding | CLDN15 | 5' UTR | 0.01 | up |
| chr5 | 180849139 | 180849589 | 451 | ENSG00000196670 | retained_intron | ZFP62 | Exon | 0.01 | up |
| chr8 | 12183048 | 12183407 | 360 | ENSG00000186523 | retained_intron | FAM86B1 | 3' UTR | 0.01 | up |
| X | 57120027 | 57120382 | 356 | ENSG00000186787 | protein_coding | SPIN2B | Exon | 0.01 | up |
| chr6 | 30002269 | 30002660 | 392 | ENSG00000237669 | unprocessed_pseudogene | AL671277 | Exon | 0.01 | down |
| chr14 | 74060082 | 74064907 | 4826 | ENSG00000119711 | protein_coding | ALDH6A1 | 3' UTR | 0.01 | down |
| chr1 | 31297011 | 31346793 | 49783 | ENSG00000121766 | protein_coding | ZCCHC17 | 5' UTR | 0.01 | up |
| X | 37839252 | 37839733 | 482 | ENSG00000165169 | protein_coding | DYNLT3 | 3' UTR | 0.01 | up |
| chr3 | 25764076 | 25777795 | 13720 | ENSG00000151092 | protein_coding | NGLY1 | 3' UTR | 0.01 | up |
| chr8 | 23437008 | 23439947 | 2940 | ENSG00000197217 | protein_coding | ENTPD4 | Exon | 0.01 | up |
| chr11 | 44619161 | 44620149 | 989 | ENSG00000085117 | protein_coding | CD82 | 3' UTR | 0.01 | up |
| chr5 | 40827702 | 40832555 | 4854 | ENSG00000145592 | processed_transcript | RPL37 | 3' UTR | 0.01 | up |
| chr5 | 141418899 | 141419289 | 391 | ENSG00000254122 | protein_coding | PCDHGB7 | Exon | 0.01 | down |
| chr9 | 96012357 | 96013166 | 810 | ENSG00000182150 | protein_coding | ERCC6L2 | 3' UTR | 0.01 | down |
| chr11 | 68016019 | 68016290 | 272 | ENSG00000006534 | protein_coding | ALDH3B1 | Exon | 0.01 | down |
| chr2 | 177618053 | 177618293 | 241 | ENSG00000197557 | protein_coding | TTC30A | Exon | 0.01 | down |
| chr7 | 86912134 | 86912950 | 817 | ENSG00000164659 | nonsense_mediated_decay | ELAPOR2 | 3' UTR | 0.01 | down |
| chr5 | 146130844 | 146131025 | 182 | ENSG00000133706 | retained_intron | LARS1 | 3' UTR | 0.01 | down |
| chr9 | 124814405 | 124814585 | 181 | ENSG00000185585 | protein_coding | OLFML2A | 3' UTR | 0.01 | down |
| chr2 | 231170683 | 231172827 | 2145 | ENSG00000173692 | retained_intron | PSMD1 | 3' UTR | 0.01 | down |
| chr20 | 25675491 | 25676121 | 631 | ENSG00000278383 | lncRNA | AL031673 | Exon | 0.01 | down |
| chr3 | 120193383 | 120285064 | 91682 | ENSG00000175697 | nonsense_mediated_decay | GPR156 | 5' UTR | 0.01 | down |
| chr5 | 172054587 | 172057135 | 2549 | ENSG00000072786 | retained_intron | STK10 | Exon | 0.01 | down |
| chr7 | 2434315 | 2435454 | 1140 | ENSG00000136213 | processed_transcript | CHST12 | 3' UTR | 0.01 | down |
| chr5 | 40762644 | 40764403 | 1760 | ENSG00000132356 | retained_intron | PRKAA1 | 3' UTR | 0.01 | down |
| chr19 | 16520663 | 16521292 | 630 | ENSG00000085872 | retained_intron | CHERP | 3' UTR | 0.01 | up |
| chr12 | 45851204 | 45852735 | 1532 | ENSG00000189079 | protein_coding | ARID2 | 5' UTR | 0.01 | up |
| chr17 | 35352158 | 35353432 | 1275 | ENSG00000172716 | protein_coding | SLFN11 | 3' UTR | 0.01 | up |
| chr15 | 42283690 | 42284110 | 421 | ENSG00000214013 | retained_intron | GANC | 3' UTR | 0.01 | up |
| chr15 | 57706874 | 57707085 | 212 | ENSG00000255529 | processed_transcript | POLR2M | 3' UTR | 0.01 | up |
| chr4 | 16227159 | 16256405 | 29247 | ENSG00000169762 | processed_transcript | TAPT1 | Exon | 0.01 | down |
| chr5 | 179840310 | 179840460 | 151 | ENSG00000161010 | protein_coding | MRNIP | 3' UTR | 0.01 | down |
| chr17 | 36504599 | 36504660 | 62 | ENSG00000278259 | retained_intron | MYO19 | 3' UTR | 0.01 | down |
| chr20 | 25448031 | 25448152 | 122 | ENSG00000101003 | processed_transcript | GINS1 | 3' UTR | 0.01 | down |
| chr8 | 43103649 | 43103980 | 332 | ENSG00000185900 | protein_coding | POMK | Exon | 0.01 | down |
| chr1 | 173782145 | 173785825 | 3681 | ENSG00000076321 | processed_transcript | KLHL20 | 3' UTR | 0.01 | down |
| chr19 | 35544366 | 35545506 | 1141 | ENSG00000236144 | lncRNA | TMEM147-AS1 | Exon | 0.01 | down |
| chr16 | 66721491 | 66722361 | 871 | ENSG00000135720 | nonsense_mediated_decay | DYNC1LI2 | 3' UTR | 0.01 | up |
| chr7 | 139626644 | 139628992 | 2349 | ENSG00000064393 | protein_coding | HIPK2 | Exon | 0.01 | up |
| chr6 | 26452815 | 26453415 | 601 | ENSG00000111801 | retained_intron | BTN3A3 | 3' UTR | 0.01 | up |
| chr18 | 31067562 | 31068163 | 602 | ENSG00000134762 | protein_coding | DSC3 | 3' UTR | 0.01 | up |
| chr6 | 26394305 | 26394874 | 570 | ENSG00000124508 | protein_coding | BTN2A2 | 3' UTR | 0.01 | up |
| chr21 | 46556383 | 46556833 | 451 | ENSG00000160305 | retained_intron | DIP2A | Exon | 0.01 | up |
| chr17 | 63816864 | 63817105 | 242 | ENSG00000198231 | processed_transcript | DDX42 | Exon | 0.01 | up |
| chr2 | 174481533 | 174483080 | 1548 | ENSG00000163328 | processed_transcript | GPR155 | 5' UTR | 0.01 | up |
| chr6 | 136260373 | 136260554 | 182 | ENSG00000029363 | nonsense_mediated_decay | BCLAF1 | 3' UTR | 0.01 | up |
| chr7 | 86880175 | 86891290 | 11116 | ENSG00000164659 | retained_intron | ELAPOR2 | 3' UTR | 0.01 | up |
| chr3 | 133600979 | 133608955 | 7977 | ENSG00000163781 | processed_transcript | TOPBP1 | 3' UTR | 0.01 | up |
| chr2 | 135135638 | 135135878 | 241 | ENSG00000208308 | snoRNA | SNORA40B | Exon | 0.01 | down |
| chr4 | 145481889 | 145482607 | 719 | ENSG00000170365 | protein_coding | SMAD1 | 5' UTR | 0.01 | down |
| chr12 | 284465 | 284946 | 482 | ENSG00000261799 | lncRNA | AC007406 | 3' UTR | 0.01 | down |
| chr10 | 46634917 | 46635472 | 556 | ENSG00000165874 | processed_transcript | SHLD2P1 | Exon | 0.01 | down |
| chr17 | 62450245 | 62450515 | 271 | ENSG00000146872 | protein_coding | TLK2 | 3' UTR | 0.01 | down |
| chr3 | 197676379 | 197676799 | 421 | ENSG00000284146 | miRNA | MIR922 | 3' UTR | 0.01 | down |
| chr4 | 38932272 | 38938532 | 6261 | ENSG00000197712 | processed_transcript | FAM114A1 | Exon | 0.01 | down |
| chr22 | 21445050 | 21445470 | 421 | ENSG00000169635 | protein_coding | HIC2 | Exon | 0.01 | down |
| chr17 | 40399942 | 40400898 | 957 | ENSG00000131747 | retained_intron | TOP2A | Exon | 0.01 | down |
| chr19 | 9633423 | 9634351 | 929 | ENSG00000267106 | lncRNA | ZNF561-AS1 | Exon | 0.01 | down |
| chr4 | 1939629 | 1939930 | 302 | ENSG00000109685 | protein_coding | NSD2 | 3' UTR | 0.01 | down |
| chr11 | 118520882 | 118522574 | 1693 | ENSG00000118058 | retained_intron | KMT2A | 3' UTR | 0.01 | down |
| chr21 | 46659230 | 46661262 | 2033 | ENSG00000160310 | retained_intron | PRMT2 | 3' UTR | 0.01 | up |
| chr12 | 12329795 | 12330933 | 1139 | ENSG00000111261 | protein_coding | MANSC1 | 3' UTR | 0.01 | up |
| chr21 | 45072688 | 45073896 | 1209 | ENSG00000197381 | processed_transcript | ADARB1 | Exon | 0.01 | up |
| chr5 | 90461104 | 90472592 | 11489 | ENSG00000113356 | protein_coding | POLR3G | 3' UTR | 0.01 | up |
| chr19 | 43502210 | 43504012 | 1803 | ENSG00000176531 | protein_coding | PHLDB3 | Exon | 0.01 | up |
| chr1 | 117941798 | 117943479 | 1682 | ENSG00000065183 | processed_transcript | WDR3 | Exon | 0.01 | up |
| chr9 | 62837943 | 62838213 | 271 | ENSG00000229422 | lncRNA | AL512625 | Exon | 0.01 | up |
| chr7 | 149102992 | 149103469 | 478 | ENSG00000204947 | retained_intron | ZNF425 | 3' UTR | 0.01 | up |
| chr19 | 52703410 | 52703590 | 181 | ENSG00000213020 | processed_transcript | ZNF611 | 3' UTR | 0.01 | up |
| chr19 | 44228499 | 44228800 | 302 | ENSG00000131115 | processed_transcript | ZNF227 | 5' UTR | 0.01 | down |
| chr17 | 3033227 | 3033348 | 122 | ENSG00000132359 | retained_intron | RAP1GAP2 | Exon | 0.01 | down |
| chr4 | 7031949 | 7032220 | 272 | ENSG00000173011 | protein_coding | TADA2B | 3' UTR | 0.01 | down |
| chr15 | 90275448 | 90275778 | 331 | ENSG00000228998 | transcribed_processed_pseudogene | AC091167 | 3' UTR | 0.01 | down |
| X | 130066497 | 130066708 | 212 | ENSG00000102034 | protein_coding | ELF4 | 3' UTR | 0.01 | down |
| chr2 | 190895580 | 190895881 | 302 | ENSG00000115419 | retained_intron | GLS | Exon | 0.01 | up |
| chr21 | 44813980 | 44818092 | 4113 | ENSG00000184900 | protein_coding | SUMO3 | 5' UTR | 0.01 | up |
| chr22 | 38992507 | 38992804 | 298 | ENSG00000179750 | nonsense_mediated_decay | APOBEC3B | 3' UTR | 0.01 | up |
| chr9 | 127937555 | 127937794 | 240 | ENSG00000136908 | retained_intron | DPM2 | Exon | 0.01 | up |
| chr1 | 203837999 | 203849835 | 11837 | ENSG00000058673 | processed_transcript | ZC3H11A | 3' UTR | 0.01 | up |
| chr15 | 101071817 | 101072836 | 1020 | ENSG00000154237 | processed_transcript | LRRK1 | 3' UTR | 0.01 | up |
| chr11 | 134260100 | 134260311 | 212 | ENSG00000151498 | retained_intron | ACAD8 | Exon | 0.01 | up |
| chr17 | 6070513 | 6078016 | 7504 | ENSG00000179314 | protein_coding | WSCD1 | 5' UTR | 0.01 | up |
| chr1 | 193069539 | 193075869 | 6331 | ENSG00000116747 | protein_coding | RO60 | 5' UTR | 0.01 | down |
| chr1 | 16230945 | 16231335 | 391 | ENSG00000288398 | lncRNA | AL109627 | Exon | 0.01 | down |
| chr7 | 107567121 | 107576533 | 9413 | ENSG00000105865 | retained_intron | DUS4L | 3' UTR | 0.01 | down |
| chr17 | 42609223 | 42609374 | 152 | ENSG00000141699 | protein_coding | RETREG3 | 5' UTR | 0.01 | down |
| chr10 | 122248686 | 122254065 | 5380 | ENSG00000138162 | retained_intron | TACC2 | 3' UTR | 0.01 | down |
| chr16 | 25239207 | 25243903 | 4697 | ENSG00000155592 | retained_intron | ZKSCAN2 | 3' UTR | 0.01 | down |
| chr3 | 96987375 | 96987885 | 511 | ENSG00000080224 | protein_coding | EPHA6 | Exon | 0.01 | down |
| chr17 | 43251828 | 43252019 | 192 | ENSG00000278048 | snRNA | U2 | Exon | 0.01 | down |
| chr8 | 144357331 | 144357392 | 62 | ENSG00000182325 | processed_transcript | FBXL6 | Exon | 0.01 | down |
| chr17 | 20306035 | 20314588 | 8554 | ENSG00000128487 | retained_intron | SPECC1 | 3' UTR | 0.01 | down |
| chr5 | 179867054 | 179867354 | 301 | ENSG00000197226 | retained_intron | TBC1D9B | Exon | 0.01 | down |
| chr2 | 62000874 | 62001024 | 151 | ENSG00000173163 | protein_coding | COMMD1 | 3' UTR | 0.01 | down |
| chr1 | 156938918 | 156939938 | 1021 | ENSG00000132694 | processed_transcript | ARHGEF11 | Exon | 0.01 | up |
| chr22 | 21920532 | 21920592 | 61 | ENSG00000100034 | processed_transcript | PPM1F | 3' UTR | 0.01 | up |
| chr5 | 115913931 | 115914081 | 151 | ENSG00000177879 | processed_transcript | AP3S1 | 3' UTR | 0.01 | up |
| chr12 | 49642501 | 49643293 | 793 | ENSG00000110844 | retained_intron | PRPF40B | 3' UTR | 0.01 | up |
| chr17 | 36504119 | 36504330 | 212 | ENSG00000278259 | retained_intron | MYO19 | 3' UTR | 0.01 | up |
| chr6 | 43520990 | 43529563 | 8574 | ENSG00000124571 | retained_intron | XPO5 | 3' UTR | 0.01 | up |
| chr11 | 108719852 | 108723384 | 3533 | ENSG00000178105 | processed_transcript | DDX10 | Exon | 0.01 | up |
| chr11 | 121060033 | 121087236 | 27204 | ENSG00000154114 | retained_intron | TBCEL | 3' UTR | 0.01 | down |
| chr4 | 102796031 | 102796182 | 152 | ENSG00000109332 | retained_intron | UBE2D3 | 3' UTR | 0.01 | down |
| chr12 | 3303124 | 3318677 | 15554 | ENSG00000236908 | lncRNA | LINC02827 | Exon | 0.01 | down |
| chr2 | 28849969 | 28851290 | 1322 | ENSG00000171103 | protein_coding | TRMT61B | 3' UTR | 0.01 | down |
| chr17 | 36507035 | 36507475 | 441 | ENSG00000278259 | retained_intron | MYO19 | 3' UTR | 0.01 | down |
| chr20 | 44619521 | 44619761 | 241 | ENSG00000196839 | processed_transcript | ADA | 3' UTR | 0.01 | down |
| chr17 | 2394906 | 2396530 | 1625 | ENSG00000070444 | processed_transcript | MNT | Exon | 0.01 | up |
| chr17 | 63817672 | 63818510 | 839 | ENSG00000198231 | retained_intron | DDX42 | 3' UTR | 0.01 | up |
| chr19 | 45782221 | 45782552 | 332 | ENSG00000104936 | protein_coding | DMPK | 5' UTR | 0.01 | up |
| chr16 | 89269954 | 89270165 | 212 | ENSG00000268218 | lncRNA | AC137932 | Exon | 0.01 | up |
| chr2 | 85390221 | 85390372 | 152 | ENSG00000115459 | retained_intron | ELMOD3 | 3' UTR | 0.01 | up |
| chr2 | 134953917 | 134955057 | 1141 | ENSG00000082258 | protein_coding | CCNT2 | 3' UTR | 0.01 | up |
| chr2 | 200535016 | 200542585 | 7570 | ENSG00000163535 | retained_intron | SGO2 | Exon | 0.01 | up |
| chr5 | 51391419 | 51394281 | 2863 | ENSG00000016082 | processed_transcript | ISL1 | 3' UTR | 0.01 | up |
| chr1 | 236208083 | 236208443 | 361 | ENSG00000077585 | processed_transcript | GPR137B | 3' UTR | 0.01 | down |
| chr3 | 169115714 | 169120912 | 5199 | ENSG00000085276 | retained_intron | MECOM | Exon | 0.01 | down |
| chr17 | 5024891 | 5025132 | 242 | ENSG00000129250 | processed_transcript | KIF1C | 3' UTR | 0.01 | down |
| chr9 | 113260513 | 113260754 | 242 | ENSG00000136875 | protein_coding | PRPF4 | 3' UTR | 0.01 | down |
| chr9 | 14087295 | 14094321 | 7027 | ENSG00000147862 | processed_transcript | NFIB | 3' UTR | 0.01 | down |
| chr16 | 674053 | 674174 | 122 | ENSG00000140983 | retained_intron | RHOT2 | 3' UTR | 0.01 | down |
| chr2 | 109544055 | 109545505 | 1451 | ENSG00000186522 | retained_intron | SEPTIN10 | 3' UTR | 0.01 | down |
| chr7 | 98214623 | 98215043 | 421 | ENSG00000205356 | retained_intron | TECPR1 | 3' UTR | 0.01 | down |
| chr2 | 85581851 | 85582031 | 181 | ENSG00000168899 | protein_coding | VAMP5 | 3' UTR | 0.01 | down |
| chr11 | 6618728 | 6619059 | 332 | ENSG00000166340 | protein_coding | TPP1 | 3' UTR | 0.01 | up |
| chr19 | 40396102 | 40396702 | 601 | ENSG00000105227 | nonsense_mediated_decay | PRX | 3' UTR | 0.01 | up |
| chr17 | 7936476 | 7940147 | 3672 | ENSG00000170037 | retained_intron | CNTROB | 3' UTR | 0.01 | up |
| chr14 | 75863743 | 75886753 | 23011 | ENSG00000119685 | processed_transcript | TTLL5 | Exon | 0.01 | up |
| chr16 | 4821660 | 4832543 | 10884 | ENSG00000140632 | retained_intron | GLYR1 | 3' UTR | 0.01 | up |
| chr17 | 81683594 | 81684163 | 570 | ENSG00000185359 | retained_intron | HGS | 5' UTR | 0.01 | up |
| chr8 | 19394930 | 19395949 | 1020 | ENSG00000104611 | retained_intron | SH2D4A | 3' UTR | 0.01 | up |
| chr16 | 57025453 | 57026953 | 1501 | ENSG00000140853 | protein_coding | NLRC5 | Exon | 0.01 | up |
| chr6 | 35087007 | 35087098 | 92 | ENSG00000064999 | processed_transcript | ANKS1A | 3' UTR | 0.01 | up |
| chr5 | 637667 | 639177 | 1511 | ENSG00000112877 | retained_intron | CEP72 | Exon | 0.01 | up |
| chr3 | 138162059 | 138163451 | 1393 | ENSG00000138231 | retained_intron | DBR1 | 3' UTR | 0.01 | up |
| chr5 | 198693 | 199263 | 571 | ENSG00000164366 | protein_coding | CCDC127 | 3' UTR | 0.01 | up |
| chr10 | 119919454 | 119919575 | 122 | ENSG00000107651 | processed_transcript | SEC23IP | Exon | 0.01 | up |
| chr2 | 201700686 | 201701257 | 572 | ENSG00000082126 | protein_coding | MPP4 | 3' UTR | 0.01 | down |
| chr22 | 39022834 | 39022984 | 151 | ENSG00000243811 | protein_coding | APOBEC3D | Exon | 0.01 | down |
| chr9 | 33255249 | 33257038 | 1790 | ENSG00000107262 | processed_transcript | BAG1 | 3' UTR | 0.01 | down |
| chr8 | 47279999 | 47293946 | 13948 | ENSG00000164808 | retained_intron | SPIDR | 3' UTR | 0.01 | up |
| chr12 | 52058848 | 52059059 | 212 | ENSG00000123358 | retained_intron | NR4A1 | 3' UTR | 0.01 | up |
| chr22 | 50255639 | 50255760 | 122 | ENSG00000188130 | retained_intron | MAPK12 | Exon | 0.01 | up |
| chr7 | 156966247 | 156968768 | 2522 | ENSG00000146909 | retained_intron | NOM1 | Exon | 0.01 | up |
| chr11 | 63536807 | 63544786 | 7980 | ENSG00000133321 | protein_coding | PLAAT4 | 5' UTR | 0.01 | up |
| chr7 | 27128998 | 27129384 | 387 | ENSG00000197576 | protein_coding | HOXA4 | 3' UTR | 0.01 | up |
| chr1 | 21728645 | 21729750 | 1106 | ENSG00000090686 | processed_transcript | USP48 | 3' UTR | 0.01 | down |
| X | 37836816 | 37836967 | 152 | ENSG00000165169 | protein_coding | DYNLT3 | 3' UTR | 0.01 | down |
| chr16 | 29457693 | 29461406 | 3714 | ENSG00000213648 | protein_coding | SULT1A4 | 3' UTR | 0.01 | down |
| chr14 | 23532962 | 23533672 | 711 | ENSG00000136367 | processed_transcript | ZFHX2 | Exon | 0.01 | down |
| chr2 | 219236597 | 219237661 | 1065 | ENSG00000163521 | processed_transcript | GLB1L | 3' UTR | 0.01 | down |
| chr13 | 102805342 | 102821069 | 15728 | ENSG00000134897 | retained_intron | BIVM | 3' UTR | 0.01 | up |
| chr5 | 132294393 | 132294812 | 420 | ENSG00000197208 | protein_coding | SLC22A4 | 5' UTR | 0.01 | up |
| chr2 | 43947330 | 43948488 | 1159 | ENSG00000138095 | retained_intron | LRPPRC | Exon | 0.01 | up |
| chr8 | 41974744 | 41978749 | 4006 | ENSG00000083168 | protein_coding | KAT6A | 3' UTR | 0.01 | up |
| chr6 | 26390748 | 26390988 | 241 | ENSG00000124508 | protein_coding | BTN2A2 | 3' UTR | 0.01 | up |
| chr13 | 51766268 | 51766779 | 512 | ENSG00000139668 | processed_transcript | WDFY2 | 3' UTR | 0.01 | up |
| chr2 | 207598529 | 207598799 | 271 | ENSG00000118260 | processed_transcript | CREB1 | 3' UTR | 0.01 | down |
| chr12 | 53305978 | 53306183 | 206 | ENSG00000139637 | retained_intron | MYG1 | Exon | 0.01 | up |
| chr19 | 7684983 | 7685462 | 480 | ENSG00000181029 | protein_coding | TRAPPC5 | 3' UTR | 0.01 | up |
| chr10 | 68338519 | 68338876 | 358 | ENSG00000096746 | processed_transcript | HNRNPH3 | Exon | 0.01 | up |
| chr9 | 98085613 | 98085854 | 242 | ENSG00000106785 | processed_transcript | TRIM14 | 3' UTR | 0.01 | up |
| chr13 | 114246033 | 114247155 | 1123 | ENSG00000130177 | processed_transcript | CDC16 | Exon | 0.01 | up |
| chr7 | 140726128 | 140726488 | 361 | ENSG00000157764 | retained_intron | BRAF | 3' UTR | 0.01 | up |
| chr5 | 173090513 | 173090844 | 332 | ENSG00000164463 | protein_coding | CREBRF | Exon | 0.01 | up |
| chr18 | 48949835 | 48950465 | 631 | ENSG00000101665 | protein_coding | SMAD7 | 5' UTR | 0.01 | down |
| chr5 | 1801406 | 1802183 | 778 | ENSG00000145494 | protein_coding | NDUFS6 | 5' UTR | 0.01 | down |
| chr11 | 117206685 | 117206926 | 242 | ENSG00000280143 | TEC | AP000892 | 3' UTR | 0.01 | down |
| chr2 | 97809751 | 97812533 | 2783 | ENSG00000075568 | retained_intron | TMEM131 | Exon | 0.01 | down |
| chr12 | 53274347 | 53276786 | 2440 | ENSG00000135476 | processed_transcript | ESPL1 | 3' UTR | 0.01 | down |
| chr19 | 49918564 | 49927936 | 9373 | ENSG00000213024 | protein_coding | NUP62 | 5' UTR | 0.01 | up |
| chr5 | 95900964 | 95913377 | 12414 | ENSG00000118985 | retained_intron | ELL2 | Exon | 0.01 | up |
| chr1 | 67411856 | 67412367 | 512 | ENSG00000142864 | processed_transcript | SERBP1 | 3' UTR | 0.01 | up |
| chr16 | 89046859 | 89047279 | 421 | ENSG00000256982 | lncRNA | AC135782 | Exon | 0.01 | up |
| chr7 | 1086858 | 1087037 | 180 | ENSG00000164850 | protein_coding | GPER1 | 5' UTR | 0.01 | up |
| chr5 | 75361382 | 75361952 | 571 | ENSG00000113161 | retained_intron | HMGCR | 3' UTR | 0.01 | down |
| chr2 | 105339666 | 105345482 | 5817 | ENSG00000135974 | protein_coding | C2orf49 | 3' UTR | 0.01 | down |
| X | 40651476 | 40653948 | 2473 | ENSG00000185753 | protein_coding | CXorf38 | 3' UTR | 0.01 | down |
| chr5 | 177210909 | 177238292 | 27384 | ENSG00000165671 | nonsense_mediated_decay | NSD1 | 3' UTR | 0.01 | down |
| chr5 | 128258657 | 128258748 | 92 | ENSG00000138829 | protein_coding | FBN2 | 3' UTR | 0.01 | down |
| chr15 | 63071434 | 63071705 | 272 | ENSG00000140416 | retained_intron | TPM1 | 3' UTR | 0.01 | down |
| chr20 | 43591026 | 43605002 | 13977 | ENSG00000101052 | processed_transcript | IFT52 | 5' UTR | 0.01 | down |
| chr12 | 69587942 | 69588333 | 392 | ENSG00000166226 | retained_intron | CCT2 | Exon | 0.01 | down |
| chr14 | 103711459 | 103711730 | 272 | ENSG00000100711 | protein_coding | ZFYVE21 | 3' UTR | 0.01 | up |
| chr9 | 92245029 | 92250245 | 5217 | ENSG00000196305 | retained_intron | IARS1 | 3' UTR | 0.01 | up |
| chr19 | 45164862 | 45178237 | 13376 | ENSG00000007255 | protein_coding | TRAPPC6A | 5' UTR | 0.01 | up |
| chr2 | 135784576 | 135784996 | 421 | ENSG00000144224 | retained_intron | UBXN4 | 3' UTR | 0.01 | up |
| chr22 | 50768548 | 50768848 | 301 | ENSG00000079974 | processed_transcript | RABL2B | 3' UTR | 0.01 | up |
| chr17 | 55773808 | 55775366 | 1559 | ENSG00000141179 | protein_coding | PCTP | 3' UTR | 0.01 | up |
| chr1 | 225984469 | 225984740 | 272 | ENSG00000143751 | protein_coding | SDE2 | 3' UTR | 0.01 | down |
| chr19 | 51964608 | 51964937 | 330 | ENSG00000256683 | protein_coding | ZNF350 | 3' UTR | 0.01 | down |
| chr22 | 45176001 | 45177830 | 1830 | ENSG00000093000 | retained_intron | NUP50 | 3' UTR | 0.01 | down |
| chr8 | 31087863 | 31090872 | 3010 | ENSG00000165392 | retained_intron | WRN | 3' UTR | 0.01 | down |
| chr16 | 5085972 | 5086242 | 271 | ENSG00000033011 | processed_transcript | ALG1 | 3' UTR | 0.01 | down |
| chr4 | 139137604 | 139177009 | 39406 | ENSG00000109381 | protein_coding | ELF2 | 5' UTR | 0.01 | down |
| chr19 | 17505209 | 17505270 | 62 | ENSG00000130304 | protein_coding | SLC27A1 | 3' UTR | 0.01 | down |
| chr17 | 76479768 | 76481381 | 1614 | ENSG00000129667 | retained_intron | RHBDF2 | Exon | 0.01 | down |
| chr15 | 44409841 | 44413989 | 4149 | ENSG00000166734 | processed_transcript | GOLM2 | 3' UTR | 0.01 | down |
| chr15 | 40022503 | 40035591 | 13089 | ENSG00000128829 | protein_coding | EIF2AK4 | 3' UTR | 0.01 | down |
| chr17 | 82016871 | 82017406 | 536 | ENSG00000169696 | processed_transcript | ASPSCR1 | 3' UTR | 0.01 | up |
| chr2 | 32439513 | 32442334 | 2822 | ENSG00000115760 | retained_intron | BIRC6 | 3' UTR | 0.01 | up |
| chr12 | 71125323 | 71132813 | 7491 | ENSG00000127324 | processed_transcript | TSPAN8 | 3' UTR | 0.01 | up |
| chr21 | 9069469 | 9071637 | 2169 | ENSG00000188681 | transcribed_unprocessed_pseudogene | TEKT4P2 | Exon | 0.01 | up |
| chr9 | 19123427 | 19125614 | 2188 | ENSG00000147872 | processed_transcript | PLIN2 | 3' UTR | 0.01 | up |
| chr3 | 66038174 | 66038714 | 541 | ENSG00000151276 | protein_coding | MAGI1 | 5' UTR | 0.01 | up |
| chr16 | 31141748 | 31142568 | 821 | ENSG00000178226 | retained_intron | PRSS36 | Exon | 0.01 | up |
| chr19 | 18365313 | 18365613 | 301 | ENSG00000130517 | protein_coding | PGPEP1 | 3' UTR | 0.01 | down |
| chr13 | 102766213 | 102767411 | 1199 | ENSG00000151287 | protein_coding | TEX30 | 3' UTR | 0.01 | down |
| chr17 | 7314993 | 7315354 | 362 | ENSG00000132522 | protein_coding | GPS2 | 3' UTR | 0.01 | down |
| chr15 | 44521308 | 44524580 | 3273 | ENSG00000137770 | retained_intron | CTDSPL2 | 3' UTR | 0.01 | down |
| chr5 | 141214846 | 141215835 | 990 | ENSG00000187372 | protein_coding | PCDHB13 | Exon | 0.01 | up |
| chr12 | 57783202 | 57783774 | 573 | ENSG00000123297 | processed_transcript | TSFM | 5' UTR | 0.01 | up |
| chr7 | 157386292 | 157410122 | 23831 | ENSG00000105993 | retained_intron | DNAJB6 | 3' UTR | 0.01 | up |
| chr2 | 86065393 | 86065574 | 182 | ENSG00000068654 | processed_transcript | POLR1A | Exon | 0.01 | up |
| chr5 | 134855655 | 134856016 | 362 | ENSG00000181904 | protein_coding | C5orf24 | 3' UTR | 0.01 | up |
| chr14 | 54479730 | 54480030 | 301 | ENSG00000197045 | retained_intron | GMFB | 3' UTR | 0.01 | up |
| chr14 | 20699481 | 20699840 | 360 | ENSG00000258818 | protein_coding | RNASE4 | 3' UTR | 0.01 | up |
| chr10 | 121911008 | 121924285 | 13278 | ENSG00000107669 | protein_coding | ATE1 | 5' UTR | 0.01 | up |
| chr10 | 49974164 | 49974344 | 181 | ENSG00000178440 | processed_transcript | TIMM23B-AGAP6 | 3' UTR | 0.01 | up |
| chr10 | 5890262 | 5894473 | 4212 | ENSG00000134452 | processed_transcript | FBH1 | 5' UTR | 0.01 | up |
| chr2 | 43794526 | 43794797 | 272 | ENSG00000138036 | retained_intron | DYNC2LI1 | 3' UTR | 0.01 | down |
| chr11 | 77813318 | 77813587 | 270 | ENSG00000219529 | processed_pseudogene | AP000580 | Exon | 0.01 | down |
| chr16 | 15032382 | 15032832 | 451 | ENSG00000179889 | retained_intron | PDXDC1 | Exon | 0.01 | down |
| chr1 | 15932130 | 15933151 | 1022 | ENSG00000065526 | processed_transcript | SPEN | Exon | 0.01 | down |
| chr22 | 26477075 | 26479474 | 2400 | ENSG00000100099 | nonsense_mediated_decay | HPS4 | 5' UTR | 0.01 | down |
| chr16 | 31083454 | 31084501 | 1048 | ENSG00000151006 | retained_intron | PRSS53 | 3' UTR | 0.01 | down |
| chr3 | 12816472 | 12817369 | 898 | ENSG00000144712 | processed_transcript | CAND2 | 3' UTR | 0.01 | up |
| chr16 | 57235199 | 57239749 | 4551 | ENSG00000102931 | processed_transcript | ARL2BP | 3' UTR | 0.01 | up |
| chr12 | 65825358 | 65828637 | 3280 | ENSG00000149948 | nonsense_mediated_decay | HMGA2 | Exon | 0.01 | up |
| chr16 | 30530515 | 30530933 | 419 | ENSG00000169957 | protein_coding | ZNF768 | 3' UTR | 0.01 | up |
| chr5 | 40827541 | 40828082 | 542 | ENSG00000145592 | processed_transcript | RPL37 | 3' UTR | 0.01 | up |
| chr4 | 73836639 | 73836820 | 182 | ENSG00000124875 | protein_coding | CXCL6 | 5' UTR | 0.01 | up |
| chr14 | 100128716 | 100132775 | 4060 | ENSG00000196405 | nonsense_mediated_decay | EVL | 3' UTR | 0.01 | up |
| chr19 | 55485247 | 55485740 | 494 | ENSG00000090971 | protein_coding | NAT14 | 5' UTR | 0.01 | up |
| chr12 | 132593117 | 132593698 | 582 | ENSG00000277186 | lncRNA | AC131212 | Exon | 0.01 | up |
| chr12 | 57931516 | 57933959 | 2444 | ENSG00000257698 | retained_intron | GIHCG | Exon | 0.01 | up |
| chr3 | 149153896 | 149154076 | 181 | ENSG00000163755 | nonsense_mediated_decay | HPS3 | Exon | 0.01 | down |
| chr2 | 134953258 | 134953738 | 481 | ENSG00000082258 | protein_coding | CCNT2 | 3' UTR | 0.01 | down |
| chr8 | 94391619 | 94393471 | 1853 | ENSG00000197275 | retained_intron | RAD54B | 3' UTR | 0.01 | down |
| chr2 | 28849985 | 28850492 | 508 | ENSG00000163806 | processed_transcript | SPDYA | 3' UTR | 0.01 | down |
| chr8 | 144355380 | 144357397 | 2018 | ENSG00000182325 | processed_transcript | FBXL6 | 5' UTR | 0.01 | down |
| chr10 | 71964603 | 72007223 | 42621 | ENSG00000122863 | protein_coding | CHST3 | 5' UTR | 0.01 | down |
| chr14 | 104719599 | 104720289 | 691 | ENSG00000203485 | processed_transcript | INF2 | 3' UTR | 0.01 | down |
| chr3 | 127022303 | 127026490 | 4188 | ENSG00000114554 | retained_intron | PLXNA1 | Exon | 0.01 | down |
| chr1 | 227767080 | 227775791 | 8712 | ENSG00000286389 | lncRNA | AL731702 | 3' UTR | 0.01 | up |
| chr7 | 73737179 | 73737329 | 151 | ENSG00000106077 | retained_intron | ABHD11 | 3' UTR | 0.01 | up |
| chr4 | 87134682 | 87136647 | 1966 | ENSG00000172493 | retained_intron | AFF1 | 3' UTR | 0.01 | up |
| chr10 | 89339866 | 89340971 | 1106 | ENSG00000119917 | protein_coding | IFIT3 | 3' UTR | 0.01 | up |
| chr1 | 94170663 | 94170814 | 152 | ENSG00000137962 | retained_intron | ARHGAP29 | 3' UTR | 0.01 | up |
| X | 18604182 | 18608814 | 4633 | ENSG00000008086 | retained_intron | CDKL5 | Exon | 0.01 | up |
| chr17 | 8115662 | 8116929 | 1268 | ENSG00000179148 | protein_coding | ALOXE3 | Exon | 0.01 | down |
| chr20 | 45931669 | 45932090 | 422 | ENSG00000285796 | lncRNA | AL162458 | Exon | 0.01 | down |
| chr3 | 157147932 | 157148563 | 632 | ENSG00000163660 | retained_intron | CCNL1 | 3' UTR | 0.01 | down |
| chr17 | 7553637 | 7556799 | 3163 | ENSG00000161955 | protein_coding | TNFSF13 | 3' UTR | 0.01 | down |
| chr20 | 23369698 | 23370824 | 1127 | ENSG00000125812 | processed_transcript | GZF1 | 3' UTR | 0.01 | up |
| chr3 | 42591595 | 42594986 | 3392 | ENSG00000008324 | retained_intron | SS18L2 | 3' UTR | 0.01 | up |
| chr19 | 55386990 | 55387081 | 92 | ENSG00000108107 | protein_coding | RPL28 | 3' UTR | 0.01 | up |
| chr1 | 214333810 | 214334051 | 242 | ENSG00000143499 | protein_coding | SMYD2 | Exon | 0.01 | down |
| chr2 | 135349988 | 135353423 | 3436 | ENSG00000121988 | processed_transcript | ZRANB3 | 5' UTR | 0.01 | down |
| chr6 | 30006179 | 30006706 | 528 | ENSG00000204622 | processed_transcript | HLA-J | Exon | 0.01 | down |
| chr2 | 113460675 | 113462574 | 1900 | ENSG00000136682 | retained_intron | CBWD2 | 3' UTR | 0.01 | down |
| chr16 | 28713956 | 28720658 | 6703 | ENSG00000184110 | processed_transcript | EIF3C | Exon | 0.01 | down |
| chr13 | 36335059 | 36335659 | 601 | ENSG00000133104 | processed_transcript | SPART | Exon | 0.01 | down |
| chr6 | 32067959 | 32068575 | 617 | ENSG00000168477 | protein_coding | TNXB | 3' UTR | 0.01 | down |
| chr12 | 57748816 | 57749145 | 330 | ENSG00000135452 | retained_intron | TSPAN31 | 3' UTR | 0.01 | down |
| chr11 | 62803791 | 62804091 | 301 | ENSG00000162231 | protein_coding | NXF1 | 5' UTR | 0.01 | down |
| chr8 | 23328534 | 23328684 | 151 | ENSG00000134013 | retained_intron | LOXL2 | 3' UTR | 0.01 | down |
| chr10 | 100923985 | 100924885 | 901 | ENSG00000119906 | protein_coding | SLF2 | Exon | 0.01 | up |
| chr16 | 88715097 | 88715396 | 300 | ENSG00000174177 | retained_intron | CTU2 | 3' UTR | 0.01 | up |
| chr6 | 31534387 | 31534748 | 362 | ENSG00000198563 | protein_coding | DDX39B | Exon | 0.01 | up |
| chr2 | 219235527 | 219235737 | 211 | ENSG00000163516 | retained_intron | ANKZF1 | Exon | 0.01 | up |
| chr2 | 230814017 | 230817926 | 3910 | ENSG00000135932 | retained_intron | CAB39 | Exon | 0.01 | up |
| chr6 | 7881551 | 7881672 | 122 | ENSG00000239264 | retained_intron | TXNDC5 | 3' UTR | 0.01 | up |
| chr3 | 194404500 | 194404801 | 302 | ENSG00000178732 | protein_coding | GP5 | 3' UTR | 0.01 | up |
| chr4 | 99062449 | 99062659 | 211 | ENSG00000164024 | protein_coding | METAP1 | 3' UTR | 0.01 | up |
| chr15 | 78024164 | 78024344 | 181 | ENSG00000167202 | retained_intron | TBC1D2B | Exon | 0.01 | up |
| chr4 | 125450296 | 125451135 | 840 | ENSG00000196159 | retained_intron | FAT4 | Exon | 0.01 | up |
| chr17 | 43104152 | 43104938 | 787 | ENSG00000012048 | protein_coding | BRCA1 | 3' UTR | 0.01 | down |
| chr7 | 66154207 | 66154268 | 62 | ENSG00000241258 | processed_transcript | CRCP | 3' UTR | 0.01 | down |
| chr16 | 18808966 | 18809477 | 512 | ENSG00000157106 | retained_intron | SMG1 | 3' UTR | 0.01 | down |
| X | 7351501 | 7352041 | 541 | ENSG00000101846 | protein_coding | STS | 3' UTR | 0.01 | down |
| chr22 | 41584259 | 41584470 | 212 | ENSG00000100417 | retained_intron | PMM1 | 3' UTR | 0.01 | down |
| chr7 | 74691032 | 74711045 | 20014 | ENSG00000263001 | retained_intron | GTF2I | Exon | 0.01 | down |
| chr1 | 40509780 | 40516021 | 6242 | ENSG00000227278 | lncRNA | AL603839 | 3' UTR | 0.01 | up |
| chr16 | 71745475 | 71746659 | 1185 | ENSG00000166747 | protein_coding | AP1G1 | 3' UTR | 0.01 | up |
| chr11 | 66664997 | 66665296 | 300 | ENSG00000173914 | processed_transcript | RBM4B | 3' UTR | 0.01 | up |
| chr22 | 37612996 | 37613177 | 182 | ENSG00000100083 | protein_coding | GGA1 | 5' UTR | 0.01 | up |
| chr17 | 59707647 | 59707858 | 212 | ENSG00000062716 | nonsense_mediated_decay | VMP1 | 5' UTR | 0.01 | up |
| chr19 | 57419698 | 57419994 | 297 | ENSG00000186272 | retained_intron | ZNF17 | 3' UTR | 0.01 | up |
| chr14 | 23521006 | 23521486 | 481 | ENSG00000136367 | processed_transcript | ZFHX2 | 3' UTR | 0.01 | up |
| chr4 | 169720767 | 169721067 | 301 | ENSG00000109572 | protein_coding | CLCN3 | 3' UTR | 0.01 | up |
| chr1 | 214647215 | 214652915 | 5701 | ENSG00000117724 | protein_coding | CENPF | Exon | 0.01 | up |
| chr2 | 200481215 | 200481425 | 211 | ENSG00000196141 | processed_transcript | SPATS2L | 3' UTR | 0.01 | down |
| chr1 | 221702137 | 221702554 | 418 | ENSG00000143507 | nonsense_mediated_decay | DUSP10 | 3' UTR | 0.01 | down |
| chr12 | 93738286 | 93738616 | 331 | ENSG00000169372 | processed_transcript | CRADD | 3' UTR | 0.01 | down |
| chr7 | 30628596 | 30628747 | 152 | ENSG00000106105 | retained_intron | GARS1 | 3' UTR | 0.01 | down |
| chr20 | 46733868 | 46734317 | 450 | ENSG00000197496 | processed_transcript | SLC2A10 | 3' UTR | 0.01 | down |
| chr1 | 46303307 | 46303608 | 302 | ENSG00000132128 | protein_coding | LRRC41 | 5' UTR | 0.01 | down |
| chr6 | 43179956 | 43180523 | 568 | ENSG00000112659 | retained_intron | CUL9 | 3' UTR | 0.01 | down |
| chr19 | 2015300 | 2015511 | 212 | ENSG00000133243 | nonsense_mediated_decay | BTBD2 | Exon | 0.01 | down |
| chr1 | 19151596 | 19152409 | 814 | ENSG00000127481 | protein_coding | UBR4 | Exon | 0.01 | down |
| chr17 | 4969194 | 4969405 | 212 | ENSG00000108509 | retained_intron | CAMTA2 | 3' UTR | 0.01 | up |
| chr11 | 46811082 | 46820790 | 9709 | ENSG00000175216 | protein_coding | CKAP5 | Exon | 0.01 | up |
| chr14 | 103527637 | 103527997 | 361 | ENSG00000260285 | lncRNA | AL133367 | Exon | 0.01 | up |
| chr12 | 57070703 | 57070884 | 182 | ENSG00000166881 | nonsense_mediated_decay | NEMP1 | Exon | 0.01 | up |
| chr19 | 46390470 | 46390830 | 361 | ENSG00000011485 | retained_intron | PPP5C | 3' UTR | 0.01 | up |
| chr13 | 102676322 | 102678463 | 2142 | ENSG00000134900 | retained_intron | TPP2 | 3' UTR | 0.01 | up |
| chr19 | 53441839 | 53442049 | 211 | ENSG00000241015 | transcribed_processed_pseudogene | TPM3P9 | Exon | 0.01 | up |
| chr1 | 201999407 | 201999887 | 481 | ENSG00000234678 | lncRNA | ELF3-AS1 | Exon | 0.01 | down |
| chr19 | 53067923 | 53069003 | 1081 | ENSG00000170949 | protein_coding | ZNF160 | 3' UTR | 0.01 | down |
| chr14 | 60133784 | 60134719 | 936 | ENSG00000126773 | nonsense_mediated_decay | PCNX4 | 3' UTR | 0.01 | down |
| chr8 | 47712888 | 47713367 | 480 | ENSG00000164808 | protein_coding | SPIDR | Exon | 0.01 | down |
| chr18 | 10550851 | 10551300 | 450 | ENSG00000134265 | retained_intron | NAPG | 3' UTR | 0.01 | down |
| chr1 | 19251804 | 19252013 | 210 | ENSG00000053372 | protein_coding | MRTO4 | 5' UTR | 0.01 | down |
| chr6 | 57182085 | 57184351 | 2267 | ENSG00000112208 | protein_coding | BAG2 | 3' UTR | 0.01 | down |
| chr6 | 1612070 | 1612789 | 720 | ENSG00000054598 | protein_coding | FOXC1 | 3' UTR | 0.01 | down |
| X | 48597604 | 48597904 | 301 | ENSG00000101940 | retained_intron | WDR13 | 5' UTR | 0.01 | up |
| chr15 | 29732686 | 29737347 | 4662 | ENSG00000104067 | retained_intron | TJP1 | Exon | 0.01 | up |
| chr5 | 73078208 | 73081888 | 3681 | ENSG00000157107 | retained_intron | FCHO2 | Exon | 0.01 | up |
| chr19 | 1065271 | 1065572 | 302 | ENSG00000180448 | protein_coding | ARHGAP45 | 3' UTR | 0.01 | up |
| chr4 | 38967190 | 38971117 | 3928 | ENSG00000121895 | processed_transcript | TMEM156 | 3' UTR | 0.01 | up |
| chr3 | 180948438 | 180949286 | 849 | ENSG00000114416 | retained_intron | FXR1 | Exon | 0.01 | up |
| chr7 | 75361426 | 75364633 | 3208 | ENSG00000205583 | transcribed_unprocessed_pseudogene | STAG3L1 | Exon | 0.01 | up |
| chr8 | 128016963 | 128027555 | 10593 | ENSG00000249859 | lncRNA | PVT1 | Exon | 0.01 | up |
| chr19 | 52800782 | 52801082 | 301 | ENSG00000198538 | protein_coding | ZNF28 | Exon | 0.01 | down |
| chr3 | 9422749 | 9424469 | 1721 | ENSG00000168137 | protein_coding | SETD5 | 5' UTR | 0.01 | down |
| X | 134426158 | 134426428 | 271 | ENSG00000165704 | protein_coding | HPRT1 | 3' UTR | 0.01 | down |
| chr17 | 76778287 | 76778647 | 361 | ENSG00000092931 | processed_transcript | MFSD11 | 3' UTR | 0.01 | up |
| chr22 | 35778054 | 35809878 | 31825 | ENSG00000100320 | protein_coding | RBFOX2 | Exon | 0.01 | up |
| chr11 | 6557063 | 6557723 | 661 | ENSG00000179532 | retained_intron | DNHD1 | Exon | 0.01 | up |
| chr12 | 7094640 | 7095300 | 661 | ENSG00000139178 | processed_transcript | C1RL | 3' UTR | 0.01 | up |
| chr10 | 22541967 | 22567612 | 25646 | ENSG00000150867 | processed_transcript | PIP4K2A | Exon | 0.01 | up |
| chr21 | 41404853 | 41408315 | 3463 | ENSG00000183486 | retained_intron | MX2 | 3' UTR | 0.01 | up |
| chr16 | 29457694 | 29457843 | 150 | ENSG00000181625 | retained_intron | SLX1B | Exon | 0.01 | down |
| chr1 | 225144403 | 225147185 | 2783 | ENSG00000185842 | nonsense_mediated_decay | DNAH14 | 3' UTR | 0.01 | down |
| chr14 | 103712925 | 103715444 | 2520 | ENSG00000126215 | protein_coding | XRCC3 | 5' UTR | 0.01 | down |
| chr4 | 147875026 | 147881907 | 6882 | ENSG00000071205 | protein_coding | ARHGAP10 | Exon | 0.01 | down |
| chr1 | 19217334 | 19217963 | 630 | ENSG00000127463 | retained_intron | EMC1 | 3' UTR | 0.01 | down |
| chr18 | 58349600 | 58360013 | 10414 | ENSG00000049759 | processed_transcript | NEDD4L | 3' UTR | 0.01 | up |
| chr19 | 33194441 | 33202861 | 8421 | ENSG00000130881 | retained_intron | LRP3 | 5' UTR | 0.01 | up |
| chr22 | 35321963 | 35322144 | 182 | ENSG00000100284 | retained_intron | TOM1 | 3' UTR | 0.01 | up |
| chr17 | 4197562 | 4208082 | 10521 | ENSG00000185722 | retained_intron | ANKFY1 | 3' UTR | 0.01 | up |
| chr5 | 477365 | 477974 | 610 | ENSG00000225138 | lncRNA | SLC9A3-AS1 | Exon | 0.01 | up |
| chr22 | 39080224 | 39080702 | 479 | ENSG00000239713 | retained_intron | APOBEC3G | Exon | 0.01 | up |
| chr3 | 44750314 | 44750615 | 302 | ENSG00000163807 | processed_transcript | KIAA1143 | 3' UTR | 0.01 | down |
| chr2 | 74833276 | 74833957 | 682 | ENSG00000272711 | lncRNA | AC019069 | Exon | 0.01 | down |
| X | 150445607 | 150470264 | 24658 | ENSG00000013619 | protein_coding | MAMLD1 | Exon | 0.01 | down |
| chr7 | 44114680 | 44114800 | 121 | ENSG00000106628 | retained_intron | POLD2 | 3' UTR | 0.01 | down |
| chr14 | 102708464 | 102721503 | 13040 | ENSG00000089902 | retained_intron | RCOR1 | Exon | 0.01 | up |
| chr15 | 64204526 | 64214046 | 9521 | ENSG00000169118 | protein_coding | CSNK1G1 | 3' UTR | 0.01 | up |
| chr14 | 20289664 | 20292170 | 2507 | ENSG00000136319 | processed_transcript | TTC5 | 3' UTR | 0.01 | up |
| chr7 | 149069729 | 149071009 | 1281 | ENSG00000197362 | protein_coding | ZNF786 | 3' UTR | 0.01 | up |
| chr17 | 6641731 | 6641971 | 241 | ENSG00000129235 | processed_transcript | TXNDC17 | 3' UTR | 0.01 | up |
| chr17 | 47694080 | 47695344 | 1265 | ENSG00000198933 | protein_coding | TBKBP1 | 5' UTR | 0.01 | up |
| chr6 | 20546453 | 20558598 | 12146 | ENSG00000145996 | protein_coding | CDKAL1 | 3' UTR | 0.01 | up |
| chr10 | 73727418 | 73730466 | 3049 | ENSG00000242288 | lncRNA | BMS1P4-AGAP5 | Exon | 0.01 | up |
| chr10 | 73727420 | 73730487 | 3068 | ENSG00000242288 | lncRNA | BMS1P4-AGAP5 | Exon | 0.01 | up |
| chr2 | 112549285 | 112550773 | 1489 | ENSG00000125630 | retained_intron | POLR1B | 3' UTR | 0.01 | up |
| chr16 | 19211433 | 19224753 | 13321 | ENSG00000103528 | protein_coding | SYT17 | Exon | 0.01 | up |
| chr6 | 17609262 | 17609502 | 241 | ENSG00000137414 | protein_coding | FAM8A1 | 3' UTR | 0.01 | down |
| chr22 | 23659921 | 23678706 | 18786 | ENSG00000244723 | unprocessed_pseudogene | ASLP1 | Exon | 0.01 | down |
| chr17 | 63237914 | 63238064 | 151 | ENSG00000170921 | protein_coding | TANC2 | Exon | 0.01 | down |
| chr3 | 197817254 | 197823006 | 5753 | ENSG00000186001 | processed_transcript | LRCH3 | 3' UTR | 0.01 | down |
| chr1 | 146976979 | 146983007 | 6029 | ENSG00000268043 | protein_coding | NBPF12 | Exon | 0.01 | down |
| chr3 | 160437928 | 160448765 | 10838 | ENSG00000213186 | protein_coding | TRIM59 | 3' UTR | 0.01 | down |
| chr21 | 10473116 | 10482817 | 9702 | ENSG00000273840 | lncRNA | AF254983 | Exon | 0.01 | down |
| chr6 | 83211772 | 83212071 | 300 | ENSG00000013375 | protein_coding | PGM3 | 3' UTR | 0.01 | up |
| X | 41338446 | 41338626 | 181 | ENSG00000215301 | retained_intron | DDX3X | 3' UTR | 0.01 | up |
| chr21 | 42910462 | 42910762 | 301 | ENSG00000160194 | processed_transcript | NDUFV3 | 3' UTR | 0.01 | up |
| chr10 | 71322850 | 71323000 | 151 | ENSG00000198246 | nonsense_mediated_decay | SLC29A3 | 5' UTR | 0.01 | up |
| chr12 | 48691355 | 48691506 | 152 | ENSG00000129315 | retained_intron | CCNT1 | 3' UTR | 0.01 | down |
| chr22 | 50768129 | 50768309 | 181 | ENSG00000079974 | processed_transcript | RABL2B | 3' UTR | 0.01 | down |
| chr5 | 134156390 | 134156541 | 152 | ENSG00000113558 | retained_intron | SKP1 | 3' UTR | 0.01 | down |
| chr7 | 193766 | 195294 | 1529 | ENSG00000177706 | processed_transcript | FAM20C | Exon | 0.01 | down |
| chr16 | 30067876 | 30068206 | 331 | ENSG00000149925 | protein_coding | ALDOA | Exon | 0.01 | down |
| chr7 | 23604264 | 23642980 | 38717 | ENSG00000227436 | processed_pseudogene | FCF1P1 | 5' UTR | 0.01 | down |
| chr8 | 92886129 | 92916992 | 30864 | ENSG00000248858 | TEC | FLJ46284 | 3' UTR | 0.01 | down |
| chr12 | 121442242 | 121442693 | 452 | ENSG00000089094 | retained_intron | KDM2B | Exon | 0.01 | up |
| chr4 | 88727183 | 88728562 | 1380 | ENSG00000138640 | retained_intron | FAM13A | 3' UTR | 0.01 | up |
| chr17 | 6641163 | 6641344 | 182 | ENSG00000129235 | processed_transcript | TXNDC17 | Exon | 0.01 | up |
| chr15 | 71731103 | 71731344 | 242 | ENSG00000278570 | protein_coding | NR2E3 | Exon | 0.01 | up |
| chr12 | 111449113 | 111449263 | 151 | ENSG00000111252 | protein_coding | SH2B3 | 3' UTR | 0.01 | up |
| chr7 | 141738363 | 141738640 | 278 | ENSG00000106028 | retained_intron | SSBP1 | 5' UTR | 0.01 | up |
| chr2 | 61278235 | 61280257 | 2023 | ENSG00000115464 | protein_coding | USP34 | Exon | 0.01 | up |
| chr3 | 179328321 | 179329973 | 1653 | ENSG00000121864 | processed_transcript | ZNF639 | Exon | 0.01 | up |
| chr7 | 94628233 | 94628384 | 152 | ENSG00000127990 | nonsense_mediated_decay | SGCE | 3' UTR | 0.01 | up |
| chr5 | 33011349 | 33016995 | 5647 | ENSG00000251281 | lncRNA | AC034223 | Exon | 0.01 | down |
| chr12 | 54011098 | 54011934 | 837 | ENSG00000037965 | protein_coding | HOXC8 | 3' UTR | 0.01 | down |
| chr13 | 72759470 | 72760600 | 1131 | ENSG00000083520 | nonsense_mediated_decay | DIS3 | 3' UTR | 0.01 | down |
| chr20 | 47627099 | 47628514 | 1416 | ENSG00000124151 | processed_transcript | NCOA3 | Exon | 0.01 | down |
| chr18 | 58044675 | 58045536 | 862 | ENSG00000049759 | processed_transcript | NEDD4L | 3' UTR | 0.01 | down |
| X | 40051846 | 40052355 | 510 | ENSG00000183337 | retained_intron | BCOR | 3' UTR | 0.01 | down |
| chr9 | 134440015 | 134440585 | 571 | ENSG00000186350 | protein_coding | RXRA | 3' UTR | 0.01 | up |
| chr13 | 24500370 | 24503730 | 3361 | ENSG00000102699 | protein_coding | PARP4 | Exon | 0.01 | up |
| chr12 | 103930375 | 103931691 | 1317 | ENSG00000166598 | retained_intron | HSP90B1 | 5' UTR | 0.01 | up |
| X | 53078279 | 53078909 | 631 | ENSG00000184205 | retained_intron | TSPYL2 | 3' UTR | 0.01 | up |
| chr8 | 97726037 | 97726278 | 242 | ENSG00000147649 | retained_intron | MTDH | 3' UTR | 0.01 | up |
| chr1 | 11654185 | 11655785 | 1601 | ENSG00000116661 | protein_coding | FBXO2 | 5' UTR | 0.01 | up |
| chr22 | 20119066 | 20119217 | 152 | ENSG00000099901 | protein_coding | RANBP1 | 3' UTR | 0.01 | up |
| chr2 | 112756473 | 112760718 | 4246 | ENSG00000169607 | retained_intron | CKAP2L | Exon | 0.01 | up |
| X | 136044874 | 136045144 | 271 | ENSG00000198689 | retained_intron | SLC9A6 | 3' UTR | 0.01 | down |
| chr10 | 103882277 | 103882667 | 391 | ENSG00000260461 | lncRNA | AL133355 | 3' UTR | 0.01 | down |
| chr1 | 214029747 | 214030753 | 1007 | ENSG00000272167 | lncRNA | AL606537 | Exon | 0.01 | down |
| chr1 | 175010020 | 175010321 | 302 | ENSG00000116161 | retained_intron | CACYBP | 3' UTR | 0.01 | down |
| chr2 | 191392197 | 191401652 | 9456 | ENSG00000128641 | retained_intron | MYO1B | Exon | 0.01 | down |
| chr2 | 27378888 | 27380407 | 1520 | ENSG00000163795 | processed_transcript | ZNF513 | 5' UTR | 0.01 | up |
| chr18 | 48758014 | 48760324 | 2311 | ENSG00000134030 | retained_intron | CTIF | Exon | 0.01 | up |
| chr17 | 37085590 | 37086040 | 451 | ENSG00000278540 | protein_coding | ACACA | 3' UTR | 0.01 | up |
| chr20 | 50313244 | 50314084 | 841 | ENSG00000233077 | lncRNA | LINC01271 | Exon | 0.01 | up |
| chr10 | 51697255 | 51697496 | 242 | ENSG00000177613 | protein_coding | CSTF2T | 3' UTR | 0.01 | up |
| chr6 | 29723388 | 29723622 | 235 | ENSG00000204642 | processed_transcript | HLA-F | Exon | 0.01 | up |
| chr6 | 29723409 | 29723619 | 211 | ENSG00000204642 | processed_transcript | HLA-F | 5' UTR | 0.01 | up |
| chr12 | 133006299 | 133006599 | 301 | ENSG00000198393 | retained_intron | ZNF26 | Exon | 0.01 | down |
| chr6 | 32048628 | 32049518 | 891 | ENSG00000168477 | retained_intron | TNXB | Exon | 0.01 | down |
| chr7 | 74224084 | 74225097 | 1014 | ENSG00000086730 | retained_intron | LAT2 | 3' UTR | 0.01 | down |
| chr22 | 30937879 | 30938365 | 487 | ENSG00000133422 | retained_intron | MORC2 | Exon | 0.01 | down |
| chr20 | 63704889 | 63705159 | 271 | ENSG00000101246 | retained_intron | ARFRP1 | Exon | 0.01 | down |
| chr12 | 113942359 | 113946391 | 4033 | ENSG00000122965 | retained_intron | RBM19 | Exon | 0.01 | down |
| chr17 | 18018092 | 18018570 | 479 | ENSG00000171953 | retained_intron | ATPAF2 | 3' UTR | 0.01 | up |
| chr8 | 48050490 | 48061293 | 10804 | ENSG00000169139 | retained_intron | UBE2V2 | 3' UTR | 0.01 | up |
| chr1 | 156594598 | 156595017 | 420 | ENSG00000160818 | retained_intron | GPATCH4 | 3' UTR | 0.01 | up |
| chr1 | 15409917 | 15410126 | 210 | ENSG00000142634 | protein_coding | EFHD2 | 5' UTR | 0.01 | up |
| chr22 | 31325803 | 31325984 | 182 | ENSG00000100105 | retained_intron | PATZ1 | 3' UTR | 0.01 | up |
| chr1 | 3823164 | 3826434 | 3271 | ENSG00000116198 | retained_intron | CEP104 | 3' UTR | 0.01 | up |
| chr1 | 52474833 | 52481595 | 6763 | ENSG00000134744 | retained_intron | TUT4 | 3' UTR | 0.01 | up |
| chr17 | 75793940 | 75808960 | 15021 | ENSG00000132478 | retained_intron | UNK | 3' UTR | 0.01 | up |
| chr12 | 103763665 | 103766818 | 3154 | ENSG00000257681 | lncRNA | AC025265 | Exon | 0.01 | down |
| chr1 | 155614755 | 155615668 | 914 | ENSG00000232519 | lncRNA | AL353807 | Exon | 0.01 | down |
| X | 120250930 | 120253862 | 2933 | ENSG00000177485 | protein_coding | ZBTB33 | 5' UTR | 0.01 | down |
| chr18 | 54378179 | 54378719 | 541 | ENSG00000166845 | nonsense_mediated_decay | C18orf54 | 3' UTR | 0.01 | down |
| chr21 | 36132545 | 36133055 | 511 | ENSG00000236830 | lncRNA | CBR3-AS1 | Exon | 0.01 | down |
| chr8 | 18801296 | 18803239 | 1944 | ENSG00000156011 | protein_coding | PSD3 | 3' UTR | 0.01 | down |
| chr8 | 143839968 | 143840450 | 483 | ENSG00000185189 | retained_intron | NRBP2 | 5' UTR | 0.01 | down |
| chr16 | 71921371 | 71921611 | 241 | ENSG00000182149 | retained_intron | IST1 | Exon | 0.01 | down |
| chr6 | 89081229 | 89081989 | 761 | ENSG00000146278 | protein_coding | PNRC1 | 5' UTR | 0.01 | up |
| chr15 | 30904499 | 30905788 | 1290 | ENSG00000198690 | nonsense_mediated_decay | FAN1 | 5' UTR | 0.01 | up |
| chr1 | 37984767 | 37989955 | 5189 | ENSG00000183431 | processed_transcript | SF3A3 | Exon | 0.01 | up |
| chr17 | 64043368 | 64045389 | 2022 | ENSG00000178607 | retained_intron | ERN1 | 3' UTR | 0.01 | up |
| chr2 | 200572713 | 200583468 | 10756 | ENSG00000138356 | protein_coding | AOX1 | 3' UTR | 0.01 | up |
| chr16 | 23067458 | 23067819 | 362 | ENSG00000260566 | lncRNA | AC127459 | 3' UTR | 0.01 | up |
| chr2 | 111873626 | 111880549 | 6924 | ENSG00000153107 | protein_coding | ANAPC1 | Exon | 0.01 | up |
| chr19 | 57942084 | 57943997 | 1914 | ENSG00000152454 | protein_coding | ZNF256 | 3' UTR | 0.01 | down |
| chr16 | 83808007 | 83808278 | 272 | ENSG00000230989 | nonsense_mediated_decay | HSBP1 | 3' UTR | 0.01 | down |
| chr1 | 31586737 | 31587626 | 890 | ENSG00000142910 | processed_transcript | TINAGL1 | 3' UTR | 0.01 | down |
| chr15 | 64204506 | 64214055 | 9550 | ENSG00000169118 | protein_coding | CSNK1G1 | 3' UTR | 0.01 | up |
| chr9 | 128473630 | 128485374 | 11745 | ENSG00000136811 | protein_coding | ODF2 | Exon | 0.01 | up |
| chr10 | 61940210 | 62000133 | 59924 | ENSG00000150347 | protein_coding | ARID5B | Exon | 0.01 | up |
| chr1 | 173476698 | 173476998 | 301 | ENSG00000203739 | lncRNA | AL645568 | Exon | 0.01 | up |
| chr1 | 98043712 | 98044162 | 451 | ENSG00000225206 | lncRNA | MIR137HG | Exon | 0.01 | up |
| chr6 | 63712638 | 63713089 | 452 | ENSG00000118482 | protein_coding | PHF3 | 3' UTR | 0.01 | up |
| chr19 | 23920995 | 23921235 | 241 | ENSG00000213967 | protein_coding | ZNF726 | 3' UTR | 0.01 | down |
| chr17 | 81121478 | 81121988 | 511 | ENSG00000181409 | nonsense_mediated_decay | AATK | Exon | 0.01 | down |
| chr12 | 15631541 | 15631722 | 182 | ENSG00000151491 | retained_intron | EPS8 | 3' UTR | 0.01 | down |
| chr8 | 23403964 | 23404175 | 212 | ENSG00000134013 | protein_coding | LOXL2 | 5' UTR | 0.01 | down |
| chr5 | 137755858 | 137759007 | 3150 | ENSG00000287067 | transcribed_processed_pseudogene | AC106791 | Exon | 0.01 | down |
| chr16 | 89526460 | 89527180 | 721 | ENSG00000197912 | nonsense_mediated_decay | SPG7 | 3' UTR | 0.01 | down |
| chr7 | 100319221 | 100319760 | 540 | ENSG00000078319 | processed_transcript | PMS2P1 | Exon | 0.01 | up |
| chr20 | 50313246 | 50314084 | 839 | ENSG00000203999 | lncRNA | LINC01270 | Exon | 0.01 | up |
| chr2 | 96609037 | 96610580 | 1544 | ENSG00000114982 | retained_intron | KANSL3 | 3' UTR | 0.01 | up |
| chr8 | 2000414 | 2001372 | 959 | ENSG00000270988 | lncRNA | AC019257 | 5' UTR | 0.01 | up |
| chr10 | 87009122 | 87009693 | 572 | ENSG00000261011 | transcribed_unprocessed_pseudogene | AL136982 | Exon | 0.01 | up |
| chr10 | 110133375 | 110133826 | 452 | ENSG00000148700 | processed_transcript | ADD3 | 3' UTR | 0.01 | up |
| chr2 | 177617574 | 177617964 | 391 | ENSG00000197557 | protein_coding | TTC30A | Exon | 0.01 | down |
| chr16 | 67098761 | 67099031 | 271 | ENSG00000067955 | retained_intron | CBFB | 3' UTR | 0.01 | down |
| chr3 | 184172090 | 184172210 | 121 | ENSG00000161203 | protein_coding | AP2M1 | 3' UTR | 0.01 | down |
| chr19 | 15373117 | 15379038 | 5922 | ENSG00000105127 | nonsense_mediated_decay | AKAP8 | Exon | 0.01 | down |
| chr19 | 58341526 | 58342332 | 807 | ENSG00000268895 | lncRNA | A1BG-AS1 | 3' UTR | 0.01 | down |
| chr22 | 29788608 | 29790491 | 1884 | ENSG00000100325 | processed_transcript | ASCC2 | 3' UTR | 0.01 | up |
| chr13 | 102805323 | 102816614 | 11292 | ENSG00000270181 | nonsense_mediated_decay | BIVM-ERCC5 | 3' UTR | 0.01 | up |
| chr15 | 48825148 | 48826108 | 961 | ENSG00000103995 | non_stop_decay | CEP152 | 3' UTR | 0.01 | down |
| chr17 | 2373369 | 2373639 | 271 | ENSG00000141258 | retained_intron | SGSM2 | Exon | 0.01 | down |
| chr14 | 77341410 | 77343469 | 2060 | ENSG00000009830 | protein_coding | POMT2 | 3' UTR | 0.01 | down |
| chr16 | 4803292 | 4803801 | 510 | ENSG00000067836 | retained_intron | ROGDI | 3' UTR | 0.01 | down |
| chr20 | 49371695 | 49374545 | 2851 | ENSG00000158445 | processed_transcript | KCNB1 | 3' UTR | 0.01 | down |
| chr1 | 32231657 | 32234119 | 2463 | ENSG00000220785 | processed_transcript | MTMR9LP | Exon | 0.01 | up |
| chr1 | 172570765 | 172579236 | 8472 | ENSG00000094975 | retained_intron | SUCO | 5' UTR | 0.01 | up |
| chr6 | 170578866 | 170579167 | 302 | ENSG00000071994 | retained_intron | PDCD2 | 3' UTR | 0.01 | up |
| chr10 | 87009109 | 87009708 | 600 | ENSG00000261011 | transcribed_unprocessed_pseudogene | AL136982 | Exon | 0.01 | up |
| chr8 | 141188621 | 141188922 | 302 | ENSG00000105339 | retained_intron | DENND3 | 5' UTR | 0.01 | up |
| chr7 | 39571001 | 39572871 | 1871 | ENSG00000241127 | retained_intron | YAE1 | 3' UTR | 0.01 | up |
| chr5 | 95017411 | 95038143 | 20733 | ENSG00000175471 | protein_coding | MCTP1 | Exon | 0.01 | up |
| chr14 | 76202353 | 76202594 | 242 | ENSG00000089916 | nonsense_mediated_decay | GPATCH2L | 3' UTR | 0.00 | down |
| chr15 | 20443506 | 20444392 | 887 | ENSG00000180229 | retained_intron | HERC2P3 | Exon | 0.00 | down |
| chr22 | 41525858 | 41526039 | 182 | ENSG00000100413 | processed_transcript | POLR3H | 3' UTR | 0.00 | down |
| chr22 | 36387816 | 36387967 | 152 | ENSG00000100345 | protein_coding | MYH9 | 5' UTR | 0.00 | down |
| chr4 | 73056911 | 73057481 | 571 | ENSG00000163626 | protein_coding | COX18 | 3' UTR | 0.00 | down |
| chr1 | 22025316 | 22025825 | 510 | ENSG00000070831 | protein_coding | CDC42 | 5' UTR | 0.00 | down |
| chr19 | 49590029 | 49590676 | 648 | ENSG00000142546 | protein_coding | NOSIP | 3' UTR | 0.00 | up |
| chr16 | 23452994 | 23455761 | 2768 | ENSG00000168434 | protein_coding | COG7 | Exon | 0.00 | up |
| X | 119629377 | 119649965 | 20589 | ENSG00000125354 | processed_transcript | SEPTIN6 | 3' UTR | 0.00 | up |
| chr17 | 38749546 | 38749787 | 242 | ENSG00000277258 | protein_coding | PCGF2 | 5' UTR | 0.00 | up |
| chr1 | 145892491 | 145893207 | 717 | ENSG00000244619 | lncRNA | AC243547 | 3' UTR | 0.00 | up |
| chr19 | 49999388 | 49999598 | 211 | ENSG00000105053 | retained_intron | VRK3 | Exon | 0.00 | up |
| chr6 | 2954888 | 2955219 | 332 | ENSG00000124570 | retained_intron | SERPINB6 | Exon | 0.00 | down |
| chr14 | 31859153 | 31859634 | 482 | ENSG00000151413 | retained_intron | NUBPL | 3' UTR | 0.00 | down |
| chr19 | 35544991 | 35545319 | 329 | ENSG00000236144 | lncRNA | TMEM147-AS1 | 3' UTR | 0.00 | down |
| chr17 | 46941534 | 46941865 | 332 | ENSG00000108433 | nonsense_mediated_decay | GOSR2 | 3' UTR | 0.00 | down |
| chr2 | 108782219 | 108784039 | 1821 | ENSG00000163006 | protein_coding | CCDC138 | 3' UTR | 0.00 | down |
| chr6 | 39105314 | 39109359 | 4046 | ENSG00000112167 | protein_coding | SAYSD1 | 3' UTR | 0.00 | down |
| chr20 | 1166721 | 1167231 | 511 | ENSG00000125818 | processed_transcript | PSMF1 | 3' UTR | 0.00 | down |
| chr2 | 85318438 | 85318799 | 362 | ENSG00000152291 | protein_coding | TGOLN2 | 3' UTR | 0.00 | up |
| chr11 | 13417259 | 13421802 | 4544 | ENSG00000148925 | processed_transcript | BTBD10 | 3' UTR | 0.00 | up |
| chr16 | 1822869 | 1826699 | 3831 | ENSG00000063854 | protein_coding | HAGH | 5' UTR | 0.00 | up |
| X | 334231 | 334652 | 422 | ENSG00000167393 | retained_intron | PPP2R3B | 3' UTR | 0.00 | up |
| chr1 | 32786377 | 32786588 | 212 | ENSG00000134684 | retained_intron | YARS1 | 3' UTR | 0.00 | up |
| chr6 | 3076825 | 3077827 | 1003 | ENSG00000137275 | protein_coding | RIPK1 | Exon | 0.00 | up |
| chr14 | 23352672 | 23352912 | 241 | ENSG00000092096 | protein_coding | SLC22A17 | 5' UTR | 0.00 | up |
| chr5 | 80472826 | 80474819 | 1994 | ENSG00000152380 | nonsense_mediated_decay | FAM151B | 3' UTR | 0.00 | down |
| chr1 | 63453892 | 63457269 | 3378 | ENSG00000142856 | processed_transcript | ITGB3BP | Exon | 0.00 | down |
| chr16 | 68564390 | 68565590 | 1201 | ENSG00000184939 | retained_intron | ZFP90 | 3' UTR | 0.00 | down |
| chr19 | 45693496 | 45695496 | 2001 | ENSG00000011478 | nonsense_mediated_decay | QPCTL | Exon | 0.00 | down |
| chr5 | 132756574 | 132757085 | 512 | ENSG00000164402 | processed_transcript | SEPTIN8 | 3' UTR | 0.00 | down |
| chr5 | 135033064 | 135034544 | 1481 | ENSG00000069011 | protein_coding | PITX1 | 5' UTR | 0.00 | down |
| chr22 | 26463939 | 26464749 | 811 | ENSG00000100099 | processed_transcript | HPS4 | 3' UTR | 0.00 | down |
| chr14 | 76762276 | 76762876 | 601 | ENSG00000071246 | protein_coding | VASH1 | 5' UTR | 0.00 | down |
| chr9 | 133789284 | 133812196 | 22913 | ENSG00000123453 | protein_coding | SARDH | Exon | 0.00 | up |
| chr19 | 52554244 | 52563654 | 9411 | ENSG00000167562 | protein_coding | ZNF701 | 3' UTR | 0.00 | up |
| chr12 | 121814868 | 121817525 | 2658 | ENSG00000139718 | protein_coding | SETD1B | Exon | 0.00 | up |
| chr16 | 72112425 | 72119155 | 6731 | ENSG00000118557 | protein_coding | PMFBP1 | 3' UTR | 0.00 | up |
| chr16 | 3129995 | 3132228 | 2234 | ENSG00000263072 | lncRNA | ZNF213-AS1 | Exon | 0.00 | up |
| chr17 | 4182185 | 4183531 | 1347 | ENSG00000185722 | retained_intron | ANKFY1 | 3' UTR | 0.00 | up |
| chr2 | 119223830 | 119230771 | 6942 | ENSG00000115107 | protein_coding | STEAP3 | 5' UTR | 0.00 | up |
| chr12 | 104262371 | 104280722 | 18352 | ENSG00000213442 | processed_pseudogene | RPL18AP3 | Exon | 0.00 | up |
| chr8 | 102311846 | 102313804 | 1959 | ENSG00000104517 | protein_coding | UBR5 | Exon | 0.00 | up |
| chr3 | 50113435 | 50114319 | 885 | ENSG00000003756 | retained_intron | RBM5 | Exon | 0.00 | up |
| chr14 | 91313693 | 91315702 | 2010 | ENSG00000015133 | retained_intron | CCDC88C | Exon | 0.00 | down |
| chr4 | 38137170 | 38137619 | 450 | ENSG00000065882 | retained_intron | TBC1D1 | 3' UTR | 0.00 | down |
| chr1 | 114773995 | 114778979 | 4985 | ENSG00000052723 | processed_transcript | SIKE1 | 3' UTR | 0.00 | down |
| chr22 | 39087414 | 39087743 | 330 | ENSG00000225720 | transcribed_unprocessed_pseudogene | AL031846 | 3' UTR | 0.00 | down |
| chr10 | 44991342 | 44993499 | 2158 | ENSG00000107551 | retained_intron | RASSF4 | 3' UTR | 0.00 | down |
| chr4 | 73057570 | 73058140 | 571 | ENSG00000163626 | protein_coding | COX18 | 3' UTR | 0.00 | down |
| chr8 | 38840579 | 38843508 | 2930 | ENSG00000147526 | retained_intron | TACC1 | Exon | 0.00 | down |
| chr12 | 6577798 | 6578278 | 481 | ENSG00000111642 | retained_intron | CHD4 | 3' UTR | 0.00 | up |
| chr6 | 31969571 | 31969751 | 181 | ENSG00000204351 | processed_transcript | SKIV2L | 3' UTR | 0.00 | up |
| chr6 | 7881576 | 7881696 | 121 | ENSG00000239264 | retained_intron | TXNDC5 | 3' UTR | 0.00 | up |
| chr2 | 25234033 | 25235799 | 1767 | ENSG00000119772 | retained_intron | DNMT3A | 3' UTR | 0.00 | up |
| chr11 | 111726149 | 111726450 | 302 | ENSG00000170145 | processed_transcript | SIK2 | 3' UTR | 0.00 | up |
| chr10 | 30014507 | 30015136 | 630 | ENSG00000165757 | processed_transcript | JCAD | 3' UTR | 0.00 | up |
| chr5 | 34823734 | 34826146 | 2413 | ENSG00000039560 | retained_intron | RAI14 | Exon | 0.00 | up |
| chr16 | 68566668 | 68575802 | 9135 | ENSG00000184939 | retained_intron | ZFP90 | 3' UTR | 0.00 | up |
| chr9 | 108939884 | 108940242 | 359 | ENSG00000119328 | protein_coding | ABITRAM | 3' UTR | 0.00 | up |
| chr5 | 51378569 | 51378959 | 391 | ENSG00000259663 | lncRNA | AC010478 | Exon | 0.00 | up |
| chr2 | 62705677 | 62705858 | 182 | ENSG00000115504 | retained_intron | EHBP1 | 5' UTR | 0.00 | down |
| chr19 | 57397134 | 57411135 | 14002 | ENSG00000186272 | processed_transcript | ZNF17 | 3' UTR | 0.00 | down |
| chr13 | 27648371 | 27650619 | 2249 | ENSG00000186184 | processed_transcript | POLR1D | 3' UTR | 0.00 | down |
| chr3 | 161235290 | 161241068 | 5779 | ENSG00000169251 | processed_transcript | NMD3 | Exon | 0.00 | down |
| chr12 | 68651978 | 68657364 | 5387 | ENSG00000127314 | retained_intron | RAP1B | 3' UTR | 0.00 | down |
| chr4 | 56469764 | 56475710 | 5947 | ENSG00000174780 | retained_intron | SRP72 | 3' UTR | 0.00 | up |
| chr7 | 1057680 | 1059201 | 1522 | ENSG00000164849 | protein_coding | GPR146 | 3' UTR | 0.00 | up |
| X | 120526310 | 120532445 | 6136 | ENSG00000158290 | nonsense_mediated_decay | CUL4B | 3' UTR | 0.00 | up |
| chr2 | 86070086 | 86070177 | 92 | ENSG00000068654 | processed_transcript | POLR1A | Exon | 0.00 | up |
| chr6 | 26430980 | 26431846 | 867 | ENSG00000124549 | processed_transcript | BTN2A3P | Exon | 0.00 | up |
| chr15 | 89899255 | 89899615 | 361 | ENSG00000250021 | processed_transcript | ARPIN-AP3S2 | 3' UTR | 0.00 | down |
| chr22 | 17830109 | 17834478 | 4370 | ENSG00000243156 | protein_coding | MICAL3 | 3' UTR | 0.00 | down |
| chr6 | 26636387 | 26637765 | 1379 | ENSG00000181315 | protein_coding | ZNF322 | 3' UTR | 0.00 | down |
| chr11 | 66058824 | 66059310 | 487 | ENSG00000087365 | retained_intron | SF3B2 | Exon | 0.00 | down |
| chr3 | 48892021 | 48898904 | 6884 | ENSG00000178537 | protein_coding | SLC25A20 | 5' UTR | 0.00 | down |
| chr2 | 159279843 | 159286434 | 6592 | ENSG00000196151 | protein_coding | WDSUB1 | 5' UTR | 0.00 | down |
| chr10 | 70879897 | 70880137 | 241 | ENSG00000166224 | retained_intron | SGPL1 | 3' UTR | 0.00 | down |
| chr16 | 72109749 | 72112912 | 3164 | ENSG00000140829 | retained_intron | DHX38 | 3' UTR | 0.00 | up |
| chr5 | 136174522 | 136177646 | 3125 | ENSG00000113658 | nonsense_mediated_decay | SMAD5 | 3' UTR | 0.00 | up |
| chr16 | 2838866 | 2839555 | 690 | ENSG00000162078 | protein_coding | ZG16B | 3' UTR | 0.00 | down |
| chr17 | 44300979 | 44301429 | 451 | ENSG00000267750 | lncRNA | RUNDC3A-AS1 | Exon | 0.00 | down |
| chr20 | 6115860 | 6122791 | 6932 | ENSG00000101311 | protein_coding | FERMT1 | 5' UTR | 0.00 | down |
| chr5 | 6607210 | 6611073 | 3864 | ENSG00000037474 | nonsense_mediated_decay | NSUN2 | 3' UTR | 0.00 | up |
| chr17 | 6080265 | 6081950 | 1686 | ENSG00000179314 | nonsense_mediated_decay | WSCD1 | 5' UTR | 0.00 | up |
| X | 130014267 | 130014537 | 271 | ENSG00000085185 | protein_coding | BCORL1 | Exon | 0.00 | up |
| X | 71283609 | 71283859 | 251 | ENSG00000147140 | protein_coding | NONO | 5' UTR | 0.00 | up |
| chr1 | 161166411 | 161166621 | 211 | ENSG00000143224 | protein_coding | PPOX | 5' UTR | 0.00 | up |
| chr11 | 72017682 | 72017951 | 270 | ENSG00000251143 | lncRNA | AP002490 | Exon | 0.00 | up |
| chr17 | 38716397 | 38716638 | 242 | ENSG00000275023 | protein_coding | MLLT6 | Exon | 0.00 | up |
| chr2 | 112067011 | 112081069 | 14059 | ENSG00000153214 | processed_transcript | TMEM87B | Exon | 0.00 | up |
| chr1 | 19217065 | 19217275 | 211 | ENSG00000127463 | retained_intron | EMC1 | 3' UTR | 0.00 | up |
| chr5 | 138156985 | 138157256 | 272 | ENSG00000112983 | nonsense_mediated_decay | BRD8 | 3' UTR | 0.00 | up |
| chr17 | 67109410 | 67120409 | 11000 | ENSG00000198265 | processed_transcript | HELZ | 3' UTR | 0.00 | up |
| chr1 | 25960871 | 25961261 | 391 | ENSG00000158006 | processed_transcript | PAFAH2 | 3' UTR | 0.00 | down |
| chr16 | 791268 | 791419 | 152 | ENSG00000127586 | retained_intron | CHTF18 | 3' UTR | 0.00 | down |
| chr1 | 15834503 | 15834742 | 240 | ENSG00000179743 | lncRNA | FLJ37453 | Exon | 0.00 | down |
| chr18 | 50271050 | 50272634 | 1585 | ENSG00000141644 | retained_intron | MBD1 | 3' UTR | 0.00 | down |
| chr9 | 136006536 | 136007196 | 661 | ENSG00000148411 | processed_transcript | NACC2 | 3' UTR | 0.00 | down |
| chr3 | 160433101 | 160434057 | 957 | ENSG00000113810 | retained_intron | SMC4 | 3' UTR | 0.00 | down |
| chr4 | 2938110 | 2940439 | 2330 | ENSG00000249673 | lncRNA | NOP14-AS1 | Exon | 0.00 | down |
| chr9 | 107488964 | 107489597 | 634 | ENSG00000136826 | retained_intron | KLF4 | 5' UTR | 0.00 | down |
| chr18 | 80138100 | 80139358 | 1259 | ENSG00000267270 | lncRNA | PARD6G-AS1 | 3' UTR | 0.00 | down |
| chr10 | 102658019 | 102658318 | 300 | ENSG00000171206 | protein_coding | TRIM8 | 3' UTR | 0.00 | up |
| chr12 | 6688459 | 6689153 | 695 | ENSG00000126746 | protein_coding | ZNF384 | 5' UTR | 0.00 | up |
| chr10 | 102476566 | 102476896 | 331 | ENSG00000138111 | processed_transcript | MFSD13A | 3' UTR | 0.00 | up |
| chr10 | 69189275 | 69191675 | 2401 | ENSG00000156502 | processed_transcript | SUPV3L1 | Exon | 0.00 | up |
| chr19 | 43552219 | 43552830 | 612 | ENSG00000073050 | retained_intron | XRCC1 | Exon | 0.00 | up |
| chr7 | 76074663 | 76074963 | 301 | ENSG00000230882 | processed_pseudogene | AC005077 | Exon | 0.00 | up |
| chr10 | 29458522 | 29468975 | 10454 | ENSG00000224597 | processed_transcript | SVIL-AS1 | Exon | 0.00 | up |
| chr1 | 220253686 | 220254009 | 324 | ENSG00000218283 | processed_pseudogene | MORF4L1P1 | Exon | 0.00 | up |
| chr17 | 58094522 | 58094822 | 301 | ENSG00000279207 | TEC | AC015813 | 3' UTR | 0.00 | up |
| chr19 | 41957959 | 41958227 | 269 | ENSG00000105404 | retained_intron | RABAC1 | Exon | 0.00 | down |
| chr15 | 100861923 | 100885270 | 23348 | ENSG00000184254 | protein_coding | ALDH1A3 | 3' UTR | 0.00 | down |
| X | 148052489 | 148052746 | 258 | ENSG00000219507 | processed_pseudogene | FTH1P8 | Exon | 0.00 | down |
| chr11 | 93784616 | 93788313 | 3698 | ENSG00000042429 | nonsense_mediated_decay | MED17 | 3' UTR | 0.00 | down |
| chr1 | 25834829 | 25859280 | 24452 | ENSG00000127423 | processed_transcript | AUNIP | 3' UTR | 0.00 | up |
| chr12 | 68836721 | 68839934 | 3214 | ENSG00000256664 | processed_pseudogene | AC025423 | 3' UTR | 0.00 | up |
| chr17 | 64547628 | 64555956 | 8329 | ENSG00000108854 | retained_intron | SMURF2 | 3' UTR | 0.00 | up |
| chr7 | 100688847 | 100688997 | 151 | ENSG00000146830 | protein_coding | GIGYF1 | 5' UTR | 0.00 | up |
| chr16 | 20924676 | 20925006 | 331 | ENSG00000102897 | protein_coding | LYRM1 | 3' UTR | 0.00 | up |
| chr7 | 123692826 | 123706354 | 13529 | ENSG00000106299 | protein_coding | WASL | Exon | 0.00 | up |
| chr5 | 96715069 | 96728631 | 13563 | ENSG00000153113 | processed_transcript | CAST | 3' UTR | 0.00 | up |
| chr1 | 44036154 | 44040488 | 4335 | ENSG00000230615 | lncRNA | AL139220 | Exon | 0.00 | up |
| chr19 | 1004585 | 1005302 | 718 | ENSG00000116032 | processed_transcript | GRIN3B | Exon | 0.00 | up |
| chr16 | 3306904 | 3307414 | 511 | ENSG00000162086 | protein_coding | ZNF75A | Exon | 0.00 | down |
| chr1 | 110347145 | 110355974 | 8830 | ENSG00000224699 | lncRNA | LAMTOR5-AS1 | Exon | 0.00 | down |
| chr11 | 62832910 | 62833061 | 152 | ENSG00000256690 | lncRNA | AP001160 | 3' UTR | 0.00 | up |
| chr15 | 60448944 | 60451097 | 2154 | ENSG00000128915 | retained_intron | ICE2 | 3' UTR | 0.00 | up |
| chr6 | 53514233 | 53516180 | 1948 | ENSG00000001084 | protein_coding | GCLC | 3' UTR | 0.00 | up |
| chr8 | 67111998 | 67113937 | 1940 | ENSG00000252637 | rRNA_pseudogene | RNA5SP268 | 3' UTR | 0.00 | up |
| chr19 | 781179 | 781447 | 269 | ENSG00000272473 | lncRNA | AC006273 | Exon | 0.00 | up |
| chr14 | 24210470 | 24210819 | 350 | ENSG00000254505 | retained_intron | CHMP4A | 3' UTR | 0.00 | up |
| chr19 | 36639364 | 36639842 | 479 | ENSG00000197808 | protein_coding | ZNF461 | 3' UTR | 0.00 | up |
| chr19 | 47045476 | 47045775 | 300 | ENSG00000130751 | retained_intron | NPAS1 | 3' UTR | 0.00 | down |
| chr16 | 5086421 | 5087080 | 660 | ENSG00000033011 | processed_transcript | ALG1 | 3' UTR | 0.00 | down |
| chr13 | 38354857 | 38359501 | 4645 | ENSG00000120686 | protein_coding | UFM1 | 3' UTR | 0.00 | down |
| chr19 | 18443681 | 18443862 | 182 | ENSG00000105656 | retained_intron | ELL | 3' UTR | 0.00 | down |
| chr18 | 692391 | 692901 | 511 | ENSG00000132199 | retained_intron | ENOSF1 | Exon | 0.00 | down |
| X | 153345617 | 153347384 | 1768 | ENSG00000063587 | retained_intron | ZNF275 | 5' UTR | 0.00 | down |
| chr3 | 28323512 | 28326593 | 3082 | ENSG00000163512 | retained_intron | AZI2 | 3' UTR | 0.00 | down |
| chr5 | 172768302 | 172769751 | 1450 | ENSG00000120129 | protein_coding | DUSP1 | 3' UTR | 0.00 | up |
| chr16 | 8847706 | 8848724 | 1019 | ENSG00000260276 | lncRNA | AC022167 | Exon | 0.00 | up |
| chr14 | 54418403 | 54420218 | 1816 | ENSG00000100526 | protein_coding | CDKN3 | 3' UTR | 0.00 | up |
| chr18 | 58981547 | 58984771 | 3225 | ENSG00000074657 | retained_intron | ZNF532 | 3' UTR | 0.00 | up |
| chr5 | 149620371 | 149621880 | 1510 | ENSG00000183111 | retained_intron | ARHGEF37 | Exon | 0.00 | up |
| chr16 | 71648873 | 71650042 | 1170 | ENSG00000040199 | nonsense_mediated_decay | PHLPP2 | 3' UTR | 0.00 | up |
| chr3 | 126024341 | 126024552 | 212 | ENSG00000114544 | retained_intron | SLC41A3 | Exon | 0.00 | up |
| chr20 | 58618810 | 58619290 | 481 | ENSG00000268941 | lncRNA | LINC01711 | Exon | 0.00 | down |
| chr9 | 37086671 | 37087181 | 511 | ENSG00000281649 | lncRNA | EBLN3P | Exon | 0.00 | down |
| chr7 | 100212738 | 100213099 | 362 | ENSG00000239521 | processed_transcript | CASTOR3 | Exon | 0.00 | down |
| chr8 | 123344948 | 123347280 | 2333 | ENSG00000283172 | miRNA | MIR548D1 | 3' UTR | 0.00 | down |
| chr8 | 27608925 | 27610673 | 1749 | ENSG00000284280 | miRNA | MIR6843 | 5' UTR | 0.00 | down |
| chr1 | 38855820 | 38866986 | 11167 | ENSG00000116954 | protein_coding | RRAGC | 3' UTR | 0.00 | up |
| chr9 | 32543805 | 32544135 | 331 | ENSG00000197579 | protein_coding | TOPORS | Exon | 0.00 | down |
| chr13 | 113842384 | 113843105 | 722 | ENSG00000233695 | lncRNA | GAS6-AS1 | Exon | 0.00 | down |
| chr4 | 56436282 | 56441946 | 5665 | ENSG00000128050 | protein_coding | PAICS | 5' UTR | 0.00 | down |
| chr1 | 52355631 | 52356517 | 887 | ENSG00000154222 | retained_intron | CC2D1B | Exon | 0.00 | down |
| chr6 | 87215127 | 87218707 | 3581 | ENSG00000188994 | processed_transcript | ZNF292 | Exon | 0.00 | up |
| chr7 | 5476844 | 5477144 | 301 | ENSG00000207973 | miRNA | MIR589 | 3' UTR | 0.00 | down |
| chr10 | 24527940 | 24531980 | 4041 | ENSG00000120549 | protein_coding | KIAA1217 | Exon | 0.00 | down |
| chr19 | 10557568 | 10559632 | 2065 | ENSG00000129347 | retained_intron | KRI1 | Exon | 0.00 | down |
| chr14 | 102084710 | 102085489 | 780 | ENSG00000080824 | retained_intron | HSP90AA1 | 3' UTR | 0.00 | up |
| chr6 | 32838020 | 32838763 | 744 | ENSG00000204267 | protein_coding | TAP2 | 5' UTR | 0.00 | up |
| chr7 | 108514555 | 108518216 | 3662 | ENSG00000135241 | protein_coding | PNPLA8 | 5' UTR | 0.00 | up |
| chr12 | 118380211 | 118402325 | 22115 | ENSG00000111707 | processed_transcript | SUDS3 | Exon | 0.00 | up |
| chr4 | 74036888 | 74037398 | 511 | ENSG00000163734 | processed_transcript | CXCL3 | 3' UTR | 0.00 | up |
| chr22 | 37723991 | 37724739 | 749 | ENSG00000100106 | protein_coding | TRIOBP | 3' UTR | 0.00 | up |
| chr1 | 150346022 | 150346292 | 271 | ENSG00000117360 | processed_transcript | PRPF3 | Exon | 0.00 | up |
| MT | 3396 | 3874 | 479 | ENSG00000198888 | protein_coding | MT-ND1 | Exon | 0.00 | up |
| chr18 | 21651229 | 21654983 | 3755 | ENSG00000158201 | protein_coding | ABHD3 | 3' UTR | 0.00 | up |
| chr1 | 31585723 | 31585992 | 270 | ENSG00000142910 | processed_transcript | TINAGL1 | Exon | 0.00 | up |
| chr17 | 64356203 | 64363216 | 7014 | ENSG00000261371 | processed_transcript | PECAM1 | Exon | 0.00 | up |
| chr15 | 30625097 | 30625714 | 618 | ENSG00000247728 | lncRNA | AC091057 | Exon | 0.00 | up |
| chr13 | 106543799 | 106557376 | 13578 | ENSG00000134884 | processed_transcript | ARGLU1 | 3' UTR | 0.00 | up |
| chr11 | 537526 | 538786 | 1261 | ENSG00000161328 | protein_coding | LRRC56 | 5' UTR | 0.00 | down |
| chr2 | 71331773 | 71332968 | 1196 | ENSG00000075292 | processed_transcript | ZNF638 | 5' UTR | 0.00 | down |
| chr1 | 222712054 | 222712412 | 359 | ENSG00000186063 | processed_transcript | AIDA | 5' UTR | 0.00 | down |
| chr5 | 149836459 | 149837060 | 602 | ENSG00000155846 | protein_coding | PPARGC1B | Exon | 0.00 | down |
| chr14 | 21403571 | 21405112 | 1542 | ENSG00000100888 | retained_intron | CHD8 | 3' UTR | 0.00 | down |
| chr12 | 132722284 | 132722435 | 152 | ENSG00000247077 | protein_coding | PGAM5 | 3' UTR | 0.00 | down |
| chr11 | 34108304 | 34112081 | 3778 | ENSG00000135372 | protein_coding | NAT10 | 5' UTR | 0.00 | up |
| chr1 | 162561684 | 162566295 | 4612 | ENSG00000117143 | processed_transcript | UAP1 | 5' UTR | 0.00 | up |
| chr12 | 122225343 | 122225764 | 422 | ENSG00000184047 | nonsense_mediated_decay | DIABLO | Exon | 0.00 | up |
| chr11 | 119310409 | 119310908 | 500 | ENSG00000076706 | processed_transcript | MCAM | Exon | 0.00 | down |
| chr4 | 5812481 | 5813558 | 1078 | ENSG00000072840 | processed_transcript | EVC | 3' UTR | 0.00 | down |
| chr12 | 119783410 | 119783831 | 422 | ENSG00000122966 | processed_transcript | CIT | Exon | 0.00 | down |
| chr1 | 207048245 | 207049681 | 1437 | ENSG00000180667 | protein_coding | YOD1 | 3' UTR | 0.00 | up |
| chr15 | 90946912 | 90947033 | 122 | ENSG00000140553 | retained_intron | UNC45A | 3' UTR | 0.00 | up |
| chr5 | 74627674 | 74627825 | 152 | ENSG00000171617 | processed_transcript | ENC1 | 3' UTR | 0.00 | up |
| chr10 | 62239589 | 62262816 | 23228 | ENSG00000182010 | protein_coding | RTKN2 | 3' UTR | 0.00 | up |
| X | 71377795 | 71378374 | 580 | ENSG00000147133 | retained_intron | TAF1 | Exon | 0.00 | up |
| chr16 | 27233074 | 27234060 | 987 | ENSG00000169189 | protein_coding | NSMCE1 | 3' UTR | 0.00 | up |
| chr16 | 71647884 | 71648574 | 691 | ENSG00000040199 | nonsense_mediated_decay | PHLPP2 | 3' UTR | 0.00 | up |
| chr17 | 7012952 | 7013251 | 300 | ENSG00000219200 | nonsense_mediated_decay | RNASEK | 5' UTR | 0.00 | up |
| chr9 | 6984354 | 6986713 | 2360 | ENSG00000107077 | processed_transcript | KDM4C | 3' UTR | 0.00 | up |
| chr6 | 34425861 | 34426041 | 181 | ENSG00000124614 | protein_coding | RPS10 | 5' UTR | 0.00 | down |
| chr12 | 108560903 | 108561173 | 271 | ENSG00000075856 | protein_coding | SART3 | 5' UTR | 0.00 | down |
| chr7 | 98207151 | 98207661 | 511 | ENSG00000180535 | protein_coding | BHLHA15 | 3' UTR | 0.00 | down |
| chr5 | 73909784 | 73911701 | 1918 | ENSG00000214944 | retained_intron | ARHGEF28 | 3' UTR | 0.00 | down |
| chr4 | 151174927 | 151176687 | 1761 | ENSG00000109686 | protein_coding | SH3D19 | 5' UTR | 0.00 | down |
| chr14 | 75003308 | 75005861 | 2554 | ENSG00000119718 | protein_coding | EIF2B2 | Exon | 0.00 | down |
| chr14 | 52857985 | 52858673 | 689 | ENSG00000073712 | retained_intron | FERMT2 | 3' UTR | 0.00 | up |
| chr15 | 41285674 | 41287393 | 1720 | ENSG00000285920 | protein_coding | AC087721 | 5' UTR | 0.00 | up |
| chr3 | 159764324 | 159764807 | 484 | ENSG00000283154 | protein_coding | IQCJ-SCHIP1 | 5' UTR | 0.00 | up |
| chr10 | 58269221 | 58276175 | 6955 | ENSG00000122873 | processed_transcript | CISD1 | 5' UTR | 0.00 | up |
| chr16 | 3115153 | 3115813 | 661 | ENSG00000122386 | protein_coding | ZNF205 | Exon | 0.00 | up |
| chr1 | 85319349 | 85319560 | 212 | ENSG00000142867 | protein_coding | BCL10 | 3' UTR | 0.00 | up |
| chr2 | 135531454 | 135565767 | 34314 | ENSG00000048991 | processed_transcript | R3HDM1 | 5' UTR | 0.00 | down |
| chr17 | 80147339 | 80147669 | 331 | ENSG00000279259 | TEC | AC087741 | Exon | 0.00 | down |
| chr12 | 14424536 | 14438249 | 13714 | ENSG00000171681 | protein_coding | ATF7IP | 5' UTR | 0.00 | down |
| chr9 | 33921692 | 33921993 | 302 | ENSG00000137073 | retained_intron | UBAP2 | 3' UTR | 0.00 | down |
| chr19 | 10224775 | 10231331 | 6557 | ENSG00000130816 | protein_coding | DNMT1 | 5' UTR | 0.00 | down |
| chr12 | 53189832 | 53190223 | 392 | ENSG00000139651 | processed_transcript | ZNF740 | 3' UTR | 0.00 | up |
| chr17 | 42666382 | 42666917 | 536 | ENSG00000037042 | retained_intron | TUBG2 | 3' UTR | 0.00 | up |
| chr16 | 11847641 | 11850285 | 2645 | ENSG00000171490 | protein_coding | RSL1D1 | 3' UTR | 0.00 | up |
| chr9 | 120903526 | 120905168 | 1643 | ENSG00000119403 | protein_coding | PHF19 | 3' UTR | 0.00 | up |
| chr4 | 112431753 | 112435190 | 3438 | ENSG00000073331 | retained_intron | ALPK1 | 3' UTR | 0.00 | up |
| chr22 | 37723992 | 37724742 | 751 | ENSG00000100106 | protein_coding | TRIOBP | 3' UTR | 0.00 | up |
| chr5 | 177618749 | 177619754 | 1006 | ENSG00000247679 | lncRNA | AC139795 | Exon | 0.00 | down |
| chr8 | 143325369 | 143327375 | 2007 | ENSG00000184428 | nonsense_mediated_decay | TOP1MT | 3' UTR | 0.00 | down |
| chr13 | 94712186 | 94712545 | 360 | ENSG00000125285 | protein_coding | SOX21 | 5' UTR | 0.00 | down |
| chr1 | 201490170 | 201490411 | 242 | ENSG00000159176 | retained_intron | CSRP1 | Exon | 0.00 | down |
| chr10 | 30340260 | 30341517 | 1258 | ENSG00000107951 | protein_coding | MTPAP | Exon | 0.00 | up |
| X | 78046314 | 78046645 | 332 | ENSG00000102144 | processed_transcript | PGK1 | 3' UTR | 0.00 | down |
| chr11 | 68755964 | 68756084 | 121 | ENSG00000132749 | protein_coding | TESMIN | 3' UTR | 0.00 | down |
| chr2 | 214728608 | 214730443 | 1836 | ENSG00000138376 | protein_coding | BARD1 | 3' UTR | 0.00 | down |
| chr14 | 76786188 | 76786575 | 388 | ENSG00000258301 | lncRNA | VASH1-AS1 | 3' UTR | 0.00 | down |
| chr16 | 3397221 | 3400976 | 3756 | ENSG00000140987 | protein_coding | ZSCAN32 | 5' UTR | 0.00 | down |
| chr15 | 90474548 | 90474849 | 302 | ENSG00000140575 | processed_transcript | IQGAP1 | Exon | 0.00 | down |
| chr1 | 231266147 | 231269931 | 3785 | ENSG00000116906 | processed_transcript | GNPAT | 3' UTR | 0.00 | down |
| chr17 | 68421409 | 68425933 | 4525 | ENSG00000207561 | miRNA | MIR635 | 3' UTR | 0.00 | up |
| chr7 | 43955121 | 43955987 | 867 | ENSG00000078967 | retained_intron | UBE2D4 | 3' UTR | 0.00 | up |
| chr10 | 124408589 | 124408963 | 375 | ENSG00000065154 | processed_transcript | OAT | 5' UTR | 0.00 | up |
| chr2 | 127706004 | 127708759 | 2756 | ENSG00000072163 | protein_coding | LIMS2 | 3' UTR | 0.00 | up |
| chr6 | 32838025 | 32838770 | 746 | ENSG00000204267 | protein_coding | TAP2 | 5' UTR | 0.00 | up |
| chr2 | 37201641 | 37211891 | 10251 | ENSG00000272054 | lncRNA | AC007390 | 3' UTR | 0.00 | up |
| chr14 | 103707545 | 103707785 | 241 | ENSG00000126215 | retained_intron | XRCC3 | Exon | 0.00 | up |
| chr7 | 139047349 | 139047620 | 272 | ENSG00000229677 | processed_pseudogene | AC018644 | 3' UTR | 0.00 | up |
| chr11 | 62832952 | 62833067 | 116 | ENSG00000133316 | protein_coding | WDR74 | Exon | 0.00 | up |
| chr15 | 30624812 | 30625710 | 899 | ENSG00000285077 | protein_coding | ARHGAP11B | Exon | 0.00 | up |
| chr16 | 89529090 | 89529420 | 331 | ENSG00000197912 | nonsense_mediated_decay | SPG7 | 3' UTR | 0.00 | down |
| chr5 | 131677677 | 131679104 | 1428 | ENSG00000286676 | processed_pseudogene | AC008695 | 3' UTR | 0.00 | down |
| chr9 | 21994807 | 22012223 | 17417 | ENSG00000147883 | protein_coding | CDKN2B | Exon | 0.00 | down |
| chr15 | 98961351 | 98962072 | 722 | ENSG00000140443 | processed_transcript | IGF1R | 3' UTR | 0.00 | down |
| chr1 | 150472885 | 150473694 | 810 | ENSG00000143374 | protein_coding | TARS2 | 3' UTR | 0.00 | down |
| chr9 | 127712148 | 127713042 | 895 | ENSG00000160401 | retained_intron | CFAP157 | 3' UTR | 0.00 | up |
| chr3 | 119393583 | 119402021 | 8439 | ENSG00000163389 | protein_coding | POGLUT1 | Exon | 0.00 | up |
| chr1 | 23695840 | 23696387 | 548 | ENSG00000142676 | retained_intron | RPL11 | 3' UTR | 0.00 | down |
| chr4 | 17801910 | 17802210 | 301 | ENSG00000163257 | processed_transcript | DCAF16 | 3' UTR | 0.00 | down |
| chr1 | 88834935 | 88835326 | 392 | ENSG00000065243 | processed_transcript | PKN2 | 3' UTR | 0.00 | down |
| chr2 | 218568579 | 218568849 | 271 | ENSG00000144580 | protein_coding | CNOT9 | 5' UTR | 0.00 | down |
| chr4 | 39302506 | 39303103 | 598 | ENSG00000035928 | retained_intron | RFC1 | Exon | 0.00 | down |
| chr18 | 8831639 | 8832179 | 541 | ENSG00000168502 | processed_transcript | MTCL1 | 3' UTR | 0.00 | down |
| chr14 | 63453684 | 63539543 | 85860 | ENSG00000154001 | protein_coding | PPP2R5E | 5' UTR | 0.00 | down |
| chr9 | 127980062 | 127980571 | 510 | ENSG00000167106 | processed_transcript | FAM102A | 5' UTR | 0.00 | up |
| chr10 | 101798099 | 101803746 | 5648 | ENSG00000198408 | processed_transcript | OGA | Exon | 0.00 | up |
| chr3 | 100377690 | 100386265 | 8576 | ENSG00000154174 | retained_intron | TOMM70 | Exon | 0.00 | up |
| X | 136046520 | 136047149 | 630 | ENSG00000198689 | retained_intron | SLC9A6 | 3' UTR | 0.00 | up |
| chr10 | 87009124 | 87009695 | 572 | ENSG00000261011 | transcribed_unprocessed_pseudogene | AL136982 | Exon | 0.00 | up |
| chr9 | 128604347 | 128605321 | 975 | ENSG00000197694 | retained_intron | SPTAN1 | Exon | 0.00 | down |
| chr20 | 63699610 | 63700273 | 664 | ENSG00000101246 | processed_transcript | ARFRP1 | 3' UTR | 0.00 | down |
| chr12 | 6669138 | 6672069 | 2932 | ENSG00000126746 | protein_coding | ZNF384 | Exon | 0.00 | down |
| chr16 | 84995931 | 85011535 | 15605 | ENSG00000153786 | protein_coding | ZDHHC7 | 5' UTR | 0.00 | down |
| chr4 | 148435123 | 148436021 | 899 | ENSG00000151623 | protein_coding | NR3C2 | Exon | 0.00 | down |
| chr17 | 38749509 | 38749809 | 301 | ENSG00000277182 | lncRNA | AC006449 | Exon | 0.00 | up |
| chr21 | 46636522 | 46643405 | 6884 | ENSG00000160310 | retained_intron | PRMT2 | 5' UTR | 0.00 | up |
| chr2 | 162310657 | 162318295 | 7639 | ENSG00000115267 | processed_transcript | IFIH1 | 3' UTR | 0.00 | up |
| chr4 | 107656944 | 107682077 | 25134 | ENSG00000138801 | retained_intron | PAPSS1 | Exon | 0.00 | down |
| chr3 | 48922462 | 48925005 | 2544 | ENSG00000177479 | retained_intron | ARIH2 | 5' UTR | 0.00 | down |
| chr8 | 144792226 | 144792377 | 152 | ENSG00000161016 | protein_coding | RPL8 | 5' UTR | 0.00 | down |
| chr1 | 161042648 | 161045977 | 3330 | ENSG00000158773 | protein_coding | USF1 | 5' UTR | 0.00 | up |
| chr13 | 40808554 | 40809919 | 1366 | ENSG00000207652 | miRNA | MIR621 | 3' UTR | 0.00 | up |
| chr17 | 78190953 | 78192770 | 1818 | ENSG00000183077 | processed_transcript | AFMID | 3' UTR | 0.00 | up |
| chr8 | 213185 | 213595 | 411 | ENSG00000223508 | transcribed_processed_pseudogene | RPL23AP53 | Exon | 0.00 | up |
| chr11 | 34882589 | 34882770 | 182 | ENSG00000149089 | nonsense_mediated_decay | APIP | 3' UTR | 0.00 | up |
| chr1 | 120548665 | 120550197 | 1533 | ENSG00000275131 | processed_transcript | AC241952 | Exon | 0.00 | up |
| chr12 | 53296486 | 53296786 | 301 | ENSG00000123349 | retained_intron | PFDN5 | 3' UTR | 0.00 | down |
| chr16 | 19430630 | 19444103 | 13474 | ENSG00000103534 | protein_coding | TMC5 | 5' UTR | 0.00 | down |
| chr9 | 120855680 | 120855921 | 242 | ENSG00000119403 | processed_transcript | PHF19 | 3' UTR | 0.00 | down |
| chr10 | 73911822 | 73911973 | 152 | ENSG00000222047 | lncRNA | C10orf55 | Exon | 0.00 | down |
| chr1 | 26531433 | 26543182 | 11750 | ENSG00000117676 | protein_coding | RPS6KA1 | 3' UTR | 0.00 | down |
| chr2 | 240523911 | 240525934 | 2024 | ENSG00000144504 | retained_intron | ANKMY1 | 3' UTR | 0.00 | up |
| chr1 | 116400280 | 116400671 | 392 | ENSG00000203865 | lncRNA | ATP1A1-AS1 | Exon | 0.00 | up |
| chr5 | 14681577 | 14687804 | 6228 | ENSG00000154124 | retained_intron | OTULIN | 3' UTR | 0.00 | up |
| chr14 | 34874461 | 34874850 | 390 | ENSG00000258738 | lncRNA | AL121603 | Exon | 0.00 | up |
| chr19 | 12579361 | 12579541 | 181 | ENSG00000269693 | nonsense_mediated_decay | AC010422 | 3' UTR | 0.00 | down |
| chr19 | 12579360 | 12579540 | 181 | ENSG00000269693 | nonsense_mediated_decay | AC010422 | 3' UTR | 0.00 | down |
| chr9 | 136010158 | 136010399 | 242 | ENSG00000148411 | processed_transcript | NACC2 | 3' UTR | 0.00 | down |
| chr14 | 76786158 | 76786578 | 421 | ENSG00000258301 | lncRNA | VASH1-AS1 | 3' UTR | 0.00 | down |
| chr12 | 98514770 | 98515220 | 451 | ENSG00000257167 | lncRNA | TMPO-AS1 | Exon | 0.00 | down |
| chr6 | 122444986 | 122447144 | 2159 | ENSG00000279453 | TEC | Z99129 | 3' UTR | 0.00 | down |
| chr2 | 222934632 | 222942129 | 7498 | ENSG00000123983 | retained_intron | ACSL3 | 3' UTR | 0.00 | up |
| chr1 | 11022209 | 11024213 | 2005 | ENSG00000120948 | nonsense_mediated_decay | TARDBP | 3' UTR | 0.00 | up |
| chr4 | 127883500 | 127890092 | 6593 | ENSG00000142731 | retained_intron | PLK4 | Exon | 0.00 | up |
| chr3 | 15247435 | 15251420 | 3986 | ENSG00000131375 | retained_intron | CAPN7 | 3' UTR | 0.00 | up |
| chr5 | 67165940 | 67166421 | 482 | ENSG00000069020 | protein_coding | MAST4 | Exon | 0.00 | down |
| chr10 | 35314670 | 35315324 | 655 | ENSG00000271335 | lncRNA | AL117336 | Exon | 0.00 | down |
| chr17 | 76727277 | 76732681 | 5405 | ENSG00000181038 | retained_intron | METTL23 | 5' UTR | 0.00 | down |
| chr3 | 49415340 | 49415550 | 211 | ENSG00000145022 | processed_transcript | TCTA | 3' UTR | 0.00 | down |
| chr1 | 26731211 | 26731541 | 331 | ENSG00000117713 | protein_coding | ARID1A | Exon | 0.00 | up |
| chr3 | 188875026 | 188875297 | 272 | ENSG00000188001 | protein_coding | TPRG1 | 3' UTR | 0.00 | up |
| chr11 | 111926423 | 111926841 | 419 | ENSG00000150764 | protein_coding | DIXDC1 | 3' UTR | 0.00 | up |
| chr11 | 111926421 | 111926841 | 421 | ENSG00000150764 | protein_coding | DIXDC1 | 3' UTR | 0.00 | up |
| chr14 | 103707531 | 103707772 | 242 | ENSG00000126214 | retained_intron | KLC1 | 3' UTR | 0.00 | up |
| chr2 | 47065935 | 47068464 | 2530 | ENSG00000225187 | lncRNA | AC073283 | 3' UTR | 0.00 | down |
| chr3 | 28323495 | 28324574 | 1080 | ENSG00000187118 | processed_transcript | CMC1 | 3' UTR | 0.00 | down |
| chr2 | 190969706 | 190969977 | 272 | ENSG00000115415 | processed_transcript | STAT1 | 3' UTR | 0.00 | down |
| chr19 | 1985437 | 1985558 | 122 | ENSG00000133243 | retained_intron | BTBD2 | 3' UTR | 0.00 | down |
| chr17 | 19330652 | 19334496 | 3845 | ENSG00000072134 | retained_intron | EPN2 | 3' UTR | 0.00 | up |
| chr17 | 68421383 | 68421833 | 451 | ENSG00000267009 | lncRNA | AC007780 | 3' UTR | 0.00 | up |
| chr15 | 40036401 | 40036826 | 426 | ENSG00000140319 | processed_transcript | SRP14 | 3' UTR | 0.00 | up |
| chr1 | 233905726 | 233905931 | 206 | ENSG00000287921 | lncRNA | AL713868 | Exon | 0.00 | up |
| chr17 | 81978304 | 81978755 | 452 | ENSG00000169696 | nonsense_mediated_decay | ASPSCR1 | 5' UTR | 0.00 | up |
| chr20 | 23356651 | 23356855 | 205 | ENSG00000232645 | lncRNA | LINC01431 | Exon | 0.00 | down |
| chr5 | 664404 | 664704 | 301 | ENSG00000171368 | protein_coding | TPPP | 3' UTR | 0.00 | down |
| chr6 | 139166382 | 139174399 | 8018 | ENSG00000112406 | protein_coding | HECA | Exon | 0.00 | up |
| chr11 | 125102371 | 125102582 | 212 | ENSG00000150433 | protein_coding | TMEM218 | 5' UTR | 0.00 | up |
| chr3 | 40311470 | 40311681 | 212 | ENSG00000114784 | retained_intron | EIF1B | Exon | 0.00 | up |
| chr1 | 233905732 | 234214455 | 308724 | ENSG00000183780 | protein_coding | SLC35F3 | 5' UTR | 0.00 | up |
| chr1 | 40257226 | 40257760 | 535 | ENSG00000259943 | lncRNA | AL050341 | Exon | 0.00 | down |
| chr12 | 111744972 | 111745453 | 482 | ENSG00000111271 | retained_intron | ACAD10 | 3' UTR | 0.00 | down |
| chr1 | 3829275 | 3829863 | 589 | ENSG00000116198 | retained_intron | CEP104 | 3' UTR | 0.00 | up |
| chr11 | 68929837 | 68932398 | 2562 | ENSG00000132740 | processed_transcript | IGHMBP2 | Exon | 0.00 | up |
| chr10 | 3778780 | 3779110 | 331 | ENSG00000067082 | processed_transcript | KLF6 | 3' UTR | 0.00 | up |
| chr17 | 40140646 | 40140978 | 333 | ENSG00000108349 | protein_coding | CASC3 | 5' UTR | 0.00 | up |
| chr5 | 69282604 | 69294837 | 12234 | ENSG00000213830 | processed_pseudogene | CFL1P5 | 3' UTR | 0.00 | up |
| chr11 | 83087970 | 83092241 | 4272 | ENSG00000279900 | TEC | AP001767 | Exon | 0.00 | down |
| chr2 | 112483242 | 112485918 | 2677 | ENSG00000114999 | processed_transcript | TTL | Exon | 0.00 | down |
| chr1 | 151364333 | 151364574 | 242 | ENSG00000143416 | retained_intron | SELENBP1 | 3' UTR | 0.00 | down |
| chr19 | 12675682 | 12675832 | 151 | ENSG00000123154 | retained_intron | WDR83 | 3' UTR | 0.00 | down |
| chr11 | 66363342 | 66363995 | 654 | ENSG00000255468 | lncRNA | AP001107 | Exon | 0.00 | down |
| chr21 | 41739758 | 41739998 | 241 | ENSG00000275166 | miRNA | MIR6814 | 3' UTR | 0.00 | down |
| chr1 | 155749721 | 155751865 | 2145 | ENSG00000116580 | retained_intron | GON4L | 3' UTR | 0.00 | up |
| chr17 | 40475827 | 40476067 | 241 | ENSG00000279806 | TEC | AC018629 | 3' UTR | 0.00 | up |
| chr1 | 114707839 | 114713952 | 6114 | ENSG00000213281 | protein_coding | NRAS | 3' UTR | 0.00 | up |
| chr10 | 67849971 | 67850359 | 389 | ENSG00000272892 | lncRNA | AL133551 | Exon | 0.00 | down |
| chr22 | 50529281 | 50529852 | 572 | ENSG00000025708 | nonsense_mediated_decay | TYMP | 5' UTR | 0.00 | down |
| chr1 | 109313726 | 109323102 | 9377 | ENSG00000134243 | processed_transcript | SORT1 | 3' UTR | 0.00 | down |
| chr1 | 41142905 | 41152650 | 9746 | ENSG00000010803 | protein_coding | SCMH1 | 5' UTR | 0.00 | down |
| chr2 | 58088362 | 58131857 | 43496 | ENSG00000028116 | protein_coding | VRK2 | 3' UTR | 0.00 | up |
| chr8 | 98046108 | 98046469 | 362 | ENSG00000156482 | protein_coding | RPL30 | 5' UTR | 0.00 | up |
| chr15 | 89645383 | 89645945 | 563 | ENSG00000166813 | nonsense_mediated_decay | KIF7 | Exon | 0.00 | up |
| chr7 | 130293133 | 130293404 | 272 | ENSG00000128510 | protein_coding | CPA4 | 5' UTR | 0.00 | up |
| chr15 | 99427008 | 99430972 | 3965 | ENSG00000259341 | lncRNA | AC015660 | Exon | 0.00 | up |
| chr20 | 25229239 | 25229689 | 451 | ENSG00000277938 | lncRNA | AL035252 | Exon | 0.00 | down |
| X | 153349600 | 153350289 | 690 | ENSG00000063587 | processed_transcript | ZNF275 | 3' UTR | 0.00 | down |
| chr11 | 4126767 | 4129147 | 2381 | ENSG00000167325 | retained_intron | RRM1 | 3' UTR | 0.00 | up |
| chr19 | 52849120 | 52849301 | 182 | ENSG00000204604 | protein_coding | ZNF468 | 3' UTR | 0.00 | down |
| chr16 | 69167056 | 69167326 | 271 | ENSG00000141076 | processed_transcript | UTP4 | 3' UTR | 0.00 | up |
| chr5 | 141640737 | 141641064 | 328 | ENSG00000164620 | protein_coding | RELL2 | 3' UTR | 0.00 | up |
| chr2 | 197500408 | 197500558 | 151 | ENSG00000115541 | retained_intron | HSPE1 | 5' UTR | 0.00 | up |
| chr5 | 10649302 | 10649992 | 691 | ENSG00000164236 | nonsense_mediated_decay | ANKRD33B | 3' UTR | 0.00 | up |
| chr5 | 58458096 | 58458306 | 211 | ENSG00000145632 | retained_intron | PLK2 | Exon | 0.00 | up |
| chr15 | 40161202 | 40170593 | 9392 | ENSG00000156970 | protein_coding | BUB1B | 3' UTR | 0.00 | down |
| chr13 | 25339993 | 25340324 | 332 | ENSG00000139496 | processed_transcript | NUP58 | 3' UTR | 0.00 | down |
| chr19 | 16089015 | 16089286 | 272 | ENSG00000167460 | processed_transcript | TPM4 | 3' UTR | 0.00 | down |
| chr1 | 223770440 | 223770718 | 279 | ENSG00000162909 | processed_transcript | CAPN2 | Exon | 0.00 | down |
| chr9 | 95086116 | 95087188 | 1073 | ENSG00000284459 | miRNA | MIR24-1 | 3' UTR | 0.00 | down |
| chr16 | 1315631 | 1315872 | 242 | ENSG00000103275 | retained_intron | UBE2I | Exon | 0.00 | up |
| chr15 | 75578745 | 75579045 | 301 | ENSG00000169410 | protein_coding | PTPN9 | 5' UTR | 0.00 | up |
| chr19 | 11805990 | 11806950 | 961 | ENSG00000171295 | protein_coding | ZNF440 | Exon | 0.00 | up |
| chr1 | 11107343 | 11111598 | 4256 | ENSG00000198793 | processed_transcript | MTOR | 3' UTR | 0.00 | down |
| chr2 | 189771492 | 189774752 | 3261 | ENSG00000128699 | processed_transcript | ORMDL1 | 3' UTR | 0.00 | up |
| chr10 | 68981560 | 68983151 | 1592 | ENSG00000198954 | retained_intron | KIFBP | 3' UTR | 0.00 | up |
| chr3 | 101574209 | 101589871 | 15663 | ENSG00000081154 | processed_transcript | PCNP | 3' UTR | 0.00 | down |
| chr15 | 68779791 | 68780628 | 838 | ENSG00000140350 | retained_intron | ANP32A | 3' UTR | 0.00 | up |
| chr2 | 74047843 | 74048263 | 421 | ENSG00000187605 | retained_intron | TET3 | Exon | 0.00 | up |
| chr7 | 148698953 | 148730240 | 31288 | ENSG00000055130 | protein_coding | CUL1 | 5' UTR | 0.00 | up |
| chr1 | 97721574 | 97855062 | 133489 | ENSG00000188641 | retained_intron | DPYD | Exon | 0.00 | up |
| chr4 | 127830675 | 127832978 | 2304 | ENSG00000164070 | processed_transcript | HSPA4L | 3' UTR | 0.00 | up |
| chr19 | 510331 | 519415 | 9085 | ENSG00000141933 | retained_intron | TPGS1 | Exon | 0.00 | down |
| chr1 | 112700565 | 112700746 | 182 | ENSG00000155363 | processed_transcript | MOV10 | 3' UTR | 0.00 | up |
| chr14 | 93185031 | 93209860 | 24830 | ENSG00000012963 | protein_coding | UBR7 | 3' UTR | 0.00 | up |
| chr9 | 83643505 | 83643892 | 388 | ENSG00000148057 | protein_coding | IDNK | 3' UTR | 0.00 | down |
| chr1 | 160997956 | 160998403 | 448 | ENSG00000158769 | processed_transcript | F11R | 3' UTR | 0.00 | down |
| chr3 | 183803961 | 183809326 | 5366 | ENSG00000163872 | retained_intron | YEATS2 | Exon | 0.00 | down |
| chr11 | 78654835 | 78655345 | 511 | ENSG00000149256 | protein_coding | TENM4 | 3' UTR | 0.00 | up |
| chr14 | 61452854 | 61456994 | 4141 | ENSG00000027075 | processed_transcript | PRKCH | Exon | 0.00 | up |
| chr1 | 10653641 | 10654442 | 802 | ENSG00000130940 | retained_intron | CASZ1 | Exon | 0.00 | up |
| chr5 | 141409275 | 141409634 | 360 | ENSG00000253305 | protein_coding | PCDHGB6 | Exon | 0.00 | down |
| chr3 | 49720139 | 49720319 | 181 | ENSG00000176020 | protein_coding | AMIGO3 | 3' UTR | 0.00 | down |
| chr10 | 132650427 | 132697884 | 47458 | ENSG00000068383 | processed_transcript | INPP5A | Exon | 0.00 | down |
| chr14 | 23063016 | 23063197 | 182 | ENSG00000100813 | retained_intron | ACIN1 | 3' UTR | 0.00 | up |
| chr5 | 70109721 | 70110228 | 508 | ENSG00000179978 | unprocessed_pseudogene | NAIPP2 | Exon | 0.00 | down |
| chr2 | 95180246 | 95182707 | 2462 | ENSG00000275111 | protein_coding | ZNF2 | 3' UTR | 0.00 | down |
| chr11 | 32103347 | 32104828 | 1482 | ENSG00000049449 | nonsense_mediated_decay | RCN1 | 3' UTR | 0.00 | down |
| chr11 | 8912516 | 8917553 | 5038 | ENSG00000166452 | protein_coding | AKIP1 | 3' UTR | 0.00 | down |
| chr8 | 126556322 | 126557373 | 1052 | ENSG00000168672 | processed_transcript | LRATD2 | Exon | 0.00 | down |
| chr19 | 13116773 | 13117102 | 330 | ENSG00000160877 | protein_coding | NACC1 | 5' UTR | 0.00 | up |
| chr17 | 28384863 | 28386628 | 1766 | ENSG00000004139 | protein_coding | SARM1 | 3' UTR | 0.00 | up |
| chr12 | 112160320 | 112161063 | 744 | ENSG00000173064 | processed_transcript | HECTD4 | 3' UTR | 0.00 | up |
| chr7 | 93105528 | 93117903 | 12376 | ENSG00000205413 | protein_coding | SAMD9 | 5' UTR | 0.00 | up |
| chr17 | 44802221 | 44802581 | 361 | ENSG00000182963 | protein_coding | GJC1 | 3' UTR | 0.00 | down |
| chr5 | 141489917 | 141491594 | 1678 | ENSG00000240764 | protein_coding | PCDHGC5 | Exon | 0.00 | down |
| chr8 | 81480442 | 81483117 | 2676 | ENSG00000170323 | nonsense_mediated_decay | FABP4 | 3' UTR | 0.00 | up |
| chr5 | 457954 | 459498 | 1545 | ENSG00000180104 | retained_intron | EXOC3 | Exon | 0.00 | up |
| chr1 | 120464411 | 120466571 | 2161 | ENSG00000270231 | transcribed_unprocessed_pseudogene | NBPF8 | Exon | 0.00 | up |
| chr19 | 18905819 | 18906179 | 361 | ENSG00000105669 | processed_transcript | COPE | Exon | 0.00 | up |
| chr1 | 150218744 | 150219072 | 329 | ENSG00000143401 | protein_coding | ANP32E | 3' UTR | 0.00 | up |
| chr18 | 26170960 | 26171137 | 178 | ENSG00000188985 | processed_pseudogene | DHFRP1 | Exon | 0.00 | down |
| chr12 | 112509257 | 112509558 | 302 | ENSG00000179295 | protein_coding | PTPN11 | 3' UTR | 0.00 | down |
| chr15 | 88459476 | 88459956 | 481 | ENSG00000259494 | nonsense_mediated_decay | MRPL46 | 3' UTR | 0.00 | down |
| chr13 | 106491961 | 106535063 | 43103 | ENSG00000125266 | processed_transcript | EFNB2 | 3' UTR | 0.00 | up |
| X | 136875382 | 136879114 | 3733 | ENSG00000206979 | snoRNA | SNORD61 | 3' UTR | 0.00 | up |
| chr11 | 68050744 | 68050895 | 152 | ENSG00000255031 | lncRNA | AP002807 | 3' UTR | 0.00 | up |
| chr5 | 38883941 | 38886570 | 2630 | ENSG00000145623 | protein_coding | OSMR | 3' UTR | 0.00 | up |
| chr7 | 1057817 | 1059202 | 1386 | ENSG00000257607 | lncRNA | AC073957 | Exon | 0.00 | up |
| chr15 | 43406566 | 43406836 | 271 | ENSG00000137822 | nonsense_mediated_decay | TUBGCP4 | 3' UTR | 0.00 | up |
| chr15 | 43406566 | 43406837 | 272 | ENSG00000067369 | protein_coding | TP53BP1 | 3' UTR | 0.00 | up |
| chr11 | 32856651 | 32857012 | 362 | ENSG00000135378 | protein_coding | PRRG4 | 3' UTR | 0.00 | down |
| chr16 | 31083438 | 31084656 | 1219 | ENSG00000151006 | retained_intron | PRSS53 | 3' UTR | 0.00 | down |
| chr15 | 43800580 | 43801317 | 738 | ENSG00000242028 | protein_coding | HYPK | 3' UTR | 0.00 | down |
| chr19 | 47495043 | 47495547 | 505 | ENSG00000268061 | lncRNA | NAPA-AS1 | Exon | 0.00 | down |
| chr2 | 174123202 | 174217906 | 94705 | ENSG00000138430 | processed_transcript | OLA1 | 5' UTR | 0.00 | up |
| chr10 | 104025613 | 104026182 | 570 | ENSG00000065613 | protein_coding | SLK | 3' UTR | 0.00 | up |
| chr16 | 4614729 | 4614937 | 209 | ENSG00000153443 | protein_coding | UBALD1 | 5' UTR | 0.00 | up |
| chr16 | 89151997 | 89153287 | 1291 | ENSG00000176715 | processed_transcript | ACSF3 | Exon | 0.00 | down |
| chr2 | 63933815 | 63975025 | 41211 | ENSG00000143952 | protein_coding | VPS54 | 5' UTR | 0.00 | up |
| chr7 | 17344625 | 17344775 | 151 | ENSG00000106546 | processed_transcript | AHR | 3' UTR | 0.00 | down |
| chr19 | 12079678 | 12080547 | 870 | ENSG00000286132 | nonsense_mediated_decay | AC022415 | 3' UTR | 0.00 | down |
| chr13 | 73813272 | 73995056 | 181785 | ENSG00000118922 | protein_coding | KLF12 | 5' UTR | 0.00 | down |
| chr2 | 98599583 | 98602399 | 2817 | ENSG00000183513 | retained_intron | COA5 | 3' UTR | 0.00 | down |
| chr14 | 20684559 | 20688792 | 4234 | ENSG00000214274 | processed_transcript | ANG | 5' UTR | 0.00 | up |
| chr9 | 127612262 | 127613447 | 1186 | ENSG00000136854 | nonsense_mediated_decay | STXBP1 | 3' UTR | 0.00 | up |
| chr14 | 81221484 | 81222014 | 531 | ENSG00000273783 | lncRNA | AL136040 | Exon | 0.00 | up |
| chr16 | 11312858 | 11345578 | 32721 | ENSG00000175643 | processed_transcript | RMI2 | 5' UTR | 0.00 | up |
| chr16 | 30894568 | 30895220 | 653 | ENSG00000099385 | protein_coding | BCL7C | Exon | 0.00 | up |
| chr6 | 7284066 | 7284516 | 451 | ENSG00000124783 | processed_transcript | SSR1 | 3' UTR | 0.00 | down |
| chr6 | 34425884 | 34426069 | 186 | ENSG00000124614 | protein_coding | RPS10 | 5' UTR | 0.00 | down |
| chr2 | 85561621 | 85579823 | 18203 | ENSG00000118640 | protein_coding | VAMP8 | 5' UTR | 0.00 | down |
| chr15 | 44561333 | 44562078 | 746 | ENSG00000259659 | lncRNA | AC009996 | 3' UTR | 0.00 | up |
| chr3 | 50583993 | 50585404 | 1412 | ENSG00000114735 | protein_coding | HEMK1 | 3' UTR | 0.00 | up |
| chr1 | 1071114 | 1072065 | 952 | ENSG00000237330 | protein_coding | RNF223 | 3' UTR | 0.00 | up |
| chr12 | 112247489 | 112249588 | 2100 | ENSG00000173064 | retained_intron | HECTD4 | Exon | 0.00 | up |
| chr1 | 207079018 | 207079558 | 541 | ENSG00000123836 | processed_transcript | PFKFB2 | 3' UTR | 0.00 | up |
| chr9 | 100346693 | 100352533 | 5841 | ENSG00000136891 | protein_coding | TEX10 | 5' UTR | 0.00 | down |
| chr19 | 5708013 | 5708563 | 551 | ENSG00000196365 | retained_intron | LONP1 | 3' UTR | 0.00 | down |
| chr2 | 101007616 | 101011185 | 3570 | ENSG00000223947 | lncRNA | AC016738 | 3' UTR | 0.00 | up |
| chr12 | 112160307 | 112161118 | 812 | ENSG00000173064 | processed_transcript | HECTD4 | 3' UTR | 0.00 | up |
| chr4 | 7062441 | 7064126 | 1686 | ENSG00000109519 | retained_intron | GRPEL1 | Exon | 0.00 | up |
| chr1 | 155478845 | 155479295 | 451 | ENSG00000227773 | lncRNA | ASH1L-IT1 | Exon | 0.00 | up |
| chr14 | 19075098 | 19103688 | 28591 | ENSG00000225210 | transcribed_processed_pseudogene | DUXAP9 | Exon | 0.00 | up |
| chr9 | 21994822 | 21995301 | 480 | ENSG00000147889 | protein_coding | CDKN2A | 5' UTR | 0.00 | down |
| chr1 | 113973909 | 113974270 | 362 | ENSG00000116774 | protein_coding | OLFML3 | 3' UTR | 0.00 | down |
| chr17 | 28396087 | 28396477 | 391 | ENSG00000004139 | protein_coding | SARM1 | 3' UTR | 0.00 | down |
| chr3 | 155914463 | 155922279 | 7817 | ENSG00000163655 | processed_transcript | GMPS | Exon | 0.00 | down |
| chr3 | 70958724 | 70959295 | 572 | ENSG00000114861 | retained_intron | FOXP1 | 3' UTR | 0.00 | down |
| chr14 | 99398077 | 99404244 | 6168 | ENSG00000183576 | retained_intron | SETD3 | 3' UTR | 0.00 | down |
| chr6 | 30652792 | 30653210 | 419 | ENSG00000204564 | retained_intron | C6orf136 | 3' UTR | 0.00 | up |
| chr19 | 53576730 | 53578350 | 1621 | ENSG00000130844 | protein_coding | ZNF331 | 3' UTR | 0.00 | up |
| chr6 | 143948072 | 143960590 | 12519 | ENSG00000118495 | protein_coding | PLAGL1 | 5' UTR | 0.00 | up |
| chr14 | 72731808 | 72742556 | 10749 | ENSG00000205683 | processed_transcript | DPF3 | Exon | 0.00 | up |
| chr15 | 52019266 | 52021512 | 2247 | ENSG00000069956 | processed_transcript | MAPK6 | 5' UTR | 0.00 | up |
| chr2 | 32607574 | 32618090 | 10517 | ENSG00000115760 | retained_intron | BIRC6 | 3' UTR | 0.00 | down |
| chr2 | 241494108 | 241494529 | 422 | ENSG00000115694 | processed_transcript | STK25 | 3' UTR | 0.00 | down |
| chr10 | 95627524 | 95628395 | 872 | ENSG00000059573 | processed_transcript | ALDH18A1 | Exon | 0.00 | down |
| chr3 | 50350616 | 50350796 | 181 | ENSG00000114388 | protein_coding | NPRL2 | 5' UTR | 0.00 | down |
| chr1 | 26244246 | 26254745 | 10500 | ENSG00000130695 | processed_transcript | CEP85 | Exon | 0.00 | down |
| chr3 | 33142733 | 33142794 | 62 | ENSG00000170275 | processed_transcript | CRTAP | 3' UTR | 0.00 | down |
| chr10 | 101000962 | 101001412 | 451 | ENSG00000107816 | protein_coding | LZTS2 | 5' UTR | 0.00 | up |
| chr12 | 107710458 | 107712476 | 2019 | ENSG00000240441 | processed_pseudogene | AC007622 | 3' UTR | 0.00 | up |
| chr2 | 96833558 | 96833858 | 301 | ENSG00000168763 | processed_transcript | CNNM3 | 3' UTR | 0.00 | up |
| chr18 | 62945135 | 62958643 | 13509 | ENSG00000081913 | processed_transcript | PHLPP1 | Exon | 0.00 | up |
| chr12 | 8932798 | 8932949 | 152 | ENSG00000111752 | nonsense_mediated_decay | PHC1 | 3' UTR | 0.00 | down |
| chr6 | 15496974 | 15501159 | 4186 | ENSG00000008083 | processed_transcript | JARID2 | Exon | 0.00 | down |
| chr1 | 222654245 | 222659965 | 5721 | ENSG00000154305 | processed_transcript | MIA3 | Exon | 0.00 | down |
| X | 150470921 | 150471312 | 392 | ENSG00000013619 | protein_coding | MAMLD1 | Exon | 0.00 | down |
| chr2 | 121338464 | 121338855 | 392 | ENSG00000074054 | retained_intron | CLASP1 | 3' UTR | 0.00 | down |
| chr17 | 43877843 | 43879345 | 1503 | ENSG00000108852 | protein_coding | MPP2 | Exon | 0.00 | up |
| chr17 | 82442256 | 82442645 | 390 | ENSG00000169660 | retained_intron | HEXD | 3' UTR | 0.00 | up |
| chr16 | 29995116 | 29995326 | 211 | ENSG00000149929 | retained_intron | HIRIP3 | Exon | 0.00 | up |
| chr10 | 117281017 | 117281407 | 391 | ENSG00000277879 | lncRNA | AL391988 | 3' UTR | 0.00 | down |
| chr12 | 108560896 | 108561193 | 298 | ENSG00000075856 | protein_coding | SART3 | 5' UTR | 0.00 | down |
| chr2 | 241494151 | 241494542 | 392 | ENSG00000006607 | protein_coding | FARP2 | 3' UTR | 0.00 | down |
| chr16 | 15625086 | 15625765 | 680 | ENSG00000166783 | retained_intron | MARF1 | Exon | 0.00 | down |
| chr6 | 28276817 | 28278074 | 1258 | ENSG00000137338 | protein_coding | PGBD1 | 3' UTR | 0.00 | down |
| chr5 | 9041710 | 9042998 | 1289 | ENSG00000266415 | miRNA | MIR4636 | 3' UTR | 0.00 | down |
| chr8 | 98035706 | 98036126 | 421 | ENSG00000132561 | protein_coding | MATN2 | 3' UTR | 0.00 | up |
| chr15 | 43795912 | 43798932 | 3021 | ENSG00000242028 | protein_coding | HYPK | 3' UTR | 0.00 | up |
| chr1 | 113114442 | 113116333 | 1892 | ENSG00000198799 | processed_transcript | LRIG2 | Exon | 0.00 | down |
| X | 153790845 | 153791215 | 371 | ENSG00000067829 | protein_coding | IDH3G | 5' UTR | 0.00 | up |
| chr9 | 133030704 | 133031064 | 361 | ENSG00000148308 | protein_coding | GTF3C5 | 5' UTR | 0.00 | up |
| chr15 | 74640587 | 74640738 | 152 | ENSG00000179335 | retained_intron | CLK3 | 3' UTR | 0.00 | up |
| chr9 | 98082158 | 98083077 | 920 | ENSG00000095380 | protein_coding | NANS | 3' UTR | 0.00 | up |
| chr3 | 44917295 | 44918136 | 842 | ENSG00000163812 | processed_transcript | ZDHHC3 | 3' UTR | 0.00 | down |
| chr8 | 37493450 | 37493868 | 419 | ENSG00000253161 | lncRNA | LINC01605 | Exon | 0.00 | down |
| chr18 | 30993438 | 30996982 | 3545 | ENSG00000134762 | protein_coding | DSC3 | 3' UTR | 0.00 | down |
| chr22 | 50199089 | 50200513 | 1425 | ENSG00000273253 | lncRNA | AL022328 | Exon | 0.00 | up |
| chr20 | 54215315 | 54219366 | 4052 | ENSG00000101132 | processed_transcript | PFDN4 | 3' UTR | 0.00 | up |
| chr21 | 45974546 | 45974750 | 205 | ENSG00000274248 | lncRNA | AJ011932 | Exon | 0.00 | up |
| chr16 | 67933279 | 67933759 | 481 | ENSG00000141086 | retained_intron | CTRL | Exon | 0.00 | down |
| chr5 | 150709606 | 150709786 | 181 | ENSG00000086589 | retained_intron | RBM22 | 3' UTR | 0.00 | down |
| chr4 | 1693059 | 1693359 | 301 | ENSG00000174137 | protein_coding | FAM53A | 3' UTR | 0.00 | up |
| chr14 | 22976769 | 22977159 | 391 | ENSG00000129474 | protein_coding | AJUBA | 5' UTR | 0.00 | up |
| chr4 | 169697257 | 169704090 | 6834 | ENSG00000109572 | protein_coding | CLCN3 | Exon | 0.00 | up |
| chr21 | 44460607 | 44461027 | 421 | ENSG00000160233 | protein_coding | LRRC3 | 3' UTR | 0.00 | down |
| chr2 | 195680037 | 195683884 | 3848 | ENSG00000196950 | retained_intron | SLC39A10 | 3' UTR | 0.00 | down |
| chr16 | 75093589 | 75095712 | 2124 | ENSG00000186187 | processed_transcript | ZNRF1 | 5' UTR | 0.00 | down |
| chr14 | 20990241 | 20990421 | 181 | ENSG00000165792 | retained_intron | METTL17 | 5' UTR | 0.00 | down |
| chr12 | 110434882 | 110436344 | 1463 | ENSG00000111229 | retained_intron | ARPC3 | 3' UTR | 0.00 | down |
| chr3 | 155833778 | 155844837 | 11060 | ENSG00000169359 | protein_coding | SLC33A1 | 3' UTR | 0.00 | down |
| chr8 | 144289262 | 144291438 | 2177 | ENSG00000261236 | protein_coding | BOP1 | 5' UTR | 0.00 | up |
| chr14 | 21498217 | 21498577 | 361 | ENSG00000092203 | retained_intron | TOX4 | 3' UTR | 0.00 | up |
| chr6 | 28283766 | 28284124 | 359 | ENSG00000137338 | protein_coding | PGBD1 | 5' UTR | 0.00 | up |
| chr2 | 130917166 | 130917377 | 212 | ENSG00000136002 | protein_coding | ARHGEF4 | Exon | 0.00 | down |
| chr11 | 10778864 | 10779313 | 450 | ENSG00000198730 | nonsense_mediated_decay | CTR9 | 3' UTR | 0.00 | down |
| X | 154409422 | 154409932 | 511 | ENSG00000013563 | protein_coding | DNASE1L1 | 5' UTR | 0.00 | down |
| chr16 | 668104 | 668584 | 481 | ENSG00000140983 | retained_intron | RHOT2 | 5' UTR | 0.00 | down |
| chr1 | 90893257 | 90893435 | 179 | ENSG00000232882 | processed_pseudogene | PHKA1P1 | Exon | 0.00 | up |
| chr17 | 44781576 | 44781846 | 271 | ENSG00000073670 | retained_intron | ADAM11 | 3' UTR | 0.00 | down |
| chr4 | 56483171 | 56486389 | 3219 | ENSG00000174780 | retained_intron | SRP72 | Exon | 0.00 | down |
| chr20 | 62894434 | 62896665 | 2232 | ENSG00000101191 | protein_coding | DIDO1 | Exon | 0.00 | down |
| chr2 | 95828673 | 95829603 | 931 | ENSG00000232931 | lncRNA | LINC00342 | Exon | 0.00 | down |
| chr14 | 64486748 | 64490461 | 3714 | ENSG00000089775 | protein_coding | ZBTB25 | 3' UTR | 0.00 | up |
| chr2 | 20308523 | 20312244 | 3722 | ENSG00000055917 | protein_coding | PUM2 | 3' UTR | 0.00 | up |
| chr18 | 51058204 | 51058450 | 247 | ENSG00000141646 | nonsense_mediated_decay | SMAD4 | Exon | 0.00 | up |
| chr16 | 739646 | 739827 | 182 | ENSG00000103245 | protein_coding | CIAO3 | 3' UTR | 0.00 | up |
| chr20 | 46067111 | 46079450 | 12340 | ENSG00000124160 | protein_coding | NCOA5 | 5' UTR | 0.00 | down |
| chr8 | 29356476 | 29358666 | 2191 | ENSG00000285601 | lncRNA | AC084262 | Exon | 0.00 | down |
| chr3 | 12312390 | 12351501 | 39112 | ENSG00000132170 | retained_intron | PPARG | 5' UTR | 0.00 | down |
| chr21 | 37202632 | 37203112 | 481 | ENSG00000230366 | lncRNA | DSCR9 | 3' UTR | 0.00 | down |
| chr3 | 9933032 | 9933182 | 151 | ENSG00000288550 | nonsense_mediated_decay | AC018809 | 3' UTR | 0.00 | up |
| chr14 | 20684586 | 20684737 | 152 | ENSG00000214274 | processed_transcript | ANG | 5' UTR | 0.00 | up |
| chr17 | 12095590 | 12110599 | 15010 | ENSG00000065559 | retained_intron | MAP2K4 | 3' UTR | 0.00 | up |
| chr5 | 56911178 | 56911536 | 359 | ENSG00000155542 | TEC | SETD9 | Exon | 0.00 | up |
| chr11 | 64907644 | 64909008 | 1365 | ENSG00000110046 | protein_coding | ATG2A | Exon | 0.00 | down |
| chr12 | 68746334 | 68746725 | 392 | ENSG00000175782 | protein_coding | SLC35E3 | 5' UTR | 0.00 | down |
| chr19 | 12576937 | 12578912 | 1976 | ENSG00000269693 | nonsense_mediated_decay | AC010422 | 3' UTR | 0.00 | down |
| chr4 | 3518054 | 3518913 | 860 | ENSG00000163956 | processed_transcript | LRPAP1 | 3' UTR | 0.00 | up |
| chr15 | 85208756 | 85209056 | 301 | ENSG00000259295 | processed_transcript | CSPG4P12 | Exon | 0.00 | up |
| chr9 | 41076007 | 41076333 | 327 | ENSG00000225655 | lncRNA | BX255923 | Exon | 0.00 | down |
| chr2 | 10130703 | 10130883 | 181 | ENSG00000171848 | nonsense_mediated_decay | RRM2 | 3' UTR | 0.00 | down |
| chr6 | 31139672 | 31140002 | 331 | ENSG00000238211 | processed_pseudogene | POLR2LP1 | 3' UTR | 0.00 | down |
| chr2 | 38666080 | 38675998 | 9919 | ENSG00000143891 | processed_transcript | GALM | 5' UTR | 0.00 | down |
| chr2 | 215346857 | 215347126 | 270 | ENSG00000138363 | processed_transcript | ATIC | 3' UTR | 0.00 | down |
| chr17 | 2380338 | 2380489 | 152 | ENSG00000141258 | protein_coding | SGSM2 | 3' UTR | 0.00 | up |
| chr11 | 119372953 | 119373373 | 421 | ENSG00000036672 | protein_coding | USP2 | Exon | 0.00 | down |
| chr13 | 52664038 | 52680018 | 15981 | ENSG00000165416 | retained_intron | SUGT1 | Exon | 0.00 | down |
| chr14 | 23047061 | 23047272 | 212 | ENSG00000139880 | processed_transcript | CDH24 | 3' UTR | 0.00 | down |
| chr9 | 16704790 | 16727835 | 23046 | ENSG00000173068 | protein_coding | BNC2 | 5' UTR | 0.00 | down |
| chr15 | 41302067 | 41302547 | 481 | ENSG00000285920 | protein_coding | AC087721 | Exon | 0.00 | down |
| chr14 | 44934178 | 44959350 | 25173 | ENSG00000179454 | protein_coding | KLHL28 | 5' UTR | 0.00 | down |
| chr19 | 7467304 | 7467544 | 241 | ENSG00000104880 | protein_coding | ARHGEF18 | Exon | 0.00 | down |
| chr17 | 44035495 | 44035706 | 212 | ENSG00000161654 | retained_intron | LSM12 | 3' UTR | 0.00 | up |
| chr10 | 77975510 | 77975840 | 331 | ENSG00000148606 | protein_coding | POLR3A | 3' UTR | 0.00 | up |
| chr18 | 23909196 | 23914499 | 5304 | ENSG00000053747 | retained_intron | LAMA3 | Exon | 0.00 | up |
| chr16 | 67210174 | 67210503 | 330 | ENSG00000280163 | TEC | AC040160 | 5' UTR | 0.00 | up |
| chr10 | 119872655 | 119872836 | 182 | ENSG00000197771 | protein_coding | MCMBP | 5' UTR | 0.00 | up |
| chr17 | 34961867 | 34962524 | 658 | ENSG00000198783 | nonsense_mediated_decay | ZNF830 | Exon | 0.00 | down |
| chr21 | 36247551 | 36253803 | 6253 | ENSG00000142197 | retained_intron | DOP1B | Exon | 0.00 | up |
| chr20 | 63627374 | 63628824 | 1451 | ENSG00000232442 | lncRNA | MHENCR | Exon | 0.00 | up |
| chr10 | 133398465 | 133399629 | 1165 | ENSG00000148824 | retained_intron | MTG1 | 3' UTR | 0.00 | up |
| chr10 | 133398464 | 133399631 | 1168 | ENSG00000148824 | retained_intron | MTG1 | 3' UTR | 0.00 | up |
| chr16 | 57186961 | 57205224 | 18264 | ENSG00000159579 | protein_coding | RSPRY1 | 5' UTR | 0.00 | up |
| chr6 | 155311226 | 155313204 | 1979 | ENSG00000029639 | processed_transcript | TFB1M | Exon | 0.00 | up |
| chr1 | 150460210 | 150464629 | 4420 | ENSG00000143374 | protein_coding | TARS2 | Exon | 0.00 | up |
| chr1 | 155563030 | 155563620 | 591 | ENSG00000235919 | lncRNA | ASH1L-AS1 | Exon | 0.00 | up |
| chr16 | 70349591 | 70366303 | 16713 | ENSG00000168872 | retained_intron | DDX19A | Exon | 0.00 | down |
| chr3 | 197705125 | 197736781 | 31657 | ENSG00000145016 | protein_coding | RUBCN | 5' UTR | 0.00 | down |
| chr20 | 31739598 | 31757485 | 17888 | ENSG00000088325 | protein_coding | TPX2 | 5' UTR | 0.00 | up |
| chr2 | 206147536 | 206147777 | 242 | ENSG00000023228 | protein_coding | NDUFS1 | 3' UTR | 0.00 | up |
| chr11 | 75262218 | 75262429 | 212 | ENSG00000279117 | TEC | AP001972 | 3' UTR | 0.00 | up |
| chr11 | 75262225 | 75262466 | 242 | ENSG00000279117 | TEC | AP001972 | 3' UTR | 0.00 | up |
| chr19 | 7467310 | 7467550 | 241 | ENSG00000104880 | protein_coding | ARHGEF18 | Exon | 0.00 | down |
| chr15 | 44733585 | 44736377 | 2793 | ENSG00000185880 | protein_coding | TRIM69 | 5' UTR | 0.00 | down |
| chr1 | 204257749 | 204259737 | 1989 | ENSG00000143850 | processed_transcript | PLEKHA6 | Exon | 0.00 | down |
| chr12 | 57773043 | 57784063 | 11021 | ENSG00000123297 | protein_coding | TSFM | 3' UTR | 0.00 | up |
| chr17 | 64128811 | 64129771 | 961 | ENSG00000178607 | retained_intron | ERN1 | 3' UTR | 0.00 | down |
| chr7 | 44041385 | 44041801 | 417 | ENSG00000235314 | lncRNA | LINC00957 | Exon | 0.00 | down |
| chr20 | 2483224 | 2484542 | 1319 | ENSG00000088876 | retained_intron | ZNF343 | Exon | 0.00 | down |
| chr8 | 27992522 | 27992673 | 152 | ENSG00000168079 | protein_coding | SCARA5 | 5' UTR | 0.00 | down |
| chr19 | 1777874 | 1778893 | 1020 | ENSG00000205922 | protein_coding | ONECUT3 | 3' UTR | 0.00 | down |
| chr3 | 58564116 | 58564446 | 331 | ENSG00000168309 | protein_coding | FAM107A | 3' UTR | 0.00 | up |
| chr14 | 58266114 | 58266715 | 602 | ENSG00000257621 | lncRNA | PSMA3-AS1 | Exon | 0.00 | down |
| chr16 | 70366066 | 70366307 | 242 | ENSG00000168872 | retained_intron | DDX19A | 3' UTR | 0.00 | down |
| chr2 | 86030339 | 86038755 | 8417 | ENSG00000068654 | processed_transcript | POLR1A | Exon | 0.00 | down |
| chr3 | 9743633 | 9745023 | 1391 | ENSG00000156983 | retained_intron | BRPF1 | 3' UTR | 0.00 | up |
| chr6 | 37381150 | 37391018 | 9869 | ENSG00000112130 | protein_coding | RNF8 | 3' UTR | 0.00 | up |
| chr11 | 117362500 | 117371115 | 8616 | ENSG00000110274 | processed_transcript | CEP164 | Exon | 0.00 | up |
| chr19 | 52652564 | 52653224 | 661 | ENSG00000269825 | lncRNA | AC022150 | Exon | 0.00 | up |
| chr9 | 137106157 | 137106458 | 302 | ENSG00000177239 | retained_intron | MAN1B1 | Exon | 0.00 | up |
| X | 129567330 | 129587099 | 19770 | ENSG00000122126 | processed_transcript | OCRL | 3' UTR | 0.00 | up |
| chr1 | 207027527 | 207032726 | 5200 | ENSG00000182795 | protein_coding | C1orf116 | 5' UTR | 0.00 | up |
| chr11 | 65309087 | 65314036 | 4950 | ENSG00000287917 | lncRNA | AP000944 | Exon | 0.00 | down |
| chr14 | 70367006 | 70367575 | 570 | ENSG00000133983 | processed_transcript | COX16 | 3' UTR | 0.00 | down |
| X | 106941456 | 106941786 | 331 | ENSG00000133131 | retained_intron | MORC4 | 3' UTR | 0.00 | down |
| chr3 | 194644929 | 194645401 | 473 | ENSG00000229334 | lncRNA | AC046143 | Exon | 0.00 | down |
| chr15 | 99728806 | 99731427 | 2622 | ENSG00000183060 | retained_intron | LYSMD4 | 3' UTR | 0.00 | up |
| chr11 | 62832292 | 62833027 | 736 | ENSG00000256690 | lncRNA | AP001160 | Exon | 0.00 | up |
| chr1 | 156638766 | 156639513 | 748 | ENSG00000272068 | lncRNA | AL365181 | Exon | 0.00 | down |
| chr2 | 235054547 | 235054698 | 152 | ENSG00000130147 | protein_coding | SH3BP4 | 3' UTR | 0.00 | down |
| chr16 | 69323416 | 69323567 | 152 | ENSG00000213380 | processed_transcript | COG8 | 3' UTR | 0.00 | down |
| chr4 | 186610014 | 186613262 | 3249 | ENSG00000083857 | retained_intron | FAT1 | Exon | 0.00 | down |
| chr9 | 98094867 | 98109942 | 15076 | ENSG00000106785 | processed_transcript | TRIM14 | 3' UTR | 0.00 | up |
| chr9 | 83665137 | 83665288 | 152 | ENSG00000135018 | retained_intron | UBQLN1 | Exon | 0.00 | up |
| chr9 | 39166061 | 39172838 | 6778 | ENSG00000243695 | processed_pseudogene | AL353729 | Exon | 0.00 | down |
| chr10 | 70146520 | 70146700 | 181 | ENSG00000156521 | protein_coding | TYSND1 | 5' UTR | 0.00 | down |
| chr19 | 19637938 | 19640468 | 2531 | ENSG00000089639 | protein_coding | GMIP | 3' UTR | 0.00 | up |
| chr1 | 42179356 | 42179807 | 452 | ENSG00000198815 | protein_coding | FOXJ3 | 3' UTR | 0.00 | up |
| chr5 | 139022566 | 139051203 | 28638 | ENSG00000120725 | protein_coding | SIL1 | Exon | 0.00 | up |
| chr10 | 69171240 | 69171690 | 451 | ENSG00000122958 | processed_transcript | VPS26A | 3' UTR | 0.00 | up |
| chr7 | 34928935 | 34929176 | 242 | ENSG00000173852 | protein_coding | DPY19L1 | 3' UTR | 0.00 | down |
| chr17 | 81880777 | 81880987 | 211 | ENSG00000263731 | lncRNA | AC145207 | Exon | 0.00 | down |
| chr8 | 118194340 | 118196049 | 1710 | ENSG00000182197 | protein_coding | EXT1 | 3' UTR | 0.00 | down |
| chr1 | 202874050 | 202874676 | 627 | ENSG00000234996 | transcribed_processed_pseudogene | AC098934 | Exon | 0.00 | down |
| chr12 | 22643756 | 22644394 | 639 | ENSG00000139163 | retained_intron | ETNK1 | 3' UTR | 0.00 | down |
| chr12 | 95266425 | 95269975 | 3551 | ENSG00000028203 | retained_intron | VEZT | 3' UTR | 0.00 | down |
| chr12 | 48692520 | 48694434 | 1915 | ENSG00000129315 | retained_intron | CCNT1 | 3' UTR | 0.00 | down |
| chr19 | 18444311 | 18445267 | 957 | ENSG00000105656 | retained_intron | ELL | 3' UTR | 0.00 | down |
| chr17 | 1050019 | 1055622 | 5604 | ENSG00000159842 | nonsense_mediated_decay | ABR | 3' UTR | 0.00 | down |
| chr11 | 123619149 | 123619390 | 242 | ENSG00000023171 | retained_intron | GRAMD1B | Exon | 0.00 | up |
| chr14 | 35028123 | 35029418 | 1296 | ENSG00000100883 | retained_intron | SRP54 | 3' UTR | 0.00 | up |
| chr16 | 30200796 | 30201005 | 210 | ENSG00000261052 | protein_coding | SULT1A3 | Exon | 0.00 | down |
| chr19 | 16893550 | 16894330 | 781 | ENSG00000160111 | protein_coding | CPAMD8 | 3' UTR | 0.00 | down |
| chr17 | 43193207 | 43194432 | 1226 | ENSG00000188554 | retained_intron | NBR1 | Exon | 0.00 | down |
| chr7 | 90154485 | 90161195 | 6711 | ENSG00000164647 | protein_coding | STEAP1 | 5' UTR | 0.00 | down |
| chr5 | 128083765 | 128084544 | 780 | ENSG00000064651 | protein_coding | SLC12A2 | 5' UTR | 0.00 | down |
| chr12 | 8092856 | 8093187 | 332 | ENSG00000089818 | protein_coding | NECAP1 | 3' UTR | 0.00 | up |
| chr16 | 56682469 | 56682619 | 151 | ENSG00000187193 | protein_coding | MT1X | 5' UTR | 0.00 | up |
| chr11 | 130448973 | 130449690 | 718 | ENSG00000166106 | protein_coding | ADAMTS15 | Exon | 0.00 | down |
| chr10 | 74825346 | 74826475 | 1130 | ENSG00000156650 | protein_coding | KAT6B | 5' UTR | 0.00 | up |
| chr1 | 78649825 | 78655209 | 5385 | ENSG00000137965 | protein_coding | IFI44 | 5' UTR | 0.00 | up |
| chr12 | 56605344 | 56605465 | 122 | ENSG00000076108 | retained_intron | BAZ2A | Exon | 0.00 | down |
| chr10 | 100548709 | 100548920 | 212 | ENSG00000166135 | retained_intron | HIF1AN | 3' UTR | 0.00 | down |
| chr11 | 123591176 | 123591537 | 362 | ENSG00000023171 | protein_coding | GRAMD1B | 5' UTR | 0.00 | up |
| chr5 | 55161022 | 55164811 | 3790 | ENSG00000164294 | retained_intron | GPX8 | 3' UTR | 0.00 | down |
| chr4 | 2836268 | 2836719 | 452 | ENSG00000087266 | nonsense_mediated_decay | SH3BP2 | 3' UTR | 0.00 | down |
| chr2 | 73227071 | 73227221 | 151 | ENSG00000135632 | processed_transcript | SMYD5 | 3' UTR | 0.00 | down |
| chr10 | 5925437 | 5928473 | 3037 | ENSG00000134452 | processed_transcript | FBH1 | Exon | 0.00 | down |
| chr20 | 62285932 | 62286597 | 666 | ENSG00000130703 | retained_intron | OSBPL2 | 3' UTR | 0.00 | down |
| chr14 | 55154775 | 55169523 | 14749 | ENSG00000126787 | retained_intron | DLGAP5 | Exon | 0.00 | down |
| chr1 | 198320771 | 198321162 | 392 | ENSG00000151414 | retained_intron | NEK7 | 3' UTR | 0.00 | down |
| chr2 | 233034119 | 233034852 | 734 | ENSG00000115488 | protein_coding | NEU2 | Exon | 0.00 | down |
| chr7 | 100887191 | 100887709 | 519 | ENSG00000087087 | retained_intron | SRRT | Exon | 0.00 | up |
| chr2 | 96250038 | 96250428 | 391 | ENSG00000135956 | protein_coding | TMEM127 | 3' UTR | 0.00 | up |
| chr1 | 27105578 | 27105849 | 272 | ENSG00000090020 | retained_intron | SLC9A1 | 3' UTR | 0.00 | up |
| chr2 | 183157479 | 183159713 | 2235 | ENSG00000272800 | lncRNA | AC021851 | 3' UTR | 0.00 | down |
| chr12 | 98515549 | 98516242 | 694 | ENSG00000120802 | processed_transcript | TMPO | Exon | 0.00 | down |
| chr1 | 155262987 | 155263288 | 302 | ENSG00000116521 | protein_coding | SCAMP3 | 3' UTR | 0.00 | up |
| chr7 | 75907622 | 75915319 | 7698 | ENSG00000127948 | nonsense_mediated_decay | POR | 5' UTR | 0.00 | down |
| chr9 | 108979353 | 108991982 | 12630 | ENSG00000119326 | processed_transcript | CTNNAL1 | 3' UTR | 0.00 | down |
| chr8 | 143295877 | 143296656 | 780 | ENSG00000185730 | protein_coding | ZNF696 | Exon | 0.00 | up |
| chr5 | 133956883 | 133968525 | 11643 | ENSG00000113583 | processed_transcript | C5orf15 | Exon | 0.00 | up |
| chr20 | 51390396 | 51398844 | 8449 | ENSG00000101096 | retained_intron | NFATC2 | 3' UTR | 0.00 | up |
| chr2 | 74662813 | 74673755 | 10943 | ENSG00000135622 | processed_transcript | SEMA4F | 3' UTR | 0.00 | up |
| chr2 | 218453153 | 218453424 | 272 | ENSG00000135913 | processed_transcript | USP37 | 3' UTR | 0.00 | down |
| chr15 | 41767716 | 41768137 | 422 | ENSG00000174197 | protein_coding | MGA | 3' UTR | 0.00 | down |
| chr12 | 43793722 | 43794261 | 540 | ENSG00000151239 | retained_intron | TWF1 | 3' UTR | 0.00 | down |
| chr12 | 56238346 | 56238796 | 451 | ENSG00000139645 | processed_transcript | ANKRD52 | 3' UTR | 0.00 | up |
| chr17 | 43520169 | 43521382 | 1214 | ENSG00000067596 | protein_coding | DHX8 | Exon | 0.00 | up |
| chr14 | 81270729 | 81277296 | 6568 | ENSG00000140022 | protein_coding | STON2 | Exon | 0.00 | up |
| chr4 | 39499857 | 39500096 | 240 | ENSG00000109814 | protein_coding | UGDH | 3' UTR | 0.00 | down |
| chr11 | 108244035 | 108247002 | 2968 | ENSG00000149311 | processed_transcript | ATM | Exon | 0.00 | down |
| chr4 | 2936765 | 2937065 | 301 | ENSG00000249673 | lncRNA | NOP14-AS1 | Exon | 0.00 | down |
| chr3 | 51944793 | 51944974 | 182 | ENSG00000041880 | retained_intron | PARP3 | 3' UTR | 0.00 | down |
| chr11 | 123724599 | 123725972 | 1374 | ENSG00000166261 | protein_coding | ZNF202 | 3' UTR | 0.00 | up |
| chr12 | 71157589 | 71157888 | 300 | ENSG00000127324 | protein_coding | TSPAN8 | 5' UTR | 0.00 | up |
| chr14 | 80497442 | 80530857 | 33416 | ENSG00000100629 | retained_intron | CEP128 | 3' UTR | 0.00 | up |
| X | 153347473 | 153349481 | 2009 | ENSG00000063587 | processed_transcript | ZNF275 | 3' UTR | 0.00 | down |
| chr22 | 41433493 | 41434063 | 571 | ENSG00000183864 | protein_coding | TOB2 | 3' UTR | 0.00 | up |
| chr1 | 1401688 | 1402046 | 359 | ENSG00000224870 | lncRNA | MRPL20-AS1 | Exon | 0.00 | up |
| chr19 | 18929408 | 18931401 | 1994 | ENSG00000051128 | retained_intron | HOMER3 | 3' UTR | 0.00 | up |
| chr22 | 35648595 | 35659838 | 11244 | ENSG00000221963 | protein_coding | APOL6 | 3' UTR | 0.00 | up |
| chr6 | 142218567 | 142219137 | 571 | ENSG00000009844 | processed_transcript | VTA1 | 3' UTR | 0.00 | up |
| chr17 | 20008139 | 20008672 | 534 | ENSG00000261033 | lncRNA | AC005730 | Exon | 0.00 | down |
| chr3 | 10151776 | 10152767 | 992 | ENSG00000134086 | processed_transcript | VHL | 3' UTR | 0.00 | down |
| chr11 | 67252701 | 67253506 | 806 | ENSG00000173120 | processed_transcript | KDM2A | 3' UTR | 0.00 | down |
| chr3 | 75678809 | 75709811 | 31003 | ENSG00000243422 | processed_transcript | RPL23AP49 | Exon | 0.00 | up |
| chr3 | 100339161 | 100341159 | 1999 | ENSG00000114021 | retained_intron | NIT2 | 3' UTR | 0.00 | up |
| chr1 | 65226903 | 65227503 | 601 | ENSG00000116675 | processed_transcript | DNAJC6 | 3' UTR | 0.00 | down |
| chr19 | 49927701 | 49929539 | 1839 | ENSG00000104951 | nonsense_mediated_decay | IL4I1 | 5' UTR | 0.00 | up |
| chr6 | 158503675 | 158506854 | 3180 | ENSG00000146433 | protein_coding | TMEM181 | 3' UTR | 0.00 | up |
| chr9 | 35108570 | 35108901 | 332 | ENSG00000005238 | protein_coding | FAM214B | 5' UTR | 0.00 | down |
| chr16 | 15586774 | 15587914 | 1141 | ENSG00000166780 | processed_transcript | BMERB1 | 3' UTR | 0.00 | down |
| chr2 | 85549089 | 85550716 | 1628 | ENSG00000115486 | processed_transcript | GGCX | 3' UTR | 0.00 | up |
| chr5 | 168371505 | 168408557 | 37053 | ENSG00000113645 | retained_intron | WWC1 | Exon | 0.00 | up |
| chr2 | 120160911 | 120175220 | 14310 | ENSG00000115109 | processed_transcript | EPB41L5 | 3' UTR | 0.00 | up |
| chr20 | 58651792 | 58652062 | 271 | ENSG00000124222 | nonsense_mediated_decay | STX16 | 5' UTR | 0.00 | up |
| chr10 | 20780901 | 20781622 | 722 | ENSG00000078114 | retained_intron | NEBL | 3' UTR | 0.00 | up |
| chr16 | 29995415 | 29995595 | 181 | ENSG00000149929 | retained_intron | HIRIP3 | Exon | 0.00 | up |
| chr1 | 43899221 | 43899708 | 488 | ENSG00000126091 | protein_coding | ST3GAL3 | 3' UTR | 0.00 | down |
| chr10 | 112448901 | 112449411 | 511 | ENSG00000151532 | processed_transcript | VTI1A | Exon | 0.00 | down |
| chr19 | 50047021 | 50048461 | 1441 | ENSG00000204666 | lncRNA | AC010624 | 3' UTR | 0.00 | down |
| chr18 | 3261478 | 3262528 | 1051 | ENSG00000118680 | protein_coding | MYL12B | 5' UTR | 0.00 | down |
| chr1 | 161153788 | 161159349 | 5562 | ENSG00000143222 | protein_coding | UFC1 | Exon | 0.00 | down |
| chr16 | 85665071 | 85666429 | 1359 | ENSG00000131149 | retained_intron | GSE1 | Exon | 0.00 | down |
| chr13 | 110643796 | 110644337 | 542 | ENSG00000134905 | processed_transcript | CARS2 | 3' UTR | 0.00 | up |
| chr14 | 69227985 | 69230488 | 2504 | ENSG00000081177 | processed_transcript | EXD2 | 5' UTR | 0.00 | up |
| chr1 | 68125617 | 68126246 | 630 | ENSG00000116729 | processed_transcript | WLS | 3' UTR | 0.00 | down |
| chr6 | 26422947 | 26426265 | 3319 | ENSG00000124549 | processed_transcript | BTN2A3P | Exon | 0.00 | up |
| chr14 | 35122590 | 35122711 | 122 | ENSG00000100890 | protein_coding | PRORP | 5' UTR | 0.00 | up |
| chr11 | 85626677 | 85627832 | 1156 | ENSG00000150672 | protein_coding | DLG2 | 5' UTR | 0.00 | up |
| chr12 | 109087215 | 109087694 | 480 | ENSG00000135093 | nonsense_mediated_decay | USP30 | 3' UTR | 0.00 | down |
| chr11 | 6599894 | 6600404 | 511 | ENSG00000132275 | retained_intron | RRP8 | 3' UTR | 0.00 | down |
| chr16 | 66730238 | 66730987 | 750 | ENSG00000135720 | retained_intron | DYNC1LI2 | Exon | 0.00 | up |
| chr12 | 56152584 | 56153618 | 1035 | ENSG00000196465 | protein_coding | MYL6B | 5' UTR | 0.00 | up |
| chr7 | 102375875 | 102396388 | 20514 | ENSG00000128563 | protein_coding | PRKRIP1 | 5' UTR | 0.00 | up |
| chr11 | 78068953 | 78069522 | 570 | ENSG00000259112 | protein_coding | NDUFC2-KCTD14 | 3' UTR | 0.00 | down |
| chr17 | 75666709 | 75667189 | 481 | ENSG00000108469 | protein_coding | RECQL5 | 5' UTR | 0.00 | up |
| chr5 | 43044399 | 43045177 | 779 | ENSG00000215068 | lncRNA | AC025171 | Exon | 0.00 | up |
| chr19 | 45409930 | 45410319 | 390 | ENSG00000117877 | protein_coding | CD3EAP | 3' UTR | 0.00 | down |
| chr19 | 8374372 | 8377823 | 3452 | ENSG00000269386 | lncRNA | RAB11B-AS1 | Exon | 0.00 | down |
| chr13 | 110879523 | 110883351 | 3829 | ENSG00000255874 | lncRNA | PRECSIT | 3' UTR | 0.00 | down |
| chr3 | 27434072 | 27452417 | 18346 | ENSG00000033867 | protein_coding | SLC4A7 | Exon | 0.00 | down |
| chr1 | 109094485 | 109095113 | 629 | ENSG00000215717 | processed_transcript | TMEM167B | 3' UTR | 0.00 | down |
| chr1 | 150283531 | 150284790 | 1260 | ENSG00000159208 | processed_transcript | CIART | 5' UTR | 0.00 | up |
| chr3 | 48695319 | 48699362 | 4044 | ENSG00000068745 | retained_intron | IP6K2 | 5' UTR | 0.00 | up |
| chr1 | 26473464 | 26473912 | 449 | ENSG00000198830 | processed_transcript | HMGN2 | Exon | 0.00 | up |
| chr1 | 236897040 | 236898410 | 1371 | ENSG00000116984 | processed_transcript | MTR | 3' UTR | 0.00 | up |
| chr2 | 148458280 | 148458851 | 572 | ENSG00000204406 | nonsense_mediated_decay | MBD5 | 5' UTR | 0.00 | up |
| chr1 | 184794558 | 184795158 | 601 | ENSG00000135842 | protein_coding | NIBAN1 | 3' UTR | 0.00 | up |
| chr1 | 201622884 | 201623604 | 721 | ENSG00000134369 | protein_coding | NAV1 | 5' UTR | 0.00 | down |
| chr9 | 136920299 | 136921061 | 763 | ENSG00000127191 | processed_transcript | TRAF2 | Exon | 0.00 | down |
| chr10 | 69934902 | 69941332 | 6431 | ENSG00000197467 | processed_transcript | COL13A1 | 3' UTR | 0.00 | down |
| chr1 | 156082164 | 156082465 | 302 | ENSG00000254726 | protein_coding | MEX3A | 5' UTR | 0.00 | down |
| chr4 | 2061155 | 2064533 | 3379 | ENSG00000185818 | protein_coding | NAT8L | 3' UTR | 0.00 | down |
| chr5 | 177301436 | 177302033 | 598 | ENSG00000169228 | retained_intron | RAB24 | 3' UTR | 0.00 | up |
| chr5 | 150379379 | 150379713 | 335 | ENSG00000070814 | nonsense_mediated_decay | TCOF1 | Exon | 0.00 | up |
| chr7 | 72925339 | 72925759 | 421 | ENSG00000196313 | protein_coding | POM121 | Exon | 0.00 | down |
| chr14 | 21498222 | 21499147 | 926 | ENSG00000165819 | retained_intron | METTL3 | 3' UTR | 0.00 | up |
| chr7 | 44072091 | 44072361 | 271 | ENSG00000283969 | miRNA | MIR6838 | 3' UTR | 0.00 | up |
| chr17 | 75945813 | 75946053 | 241 | ENSG00000161533 | processed_transcript | ACOX1 | 3' UTR | 0.00 | up |
| chr4 | 119606717 | 119607138 | 422 | ENSG00000138735 | protein_coding | PDE5A | Exon | 0.00 | down |
| chr14 | 60109835 | 60110915 | 1081 | ENSG00000126773 | retained_intron | PCNX4 | 3' UTR | 0.00 | down |
| chr17 | 16027399 | 16027640 | 242 | ENSG00000011295 | protein_coding | TTC19 | 3' UTR | 0.00 | down |
| chr19 | 1397988 | 1398760 | 773 | ENSG00000130005 | retained_intron | GAMT | 3' UTR | 0.00 | down |
| chr14 | 64786449 | 64791757 | 5309 | ENSG00000284269 | miRNA | MIR7855 | Exon | 0.00 | up |
| chr11 | 88321438 | 88322578 | 1141 | ENSG00000109861 | protein_coding | CTSC | 3' UTR | 0.00 | down |
| chr17 | 4064745 | 4070876 | 6132 | ENSG00000074755 | retained_intron | ZZEF1 | 3' UTR | 0.00 | down |
| chr14 | 51689749 | 51704635 | 14887 | ENSG00000139926 | retained_intron | FRMD6 | 5' UTR | 0.00 | down |
| chr2 | 222631629 | 222648782 | 17154 | ENSG00000116120 | protein_coding | FARSB | Exon | 0.00 | up |
| chr16 | 10767951 | 10769321 | 1371 | ENSG00000103274 | processed_transcript | NUBP1 | 3' UTR | 0.00 | up |
| chr3 | 51645694 | 51656004 | 10311 | ENSG00000164081 | protein_coding | TEX264 | Exon | 0.00 | up |
| chr6 | 24840473 | 24847595 | 7123 | ENSG00000111913 | processed_transcript | RIPOR2 | 3' UTR | 0.00 | up |
| chr11 | 43445985 | 43446465 | 481 | ENSG00000052841 | retained_intron | TTC17 | 3' UTR | 0.00 | down |
| chr18 | 74508820 | 74509090 | 271 | ENSG00000133313 | retained_intron | CNDP2 | 3' UTR | 0.00 | up |
| chr2 | 238189881 | 238194423 | 4543 | ENSG00000132323 | retained_intron | ILKAP | Exon | 0.00 | up |
| chr19 | 4100164 | 4100464 | 301 | ENSG00000126934 | processed_transcript | MAP2K2 | Exon | 0.00 | down |
| chr1 | 156539837 | 156540914 | 1078 | ENSG00000183856 | processed_transcript | IQGAP3 | Exon | 0.00 | up |
| chr14 | 20684535 | 20688781 | 4247 | ENSG00000214274 | processed_transcript | ANG | 5' UTR | 0.00 | up |
| chr7 | 144186921 | 144187672 | 752 | ENSG00000213214 | protein_coding | ARHGEF35 | 3' UTR | 0.00 | up |
| chr3 | 75676602 | 75679318 | 2717 | ENSG00000242516 | lncRNA | LINC00960 | Exon | 0.00 | up |
| chr3 | 112570619 | 112581297 | 10679 | ENSG00000138459 | retained_intron | SLC35A5 | Exon | 0.00 | up |
| chr18 | 24181246 | 24271749 | 90504 | ENSG00000141447 | nonsense_mediated_decay | OSBPL1A | 3' UTR | 0.00 | up |
| X | 48561833 | 48561983 | 151 | ENSG00000102317 | processed_transcript | RBM3 | 3' UTR | 0.00 | down |
| chr7 | 56078417 | 56079353 | 937 | ENSG00000129103 | retained_intron | SUMF2 | 3' UTR | 0.00 | up |
| chr1 | 15756636 | 15758029 | 1394 | ENSG00000162458 | protein_coding | FBLIM1 | 5' UTR | 0.00 | up |
| chr6 | 89636887 | 89637737 | 851 | ENSG00000083099 | processed_transcript | LYRM2 | 3' UTR | 0.00 | up |
| chr9 | 128307550 | 128307731 | 182 | ENSG00000167112 | processed_transcript | TRUB2 | 3' UTR | 0.00 | down |
| chr3 | 129436520 | 129439730 | 3211 | ENSG00000129071 | nonsense_mediated_decay | MBD4 | Exon | 0.00 | down |
| chr1 | 107056733 | 107057632 | 900 | ENSG00000198890 | nonsense_mediated_decay | PRMT6 | Exon | 0.00 | down |
| chr19 | 38891962 | 38893709 | 1748 | ENSG00000068903 | retained_intron | SIRT2 | 3' UTR | 0.00 | down |
| chr11 | 71501379 | 71501710 | 332 | ENSG00000172890 | processed_transcript | NADSYN1 | 3' UTR | 0.00 | up |
| X | 54530362 | 54539173 | 8812 | ENSG00000130119 | processed_transcript | GNL3L | 5' UTR | 0.00 | up |
| chr2 | 38318608 | 38354175 | 35568 | ENSG00000119787 | protein_coding | ATL2 | 3' UTR | 0.00 | down |
| chr6 | 32184789 | 32185882 | 1094 | ENSG00000273333 | lncRNA | AL662884 | 3' UTR | 0.00 | down |
| chr8 | 144261305 | 144261940 | 636 | ENSG00000260428 | protein_coding | SCX | 3' UTR | 0.00 | down |
| chr8 | 70637613 | 70640706 | 3094 | ENSG00000147592 | retained_intron | LACTB2 | 3' UTR | 0.00 | down |
| chr20 | 63570239 | 63570762 | 524 | ENSG00000130589 | processed_transcript | HELZ2 | Exon | 0.00 | up |
| chr1 | 38855803 | 38859503 | 3701 | ENSG00000116954 | processed_transcript | RRAGC | Exon | 0.00 | up |
| chr3 | 113656272 | 113657143 | 872 | ENSG00000176542 | retained_intron | USF3 | Exon | 0.00 | down |
| chr11 | 126292921 | 126294254 | 1334 | ENSG00000254905 | lncRNA | AP001318 | Exon | 0.00 | down |
| chr1 | 182887233 | 182887473 | 241 | ENSG00000135829 | processed_transcript | DHX9 | 3' UTR | 0.00 | up |
| chr3 | 160433643 | 160434063 | 421 | ENSG00000213186 | protein_coding | TRIM59 | 3' UTR | 0.00 | down |
| chr9 | 37537146 | 37537891 | 746 | ENSG00000147912 | processed_transcript | FBXO10 | Exon | 0.00 | down |
| chr15 | 38355752 | 38355933 | 182 | ENSG00000171262 | protein_coding | FAM98B | 3' UTR | 0.00 | up |
| chr1 | 43451698 | 43451968 | 271 | ENSG00000178922 | retained_intron | HYI | 3' UTR | 0.00 | down |
| chr6 | 26215187 | 26216250 | 1064 | ENSG00000273802 | protein_coding | H2BC8 | 3' UTR | 0.00 | up |
| chr8 | 119416445 | 119416840 | 396 | ENSG00000136999 | protein_coding | CCN3 | 5' UTR | 0.00 | up |
| chr1 | 227921464 | 227924289 | 2826 | ENSG00000264483 | miRNA | MIR5008 | 3' UTR | 0.00 | up |
| chr1 | 11069579 | 11071109 | 1531 | ENSG00000171824 | retained_intron | EXOSC10 | Exon | 0.00 | up |
| chr1 | 86734608 | 86738686 | 4079 | ENSG00000097033 | retained_intron | SH3GLB1 | Exon | 0.00 | down |
| chr12 | 110536714 | 110539910 | 3197 | ENSG00000196850 | retained_intron | PPTC7 | 3' UTR | 0.00 | down |
| chr2 | 42326203 | 42330901 | 4699 | ENSG00000143924 | processed_transcript | EML4 | 3' UTR | 0.00 | down |
| chr1 | 24843960 | 24844291 | 332 | ENSG00000169504 | protein_coding | CLIC4 | 3' UTR | 0.00 | down |
| chr9 | 71745140 | 71750170 | 5031 | ENSG00000135048 | retained_intron | CEMIP2 | Exon | 0.00 | up |
| chr6 | 70588848 | 70589569 | 722 | ENSG00000271967 | lncRNA | AL583856 | 3' UTR | 0.00 | down |
| chr9 | 14146726 | 14180694 | 33969 | ENSG00000147862 | protein_coding | NFIB | 5' UTR | 0.00 | down |
| chr7 | 135171829 | 135172414 | 586 | ENSG00000287733 | lncRNA | AC083862 | Exon | 0.00 | up |
| chr22 | 50445807 | 50445957 | 151 | ENSG00000100241 | retained_intron | SBF1 | 3' UTR | 0.00 | down |
| chr7 | 151080536 | 151080836 | 301 | ENSG00000164896 | protein_coding | FASTK | 5' UTR | 0.00 | up |
| chr16 | 89279507 | 89280828 | 1322 | ENSG00000167522 | retained_intron | ANKRD11 | 3' UTR | 0.00 | up |
| chr12 | 122144348 | 122144978 | 631 | ENSG00000175727 | protein_coding | MLXIP | 3' UTR | 0.00 | up |
| chr21 | 41458645 | 41459184 | 540 | ENSG00000157601 | retained_intron | MX1 | 3' UTR | 0.00 | up |
| chr17 | 7593452 | 7594133 | 682 | ENSG00000129245 | retained_intron | FXR2 | Exon | 0.00 | down |
| chr15 | 64171516 | 64171966 | 451 | ENSG00000166794 | protein_coding | PPIB | 3' UTR | 0.00 | up |
| chr6 | 42881208 | 42881509 | 302 | ENSG00000146223 | processed_transcript | RPL7L1 | Exon | 0.00 | down |
| chr12 | 57154550 | 57154701 | 152 | ENSG00000123384 | retained_intron | LRP1 | Exon | 0.00 | down |
| chr1 | 110041802 | 110043025 | 1224 | ENSG00000143093 | retained_intron | STRIP1 | 3' UTR | 0.00 | up |
| chr11 | 116857787 | 116858508 | 722 | ENSG00000160584 | retained_intron | SIK3 | 3' UTR | 0.00 | down |
| chr1 | 16625517 | 16625788 | 272 | ENSG00000215908 | processed_transcript | CROCCP2 | Exon | 0.00 | down |
| chr1 | 155324501 | 155324712 | 212 | ENSG00000160753 | protein_coding | RUSC1 | 5' UTR | 0.00 | down |
| chr9 | 37536057 | 37537914 | 1858 | ENSG00000147912 | processed_transcript | FBXO10 | Exon | 0.00 | down |
| chr3 | 45679456 | 45679787 | 332 | ENSG00000230530 | lncRNA | LIMD1-AS1 | Exon | 0.00 | down |
| chr4 | 139058507 | 139059556 | 1050 | ENSG00000109381 | processed_transcript | ELF2 | 3' UTR | 0.00 | up |
| chr3 | 8767706 | 8768006 | 301 | ENSG00000180914 | protein_coding | OXTR | Exon | 0.00 | down |
| chr14 | 50634934 | 50664380 | 29447 | ENSG00000151748 | protein_coding | SAV1 | 3' UTR | 0.00 | down |
| chr2 | 68655283 | 68657442 | 2160 | ENSG00000169621 | protein_coding | APLF | 3' UTR | 0.00 | down |
| chr19 | 39369998 | 39375732 | 5735 | ENSG00000179134 | protein_coding | SAMD4B | Exon | 0.00 | down |
| chr19 | 4043302 | 4043782 | 481 | ENSG00000268670 | lncRNA | AC016586 | 3' UTR | 0.00 | up |
| chr22 | 20127144 | 20127355 | 212 | ENSG00000264346 | snoRNA | SNORA77B | 3' UTR | 0.00 | down |
| chr20 | 58651792 | 58652033 | 242 | ENSG00000124222 | nonsense_mediated_decay | STX16 | 5' UTR | 0.00 | up |
| chr7 | 44042038 | 44042306 | 269 | ENSG00000136279 | protein_coding | DBNL | Exon | 0.00 | down |
| chr7 | 98291468 | 98306619 | 15152 | ENSG00000164713 | processed_transcript | BRI3 | 3' UTR | 0.00 | down |
| chr1 | 145824236 | 145824400 | 165 | ENSG00000186141 | processed_transcript | POLR3C | 5' UTR | 0.00 | down |
| chr6 | 143503509 | 143506021 | 2513 | ENSG00000229036 | retained_intron | VDAC1P8 | Exon | 0.00 | down |
| chr20 | 33358838 | 33359257 | 420 | ENSG00000101391 | retained_intron | CDK5RAP1 | 3' UTR | 0.00 | up |
| chr6 | 33441684 | 33448069 | 6386 | ENSG00000197283 | processed_transcript | SYNGAP1 | 3' UTR | 0.00 | up |
| chr20 | 38002701 | 38006003 | 3303 | ENSG00000101407 | protein_coding | TTI1 | 3' UTR | 0.00 | up |
| chr5 | 43527128 | 43535610 | 8483 | ENSG00000172239 | protein_coding | PAIP1 | 3' UTR | 0.00 | up |
| chr1 | 149054805 | 149055075 | 271 | ENSG00000269713 | retained_intron | NBPF9 | 3' UTR | 0.00 | down |
| chr7 | 7639100 | 7640663 | 1564 | ENSG00000106399 | protein_coding | RPA3 | 5' UTR | 0.00 | down |
| chr1 | 171512580 | 171512851 | 272 | ENSG00000117523 | protein_coding | PRRC2C | Exon | 0.00 | up |
| chr11 | 119372854 | 119380774 | 7921 | ENSG00000036672 | protein_coding | USP2 | Exon | 0.00 | down |
| chr11 | 66683065 | 66685043 | 1979 | ENSG00000173898 | retained_intron | SPTBN2 | 3' UTR | 0.00 | down |
| chr3 | 100736566 | 100739481 | 2916 | ENSG00000114354 | retained_intron | TFG | 3' UTR | 0.00 | up |
| chr1 | 110043084 | 110045054 | 1971 | ENSG00000143093 | retained_intron | STRIP1 | 3' UTR | 0.00 | down |
| chr5 | 139042656 | 139108093 | 65438 | ENSG00000120725 | processed_transcript | SIL1 | Exon | 0.00 | up |
| chr7 | 100866739 | 100867010 | 272 | ENSG00000087077 | retained_intron | TRIP6 | 3' UTR | 0.00 | down |
| chr14 | 75971689 | 75982832 | 11144 | ENSG00000119699 | protein_coding | TGFB3 | 5' UTR | 0.00 | up |
| chr4 | 82851588 | 82857941 | 6354 | ENSG00000138674 | retained_intron | SEC31A | Exon | 0.00 | up |
| chr1 | 1598011 | 1598550 | 540 | ENSG00000228594 | protein_coding | FNDC10 | 3' UTR | 0.00 | up |
| chr10 | 72533144 | 72563049 | 29906 | ENSG00000107745 | nonsense_mediated_decay | MICU1 | Exon | 0.00 | down |
| chr17 | 58999143 | 59012347 | 13205 | ENSG00000108395 | protein_coding | TRIM37 | 3' UTR | 0.00 | up |
| chr10 | 100996647 | 100998885 | 2239 | ENSG00000107816 | protein_coding | LZTS2 | 5' UTR | 0.00 | down |
| chr3 | 197700706 | 197701037 | 332 | ENSG00000145016 | protein_coding | RUBCN | Exon | 0.00 | up |
| chr14 | 69236067 | 69236531 | 465 | ENSG00000081177 | retained_intron | EXD2 | Exon | 0.00 | up |
| chr7 | 35903130 | 35904768 | 1639 | ENSG00000122545 | protein_coding | SEPTIN7 | 3' UTR | 0.00 | down |
| chr11 | 63765139 | 63767398 | 2260 | ENSG00000188070 | protein_coding | C11orf95 | 3' UTR | 0.00 | down |
| chr1 | 205662885 | 205664555 | 1671 | ENSG00000158715 | processed_transcript | SLC45A3 | Exon | 0.00 | down |
| chr12 | 113389595 | 113390044 | 450 | ENSG00000151176 | processed_transcript | PLBD2 | 3' UTR | 0.00 | down |
| chr14 | 93185414 | 93187086 | 1673 | ENSG00000153485 | protein_coding | TMEM251 | 3' UTR | 0.00 | up |
| chr3 | 160521872 | 160526065 | 4194 | ENSG00000186432 | protein_coding | KPNA4 | Exon | 0.00 | down |
| chr19 | 36140372 | 36140737 | 366 | ENSG00000126247 | protein_coding | CAPNS1 | 5' UTR | 0.00 | down |
| chr1 | 171523476 | 171535553 | 12078 | ENSG00000117523 | processed_transcript | PRRC2C | 3' UTR | 0.00 | up |
| chr18 | 80148193 | 80149200 | 1008 | ENSG00000267270 | lncRNA | PARD6G-AS1 | Exon | 0.00 | up |
| chr10 | 6234919 | 6235280 | 362 | ENSG00000170525 | nonsense_mediated_decay | PFKFB3 | 3' UTR | 0.00 | down |
| chr3 | 45679448 | 45679779 | 332 | ENSG00000211456 | processed_transcript | SACM1L | 3' UTR | 0.00 | down |
| chr2 | 46901959 | 46902378 | 420 | ENSG00000180398 | protein_coding | MCFD2 | 3' UTR | 0.00 | up |
| chr4 | 173371457 | 173377473 | 6017 | ENSG00000164105 | protein_coding | SAP30 | 3' UTR | 0.00 | down |
| chr17 | 60447322 | 60456356 | 9035 | ENSG00000062725 | processed_transcript | APPBP2 | 3' UTR | 0.00 | down |
| chr16 | 89948763 | 89949606 | 844 | ENSG00000140995 | protein_coding | DEF8 | 5' UTR | 0.00 | up |
| X | 153351756 | 153352416 | 661 | ENSG00000063587 | processed_transcript | ZNF275 | 3' UTR | 0.00 | up |
| chr11 | 61815448 | 61815688 | 241 | ENSG00000149485 | protein_coding | FADS1 | Exon | 0.00 | down |
| chr19 | 35510145 | 35510505 | 361 | ENSG00000161249 | protein_coding | DMKN | 3' UTR | 0.00 | up |
| chr22 | 24558080 | 24572165 | 14086 | ENSG00000286070 | nonsense_mediated_decay | AP000356 | 3' UTR | 0.00 | up |
| chr15 | 41280511 | 41280960 | 450 | ENSG00000247556 | retained_intron | OIP5-AS1 | 3' UTR | 0.00 | down |
| chr22 | 30893966 | 30894117 | 152 | ENSG00000184792 | protein_coding | OSBP2 | 3' UTR | 0.00 | up |
| chr16 | 29973867 | 29974048 | 182 | ENSG00000149930 | protein_coding | TAOK2 | 5' UTR | 0.00 | up |
| chr6 | 112254156 | 112254517 | 362 | ENSG00000112769 | protein_coding | LAMA4 | 5' UTR | 0.00 | down |
| chr19 | 10701429 | 10701609 | 181 | ENSG00000213339 | retained_intron | QTRT1 | 5' UTR | 0.00 | down |
| chr17 | 1420897 | 1421316 | 420 | ENSG00000108953 | protein_coding | YWHAE | 3' UTR | 0.00 | up |
| chr6 | 143503914 | 143504585 | 672 | ENSG00000229036 | retained_intron | VDAC1P8 | Exon | 0.00 | down |
| chr5 | 476119 | 477097 | 979 | ENSG00000225138 | lncRNA | SLC9A3-AS1 | Exon | 0.00 | up |
| chr11 | 74949246 | 74949610 | 365 | ENSG00000118363 | protein_coding | SPCS2 | 5' UTR | 0.00 | up |
| chr4 | 113513337 | 113537371 | 24035 | ENSG00000145349 | protein_coding | CAMK2D | Exon | 0.00 | down |
| chr2 | 134452945 | 134453185 | 241 | ENSG00000152127 | protein_coding | MGAT5 | 3' UTR | 0.00 | down |
| chr1 | 179900131 | 179918471 | 18341 | ENSG00000143337 | protein_coding | TOR1AIP1 | 3' UTR | 0.00 | up |
| chr5 | 116051916 | 116052232 | 317 | ENSG00000185641 | processed_pseudogene | AC034236 | Exon | 0.00 | up |
| chr11 | 93820854 | 93821445 | 592 | ENSG00000214376 | nonsense_mediated_decay | VSTM5 | Exon | 0.00 | down |
| chr2 | 74556069 | 74557315 | 1247 | ENSG00000115325 | nonsense_mediated_decay | DOK1 | 3' UTR | 0.00 | up |
| chr17 | 81922192 | 81922342 | 151 | ENSG00000187531 | protein_coding | SIRT7 | 3' UTR | 0.00 | down |
| chr3 | 120750545 | 120776425 | 25881 | ENSG00000153767 | retained_intron | GTF2E1 | 5' UTR | 0.00 | up |
| chr8 | 103300009 | 103307774 | 7766 | ENSG00000164930 | nonsense_mediated_decay | FZD6 | 3' UTR | 0.00 | down |
| chr21 | 44128055 | 44130971 | 2917 | ENSG00000241945 | retained_intron | PWP2 | 3' UTR | 0.00 | down |
| chr6 | 29726933 | 29728013 | 1081 | ENSG00000239257 | transcribed_processed_pseudogene | RPL23AP1 | 3' UTR | 0.00 | down |
| chr17 | 21000794 | 21001065 | 272 | ENSG00000124422 | retained_intron | USP22 | 3' UTR | 0.00 | down |
| chr1 | 41513397 | 41580773 | 67377 | ENSG00000127124 | processed_transcript | HIVEP3 | Exon | 0.00 | down |
| chr19 | 55039227 | 55040504 | 1278 | ENSG00000088053 | protein_coding | GP6 | 3' UTR | 0.00 | down |
| chr2 | 86045757 | 86048983 | 3227 | ENSG00000068654 | processed_transcript | POLR1A | Exon | 0.00 | down |
| chr7 | 5307926 | 5308227 | 302 | ENSG00000182095 | protein_coding | TNRC18 | 3' UTR | 0.00 | down |
| chr5 | 157493049 | 157494709 | 1661 | ENSG00000135074 | protein_coding | ADAM19 | 3' UTR | 0.00 | up |
| chr12 | 55818040 | 55818437 | 398 | ENSG00000123353 | protein_coding | ORMDL2 | 5' UTR | 0.00 | up |
| chr20 | 299991 | 300201 | 211 | ENSG00000247315 | protein_coding | ZCCHC3 | 3' UTR | 0.00 | down |
| chr15 | 44527375 | 44533656 | 6282 | ENSG00000179523 | lncRNA | EIF3J-DT | Exon | 0.00 | down |
| chr15 | 44527391 | 44527691 | 301 | ENSG00000137770 | retained_intron | CTDSPL2 | 3' UTR | 0.00 | down |
| chr20 | 3038415 | 3038686 | 272 | ENSG00000125787 | protein_coding | GNRH2 | 3' UTR | 0.00 | down |
| chr12 | 43786456 | 43787238 | 783 | ENSG00000198001 | retained_intron | IRAK4 | 3' UTR | 0.00 | down |
| chr6 | 29726660 | 29728483 | 1824 | ENSG00000239257 | transcribed_processed_pseudogene | RPL23AP1 | Exon | 0.00 | down |
| chr8 | 86474061 | 86474503 | 443 | ENSG00000176623 | protein_coding | RMDN1 | 3' UTR | 0.00 | down |
| chr1 | 1409960 | 1410290 | 331 | ENSG00000272455 | lncRNA | MRPL20-DT | Exon | 0.00 | down |
| chr10 | 46005087 | 46005418 | 332 | ENSG00000266412 | protein_coding | NCOA4 | 3' UTR | 0.00 | down |
| chr2 | 74440601 | 74442422 | 1822 | ENSG00000114993 | protein_coding | RTKN | 5' UTR | 0.00 | down |
| chr17 | 4890729 | 4891605 | 877 | ENSG00000141503 | retained_intron | MINK1 | 3' UTR | 0.00 | up |
| chr7 | 56011466 | 56011853 | 388 | ENSG00000146733 | protein_coding | PSPH | 3' UTR | 0.00 | down |
| chr1 | 1484101 | 1484700 | 600 | ENSG00000160072 | retained_intron | ATAD3B | Exon | 0.00 | down |
| chr6 | 142770098 | 142770818 | 721 | ENSG00000010818 | processed_transcript | HIVEP2 | Exon | 0.00 | down |
| chr1 | 36341535 | 36342014 | 480 | ENSG00000142694 | protein_coding | EVA1B | 3' UTR | 0.00 | down |
| chr17 | 78674945 | 78676263 | 1319 | ENSG00000108669 | retained_intron | CYTH1 | 3' UTR | 0.00 | up |
| chr8 | 144427159 | 144428533 | 1375 | ENSG00000160948 | retained_intron | VPS28 | 5' UTR | 0.00 | down |
| chr3 | 9742900 | 9743349 | 450 | ENSG00000156983 | retained_intron | BRPF1 | 3' UTR | 0.00 | down |
| chr2 | 14632699 | 14633708 | 1010 | ENSG00000162981 | protein_coding | LRATD1 | 5' UTR | 0.00 | up |
| chr2 | 158621302 | 158631804 | 10503 | ENSG00000144283 | processed_transcript | PKP4 | 3' UTR | 0.00 | up |
| chr19 | 35624935 | 35625205 | 271 | ENSG00000126254 | protein_coding | RBM42 | 3' UTR | 0.00 | up |
| chr20 | 50096651 | 50108973 | 12323 | ENSG00000244687 | processed_transcript | UBE2V1 | 5' UTR | 0.00 | up |
| chr5 | 181199908 | 181200179 | 272 | ENSG00000146054 | protein_coding | TRIM7 | 5' UTR | 0.00 | up |
| chr7 | 155003892 | 155004635 | 744 | ENSG00000273344 | lncRNA | PAXIP1-AS1 | Exon | 0.00 | down |
| chr17 | 28723519 | 28723758 | 240 | ENSG00000238597 | snoRNA | SNORD4B | 3' UTR | 0.00 | down |
| chr2 | 32023767 | 32029710 | 5944 | ENSG00000162961 | processed_transcript | DPY30 | 3' UTR | 0.00 | up |
| chr20 | 62197822 | 62198093 | 272 | ENSG00000101181 | processed_transcript | MTG2 | Exon | 0.00 | down |
| X | 54443449 | 54444616 | 1168 | ENSG00000158526 | protein_coding | TSR2 | 3' UTR | 0.00 | down |
| chr2 | 63404026 | 63433773 | 29748 | ENSG00000143951 | nonsense_mediated_decay | WDPCP | 3' UTR | 0.00 | up |
| chr13 | 113865454 | 113866295 | 842 | ENSG00000272695 | lncRNA | GAS6-DT | Exon | 0.00 | up |
| chr15 | 79384606 | 79412034 | 27429 | ENSG00000169330 | protein_coding | MINAR1 | 3' UTR | 0.00 | down |
| chr11 | 843699 | 844459 | 761 | ENSG00000214063 | protein_coding | TSPAN4 | 5' UTR | 0.00 | down |
| chr16 | 70576709 | 70577130 | 422 | ENSG00000189091 | processed_transcript | SF3B3 | 3' UTR | 0.00 | down |
| chr3 | 122718367 | 122720383 | 2017 | ENSG00000173193 | processed_transcript | PARP14 | 3' UTR | 0.00 | up |
| X | 300696 | 301175 | 480 | ENSG00000182378 | protein_coding | PLCXD1 | 3' UTR | 0.00 | down |
| chr17 | 28400756 | 28401086 | 331 | ENSG00000076351 | retained_intron | SLC46A1 | 3' UTR | 0.00 | down |
| chr17 | 5990005 | 6080792 | 90788 | ENSG00000179314 | processed_transcript | WSCD1 | 5' UTR | 0.00 | up |
| chr2 | 101269217 | 101269782 | 566 | ENSG00000158435 | protein_coding | CNOT11 | 3' UTR | 0.00 | down |
| chr1 | 183240033 | 183240786 | 754 | ENSG00000058085 | processed_transcript | LAMC2 | 3' UTR | 0.00 | up |
| chr11 | 68158878 | 68166800 | 7923 | ENSG00000110066 | processed_transcript | KMT5B | 3' UTR | 0.00 | down |
| chr3 | 183773663 | 183786254 | 12592 | ENSG00000163872 | protein_coding | YEATS2 | Exon | 0.00 | down |
| chr7 | 152025823 | 152027715 | 1893 | ENSG00000178234 | protein_coding | GALNT11 | 5' UTR | 0.00 | down |
| chr18 | 35468398 | 35468697 | 300 | ENSG00000267583 | lncRNA | AC007998 | 3' UTR | 0.00 | up |
| chr1 | 52375448 | 52383446 | 7999 | ENSG00000154222 | protein_coding | CC2D1B | Exon | 0.00 | down |
| chr16 | 87869356 | 87869507 | 152 | ENSG00000103257 | protein_coding | SLC7A5 | 5' UTR | 0.00 | down |
| chr12 | 65824459 | 65824730 | 272 | ENSG00000149948 | nonsense_mediated_decay | HMGA2 | 5' UTR | 0.00 | up |
| chr11 | 106096817 | 106097176 | 360 | ENSG00000149313 | retained_intron | AASDHPPT | 3' UTR | 0.00 | up |
| chr6 | 136278543 | 136279549 | 1007 | ENSG00000029363 | protein_coding | BCLAF1 | Exon | 0.00 | down |
| chr15 | 44575271 | 44585818 | 10548 | ENSG00000104133 | protein_coding | SPG11 | 3' UTR | 0.00 | down |
| chr13 | 38349848 | 38354470 | 4623 | ENSG00000120686 | retained_intron | UFM1 | 5' UTR | 0.00 | down |
| chr12 | 49324153 | 49324576 | 424 | ENSG00000258334 | lncRNA | AC125611 | Exon | 0.00 | down |
| chr19 | 39839186 | 39846379 | 7194 | ENSG00000105202 | protein_coding | FBL | 3' UTR | 0.00 | down |
| chr14 | 63415173 | 63451412 | 36240 | ENSG00000154001 | processed_transcript | PPP2R5E | Exon | 0.00 | up |
| chr1 | 224386341 | 224386642 | 302 | ENSG00000266618 | miRNA | MIR4742 | 3' UTR | 0.00 | down |
| chr19 | 38406520 | 38406849 | 330 | ENSG00000130244 | retained_intron | FAM98C | Exon | 0.00 | down |
| chr9 | 122890276 | 122897342 | 7067 | ENSG00000056586 | protein_coding | RC3H2 | Exon | 0.00 | down |
| chr12 | 110719769 | 110720128 | 360 | ENSG00000186298 | retained_intron | PPP1CC | 3' UTR | 0.00 | down |
| chr9 | 123401536 | 123411314 | 9779 | ENSG00000119522 | processed_transcript | DENND1A | 3' UTR | 0.00 | down |
| chr1 | 109272884 | 109273233 | 350 | ENSG00000143126 | processed_transcript | CELSR2 | Exon | 0.00 | up |
| chr8 | 123513292 | 123514171 | 880 | ENSG00000156804 | retained_intron | FBXO32 | Exon | 0.00 | down |
| chr15 | 41838793 | 41839153 | 361 | ENSG00000243708 | retained_intron | PLA2G4B | 5' UTR | 0.00 | down |
| chr1 | 1399519 | 1399787 | 269 | ENSG00000224870 | lncRNA | MRPL20-AS1 | Exon | 0.00 | up |
| chr12 | 68660217 | 68660518 | 302 | ENSG00000127314 | retained_intron | RAP1B | 3' UTR | 0.00 | up |
| chr19 | 38907730 | 38908208 | 479 | ENSG00000104825 | nonsense_mediated_decay | NFKBIB | 3' UTR | 0.00 | up |
| chr10 | 5730657 | 5740386 | 9730 | ENSG00000108021 | protein_coding | TASOR2 | Exon | 0.00 | down |
| chr14 | 100369152 | 100370483 | 1332 | ENSG00000140105 | protein_coding | WARS1 | 5' UTR | 0.00 | up |
| chr2 | 237088625 | 237093771 | 5147 | ENSG00000198612 | nonsense_mediated_decay | COPS8 | 3' UTR | 0.00 | down |
| chr1 | 29280124 | 29283762 | 3639 | ENSG00000060656 | retained_intron | PTPRU | Exon | 0.00 | up |
| chr4 | 67635472 | 67646727 | 11256 | ENSG00000033178 | protein_coding | UBA6 | Exon | 0.00 | up |
| chr14 | 100376686 | 100381700 | 5015 | ENSG00000140105 | protein_coding | WARS1 | 5' UTR | 0.00 | down |
| chr1 | 3631005 | 3631515 | 511 | ENSG00000116213 | retained_intron | WRAP73 | 3' UTR | 0.00 | down |
| chr1 | 6210463 | 6210974 | 512 | ENSG00000158286 | processed_transcript | RNF207 | Exon | 0.00 | up |
| chr17 | 45458185 | 45459579 | 1395 | ENSG00000225190 | processed_transcript | PLEKHM1 | 3' UTR | 0.00 | down |
| chr1 | 40861053 | 40861768 | 716 | ENSG00000179862 | protein_coding | CITED4 | 3' UTR | 0.00 | up |
| chr19 | 48630567 | 48631136 | 570 | ENSG00000105516 | protein_coding | DBP | 3' UTR | 0.00 | up |
| chr6 | 7251380 | 7251980 | 601 | ENSG00000287002 | unprocessed_pseudogene | AL589644 | 3' UTR | 0.00 | down |
| chr1 | 183549248 | 183550023 | 776 | ENSG00000116698 | retained_intron | SMG7 | Exon | 0.00 | up |
| chr2 | 206085973 | 206086303 | 331 | ENSG00000114933 | protein_coding | INO80D | 5' UTR | 0.00 | up |
| chr12 | 55822616 | 55823436 | 821 | ENSG00000257390 | nonsense_mediated_decay | AC023055 | Exon | 0.00 | down |
| chr17 | 28395837 | 28396466 | 630 | ENSG00000076351 | retained_intron | SLC46A1 | 3' UTR | 0.00 | down |
| chr9 | 137282342 | 137282641 | 300 | ENSG00000270259 | processed_pseudogene | BX255925 | 3' UTR | 0.00 | down |
| chr16 | 48357345 | 48362522 | 5178 | ENSG00000196470 | processed_transcript | SIAH1 | 3' UTR | 0.00 | down |
| chr20 | 18567405 | 18568801 | 1397 | ENSG00000232388 | protein_coding | SMIM26 | 3' UTR | 0.00 | down |
| chr16 | 30966288 | 30971397 | 5110 | ENSG00000099381 | protein_coding | SETD1A | Exon | 0.00 | up |
| chr14 | 77257254 | 77258300 | 1047 | ENSG00000165548 | retained_intron | TMEM63C | 3' UTR | 0.00 | up |
| chr7 | 44833092 | 44833602 | 511 | ENSG00000105968 | protein_coding | H2AZ2 | 3' UTR | 0.00 | down |
| chr22 | 38670808 | 38681787 | 10980 | ENSG00000228274 | lncRNA | AL021707 | Exon | 0.00 | down |
| chr8 | 145052822 | 145053127 | 306 | ENSG00000182307 | retained_intron | C8orf33 | Exon | 0.00 | up |
| chr5 | 69400159 | 69414622 | 14464 | ENSG00000152939 | protein_coding | MARVELD2 | 3' UTR | 0.00 | down |
| chr15 | 41332646 | 41365468 | 32823 | ENSG00000137804 | processed_transcript | NUSAP1 | 3' UTR | 0.00 | down |
| chr16 | 81695762 | 81696333 | 572 | ENSG00000153815 | TEC | CMIP | Exon | 0.00 | up |
| chr21 | 29067576 | 29070306 | 2731 | ENSG00000156261 | processed_transcript | CCT8 | 5' UTR | 0.00 | up |
| chr14 | 102810733 | 102870422 | 59690 | ENSG00000131323 | protein_coding | TRAF3 | 5' UTR | 0.00 | up |
| chr12 | 32877941 | 32878451 | 511 | ENSG00000057294 | protein_coding | PKP2 | Exon | 0.00 | down |
| chr8 | 120395828 | 120414291 | 18464 | ENSG00000172172 | nonsense_mediated_decay | MRPL13 | 3' UTR | 0.00 | down |
| chr3 | 58564714 | 58565014 | 301 | ENSG00000168309 | protein_coding | FAM107A | 3' UTR | 0.00 | up |
| chr5 | 171391298 | 171391598 | 301 | ENSG00000181163 | retained_intron | NPM1 | 5' UTR | 0.00 | up |
| chr20 | 62284373 | 62284703 | 331 | ENSG00000130703 | retained_intron | OSBPL2 | Exon | 0.00 | down |
| X | 153778636 | 153779346 | 711 | ENSG00000198753 | processed_transcript | PLXNB3 | 3' UTR | 0.00 | up |
| chr3 | 15212134 | 15229043 | 16910 | ENSG00000131375 | protein_coding | CAPN7 | 3' UTR | 0.00 | up |
| chr20 | 38424016 | 38426077 | 2062 | ENSG00000196756 | lncRNA | SNHG17 | Exon | 0.00 | down |
| chr7 | 102569855 | 102571864 | 2010 | ENSG00000168255 | processed_transcript | POLR2J3 | Exon | 0.00 | down |
| chr2 | 241335523 | 241335966 | 444 | ENSG00000168385 | protein_coding | SEPTIN2 | 3' UTR | 0.00 | up |
| chr2 | 169560467 | 169569240 | 8774 | ENSG00000138399 | retained_intron | FASTKD1 | 5' UTR | 0.00 | up |
| chr21 | 34834399 | 34856416 | 22018 | ENSG00000286153 | lncRNA | AP000331 | 3' UTR | 0.00 | down |
| chr1 | 11072096 | 11072337 | 242 | ENSG00000171824 | retained_intron | EXOSC10 | Exon | 0.00 | down |
| chr7 | 5425798 | 5426401 | 604 | ENSG00000272953 | lncRNA | AC092171 | Exon | 0.00 | down |
| chr5 | 180331517 | 180335996 | 4480 | ENSG00000131459 | protein_coding | GFPT2 | 5' UTR | 0.00 | up |
| chr9 | 137554861 | 137555608 | 748 | ENSG00000130653 | protein_coding | PNPLA7 | 3' UTR | 0.00 | down |
| chr19 | 16155727 | 16158515 | 2789 | ENSG00000196684 | nonsense_mediated_decay | HSH2D | 3' UTR | 0.00 | up |
| chr12 | 55822627 | 55823416 | 790 | ENSG00000257390 | nonsense_mediated_decay | AC023055 | Exon | 0.00 | down |
| chr14 | 51254393 | 51254873 | 481 | ENSG00000201376 | snoRNA | SNORA70 | 3' UTR | 0.00 | up |
| chr18 | 8069896 | 8088789 | 18894 | ENSG00000173482 | protein_coding | PTPRM | 5' UTR | 0.00 | up |
| chr19 | 12186632 | 12188072 | 1441 | ENSG00000196646 | retained_intron | ZNF136 | 3' UTR | 0.00 | up |
| chr1 | 117912063 | 117920209 | 8147 | ENSG00000196505 | processed_transcript | GDAP2 | Exon | 0.00 | down |
| chr12 | 122377389 | 122395496 | 18108 | ENSG00000130779 | protein_coding | CLIP1 | 5' UTR | 0.00 | down |
| chr1 | 22913715 | 22914793 | 1079 | ENSG00000133216 | processed_transcript | EPHB2 | 3' UTR | 0.00 | down |
| X | 1403378 | 1418008 | 14631 | ENSG00000236017 | lncRNA | ASMTL-AS1 | Exon | 0.00 | down |
| chr12 | 56332851 | 56333635 | 785 | ENSG00000135473 | retained_intron | PAN2 | 5' UTR | 0.00 | down |
| chr1 | 183551883 | 183552392 | 510 | ENSG00000116698 | processed_transcript | SMG7 | 3' UTR | 0.00 | down |
| chr15 | 41332594 | 41365449 | 32856 | ENSG00000137804 | processed_transcript | NUSAP1 | 3' UTR | 0.00 | down |
| chr7 | 139563068 | 139563489 | 422 | ENSG00000188883 | protein_coding | KLRG2 | 3' UTR | 0.00 | up |
| chr6 | 161136074 | 161144035 | 7962 | ENSG00000026652 | protein_coding | AGPAT4 | 3' UTR | 0.00 | down |
| chr4 | 70827863 | 70833205 | 5343 | ENSG00000132463 | nonsense_mediated_decay | GRSF1 | 3' UTR | 0.00 | down |
| chr7 | 73704018 | 73705419 | 1402 | ENSG00000106089 | processed_transcript | STX1A | Exon | 0.00 | up |
| chr1 | 43650238 | 43653752 | 3515 | ENSG00000066135 | protein_coding | KDM4A | 3' UTR | 0.00 | up |
| chr1 | 149859485 | 149860111 | 627 | ENSG00000270276 | nonsense_mediated_decay | H4C15 | 3' UTR | 0.00 | up |
| chr7 | 99388822 | 99389379 | 558 | ENSG00000130429 | retained_intron | ARPC1B | 3' UTR | 0.00 | down |
| chr12 | 49064675 | 49065423 | 749 | ENSG00000167548 | protein_coding | KMT2D | 3' UTR | 0.00 | up |
| chr22 | 37618486 | 37619950 | 1465 | ENSG00000100083 | retained_intron | GGA1 | 3' UTR | 0.00 | up |
| chr17 | 28400707 | 28402259 | 1553 | ENSG00000076351 | retained_intron | SLC46A1 | Exon | 0.00 | down |
| chr7 | 75297405 | 75297727 | 323 | ENSG00000267828 | misc_RNA | Y_RNA | Exon | 0.00 | up |
| chr6 | 36110007 | 36110757 | 751 | ENSG00000156711 | processed_transcript | MAPK13 | 3' UTR | 0.00 | down |
| chr12 | 43795455 | 43802361 | 6907 | ENSG00000151239 | retained_intron | TWF1 | 3' UTR | 0.00 | down |
| chr17 | 79044041 | 79047635 | 3595 | ENSG00000173918 | processed_transcript | C1QTNF1 | 5' UTR | 0.00 | down |
| chr11 | 103083287 | 103091684 | 8398 | ENSG00000137692 | protein_coding | DCUN1D5 | 3' UTR | 0.00 | up |
| chr4 | 2881878 | 2884603 | 2726 | ENSG00000087274 | retained_intron | ADD1 | 3' UTR | 0.00 | down |
| chr17 | 1011659 | 1012113 | 455 | ENSG00000159842 | protein_coding | ABR | 3' UTR | 0.00 | down |
| chr9 | 131626008 | 131628007 | 2000 | ENSG00000107263 | protein_coding | RAPGEF1 | Exon | 0.00 | down |
| chr2 | 201138137 | 201140027 | 1891 | ENSG00000232133 | processed_pseudogene | IMPDH1P10 | Exon | 0.00 | up |
| X | 119538415 | 119539009 | 595 | ENSG00000018610 | processed_transcript | CXorf56 | 3' UTR | 0.00 | down |
| chr9 | 36162415 | 36163344 | 930 | ENSG00000185972 | protein_coding | CCIN | 3' UTR | 0.00 | down |
| chr11 | 83157197 | 83157557 | 361 | ENSG00000165494 | TEC | PCF11 | 5' UTR | 0.00 | down |
| chr2 | 91636728 | 91654920 | 18193 | ENSG00000143429 | processed_transcript | LSP1P4 | Exon | 0.00 | down |
| chr2 | 232336440 | 232340775 | 4336 | ENSG00000144535 | protein_coding | DIS3L2 | 3' UTR | 0.00 | down |
| chr17 | 50751522 | 50752122 | 601 | ENSG00000108848 | processed_transcript | LUC7L3 | 3' UTR | 0.00 | down |
| chr14 | 35123111 | 35127483 | 4373 | ENSG00000100890 | protein_coding | PRORP | 5' UTR | 0.00 | down |
| chr1 | 151291456 | 151291936 | 481 | ENSG00000143373 | protein_coding | ZNF687 | 3' UTR | 0.00 | up |
| chr7 | 155706841 | 155707367 | 527 | ENSG00000184863 | processed_transcript | RBM33 | 3' UTR | 0.00 | up |
| chr11 | 2400606 | 2401155 | 550 | ENSG00000184281 | protein_coding | TSSC4 | 5' UTR | 0.00 | down |
| chr8 | 140252756 | 140262674 | 9919 | ENSG00000167632 | processed_transcript | TRAPPC9 | Exon | 0.00 | up |
| chr11 | 46312404 | 46315211 | 2808 | ENSG00000157613 | processed_transcript | CREB3L1 | Exon | 0.00 | up |
| chr12 | 62467056 | 62470739 | 3684 | ENSG00000061987 | nonsense_mediated_decay | MON2 | 3' UTR | 0.00 | up |
| chr19 | 16495806 | 16553896 | 58091 | ENSG00000085872 | protein_coding | CHERP | 3' UTR | 0.00 | up |
| chr1 | 171591622 | 171592492 | 871 | ENSG00000117523 | processed_transcript | PRRC2C | 3' UTR | 0.00 | up |
| chr1 | 205894464 | 205894854 | 391 | ENSG00000286619 | lncRNA | AC119673 | Exon | 0.00 | up |
| chr19 | 58246359 | 58285924 | 39566 | ENSG00000198131 | processed_transcript | ZNF544 | 3' UTR | 0.00 | up |
| chr14 | 102036944 | 102037394 | 451 | ENSG00000197102 | retained_intron | DYNC1H1 | 3' UTR | 0.00 | down |
| chr14 | 103712899 | 103713319 | 421 | ENSG00000100711 | protein_coding | ZFYVE21 | 3' UTR | 0.00 | down |
| chr9 | 121821966 | 121822860 | 895 | ENSG00000175764 | processed_transcript | TTLL11 | 3' UTR | 0.00 | down |
| chr17 | 2030139 | 2033542 | 3404 | ENSG00000108963 | protein_coding | DPH1 | 5' UTR | 0.00 | up |
| chr15 | 64859925 | 64921987 | 62063 | ENSG00000166839 | processed_transcript | ANKDD1A | 3' UTR | 0.00 | up |
| chr22 | 20145758 | 20146479 | 722 | ENSG00000099904 | retained_intron | ZDHHC8 | 3' UTR | 0.00 | up |
| chr11 | 45917784 | 45918812 | 1029 | ENSG00000121680 | protein_coding | PEX16 | 5' UTR | 0.00 | up |
| chr7 | 102568097 | 102572564 | 4468 | ENSG00000168255 | nonsense_mediated_decay | POLR2J3 | 3' UTR | 0.00 | down |
| chr19 | 1632104 | 1650198 | 18095 | ENSG00000071564 | protein_coding | TCF3 | Exon | 0.00 | down |
| chr19 | 39314040 | 39315264 | 1225 | ENSG00000128011 | protein_coding | LRFN1 | Exon | 0.00 | down |
| X | 110174057 | 110174387 | 331 | ENSG00000157600 | processed_transcript | TMEM164 | 3' UTR | 0.00 | up |
| chr2 | 190989609 | 191007613 | 18005 | ENSG00000229023 | processed_pseudogene | RAB1AP1 | 3' UTR | 0.00 | up |
| chr20 | 43642450 | 43647150 | 4701 | ENSG00000101052 | processed_transcript | IFT52 | 3' UTR | 0.00 | up |
| chr2 | 118088451 | 118102636 | 14186 | ENSG00000125629 | nonsense_mediated_decay | INSIG2 | 5' UTR | 0.00 | down |
| chr11 | 9779325 | 9780256 | 932 | ENSG00000133812 | retained_intron | SBF2 | 3' UTR | 0.00 | down |
| chr19 | 17826721 | 17829958 | 3238 | ENSG00000248099 | protein_coding | INSL3 | 3' UTR | 0.00 | down |
| chr19 | 49981690 | 49986430 | 4741 | ENSG00000105053 | retained_intron | VRK3 | 3' UTR | 0.00 | down |
| chr3 | 15084818 | 15091390 | 6573 | ENSG00000131381 | retained_intron | RBSN | 3' UTR | 0.00 | up |
| chr5 | 81419993 | 81420383 | 391 | ENSG00000286721 | lncRNA | AC010623 | 3' UTR | 0.00 | down |
| chr17 | 35001936 | 35002949 | 1014 | ENSG00000005156 | retained_intron | LIG3 | Exon | 0.00 | up |
| chr7 | 116796861 | 116797221 | 361 | ENSG00000198898 | protein_coding | CAPZA2 | 3' UTR | 0.00 | down |
| chr16 | 88625674 | 88626184 | 511 | ENSG00000158545 | retained_intron | ZC3H18 | Exon | 0.00 | down |
| chr5 | 177971783 | 177972106 | 324 | ENSG00000249129 | processed_pseudogene | SUDS3P1 | Exon | 0.00 | down |
| chr11 | 93784628 | 93794942 | 10315 | ENSG00000042429 | nonsense_mediated_decay | MED17 | 3' UTR | 0.00 | down |
| chr2 | 170949647 | 170950116 | 470 | ENSG00000115806 | retained_intron | GORASP2 | 3' UTR | 0.00 | up |
| chr9 | 129822545 | 129822845 | 301 | ENSG00000136827 | processed_transcript | TOR1A | Exon | 0.00 | up |
| chr2 | 239960924 | 239961075 | 152 | ENSG00000130414 | processed_transcript | NDUFA10 | 3' UTR | 0.00 | up |
| chr7 | 73704081 | 73704472 | 392 | ENSG00000106089 | processed_transcript | STX1A | 3' UTR | 0.00 | up |
| chr10 | 133262509 | 133262959 | 451 | ENSG00000151651 | retained_intron | ADAM8 | 3' UTR | 0.00 | up |
| chr1 | 96679291 | 96679620 | 330 | ENSG00000137970 | processed_pseudogene | RPL7P9 | Exon | 0.00 | down |
| chr1 | 146984944 | 146992740 | 7797 | ENSG00000268043 | protein_coding | NBPF12 | 3' UTR | 0.00 | up |
| chr10 | 27154606 | 27155206 | 601 | ENSG00000120539 | protein_coding | MASTL | 5' UTR | 0.00 | up |
| chr19 | 3595309 | 3595697 | 389 | ENSG00000006638 | processed_transcript | TBXA2R | 3' UTR | 0.00 | down |
| chr14 | 35053289 | 35054766 | 1478 | ENSG00000151327 | protein_coding | FAM177A1 | 3' UTR | 0.00 | down |
| chr1 | 147647351 | 147647681 | 331 | ENSG00000162836 | retained_intron | ACP6 | 3' UTR | 0.00 | down |
| chr1 | 20649419 | 20649748 | 330 | ENSG00000117242 | lncRNA | PINK1-AS | Exon | 0.00 | down |
| chr14 | 60149333 | 60150099 | 767 | ENSG00000100612 | protein_coding | DHRS7 | Exon | 0.00 | down |
| chr17 | 7509902 | 7511576 | 1675 | ENSG00000181222 | retained_intron | POLR2A | Exon | 0.00 | up |
| chr7 | 102566713 | 102592444 | 25732 | ENSG00000105808 | protein_coding | RASA4 | 3' UTR | 0.00 | up |
| chr3 | 38134504 | 38136882 | 2379 | ENSG00000060971 | protein_coding | ACAA1 | Exon | 0.00 | down |
| chr7 | 6655824 | 6656454 | 631 | ENSG00000228010 | lncRNA | AC073343 | 3' UTR | 0.00 | up |
| chr4 | 74174529 | 74175330 | 802 | ENSG00000163738 | processed_transcript | MTHFD2L | 5' UTR | 0.00 | down |
| chr17 | 45284935 | 45287274 | 2340 | ENSG00000006062 | protein_coding | MAP3K14 | Exon | 0.00 | up |
| chr10 | 27154823 | 27155212 | 390 | ENSG00000120539 | protein_coding | MASTL | 5' UTR | 0.00 | up |
| chr15 | 85205790 | 85208607 | 2818 | ENSG00000229212 | transcribed_unprocessed_pseudogene | AC044860 | Exon | 0.00 | up |
| chr2 | 206085790 | 206086475 | 686 | ENSG00000114933 | protein_coding | INO80D | Exon | 0.00 | up |
| chr16 | 80633216 | 80735133 | 101918 | ENSG00000166446 | processed_transcript | CDYL2 | Exon | 0.00 | down |
| chr9 | 35237723 | 35291074 | 53352 | ENSG00000198722 | processed_transcript | UNC13B | 5' UTR | 0.00 | up |
| chr1 | 206684904 | 206685322 | 419 | ENSG00000162889 | protein_coding | MAPKAPK2 | 5' UTR | 0.00 | down |
| chr1 | 244856483 | 244857006 | 524 | ENSG00000153187 | nonsense_mediated_decay | HNRNPU | 3' UTR | 0.00 | up |
| chr9 | 111669318 | 111669494 | 177 | ENSG00000242616 | protein_coding | GNG10 | 3' UTR | 0.00 | up |
| chr5 | 151662322 | 151662713 | 392 | ENSG00000113140 | processed_transcript | SPARC | 3' UTR | 0.00 | up |
| chr12 | 57749705 | 57750501 | 797 | ENSG00000135446 | retained_intron | CDK4 | 3' UTR | 0.00 | down |
| chr9 | 111661604 | 111666880 | 5277 | ENSG00000242616 | protein_coding | GNG10 | 5' UTR | 0.00 | up |
| chr5 | 179621315 | 179622944 | 1630 | ENSG00000169045 | nonsense_mediated_decay | HNRNPH1 | 3' UTR | 0.00 | down |
| chr12 | 12915003 | 12915303 | 301 | ENSG00000283759 | miRNA | MIR614 | 3' UTR | 0.00 | down |
| chr16 | 30894570 | 30894960 | 391 | ENSG00000099385 | protein_coding | BCL7C | 5' UTR | 0.00 | up |
| chr6 | 33271577 | 33271927 | 351 | ENSG00000223501 | protein_coding | VPS52 | 5' UTR | 0.00 | up |
| chr14 | 69885339 | 69885607 | 269 | ENSG00000242071 | processed_pseudogene | RPL7AP6 | Exon | 0.00 | up |
| chr14 | 53949914 | 53951431 | 1518 | ENSG00000125378 | protein_coding | BMP4 | 3' UTR | 0.00 | down |
| chr17 | 37440579 | 37444704 | 4126 | ENSG00000276234 | nonsense_mediated_decay | TADA2A | 3' UTR | 0.00 | up |
| chr17 | 75889567 | 75890018 | 452 | ENSG00000141569 | protein_coding | TRIM65 | 3' UTR | 0.00 | down |
| chr5 | 6372346 | 6374394 | 2049 | ENSG00000133398 | retained_intron | MED10 | 3' UTR | 0.00 | down |
| chr2 | 37204262 | 37204503 | 242 | ENSG00000272054 | lncRNA | AC007390 | 3' UTR | 0.00 | down |
| chr2 | 170950205 | 170951602 | 1398 | ENSG00000115806 | retained_intron | GORASP2 | 3' UTR | 0.00 | up |
| chr6 | 24528029 | 24534338 | 6310 | ENSG00000112294 | retained_intron | ALDH5A1 | 3' UTR | 0.00 | down |
| chr7 | 27141614 | 27142729 | 1116 | ENSG00000106004 | processed_transcript | HOXA5 | 3' UTR | 0.00 | down |
| chr16 | 89689859 | 89690130 | 272 | ENSG00000185324 | retained_intron | CDK10 | Exon | 0.00 | down |
| chr5 | 149551201 | 149551471 | 271 | ENSG00000113712 | protein_coding | CSNK1A1 | 5' UTR | 0.00 | up |
| chr13 | 95985267 | 95990274 | 5008 | ENSG00000102595 | processed_transcript | UGGT2 | 3' UTR | 0.00 | down |
| chr22 | 18089816 | 18090117 | 302 | ENSG00000215193 | protein_coding | PEX26 | 3' UTR | 0.00 | down |
| chr1 | 35566211 | 35566572 | 362 | ENSG00000020129 | protein_coding | NCDN | 3' UTR | 0.00 | down |
| chr3 | 172807718 | 172818498 | 10781 | ENSG00000114346 | retained_intron | ECT2 | Exon | 0.00 | down |
| X | 293205 | 300339 | 7135 | ENSG00000182378 | protein_coding | PLCXD1 | 3' UTR | 0.00 | down |
| chr3 | 108071136 | 108090882 | 19747 | ENSG00000196776 | protein_coding | CD47 | 5' UTR | 0.00 | down |
| chr1 | 161158239 | 161158856 | 618 | ENSG00000143258 | protein_coding | USP21 | 3' UTR | 0.00 | up |
| chr16 | 88717926 | 88718077 | 152 | ENSG00000103335 | protein_coding | PIEZO1 | Exon | 0.00 | down |
| chr14 | 22611090 | 22611270 | 181 | ENSG00000100439 | processed_transcript | ABHD4 | 3' UTR | 0.00 | down |
| chr7 | 99394455 | 99394816 | 362 | ENSG00000130429 | processed_transcript | ARPC1B | 3' UTR | 0.00 | down |
| chr3 | 20153654 | 20154314 | 661 | ENSG00000114166 | retained_intron | KAT2B | 3' UTR | 0.00 | up |
| chr9 | 83970714 | 83971092 | 379 | ENSG00000165119 | retained_intron | HNRNPK | Exon | 0.00 | down |
| chr17 | 58210844 | 58215528 | 4685 | ENSG00000011143 | protein_coding | MKS1 | 3' UTR | 0.00 | down |
| chr5 | 139284448 | 139284689 | 242 | ENSG00000200959 | snoRNA | SNORA74A | Exon | 0.00 | down |
| chr17 | 7889666 | 7893291 | 3626 | ENSG00000170004 | processed_transcript | CHD3 | 5' UTR | 0.00 | up |
| chr1 | 35601998 | 35602329 | 332 | ENSG00000239636 | lncRNA | AC004865 | 3' UTR | 0.00 | down |
| chr22 | 37565699 | 37566587 | 889 | ENSG00000128283 | protein_coding | CDC42EP1 | 5' UTR | 0.00 | down |
| chr7 | 76099453 | 76108779 | 9327 | ENSG00000227038 | processed_transcript | GTF2IP7 | Exon | 0.00 | down |
| chr18 | 50282342 | 50282703 | 362 | ENSG00000141644 | protein_coding | MBD1 | 3' UTR | 0.00 | up |
| chr15 | 89333165 | 89333586 | 422 | ENSG00000140521 | nonsense_mediated_decay | POLG | Exon | 0.00 | down |
| chr16 | 28866510 | 28867289 | 780 | ENSG00000178188 | protein_coding | SH2B1 | 5' UTR | 0.00 | down |
| X | 46573824 | 46575082 | 1259 | ENSG00000147119 | protein_coding | CHST7 | 5' UTR | 0.00 | down |
| chr16 | 89739277 | 89740895 | 1619 | ENSG00000187741 | protein_coding | FANCA | 3' UTR | 0.00 | down |
| chr7 | 101090905 | 101092282 | 1378 | ENSG00000169871 | processed_transcript | TRIM56 | 3' UTR | 0.00 | down |
| chr11 | 72696976 | 72697635 | 660 | ENSG00000186635 | retained_intron | ARAP1 | Exon | 0.00 | down |
| chr3 | 49424136 | 49424827 | 692 | ENSG00000283189 | retained_intron | AC104452 | 3' UTR | 0.00 | down |
| chr10 | 74600395 | 74675940 | 75546 | ENSG00000156110 | processed_transcript | ADK | 3' UTR | 0.00 | down |
| chr12 | 110030708 | 110037843 | 7136 | ENSG00000076513 | retained_intron | ANKRD13A | 3' UTR | 0.00 | down |
| chr19 | 12792353 | 12872740 | 80388 | ENSG00000095066 | processed_transcript | HOOK2 | 3' UTR | 0.00 | down |
| chr1 | 168003934 | 168015884 | 11951 | ENSG00000143164 | processed_transcript | DCAF6 | Exon | 0.00 | up |
| chr1 | 2546812 | 2546989 | 178 | ENSG00000272449 | lncRNA | AL139246 | Exon | 0.00 | down |
| chr9 | 111669313 | 111669491 | 179 | ENSG00000242616 | protein_coding | GNG10 | 3' UTR | 0.00 | up |
| chr18 | 50286053 | 50286931 | 879 | ENSG00000154832 | retained_intron | CXXC1 | Exon | 0.00 | up |
| chr7 | 102637143 | 102637353 | 211 | ENSG00000267368 | protein_coding | UPK3BL1 | 3' UTR | 0.00 | down |
| chr4 | 2639842 | 2654525 | 14684 | ENSG00000125386 | protein_coding | FAM193A | 5' UTR | 0.00 | down |
| chr19 | 49090308 | 49101550 | 11243 | ENSG00000104852 | retained_intron | SNRNP70 | 5' UTR | 0.00 | up |
| chr3 | 49424147 | 49424833 | 687 | ENSG00000283189 | retained_intron | AC104452 | 3' UTR | 0.00 | down |
| chr6 | 33195957 | 33196198 | 242 | ENSG00000204231 | retained_intron | RXRB | 3' UTR | 0.00 | up |
| chr9 | 14474 | 14891 | 418 | ENSG00000181404 | protein_coding | WASHC1 | 3' UTR | 0.00 | up |
| chr12 | 121776769 | 121777249 | 481 | ENSG00000188735 | processed_transcript | TMEM120B | 3' UTR | 0.00 | down |
| chr16 | 3850330 | 3850991 | 662 | ENSG00000005339 | processed_transcript | CREBBP | Exon | 0.00 | down |
| chr16 | 89530708 | 89530949 | 242 | ENSG00000197912 | retained_intron | SPG7 | 3' UTR | 0.00 | up |
| X | 153778643 | 153779292 | 650 | ENSG00000198753 | processed_transcript | PLXNB3 | 3' UTR | 0.00 | up |
| chr17 | 45425612 | 45429783 | 4172 | ENSG00000159314 | protein_coding | ARHGAP27 | 3' UTR | 0.00 | up |
| chr3 | 105685448 | 105720101 | 34654 | ENSG00000114423 | nonsense_mediated_decay | CBLB | 3' UTR | 0.00 | up |
| chr3 | 192797646 | 192798093 | 448 | ENSG00000114279 | protein_coding | FGF12 | 3' UTR | 0.00 | down |
| chr2 | 105307684 | 105308313 | 630 | ENSG00000135966 | protein_coding | TGFBRAP1 | 5' UTR | 0.00 | down |
| chr20 | 36781908 | 36783017 | 1110 | ENSG00000149636 | protein_coding | DSN1 | 3' UTR | 0.00 | down |
| chr3 | 101782078 | 101816814 | 34737 | ENSG00000144815 | protein_coding | NXPE3 | 5' UTR | 0.00 | down |
| chr9 | 137110545 | 137110754 | 210 | ENSG00000176978 | nonsense_mediated_decay | DPP7 | 3' UTR | 0.00 | up |
| chr20 | 56373450 | 56384311 | 10862 | ENSG00000087586 | protein_coding | AURKA | Exon | 0.00 | up |
| chr17 | 63622414 | 63666959 | 44546 | ENSG00000198909 | protein_coding | MAP3K3 | 3' UTR | 0.00 | up |
| chr11 | 57325658 | 57327958 | 2301 | ENSG00000254662 | lncRNA | AP000781 | Exon | 0.00 | up |
| chr7 | 102567071 | 102567132 | 62 | ENSG00000168255 | processed_transcript | POLR2J3 | 3' UTR | 0.00 | up |
| chr1 | 12618899 | 12619215 | 317 | ENSG00000272482 | lncRNA | AC254633 | Exon | 0.00 | up |
| chr19 | 6377027 | 6377536 | 510 | ENSG00000125650 | protein_coding | PSPN | 5' UTR | 0.00 | down |
| chr2 | 25116022 | 25130072 | 14051 | ENSG00000084710 | protein_coding | EFR3B | 5' UTR | 0.00 | down |
| X | 63349983 | 63351332 | 1350 | ENSG00000186767 | protein_coding | SPIN4 | 3' UTR | 0.00 | up |
| chr12 | 57749681 | 57750159 | 479 | ENSG00000135446 | retained_intron | CDK4 | 3' UTR | 0.00 | down |
| chr3 | 113469200 | 113515091 | 45892 | ENSG00000163611 | protein_coding | SPICE1 | 5' UTR | 0.00 | down |
| chr16 | 57456940 | 57457121 | 182 | ENSG00000088682 | retained_intron | COQ9 | Exon | 0.00 | down |
| chr20 | 3827614 | 3828092 | 479 | ENSG00000125843 | protein_coding | AP5S1 | 3' UTR | 0.00 | down |
| chr18 | 5395108 | 5397208 | 2101 | ENSG00000082397 | protein_coding | EPB41L3 | 3' UTR | 0.00 | up |
| chr2 | 70288034 | 70292794 | 4761 | ENSG00000143977 | protein_coding | SNRPG | 5' UTR | 0.00 | down |
| chr4 | 38938681 | 38944215 | 5535 | ENSG00000197712 | processed_transcript | FAM114A1 | 3' UTR | 0.00 | up |
| chr3 | 48693763 | 48694153 | 391 | ENSG00000068745 | retained_intron | IP6K2 | 3' UTR | 0.00 | down |
| chr14 | 102722317 | 102727327 | 5011 | ENSG00000089902 | nonsense_mediated_decay | RCOR1 | 3' UTR | 0.00 | down |
| chr12 | 68659378 | 68659739 | 362 | ENSG00000127314 | retained_intron | RAP1B | 3' UTR | 0.00 | up |
| chr22 | 21956999 | 21957450 | 452 | ENSG00000224086 | lncRNA | PPM1F-AS1 | Exon | 0.00 | up |
| chr3 | 53224831 | 53225491 | 661 | ENSG00000163931 | retained_intron | TKT | 3' UTR | 0.00 | down |
| chr7 | 102637107 | 102637345 | 239 | ENSG00000267368 | protein_coding | UPK3BL1 | 3' UTR | 0.00 | down |
| chr12 | 56666207 | 56671778 | 5572 | ENSG00000241217 | misc_RNA | RN7SL809P | Exon | 0.00 | up |
| chr1 | 28236435 | 28236673 | 239 | ENSG00000130770 | protein_coding | ATP5IF1 | 3' UTR | 0.00 | up |
| chr19 | 17825526 | 17826423 | 898 | ENSG00000248099 | protein_coding | INSL3 | 3' UTR | 0.00 | down |
| chr19 | 58246304 | 58247382 | 1079 | ENSG00000198131 | protein_coding | ZNF544 | 3' UTR | 0.00 | up |
| chr22 | 21957024 | 21957444 | 421 | ENSG00000100034 | protein_coding | PPM1F | 3' UTR | 0.00 | up |
| chr19 | 18446788 | 18450961 | 4174 | ENSG00000105656 | retained_intron | ELL | 3' UTR | 0.00 | down |
| chr4 | 6923437 | 6987206 | 63770 | ENSG00000132405 | protein_coding | TBC1D14 | 5' UTR | 0.00 | up |
| chr2 | 46378000 | 46380099 | 2100 | ENSG00000116016 | retained_intron | EPAS1 | Exon | 0.00 | down |
| chr1 | 28237532 | 28246639 | 9108 | ENSG00000270605 | lncRNA | AL353622 | 3' UTR | 0.00 | up |
| chr1 | 146064830 | 146065101 | 272 | ENSG00000201558 | snRNA | RNVU1-6 | 3' UTR | 0.00 | up |
| chr12 | 2794969 | 2795710 | 742 | ENSG00000004478 | protein_coding | FKBP4 | 5' UTR | 0.00 | up |
| chr7 | 102541257 | 102541515 | 259 | ENSG00000284981 | protein_coding | UPK3BL2 | 3' UTR | 0.00 | up |
| chr14 | 21385193 | 21386333 | 1141 | ENSG00000100888 | retained_intron | CHD8 | 3' UTR | 0.00 | up |
| chr15 | 85209205 | 85209685 | 481 | ENSG00000259295 | processed_transcript | CSPG4P12 | Exon | 0.00 | down |
| chr22 | 29359824 | 29419166 | 59343 | ENSG00000100280 | protein_coding | AP1B1 | 5' UTR | 0.00 | up |
| chr1 | 25818848 | 25820498 | 1651 | ENSG00000117640 | protein_coding | MTFR1L | 5' UTR | 0.00 | down |
| X | 129504774 | 129511847 | 7074 | ENSG00000102038 | processed_transcript | SMARCA1 | Exon | 0.00 | down |
| chr1 | 25819928 | 25820498 | 571 | ENSG00000117640 | protein_coding | MTFR1L | Exon | 0.00 | down |
| chr5 | 6604091 | 6604689 | 599 | ENSG00000037474 | retained_intron | NSUN2 | 3' UTR | 0.00 | up |
| chr6 | 32175818 | 32177112 | 1295 | ENSG00000204310 | protein_coding | AGPAT1 | 5' UTR | 0.00 | up |
[truncated: 67,797 more chars]
